# Synthesis, DFT investigations, antioxidant, antibacterial activity and SAR-study of novel thiophene-2-carboxamide derivatives

**DOI:** 10.1186/s13065-023-00917-2

**Published:** 2023-02-20

**Authors:** Heba M. Metwally, Norhan A. Khalaf, Ehab Abdel-Latif, Mohamed A. Ismail

**Affiliations:** Department of Chemistry, Faculty of Science, Mansoura University, Mansoura, 35516 Egypt

**Keywords:** 2-Chloroacetamide, Thiophene-2-carboxamide, Antioxidant, DFT calculations, Molecular docking

## Abstract

Synthetic strategy for the synthesis of thiophene 2-carboxamide derivatives substituted with hydroxyl, methyl and amino groups at position-3 was proposed. The strategy includes the cyclization of the precursor ethyl 2-arylazo-3-mercapto-3-(phenylamino)acrylate derivatives, 2-acetyl-2-arylazo-thioacetanilide derivatives and *N*-aryl-2-cyano-3-mercapto-3-(phenylamino)acrylamide derivatives with *N*-(4-acetylphenyl)-2-chloroacetamide in alcoholic sodium ethoxide. IR, ^1^H NMR, and mass spectroscopic analyses were used to characterize the synthesized derivatives. In addition, molecular, electronic properties of the synthesized products were studied by the density functional theory (DFT) where they exhibited close HOMO–LUMO energy gap (ΔE_H-L_) in which the amino derivatives **7a-c** have the highest while the methyl derivatives **5a-c** were the lowest. Using the ABTS method, the antioxidant properties of the produced compounds were evaluated, where amino thiophene-2-carboxamide **7a** exhibit significant inhibition activity 62.0% compared to ascorbic acid The antibacterial activity against two pathogenic Gram-positive bacteria (*Staphylococcus aureus* and *Bacillus subtilis*) and two of pathogenic Gram-negative bacteria (*Escherichia coli* and *Pseudomonas aeruginosa*) revealed that **7b** records the highest activity index compared to ampicillin 83.3, 82.6, 64.0, 86.9%, respectively. Furthermore, the thiophene-2-carboxamide derivatives were docked with five different proteins with the use molecular docking tools and the results explained interactions between amino acid residue of enzyme and compounds. Compounds **3b** and **3c** showed the highest binding score with 2AS1 protein.

**Graphical Abstract:**

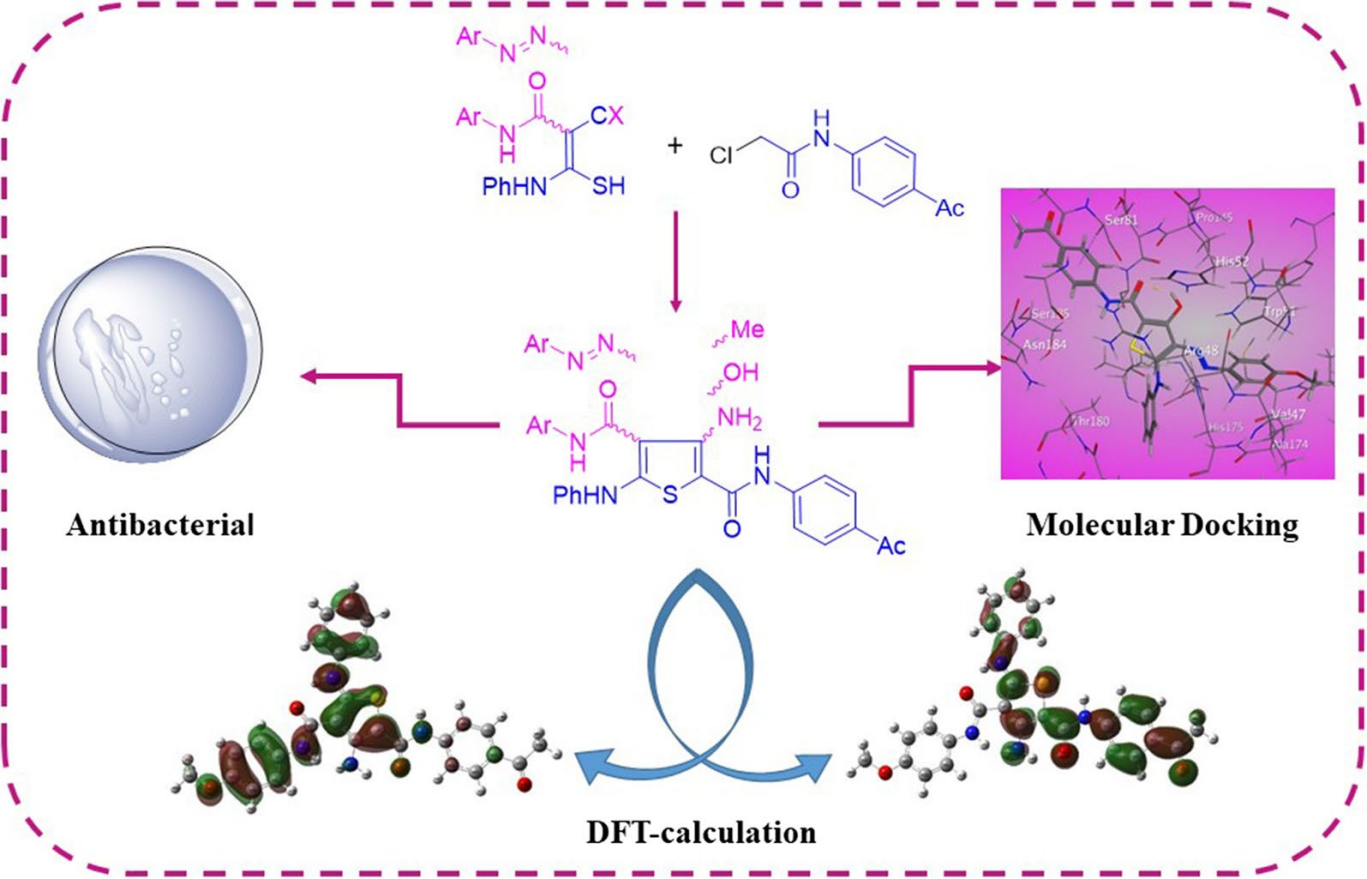

**Supplementary Information:**

The online version contains supplementary material available at 10.1186/s13065-023-00917-2.

## Introduction

**Fig. 1:**
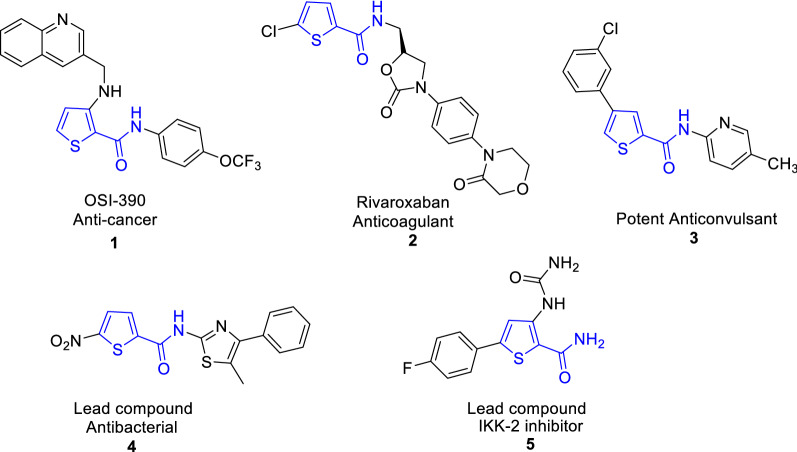
Versatile thiophene 2-carboxamide derivatives

The antibacterial resistance threatens the human health around the world. It appeared due to the human abuse of antibiotics and it reached to danger limit. In 2019, 1.27 out of 4.95 million deaths attributed to antimicrobial resistance, with three leading pathogens for deaths (*E. coli*, *S. aureus*, *P. aeuroginosa*) [1]. The researchers continue their efforts for exploration for more powerful drugs with some requirements that involves, the molecular size of a drug's affinity for its target, drug bio-activation and metabolization. They must also address the design of medications with fewer adverse effects and more desirable small-molecular drug characteristics than existing drugs [2]. Recently, thiosemicarbazone and thio/carbohydrazone grabbed attention due to their potential biological activities [3, 4]. Many five-membered heterocyclic rings were extensively reported due to their chemical properties and versatile biological activities. It is known that heterocyclic compounds containing sulfur in their structures are used widely to eliminate free radicals and stop the antimicrobial resistance in addition to other pharmaceutical applications [5–7]. Generally, thiophenes have wide applications in different fields such as solid-state electrochromic devices [8], industry and medicinal chemistry. They possess biological properties such as antioxidant [9–16], antibacterial [17–19], antifungal [20–22], inflammatory [23] and antitumor [24–26]. Some examples of thiophene 2-carboxamide are marketed drugs, for example OSI-390 is used as anticancer drug (Fig. 1) [27, 28] and Rivaroxaban is used as an antithrombotic agent (Fig. 1) [29]. Moreover, compound 3 was found to have potent anticonvulsive effects in BALB-C mice [30]. Additionally, thiophene-2-carboxamide was considered to be a lead compound for drug discovery [31, 32]; for example; nitro thiophene-2-carboxamide **4** was used as a narrow spectrum antibacterial lead compound [33] and thiophene-2-carboxamide 5 was used as IKK-2 potent lead inhibitor [34]. Previous DFT-study for thiophene-2-carboxamide derivatives showed that *N*-(thiophen-2-ylmethyl)thiophene-2-carboxamide displays close experimental and theoretical structure parameters with (ΔE_H-L_) 5.031 eV [35]. While thiophene-thiadiazole hybrid derivatives (FMOs) shows close values between 3.83 and 4.18 eV [36]. As a result, many methods for the preparation of thiophene derivatives have been published either by incorporating thiophene moiety or by construction of the ring. One of the most efficient synthetic strategy includes cyclization of valuable thiocarbamoyl derivatives with α-halogenated reagents. A variety of advantages of this unique synthetic strategy includes the synthesis of a variety polysubstituted thiophenes, easy workup and cleaner products. As a result of previous advantages, we are attracted to explore this method. In light of the aforementioned bacterial resistance findings, DFT- studies and in accordance with the current research focus on preparing bioactive substituted thiophenes, the main goal of this work is to synthesize 3-substituted thiopene 2-carboxamide derivatives decorated with different substituents Cl, OMe, Me, NH_2_, OH groups aiming at increasing anti-oxidant and antibacterial inhibition power.

## Result and discussion

### Chemistry

**Scheme 1:**
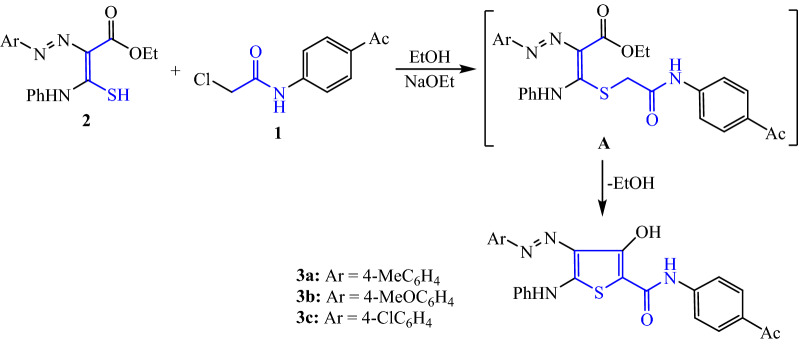
Synthesis of 3-hydroxy thiophene-2-carboxamides **3a–c**

The synthesis of our target thiophene 2-carboxamide derivatives **3**, **5** and **7** was aimed as one-step method by condensation of *N*-(4-acetylphenyl)-2-chloroacetamide (**1**) and various functionalized thiocarbamoyl compounds **2**, **4**, and **6**, respectively. The highly versatile chloroacetamide compound, *N*-(4-acetylphenyl)-2-chloroacetamide (**1**) has been prepared by the reported chloroacetylation reaction of 4-aminoacetopheone [37]. Initially, 2-chloroacetamide derivative **1** was reacted with ethyl 2-arylazo-3-mercapto-3-(phenylamino)acrylate derivatives (**2**) [38] in ethanolic sodium ethoxide which resulted in the formation of sulfide intermediate **A** (Scheme 1). Substitution of the chlorine atom from the chloroacetamide derivative **1** by the 3-mercaptoacrylate reagent **2** led to the formation of the intermediate **A**. Subsequently, ethanol is removed intramolecularly, resulting in intermediate A, which is then used to yield the 3-hydroxythiophene **3a-c**. Using IR data and ^1^H NMR, the structures of all of the newly synthesized compounds were analyzed, and the results were completely consistent with the assigned molecular structures.

**Scheme 2:**
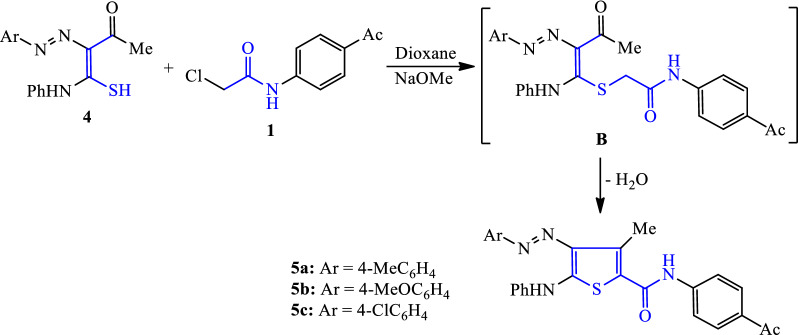
Synthesis of 3-methylthiophene-2-carboxamide derivatives **5a–c**

Based upon the above successful one step method for preparation of 4-arylazo-3-hydroxy thiophene derivatives, synthesis of our targeted thiophene derivatives **5a-c** was performed. Thus, reaction of *N*-(4-acetylphenyl)-2-chloroacetamide (**1**) with 2-acetyl-2-arylazo-thioacetanilide derivatives (**4**) [39] in boiling dioxane containing sodium methoxide gave the corresponding 4-arylazo-3-methyl-thiophene derivatives **5a–c** (Scheme 2). The mechanistic reaction scenario proceeded via the formation of intermediate **B**, which was followed by heterocyclization of the compound via the nucleophilic addition of the methylene group to the carbonyl function, and terminated with the removal of the water molecule (Scheme 2). The structures of the entire newly synthesized compound were validated using IR and ^1^H NMR data, which confirmed the molecular structures assigned.

**Scheme 3:**
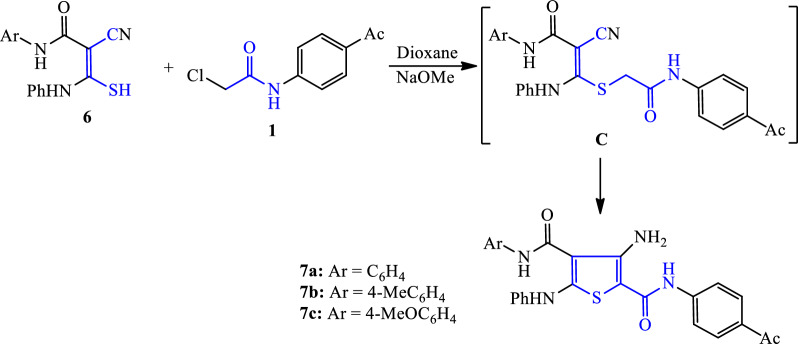
Synthesis of 3-aminothiophene-2-carboxamides derivatives **7a–c**

Similarly, the reaction of 2-chloroacetamide reagent **1** with *N*-aryl-2-cyano-3-mercapto-3-(phenylamino)acrylamide derivatives **6** [40–42] was carried out in boiling dioxane containing sodium methoxide to furnish the corresponding 3-aminothiophene derivatives **7a-c**. The reaction proceeded via the formation of alkylated sulphide intermediate **C**, which was then subjected to intramolecular methylene group addition on the nitrile function to yield the corresponding 3-aminothiophene derivatives **7a-c** (Scheme 3).

### Biological activity

#### Antioxidant assay

In this study, the antioxidant activity of the synthesized thiophene-2-carboxamide derivatives **3a-c**, **5a-c** and **7a-c** were determined using ABTS antioxidant assay [43]. The decrease of absorbance for green ABTS˙^+^ radical cation at 734 nm was measured by using a UV–Visible spectrophotometer. ABTS antioxidant activity was measured by using L-Ascorbic acid as standard and the obtained results of the ABTS antioxidant assay was reported in (Table 1). The results showed that the 3-amino thiophene-2-carboxamide derivative **7a** increased antioxidant activity by 62.0%, which is comparable to the reference antioxidant ascorbic acid (88.44%). Furthermore, 3-amino thiophene-2-carboxamide derivatives **7a-c** promoted the highest antioxidant activity with percent inhibition 62.0–46.9%. While, 3-hydroxy thiophene-2-carboxamide derivatives **3a-c** shows moderate inhibition percentage 54.9–28.4% and 3-methyl thiophene-2-carboxamide derivatives **5a-c** the lowest 22.9–12.0%.

**Table 1:** Antioxidant activity of the newly synthesized thiophene-2-carboxamide derivatives

Compounds	Absorbance of samples	Inhibition (%)
3a	0.374	26.7
3b	0.230	54.9
3c	0.365	28.4
5a	0.428	16.1
5b	0.393	22.9
5c	0.449	12.0
7a	0.194	62.0
7b	0.271	46.9
7c	0.248	51.4
Control of ABTS	0.510	0
Ascorbic acid	0.061	88.0

#### Antibacterial assay

The newly synthesized compounds **3**, **5** and **7** were evaluated for their antibacterial activity against a panel of two pathogenic Gram-positive bacteria (*S. aureus* and *B. subtilis*) and two of pathogenic Gram-negative bacteria (*E. coli* and *P. aeruginosa*), as shown in (Table 2). The antibacterial activity of the tested compounds was estimated in comparison with Ampicillin. Overall, the investigated compounds were more active against Gram-positive bacterial strains. 3-Amino thiophene-2-carboxamide compounds **7a-c** displayed higher antibacterial activity (ranged from 40.0 to 86.9%) than their corresponding 3-hydroxy thiophene-2-carboxamide compounds **3a-c** (from 20.0 to 78.3%) and the lowest inhibition values displayed by 3-methyl thiophene-2-carboxamide compounds **5a-c** (from no activity to 47.8%). The thiophene-2-carboxamide derivatives **3**, **5**, **7b** (substituted with methoxy group at aryl Th^4^) showed the best inhibition activity against Gram-positive bacteria (*S. aureus* and *B. subtilis*) and against Gram-negative bacterium (*P. aeruginosa*) rather than their corresponding derivatives substituted with methyl group or chlorine atom.

**Table 2:** Antimicrobial activity of the newly synthesized thiophene-2-carboxamide derivatives using various bacterial strains

Compound	*E. coli*	*P.aeruginosa*	*S. aureus*	*B. subtilis*
3a	5 (20.0)	11 (47.8)	10 (41.7)	13 (56.5)
3b	13 (52.0)	18 (78.3)	17 (70.8)	18 (78.3)
3c	9 (36.0)	12 (52.2)	13 (54.2)	16 (69.6)
5a	NA (––)	7 (30.4)	6 (25.0)	8 (34.8)
5b	4 (16.0)	10 (43.5)	9 (37.5)	11 (47.8)
5c	NA(––)	3 (13.0)	4 (16.7)	6 (26.1)
7a	12 (48.0)	15 (65.2)	16 (66.7)	17 (73.9)
7b	16 (64.0)	20 (86.9)	20 (83.3)	19 (82.6)
7c	10 (40.0)	17 (73.9)	16 (66.7)	17 (73.9)
Ampicillin	25	23	24	23

*NA* No activity; results of the antibacterial activity expressed as a mean on inhibition zone diameter (mm) and between brackets activity index (%) for different compounds; *E. coli, P. aeruginosa, S. aureus* and* B. subtilis*

Amino thiophene-2-carboxamide compound **7b** containing methoxy group showed excellent activity against *P. aeruginosa* (86.9%), *S. aureus* (83.3%) and *B. subtilis* (82.6%) with inhibition zones 20, 20 and 19 mm, respectively. Hydroxy thiophene-2-carboxamide compound **3b** having methoxy group showed very good effect against *B. subtilis* and *P. aeruginosa* 78.3% (inhibition zone 18 mm) and inhibition activity against *S. aureus* 70.8% (inhibition zone 17 mm).

### Structure activity relationship (SAR) studies

The following structure–activity relationship of newly synthesized thiophene 2-carboxamide derivatives **3a-c**, **5a-c**, and **7a-c** can be derived from antioxidant and antibacterial testing results: (i) Antioxidant and antibacterial activity of the amino thiophene-2-carboxamide derivatives **7a-c** are more potent than hydroxyl or methyl thiophene-2-carboxamide **3a-c**; which may be attributed to the absence of azo moiety and presence of amino group. Increasing the antioxidant power in **7a-c** is caused by presence of electron donating amino group which increases the resonating electron on the thiophene ring, and faceplate the electron trapping for peroxide radical [44, 45]. Moreover, presence of hydroxyl group in derivatives **3a-c** showing higher activity than **5a-c**, perhaps it increase the solubility of this class of compound [46, 47]. (ii) Antioxidant activity of compound **7a** possess potent activity among them; which may be due to absence of substituents on benzene ring Th^4^. While, compounds **3b** and **5b** is more potent than their derivatives which may be attributable to the presence of (-OMe group Th^4^). (iii) Antibacterial activity of amino thiophene-2-carboxamide derivatives **7b** was boosted due to presence of (4-Me on carboxamide Th^2^) against *E. coli, P. aeruginosa, S. aureus* and* B. subtilis.* The structural variations such as (4-OMe on carboxamide Th^4^) and replacement of carboxamide with azo moieties **3b** and **5b** favors the activity in positive manner [48]. The highest antibacterial values for **3b**, **5b**, **7b** may be attributed to the increase in the hydrophilicity power of the antibacterial drug agent caused by methoxy group [49, 50].

### Computational study

#### Molecular modeling

DFT calculations were used to study how the thiophene 2-carboxamide derivatives **3a-c**, **5a-c**, and **7a-c** differ in their shapes and electronic properties. DFT-optimized structures, atomic numbers, and geometrical parameters, including bond length, angle, and dihedral angle, for **3a-c**, **5a-c** and **7a-c** obtained at B3LYP/6-31G (d,p) are shown in (Additional file 1: Fig. S1) and (Additional file 1: Tables S1-S3). The data of thiophene 2-carboxamide derivatives indicated that the H-atom of NHPh(Th^5^) is involved in intramolecular hydrogen bond with *N*^1^ of azo group(Th^4^), in **3a-c** and **5a-c**, while with oxygen of the carboxamide(Th^4^) in **7a-c**. For **3a-c** and **7a-c**, the H-atoms of thiophene-OH and thiophene-NH_2_ formed hydrogen bonds with the O-carboxamide (Th^2^) and not the azo group’s nitrogen atom, (Additional file 1: Fig. S1). The distance between O-carbonyl and H-hydroxyl or H-amino in **3a-c** and **7a-c** derivatives was 1.730–1.732 Å, which was within the H-bond range [51–54].

Using the average values of the dihedral angles *N*azo(1)-Th^4^-Th^5^-S and Th^4^-*N*azo(1)-*N*azo(2)-CPh(Azo), we can see that the thienyl is flat and coplanar with the phenylazo moiety in compounds **3a-c** and **5a-c**. Despite, the NH group was in the same plane as the thienyl ring (NH-Th^5^-S-Th^2^ = 176.56–177.90°), the phenyl ring was angled on the aminothienyl plane between 21 and 24° in compounds **3a-c** and **5a-c** and at an angle of 29° in compounds **7a-c**. Furthermore, the 2-carboxamide group was skewed for compounds **7a-c** = 152° and lied in plane with the thienyl ring as S-Th^2^-Th^3^-OH(th) in range 179–176° for compounds **3a-c** and **7a-c**.

### Frontier molecular orbitals (FMOs)

**Fig. 2:**
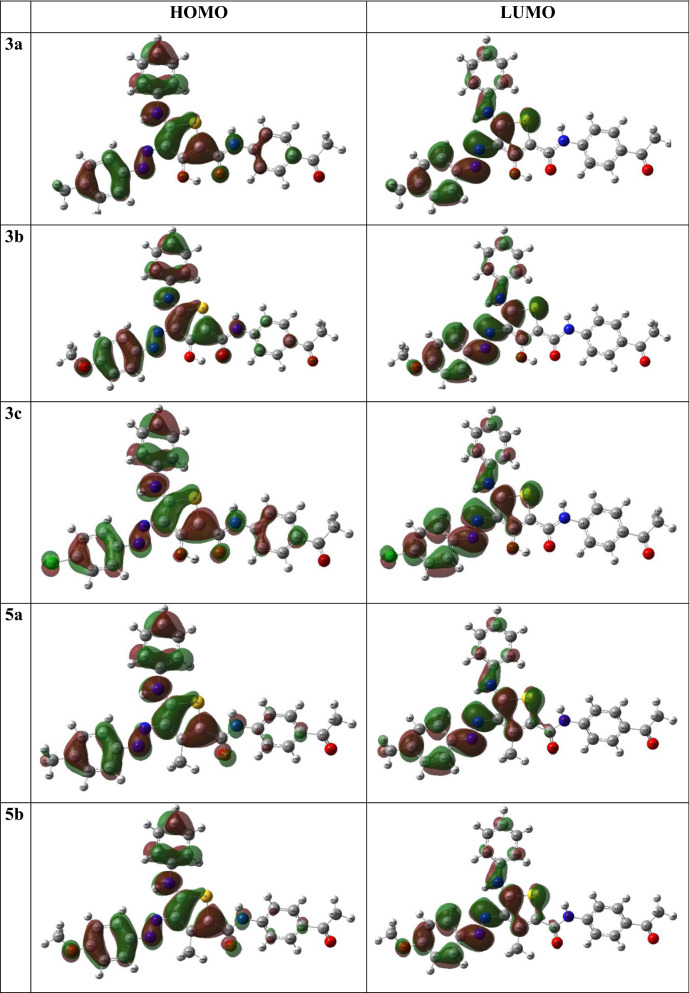

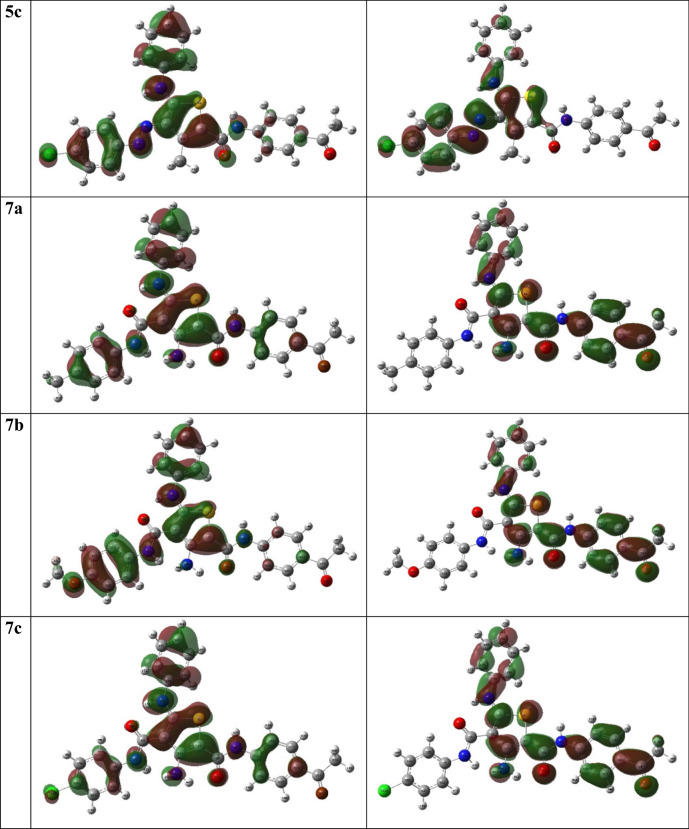
The HOMO and LUMO distribution pattern of **3a-c**, **5a-c** and **7a-c**

Electronic properties and the chemical reactivity of molecules are chiefly determined by Frontier molecular orbitals (FMOs) [55, 56]. The distribution of highest occupied and lowest unoccupied molecular orbitals, HOMO and LUMO, respectively, of the investigated molecules are presented in (Fig. 2). Figure 2 shows that the **3a-c** and **5a-c** derivatives HOMO plots are composed primarily of the π-orbitals of the thienyl, phenyl, and azo groups, as well as S, N, and O, non-bonding lone pairs except OH(Th^3^), which has a slight corporation. LUMO orbitals consist of π^∗^-orbitals of thienyl, phenyl, and azo groups with minimal heteroatom contributions. For **7a-c** derivatives, the HOMO orbitals were distributed over the π-orbitals of the thienyl, phenyl, and amide groups, while their LUMO showed completely different composition, where it is localized only on the 2-carboxamide phenyl thiophene substituent. As shown in (Table 3), the above findings were demonstrated in the E_HOMO_ and E_LUMO_ values. The values of E_HOMO_ were close to each other and ranged from 5.58 to 5.91 eV, while the E_LUMO_ values ranged from 1.99 to 2.73 eV. Furthermore, the p-chlorophenyl derivatives demonstrated the highest E_HOMO_ where **7c** ≈ **3c** > **5c**. The aminothiophene derivatives **7a-c** had the highest E_LUMO_, which might be caused by the presence of the carboxamide group at Th^4^. The values of HOMO–LUMO energy gap (ΔE_H-L_) ranged between 3.11–3.83 eV. It reveals that **7a-c** derivatives have the maximum value. Also, the hydroxyl derivatives **3a-c** were higher than corresponding methyl derivatives **5a-c**. Finally, the amino derivatives, **7a-c** showed higher ΔE_H-L_ than other derivatives.

**Table 3:** The HOMO Energy (E_HOMO_), LUMO Energy (E_LUMO_), HOMO–LUMO Energy Gap (ΔE_H-L_) in eV, electronegativity (χ), global hardness (η), softness (δ) and electrophilicity (ω) at B3LYP/6-31G* Level of Theory

Molecules	E_HOMO_	E_LUMO_	ΔE_H-L_	χ	η	δ	ω
3a	−5.74	−2.46	3.29	4.10	1.64	0.61	5.11
3b	−5.64	−2.39	3.25	4.02	1.63	0.61	4.96
3c	−5.90	−2.68	3.22	4.29	1.61	0.62	5.72
5a	−5.68	−2.50	3.18	4.09	1.59	0.63	5.26
5b	−5.58	−2.43	3.15	4.01	1.57	0.64	5.10
5c	−5.84	−2.73	3.11	4.28	1.56	0.64	5.89
7a	−5.77	−1.99	3.79	3.88	1.89	0.53	3.97
7b	−5.69	−1.99	3.71	3.84	1.85	0.54	3.98
7c	−5.91	−2.08	3.83	3.99	1.91	0.52	4.16

Using the above calculated E_HOMO_ and E_LUMO_ we managed to determine some chemical descriptors such as electronegativity (χ), global hardness (η) and softness (δ) and electrophilicity (ω), respectively (Table 3) [57], where:

$$\chi =-\frac{1}{2}\left({E}_{HOMO}+{E}_{LUMO}\right)\, \eta =-\frac{1}{2}\left({E}_{HOMO}+{E}_{LUMO}\right)$$

$$\delta =\frac{1}{\eta } \omega =\frac{{\chi }^{2}}{2\eta }$$

The results showed that **3c** derivative possessed the highest Lewis acid character, while **7b** derivative exhibited the lowest. Although **5c** was the softest derivative, **7c** was the hardest. Based on the electrophilicity (ω) results, the HOMO–LUMO electron flow in the **5c** derivative resulted in a greater decrease in energy compared to the **3c** and **7c** derivatives.

### Mulliken’s charges, Fukui’s and relative indices

Describing the electronegativity and charge transfer processes was offered as an output of quantum chemical calculations. This calculation process is described as the Mulliken’s atomic charges [58] (Additional file 1: Table S4). As expected, the results showed that all heteroatoms had negative charges owing to their high electronegativity, with the exception of *N*_azo(2),_ NH_CO_ and NH_Th5_, each of which has positive charges, showing that its lone pair is involved in the resonance of phenyl ring. Much more, in derivatives **3a-c**, the carbon atom of thienyl (Th^3^) has close negative charges, but in derivatives **5a-c**, they have close positive charges. This could be due to the OH group electron donating effect. On parallel, the thienyl carbon atoms Th^3^ in derivatives **7a-c** have close negative charges and lower than **3a-c** and the reason behind this decrease is replacing azo group with amide group in position 4. Moreover, determination of the Fukui indices (${f}_{k}^{+}$ and ${f}_{k}^{-}$) was also used to investigate the reactivity of atoms toward nucleophilic and electrophilic attacks were explored using the following equations [59–61], where *q k* (*N*), *q k* (*N* + 1) and *q k* (*N* − 1) are the systems atomic charges with *N*, *N* + 1 and *N*-1 electrons, respectively [62].

$${f}_{k}^{+}={q}_{k}\left(N+1\right)-{q}_{k}(N)$$

$${f}_{k}^{-}={q}_{k}\left(N\right)-{q}_{k}(N-1)$$

It is clear for all derivatives ${f}_{k}^{+}$ values are higher than ${f}_{k}^{-}$ for all heteroatoms. Except NH_amideTh2_, NH_Th5_, OH (**3a-c**), *N*_azo2_ (**7a-c**) and S (**7a-c**),${f}_{k}^{+}$ have lower values than ${f}_{k}^{-}$ proving lone pair participation in ring resonating structure. In some cases ${f}_{k}^{+}$ values equals ${f}_{k}^{-}$ values; *N*_azo1_(**7a-c**), O_acetyl_(**3a-c**, **5a-c**). The data showed Th^3^ has higher ${f}_{k}^{+}$ index in all derivatives, reached about 2 folds in comparison to ${f}_{k}^{-}$ (Additional file 1: Table S4).

### Molecular docking

**Fig. 3:**
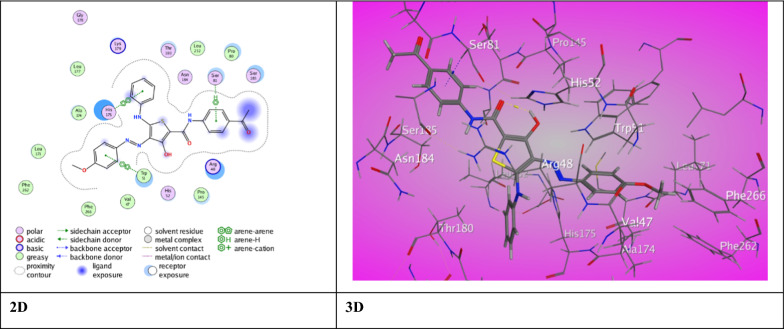
The binding interaction of 3-hydroxythiophene **3b** with (PDB ID: 2AS1)

**Fig. 4:**
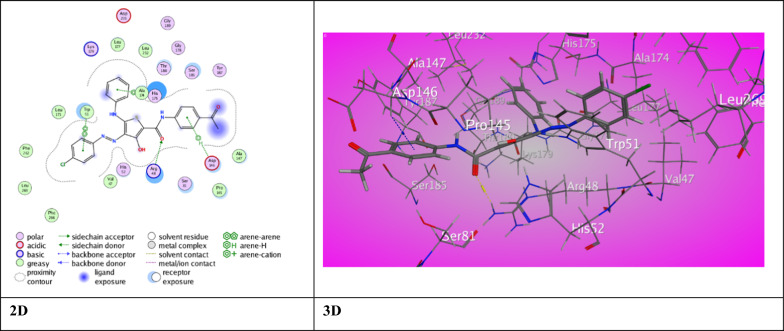
The binding interaction of 3-hydroxythiophene **3c** with (PDB ID: 2AS1)

To get a promising approach of synthesized ligands interactions with antioxidant and bacterial protein receptors, docking studies were performed. A molecular docking investigation was conducted on the newly synthesized thiophene-2-carboxamide derivatives in order to investigate their interaction with the crystallographic coordinates available in the RCSB Protein Data Bank. Downloaded from the Protein Data Bank [63] and delivered via the operating MOE “v10.2015.10 software,” (PDB ID 2AS1) was chosen as the antioxidant target for the derivatives so that its activity could be tested and specified (Table 4). It is noteworthy that all of the compounds showed excellent inhibition activities against target proteins and higher than previously prepared compounds [17] perhaps because of the presence of carboxamide and thienyl groups in all the compounds which can develop a variation of associations in the active site of proteins. The 3-hydroxy thiophene carboxamide derivative **3a** displayed one intermolecular H-bond between *N*-aniline (Th^5^) with Pro 145 (2.99 ˚A) offering a score for binding energy, S = −8.1675 kcal/mol. Derivative **3b** showed π- H interaction between aniline (Th^5^) with Ser 8 (3.84 ˚A) and π- π stick interactions between aryl azo (Th^4^) and Ph (Th^2^) with Trp 51 (3.83 ˚A) and His 175 (3.84 ˚A), respectively. An eminent binding energy score, S = −9.3283 kcal/mol was offered for derivative **3b** (Fig. 3). But, derivative **3c** displayed two distinct binding modes promoting a distinguished score of binding energy, S = −9.1141 kcal/mol (Fig. 4). The first is an H-bond between O-carboxamide (Th^2^) with Arg 84 (3.02 ˚A). The second binding type was π-H and π- π stick between Ph (Th^2^), aryl azo (Th^4^) and aniline (Th^5^) with Asp 146, Trp 51 and His 175 (4.69 ˚A, 3.85 ˚A, 3.88 ˚A), respectively.

**Table 4:** The molecular docking data of the synthesized thiophene 2-carboxamide derivatives with 2AS1 (Anti-oxidant)

Ligand	S (energy score) (Kcal/mol)	Rmsd (refine unit)	Interaction with ligand	Types of interactions	Distance (A)	rseq	E_conf
3a	−8.1675	1.2616	*N-*aniline(Th^5^) with Pro 145	H-donor	2.99	1	11.2872
3b	−9.3283	1.4389	Aniline (Th^5^) with Ser 81 Aryl azo (Th^4^) with Trp 51 Ph (Th^2^) with His 175	π-H interaction π-π interaction π-π interaction	3.84 3.83 3.84	1	16.5201
3c	−9.1141	1.6664	O-carboxamide (Th^2^) Arg 48 Ph (Th^2^) with Asp 146 Aryl azo (Th^4^) with Trp 51 Aniline (Th^5^) with His 175	H-acceptor π-H interaction π-π interaction π-π interaction	3.02 4.69 3.85 3.88	1	25.7050
5a	−8.7347	1.5232	Aniline (Th^5^) Arg 48 Aryl azo (Th^4^) Trp 51	π-H interaction π-π interaction	4.14 3.70	1	23.4919
5b	−6.4382	1.5862	O-carboxamide (Th^2^) Arg 72 O-acetyl group with Glu 135 Aniline (Th^5^) His 96	H-acceptor H-acceptor π-π interaction	3.04 3.12 3.65	1	21.0089
5c	−7.4535	1.5972	*N*^1^-azo group with Ser 81 Thiophene ring with Asp 146	H-acceptor π-H interaction	3.24 4.37	1	24.2779
7a	−8.2395	1.5308	O-carboxamide (Th^2^) with Trp 51 O-carboxamide (Th^2^) with His 52	H-acceptor H-acceptor	3.59 3.23	1	−50.4239
7b	−6.0187	1.3779	*N*-carboxamide (Th^4^) with Asp 148 *N*-amino group (Th^3^) with Asp 146 O-acetyl group with Arg 48 Thiophene ring with Ala 147 Ph (Th^4^) with Lys 149	H-donor H-donor H-acceptor π-H interaction π-cation interaction	3.49 2.90 3.10 4.32 3.44	1	−53.7450
7c	−7.3131	1.7642	S-thiophene with Ser 81 Cl-benzamide (Th^4^) with Leu 177	H-donor H-donor	3.91 3.27	1	−52.8699
Ascorbic acid	−4.7248	1.0693	O (Lac^3^) with His 181 O (Eth^1^) Leu 177	H-donor H-donor	3.16 2.88	1	87.5356

Meanwhile, 3-methyl thiophene carboxamide derivative **5a** exhibited notable binding score (S = −8.7347 kcal/mol). The π-H and π- π stick forces between aniline (Th^5^) and aryl azo(Th^4^) with Arg 48 (4.14 ˚A) and Trp 51 (3.70 ˚A) was the reason for binding. A weak score of binding (S = −6.4382 kcal/mol) showed by derivative **5b**. This is due to two H-bonds exhibited between O-carboxamide (Th^2^) and O-acetyl with Arg 72 (3.04 ˚A) Glu 135 (3.12 ˚A) as well π- π stick forces between aniline (Th^5^) and His 96 (3.65˚A). Moreover, derivative **5c** demonstrated a respectable binding score, (S = −7.4535 kcal/mol) due to H-bond between *N*^1^-azo group with Ser 81(3.65˚A) and π-H interaction between thiophene ring with Asp 146 (4.37 ˚A). However, 3-amino thiophene carboxamide derivative **7a** revealed two intermolecular H-bond between Trp 51 and His 52 with the O-carboxamide (Th^2^) (3.59 ˚A), (3.23 ˚A) respectively. Derivative **7a** exhibited a highly regarded binding score with the amino acids of 2AS (S = −8.2395 kcal/mol). Derivative **7b** exhibited three intermolecular hydrogen bonds between *N*-carboxamide (Th^4^) with Asp 148 (3.49˚A), and *N*-amino group (Th^3^) with Asp 146 (2.90˚A) besides, O-acetyl group with Arg 48 (3.10 ˚A). It also showed π-H interaction between thiophene ring with Ala 147 (4.32˚A), and π-cation interaction between Ph (Th^4^) with Lys 149 (3.44˚A) recording a weak score (S = −6.0187 kcal/mol). Moreover, derivative **7c** presented two intermolecular H-bond between Ser 81 and Leu 177 with the S-atom and Cl atom (3.91 ˚A), (3.27 ˚A) through very good binding score (S = −7.4535 kcal/mol). The ascorbic acid was subjected to amino acid 2AS1 and shows low score (S = −4.7248 kcal/mol) resulted from two H-bonds between O(Lac^3^) and O(Et^1^) with His 181 and Leu 177 (3.16 ˚A), (2.88 ˚A) respectively.

For antibacterial target, IMP-1 metallo beta-lactamase (PDB ID 1DD6), from *P. aeruginosa* and sortase A (PDB ID 2MLM) from *S. aureus* [64] and rhomboid protease (PDB ID 3ZMI) from *E. coli* [64] and Bacillus subtilis nitric oxide synthase I218V (PDB ID 4D3V) from *B. subtilis* [65] was selected and downloaded from the Protein Data Bank. For anti-bacterial protein IMP-1 metallo beta-lactamase (PDB ID 1DD6), the ligands inhibited the target by developing various associations with amino acid residues of active site of *P. aeruginosa* (Table 5). The 3-hydroxy thiophene carboxamide derivative **3a** recorded outstanding score of binding (S = −7.5022 kcal/mol) as a result of π-H interaction between anilide (Th^2^) with Trp 28 (4.17 ˚A). Moreover, derivative **3b** demonstrated intermolecular H-bonds between *N-*aniline with Leu 39 (2.89 ˚A) and one π- H interaction between aniline (Th^5^) with Glu 150 (4.30 ˚A) through binding score (S = −6.6965 kcal/mol). Additionally, derivative **3c** showed a weak binding score (S = −6.1669 kcal/mol). It has two H-bond interaction between O-carboxamide (Th^2^) with Tyr 45 (3.08 ˚A) and Lys 71 (2.84˚A) plus π- H interaction between aniline (Th^4^) with Tyr 97 (3.83 ˚A) and aryl azo (Th^5^) with Tyr 97(3.85 ˚A).

**Table 5:** The molecular docking data of the synthesized thiophene 2-carboxamide derivatives with 1DD6 (*P. aeruginosa*)

Code	S (energy score) (Kcal/mol)	Rmsd (refine unit)	Interaction with ligand	Types of interactions	Distance (A)	rseq	E_conf
3a	−7.5553	1.3950	Anilide (Th^2^) with Trp 28	H-π interaction	4.17	1	19.4438
3b	−6.6965	1.4268	*N*-aniline with Leu 39 Aniline (Th^5^) with Glu 150	H-donor π-H interaction	2.89 4.30	1	14.8238
3c	−6.1669	1.1349	O-carboxamide (Th^2^) with Tyr 45 O-carboxamide (Th^2^) with Lys 71 aniline (Th^4^) with Tyr 97 aryl azo (Th^5^) with Tyr 97	H-acceptor H-acceptor π-H interaction π-H interaction	3.08 2.84 3.83 3.85	1	18.9813
5a	−7.6609	1.4058	O-acetyl group with Ser 80 H-acetyl group with His 79	H-acceptor H-π interaction	2.95 3.90	1	26.8327
5b	−7.4306	1.4020	O-carboxamide(Th^2^) with Asn 167 Aniline (Th^5^) with Val 25 Aryl azo (Th^4^) with Ser 80	H-acceptor π-H interaction π-H interaction	3.32 4.11 4.24	1	25.9591
5c	−6.0538	1.1783	O-acetyl with Lys 215 Aniline (Th^5^) with Tyr 163	H-acceptor π-H interaction	3.02 4.51	1	27.1141
7a	−7.4729	1.0919	*N*-aniline (Th^5^) with Leu 39 O-carboxamide (Th^4^) with Asn 41 Anilide (Th^4^) with Ala 42	H-donor H-acceptor π-H interaction	3.07 3.05 4.65	1	−50.3092
7b	−6.2489	0.9195	O-carboxamide (Th^2^) with Lys 127 O-carboxamide (Th^2^) with Lys 127 O-carboxamide (Th^4^) with Tyr 97 Anilide (Th^4^) with Tyr 97 Aniline (Th^5^) with Trp 124	H-acceptor H-acceptor H-acceptor π-H interaction π-H interaction	3.42 2.88 2.92 4.37 4.43	1	−54.4244
7c	−6.5478	1.5580	*N*-amino (Th^3^) with Asp 170 Thiophene ring with Ser 80 Thiophene ring with His 79	H-donor π-H interaction π-π interaction	3.29 4.95 3.96	1	−47.9835
Ampicillin	−6.8228	1.2780	O-cyclic amide with His 197 O-carboxyl group with Lys 161	H-acceptor H-acceptor	3.06 2.88	1	76.1998

**Fig. 5:**
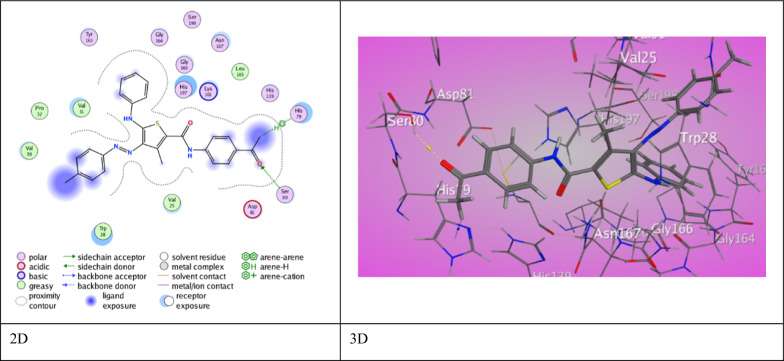
The binding interaction of 3-methylthiophene 5a with (PDB ID: 1DD6)

Meanwhile, a good score of binding (S = −7.6609 kcal/mol) offered by 3-methyl thiophene carboxamide derivative **5a** through two interaction modes (Fig. 5). An H-bond was presented between O-acetyl group with Ser 80 (2.95 ˚A), and H-π force was exhibited between H-acetyl group with His 79 (3.90 ˚A). Derivative **5b** showed decent score (S = −7.4306 kcal/mol) outcomes from one H-bond interaction between O-carboxamide with Asn 167 (3.32 ˚A), and two π-H interaction between aniline with Val 25 (4.11 ˚A) and aryl azo with Ser 80 (4.24 ˚A). Furthermore, derivative **5c** shows weak score (S = −6.0538 kcal/mol) as a consequence of one H-bond interaction between O-acetyl group with Lys 215 (3.02 ˚A), and π-H interaction between aniline with Tyr 163 (4.51 ˚A).

However, 3-amino thiophene carboxamide derivative **7a** revealed two intermolecular H-bond between *N*-aniline (Th^5^) and O-carboxamide (Th^4^) with Leu 39 (3.07 ˚A) and Asn 41 (3.05 ˚A) besides, π-H interaction between anilide with Ala 42 (4.65 ˚A). Derivative **7a** was displayed a highly regarded binding score (S = −7.4729 kcal/mol). Furthermore, derivative **7b** exhibited three intermolecular hydrogen bonds between O-carboxamide (Th^2^) with Lys 127 (3.42˚A), (2.88˚A), respectively. In addition to, O-carboxamide (Th^4^) with Tyr 97 (2.92 ˚A). It also showed two π-H interaction between anilide(Th^4^) with Tyr 97 (4.37˚A), and π-H interaction between aniline (Th^5^) with Try 124 (4.43˚A) offering a weak score (S = −6.2489 kcal/mol). Moreover, derivative **7c** presented one H-bond between Asp 170 with *N*-amino (Th^3^) (3.29 ˚A). It also displayed π-H and π-π interaction between thiophene ring with Ser 80 (4.95 ˚A) and His 79 (3.96 ˚A) showing a good score (S = −6.5478 kcal/mol). Ampicillin was also docked with 1DD6 protein and showed good binding score (S = −6.8228 kcal/mol) resulted from intermolecular H-bond between O-cyclic amide and O-carboxyl group with His 179 (3.06 ˚A) and Lys 161 (2.88 ˚A).

For anti-bacterial protein sortase A (PDB ID 2MLM), the ligands inhibited the target by developing various associations with amino acid residues of active site of *Staphyllococcus aureus* (Table 6)*.* The 3-hydroxy thiophene carboxamide derivative **3a** exhibited two intermolecular H-bonds between S-thiophene ring with Asn 56 (3.69 ˚A) and O-carboxamide (Th^2^) with Lys 117 (3.03 ˚A) through a binding score (S = −6.9492 kcal/mol). Also, derivative **3b** demonstrated two intermolecular H-bonds between S-thiophene ring with Asn 56 (3.08 ˚A) and *N*-aniline (Th^5^) with Thr 122 (3.17 ˚A) through binding score (S = −6.6975 kcal/mol). Moreover, derivative **3c** demonstrated two types of interactions. Two intermolecular H-bonds between S-thiophene ring with Gln 114 (3.67 ˚A) and O-carboxamide (Th^2^) with Lys 117 (3.06 ˚A) and one π- H interaction between thiophene ring with Gln 120 (3.95 ˚A).

**Table 6:** The molecular docking data of the synthesized thiophene 2-carboxamide derivatives with 2MLM (*S. aureus*)

Code	S (energy score) (Kcal/mol)	Rmsd (refine unit)	Interaction with ligand	Types of interactions	Distance (A)	rseq	E_conf
3a	−6.9492	1.4683	S-thiophene ring with Asn 56 O-carboxamide (Th^2^) with Lys 117	H-donor H-acceptor	3.69 3.03	1	26.3509
3b	−6.6975	1.1433	S-thiophene ring with Asn 56 *N*-aniline (Th^5^) with Thr 122	H-donor H-donor	3.08 3.17	1	17.1511
3c	−6.5149	1.0726	S-thiophene ring with Gln 114 O-carboxamide (Th^2^) with Lys 117 Thiophene ring with Gln 120	H-donor H-acceptor π-H interaction	3.67 3.06 3.95	1	17.7282
5a	−6.2959	0.9967	S-thiophene ring with Asn 56 *N*-aniline (Th^5^) with Glu 47 *N*-aniline (Th^5^) with Glu 47	H-donor H-donor H-donor	3.63 3.02 3.12	1	24.9926
5b	−6.3089	0.8325	S-thiophene ring with Val 108 *N*-aniline (Th^5^) with Val 108 O-carboxamide (Th^2^) with Arg 139 O-carboxamide (Th^5^) with Arg 139	H-donor H-donor H-acceptor H-acceptor	4.38 3.17 3.20 3.14	1	23.4584
5c	−7.0550	1.0874	O-acetyl with Gln 120 Aryl azo (Th^4^) with Ala 46	H-acceptor π-H interaction	3.14 4.06	1	26.0452
7a	−7.0842	1.1622	S-thiophene ring with Glu 113 O-acetyl group with Gln 120 Aniline (Th^5^) with Arg 139	H-donor H-acceptor π-cation interaction	3.68 3.00 3.93	1	−57.4937
7b	−6.8518	0.9057	S-thiophene ring with Asn 56 O-carboxamide (Th^2^) with Lys 117	H- donor H-acceptor	4.09 2.97	1	−54.0559
7c	−6.7376	1.3201	*N*-amide (Th^2^) with Gln 120 Aniline (Th^5^) Ser 99 Anilide (Th^4^) Thr 122	H-donor π-H interaction π-H interaction	3.16 3.68 4.12	1	−50.5308
Ampicillin	−5.0480	1.3765	C-benzyl with Glu 47 *N*-amino group with Asn 56 S-thiazole with Glu 47 O-cyclic ketone with Lys 117 O-β-lactam with Lys 117 O-carboxylic ketone with Gln 55	H-donor H-donor H-donor H-acceptor H-acceptor H-acceptor	3.35 3.23 3.63 3.00 3.07 2.93	1	72.4682

Meanwhile, 3-methyl thiophene carboxamide derivative **5a** exhibited a binding score (S = −6.2959 kcal/mol). The binding interactions are three H-bonds between *N*-aniline (Th^5^) with Glu 47 (3.02 ˚A) (3.12 ˚A) and S-thiophene ring with Asn 56 (3.63 ˚A). A weak binding score was assigned to derivative **5b** (S = −6.3089 kcal/mol). Derivative **5b** displayed four H-bond between S-thiophene ring and *N*-aniline (Th^5^) with Val 108 in addition to O-carboxamide (Th^2^) and (Th^5^) with Arg 139 (4.38 ˚A), (3.17 ˚A), (3.20 ˚A), (3.14 ˚A), respectively. Derivative **5c** demonstrated a proper binding score, (S = −7.0550 kcal/mol). The interactions in derivative **5c** involves H-bond between O-acetyl group with Gln 120 (3.14˚A) and π-H interaction between arylazo (Th^4^) with Ala 46 (4.06 ˚A).

**Fig. 6:**
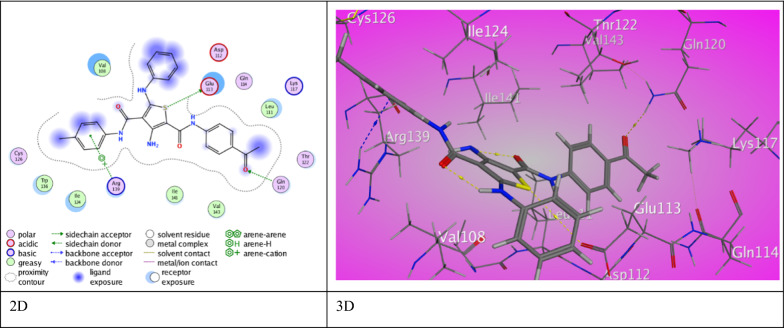
The binding interaction of 3-aminothiophene **7a** with (PDB ID: 2MLM)

However, 3-amino thiophene carboxamide derivative **7a** displayed a highly regarded score of binding with the amino acids of 2MLM (S = −7.0842 kcal/mol) rose from two H-bond between O-acetyl group with Gln 120 (3.00 ˚A) and S-thiophene ring with Glu 113 (3.68 ˚A). Also, a π-cation interaction between aniline (Th^5^) with Arg 139 (3.93 ˚A) was noticed (Fig. 6). Furthermore, derivative **7b** exhibited two H-bonds between S-thiophene ring with Asn 56 (4.09˚A) along with O-carboxamide (Th^2^) with Lys 117 (2.97 ˚A) through abundant binding score (S = −6.8518 kcal/mol). Additionally, derivative **7c** presented H-bond between Gln 120 with *N*-amide (Th^2^) (3.16 ˚A). It also exhibited two π-H interactions between aniline (Th^5^) and (Th^4^) with Ser 99 and Thr 122 (3.68 ˚A), (4.12 ˚A) respectively through good binding score (S = −6.7376 kcal/mol). Ampicillin showed with 2MLM protein good binding score (S = −5.0480 kcal/mol) resulted from six H-bonds between C-benzyl and S-thiazole with Glu 47 (3.35 ˚A), (3.63 ˚A), respectively. As well, *N*-amino group with Asn 56 (3.23 ˚A) and O-carboxylic ketone with Gln 55 (2.93 ˚A) was also appeared. Lys 117 was engaged in two H-bonds with O-cyclic ketone and O-β-lactam (3.00 ˚A and 3.07 ˚A).

For anti-bacterial protein rhomboid protease (PDB ID 3ZMI), the ligands inhibited the target by developing various associations with amino acid residues of active site of *E. coli* (Table 7).

**Table 7:** The molecular docking data of the synthesized thiophene 2-carboxamide derivatives with 3ZMI (*E. Coli*)

Code	S (energy score) (Kcal/mol)	Rmsd (refine unit)	Interaction with ligand	Types of interactions	Distance (A)	rseq	E_conf
3a	−7.5672	1.4487	O-acetyl group with Met 208 O-carboxamide (Th^2^) with Ser 201 Thiophene ring with His 150	H-donor H-acceptor π-H interaction	3.40 2.96 4.12	1	12.7966
3b	−6.8908	1.3841	O-carboxamide (Th^2^) with Arg 92	H-acceptor	2.92	1	25.5604
3c	−6.8945	1.5074	*N*-aniline (Th^5^) with Ser 201 *N*^1^-azo group with Ser 201 Thiophene ring with His 150	H-donor H-acceptor π- π interaction	3.17 3.35 3.58	1	17.9663
5a	−6.1292	0.8206	S-thiophene ring with Leu 152	H-donor	4.14	1	21.0952
5b	−7.3344	1.5308	*N*^2^-azo group with Ser 201 Thiophene ring with His 150 Ph (Th^2^)with Phe 146	H-acceptor π- π interaction π- π interaction	3.00 3.88 3.58	1	24.7280
5c	−7.2949	1.5666	Thiophene ring with His 150 Ph(Th^2^) with Phe 146	π- π interaction π- π interaction	3.78 3.95	1	23.9127
7a	−7.1277	1.2547	O-carboxamide (Th^4^) with Ser 201 O-carboxamide (Th^4^) with His 254 Thiophene ring with Gly 198 Ph (Th^2^)with Phe 146	H-acceptor H-acceptor π-H interaction π- π interaction	3.27 3.00 4.78 3.68	1	−49.7131
7b	−7.0293	1.3327	O-carboxamide with Ala 250	H-acceptor	2.96	1	−51.8681
7c	−7.8889	1.0667	Ph (Th^4^)with His 150	π- π interaction	3.95	1	−51.8923
Ampicillin	−5.5469	1.2420	O-carboxylic ketone with Arg 168 O-β-lactam with Arg 168 Benzene ring with Arg 92	H-acceptor H-acceptor π-cation interaction	3.51 3.01 3.38	1	71.6361

The 3-hydroxy thiophene carboxamide derivative **3a** displayed two H-bonds between O-carboxamide (Th^2^) with Ser 201 (2.96 ˚A), O-acetyl group with Met 208 (3.40 ˚A) and π- H interaction between thiophene ring with His 150 (4.12 ˚A). These interactions have a strong binding score (S = −7.5672 kcal/mol). Derivative **3b** demonstrated binding score (S = −6.8908 kcal/mol) presented in a H-bond between O-carboxamide (Th^2^) with Arg 92 (2.92˚A). Moreover, derivative **3c** shows two mode of interactions via a binding score (S = −6.8945 kcal/mol). Two H-bonds between N-aniline (Th^5^) (3.17 ˚A) and N^1^-azo group with Ser 201 (3.35˚A) and π- π interaction between thiophene ring with His 150 (3.58 ˚A).

Meanwhile, 3-methyl thiophene carboxamide derivative **5a** exhibited binding score (S = −6.1292 kcal/mol) resulted from H-bonds between S-thiophene ring with Leu 152 (4.14 ˚A). But, derivative **5b** showed a rising binding score (S = −7.3344 kcal/mol). Binding modes are, one H-bond displayed between *N*_2_-azo group with Ser 201 (3.00 ˚A) as well as π- π stick forces between Ph(Th^2^) with Phe 146 (3.58 ˚A) and thiophene ring with His 150 (3.88 ˚A). Derivative **5c** have a relatively high binding score, (S = −7.2949 kcal/mol). It also forms a π- π stick forces between thiophene ring with His 150 (3.78 ˚A) and Ph (Th^2^) with Phe 146 (3.95 ˚A).

**Fig. 7:**
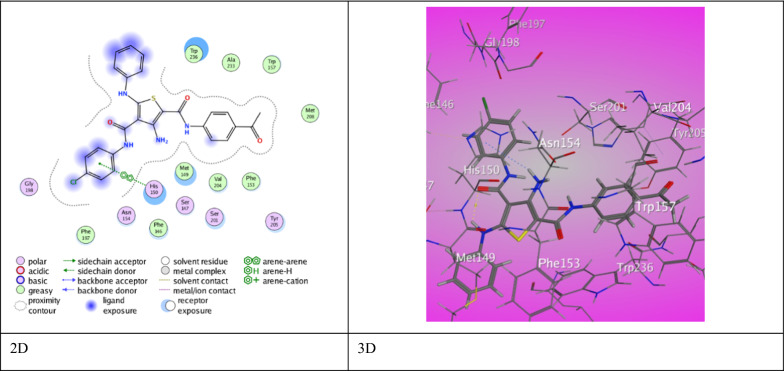
The binding interaction of 3-aminothiophene **7c** with (PDB ID: 3ZMI)

However, 3-amino thiophene carboxamide derivative **7a** established two interaction modes with a binding score (S = −7.1277 kcal/mol). Derivative **7a** shows two H-bond between O-carboxamide (Th^4^) with Ser 201 and His 254 (3.27˚A), (3.00˚A), respectively. A π-H type of interaction between thiophene ring with Gly 198 (4.78 ˚A) and π- π stick forces between Ph (Th^2^) Phe 146 (3.68 ˚A). Derivative **7b** demonstrated one H-bond between O-carboxamide (Th^2^) with Ala 250 (2.96 ˚A) through a binding score (S = −7.0293 kcal/mol). Derivative **7c** presented one π-π stick interaction between His 150 and Ph (Th^4^) (3.95 ˚A) via a unique binding score (S = −7.8889 kcal/mol) (Fig. 7). Ampicillin established a binding score (S = −5.5469 kcal/mol) with 3ZMI protein resulted from two H-bonds between Arg 168 with O-carboxylic ketone and O-β-lactam (3.51 ˚A and 3.01 ˚A). Besides π-cation interaction between benzene ring with Arg 92 (3.38 ˚A).

For anti-bacterial protein Bacillus subtilis nitric oxide synthase I218V (PDB ID 4D3V), the ligands inhibited the target by developing various associations with amino acid residues of active site of *B. subtilis* (Table 8)*.*

**Table 8:** The molecular docking data of the synthesized thiophene 2-carboxamide derivatives with 4D3V (*B. subtilis*)

Code	S (energy score) (Kcal/mol)	Rmsd (refine unit)	Interaction with ligand	Types of interactions	Distance (A)	rseq	E_conf
3a	−8.2175	1.5172	*N*-aniline (Th^5^) with Trp 329 Ph (Th^2^) with Gly 68	H-donor π-H interaction	3.17 3.98	1	24.3131
3b	−7.9612	0.9335	*N*-carboxamide (Th^2^) with Met 221 O-acetyl group with Lys 360 Aniline (Th^5^) with Arg 65 Ph (Th^2^) with His 128	H-donor H-acceptor π-H interaction π- π interaction	4.23 2.92 4.11 3.90	1	18.0041
3c	−6.5618	0.9875	*N* atom of azo group (*N*^2^) with Lys 211 Benzene ring (Th^2^) with Glu 194	H-acceptor π-H interaction	3.14 3.78	1	19.0186
5a	−7.2325	1.5263	Benzene ring (Th^2^) with Arg 65	π-H interaction	4.19	1	20.4180
5b	−6.0805	1.1811	Thiophene ring with Pro 196	π-H interaction	3.78	1	20.9885
5c	−7.3792	1.3171	S-thiophene ring with Tyr 357 O-carboxamide (Th^2^) with Arg 65 O-acetyl group with Asn 64 Ph (Th^2^) with Arg 65	H-donor H-acceptor H-acceptor π-H interaction	3.37 2.89 3.08 4.83	1	33.2914
7a	−7.2786	1.3658	H-acetyl group with Trp 238 Ph (Th^5^) with Trp 329	H-π interaction π-H interaction	3.87 4.45	1	−51.9118
7b	−7.7337	1.2784	*N*-NH_2_ (Th^3^) with Cys 66 *N*-aniline (Th^5^) with Met 221 Ph (Th^4^) with Arg 65 Thiophene ring with Phe 235	H- donor H- donor π-H interaction π-H interaction	3.68 3.70 3.77 4.65	1	−53.0105
7c	−6.4323	1.2726	*N*-carboxamide (Th^4^) with Asp 256 *N*-carboxamide (Th^2^) with Asp 256 O-acetyl group with Lys 257 O-acetyl group with Lys 260 Ph (Th^4^) with Lys 259	H-donor H-donor H-acceptor H-acceptor π-H interaction	3.29 2.96 2.93 2.98 4.34	1	−45.5377
Ampicillin	−6.5350	1.3533	O-carboxyl group with Glu 243	H-donor	2.86	1	78.6567

**Fig. 8:**
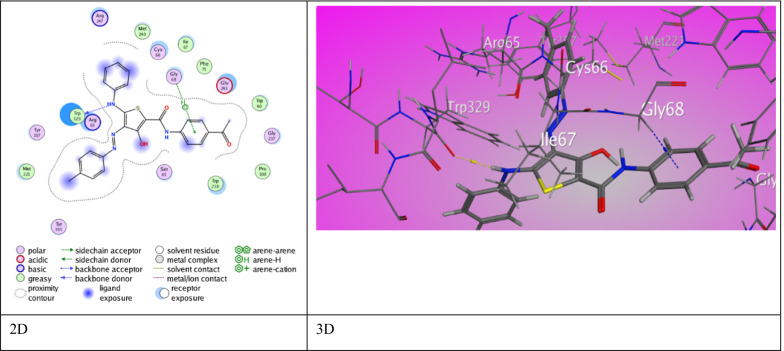
The binding interaction of 3-hydroxythiophene **3a** with (PDB ID: 4d3v)

The 3-hydroxy thiophene carboxamide derivative **3a** exhibited two types of interactions offering an eminent binding score (S = −8.2175 kcal/mol). A H-bond between *N*-aniline (Th^5^) with Trp 329 (3.17 ˚A) and π- H interaction between Ph (Th^2^) with Gly 68 (3.98 ˚A) (Fig. 8). Likewise, derivative **3b** proved pre-eminent binding score (S = −7.9612 kcal/mol) through two modes of interactions. Two H-bonds between *N*-carboxamide (Th^2^) with Met 221 (4.23˚A) and O-acetyl group with Lys 360 (2.92˚A). Besides π- H interaction and π- π stick forces between aniline (Th^5^) and Ph (Th^2^) with Arg 65 (4.11 ˚A) and His 128 (3.90 ˚A). Derivative **3c** showed two modes of interactions and a score of binding (S = −6.5618 kcal/mol). A H-bond between *N*_2_-azo group with Lys 211 (3.14 ˚A) and π- H interaction between Ph (Th^2^) with Glu 194 (3.78 ˚A). Furthermore, 3-methyl thiophene carboxamide derivative **5a** revealed binding score (S = −7.2325 kcal/mol) via π-H interaction between Ph (Th^2^) with Arg 65 (4.19 ˚A). Derivative **5b** have a fallen binding score (S = −6.0805 kcal/mol) through π-H interaction between thiophene ring with Pro 196 (3.78 ˚A). Derivative **5c** demonstrated a relatively high binding score, (S = −7.3792 kcal/mol) through three H-bonds between S-thiophene ring with Tyr 357 (3.37 ˚A) and O-carboxamide (Th^2^) with Arg 65 (2.89 ˚A) and O-acetyl group with Asn 64 (3.08 ˚A). Along with π- H interaction between Ph (Th^2^) with Arg 65 (4.83 ˚A).

However, 3-amino thiophene carboxamide derivative **7a** presented two interactions through a binding score (S = −7.2786 kcal/mol). It shows H-π interaction between H-acetyl group with Trp 238 (3.87 ˚A) and π-H interaction between Ph (Th^5^) with Trp 329 (4.45 ˚A). Derivative **7b** established excellent binding score (S = −7.7337 kcal/mol) caused by two H-bonds between *N*-amino (Th^3^) and *N*-aniline (Th^5^) with Cys 66 (3.68 ˚A) and Met 221 (3.70 ˚A) besides two π-H interaction between Ph (Th^4^) and thiophene ring with Arg 65 (3.77 ˚A) and Phe 235 (4.65 ˚A). Furthermore, derivative **7c** presented π-H interaction between Lys 259 with Ph (Th^4^) (4.34 ˚A) in addition to four H-bonds through binding score (S = −6.4323 kcal/mol). Asp 256 established H-bonds with *N*-carboxamide (Th^4^) (3.29 ˚A) and (Th^2^) (2.96 ˚A). Lys 257 and Lys 260 showed H- bonds with O-acetyl group (2.93 ˚A) (2.98 ˚A). Ampicillin showed with 4D3V protein binding score (S = −6.5350 kcal/mol) resulted from H-bond between Glu 243 with O-carboxyl group (2.86 ˚A).

## Conclusion

A series of 3-substituted thiophene 2-carboxamide with functional groups (-OH, -Me and -NH_2_) were synthesized. In addition to intramolecular H-bonds, the DFT modelling data suggested a comparable Mulliken's charge and HOMO–LUMO contribution. The newly synthesized 3-substituted thiophene 2-carboxamide **3a-c**, **5a-c**, **7a-c** antioxidant activity was examined through ABTS method, while the antibacterial activity was assessed against 4 types of bacteria. Derivatives **7a-c** inhibited better than derivatives **3a-c** and **4a-c**. SAR-study of the obtained compounds shows the enhancement of OMe substituent on the activity and absence of substituent was also a positive impact. In addition, the synthesized compounds were subjected to a molecular docking analysis using (PDB ID 2AS1) (PDB ID 1DD6) (PDB ID 2MLM) (PDB ID 3ZMI) (PDB ID 4D3V). Thiophene derivatives **3b**, **5a**, **7a**, **7c** and **3a** had better score of binding (S = −9.3283, −7.6609, −7.0842, −7.8889 and −8.2175 kcal/mol) toward the selected proteins.

## Experimental

### Synthesis of 3-hydroxythiophene compounds 3a-c

A suspension of each 2-(arylhydrazono)-2-ethoxycarbonyl-thioacetanilide derivative **2a**, **2b** or **2c** (0.002 mol) in sodium ethoxide solution (which was prepared from 0.05 g Na and 20 mL absolute ethanol) was stirred for 10 min and then *N*-(4-acetylphenyl)-2-chloroacetamide (**1**) (0.42 g, 0.002 mol) was added. The mixture was refluxed for 2 h then was permitted to be cool, and poured onto ice-water. The obtained product was dried out and crystallized from ethanol to give the targeted hydroxythiophenes **3a-c**.

### *N*-(4-Acetylphenyl)-3-hydroxy-5-phenylamino-4-(*p*-tolylazo)-thiophene-2-carboxamide (3a)

Red solid, yield = 56%, m.p. = 270–271 °C. IR (ν/cm^−1^): 3363 (N–H stretch), 1654 (C = O stretch). ^1^H NMR (δ/ppm): 2.34 (s, 3H, CH_3_), 2.52 (s, 3H, COCH_3_), 7.29 (d, *J* = 8.0 Hz, 5H, Ar–H), 7.44–7.52 (m, 4H, Ar–H and NH), 7.75 (t, *J* = 9.0 Hz, 3H, Ar–H), 7.93 (d, *J* = 8.5 Hz, 2H, Ar–H), 9.86 (s, 1H, NH), 12.38 (s, 1H, OH). ^13^C NMR (δ/ppm): 20.80, 26.46, 118.55 (3C), 118.92 (2C), 121.36 (4C), 125.93, 129.64 (4C), 129.72 (5C), 129.93 (3C), 160.83, 196.51. MS: m/z (%) = 470 (M^+^, 22.63), 430.94 (52.51), 405.13 (78.21), 400.79 (100.00), 284.79 (51.30), 284.28 (46.29), 254.68 (44.82), 244.49 (51.91), 237.61 (50.33), 236.67 (45.29), 175.37 (51.74), 172.90 (44.75), 90.64 (45.29), 40.61 (52.01). Analysis calculated for C_26_H_22_N_4_O_3_S (470.14): C, 66.37; H, 4.71; N, 11.91%. Found: C, 66.07; H, 4.63; N, 12.07%.

### *N*-(4-Acetylphenyl)-4-(*p*-anisylazo)-3-hydroxy-5-phenylamino-thiophene-2-carboxamide (3b)

Dark red solid, yield = 48%, m.p. = 234–235 °C. IR (ν/cm^−1^): 3366 (N–H stretch), 1654 (C = O stretch). ^1^H NMR (δ/ppm): 2.52 (s, 3H, COCH_3_), 3.84 (s, 3H, OCH_3_), 7.07 (d, *J* = 9.0 Hz, 3H, Ar–H), 7.26 (t, *J* = 7.5, 1H, Ar–H), 7.46–7.52 (m, 3H, Ar–H and NH), 7.74 (d, *J* = 8.5 Hz, 2H, Ar–H), 7.93 (d, *J* = 9.0 Hz, 5H, Ar–H), 9.73 (s, 1H, NH), 12.40 (s, 1H, OH). ^13^C NMR (δ/ppm): 26.48, 55.58, 114.62 (2C), 114.76, 118.55 (2C), 118.99, 121.13 (3C), 122.13 (2C), 125.63, 129.67 (4C), 129.79 (4C), 131.51, 153.91, 159.95, 196.50. Analysis calculated for C_26_H_22_N_4_O_4_S (486.14): C, 64.18; H, 4.56; N, 11.52%. Found: C, 63.96; H, 4.48; N, 11.68%.

### *N*-(4-Acetylphenyl)-4-((4-chlorophenyl)azo)-3-hydroxy-5-phenylamino-thiophene-2-carboxamide (3c)

Red crystals, yield = 52%, m.p. = 252–253 °C. IR (ν/cm^−1^): 3366 (N–H stretch), 1653 (C = O stretch). ^1^H NMR (δ/ppm): 2.52 (s, 3H, COCH_3_), 7.29 (t, *J* = 7.5 Hz, 1H, Ar–H), 7.42 (d, *J* = 7.0 Hz, 2H), 7.49–7.58 (m, 4H, Ar–H and NH), 7.74 (d, *J* = 8.0 Hz, 3H, Ar–H), 7.87 (d, *J* = 9.0 Hz, 2H, Ar–H), 7.93 (d, *J* = 9.0 Hz, 2H, Ar–H), 9.88 (s, 1H, NH), 12.47 (s, 1H, OH). Analysis calculated for C_25_H_19_ClN_4_O_3_S (490.09): C, 61.16; H, 3.90; N, 11.41%. Found: C, 61.32; H, 3.85; N, 11.50%.

### Synthesis of 3-methylthiophene compounds 5a-c

To a solution of each 2-acetyl-2-arylazo-thioacetanilide derivative **4a**, **4b** or **4c** (0.002 mol) in 20 mL dioxane, sodium methoxide (0.05 g, 0.001 mol) and *N*-(4-acetylphenyl)-2-chloroacetamide (**1**) (0.42 g, 0.002 mol) were added. The mixture was refluxed for an hour and then sodium methoxide (0.05 g, 0.001 mol) was added again. Refluxing was continued for additional 2 h and then the reaction mixture was permitted to cool. The formed solid product was collected to give the targeted methylthiophenes **5a-c**.

### *N*-(4-Acetylphenyl)-3-methyl-5-phenylamino-4-(*p*-tolylazo)thiophene-2-carboxamide (5a)

Pale red crystals, yield = 70%, m.p. = 290–291 °C. IR (ν/cm^−1^): 3447, 3277 (N–H stretch), 1675, 1640 (C = O). ^1^H NMR (δ/ppm): 2.32 (s, 3H, CH_3_), 2.49 (s, 3H, CH_3_), 2.51 (s, 3H, COCH_3_), 7.25 (d, *J* = 8.0 Hz, 2H, Ar–H), 7.30 (t, *J* = 8.5 Hz, 1H, Ar–H), 7.38 (d, *J* = 8.0 Hz, 2H, Ar–H), 7.48–7.55 (m, 4H, Ar–H), 7.75 (d, *J* = 8.5 Hz, 2H, Ar–H), 7.90 (d, *J* = 9.0 Hz, 2H, Ar–H). ^13^C NMR (δ/ppm): 12.94, 20.65, 26.47, 115.09, 116.81 (2C), 119.86 (2C), 120.86 (3C), 121.05 (2C), 125.23, 125.82, 129.26 (2C), 129.36 (2C), 129.66 (3C), 129.98, 130.07 (2C), 170.10, 196.56. Analysis calculated for C_27_H_24_N_4_O_2_S (468.16): C, 69.21; H, 5.16; N, 11.96%. Found: C, 69.43; H, 5.07; N, 12.11%.

### *N*-(4-Acetylphenyl)-4-(*p*-anisylazo)-3-methyl-5-phenylamino-thiophene-2-carboxamide (5b)

Red crystals, yield = 63%, m.p. = 319–320 °C. IR (ν/cm^−1^): 3447, 3289 (N–H stretch), 1676, 1639 (C = O). ^1^H NMR (δ/ppm): 2.52 (s, 3H, COCH_3_), 2.57 (s, 3H, CH_3_), 3.80 (s, 3H, OCH_3_), 7.05 (d, *J* = 8.5 Hz, 2H), 7.24 (t, *J* = 7.5 Hz, 1H, Ar–H), 7.42–7.51 (m, 4H, Ar–H), 7.70 (d, *J* = 9.0 Hz, 2H, Ar–H), 7.79 (d, *J* = 9.0 Hz, 2H, Ar–H), 7.91 (d, *J* = 9.0 Hz, 2H, Ar–H), 8.49 (s, 1H, NH), 10.28 (s, 1H, NH). ^13^C NMR (δ/ppm): 13.32, 26.50, 55.51, 114.78 (2C), 119.66 (2C), 120.14 (2C), 120.51 (3C), 125.33, 129.29 (3C), 129.76 (3C), 134.35, 141.41, 143.91, 158.76, 161.61, 166.67, 175.08, 196.59. MS: m/z (%) = 484 (M^+^, 5.10), 475.20 (41.81), 408.21 (33.89), 380.32( 31.14), 370.21 (76.02), 352.11 (30.03), 328.12 (34.06), 327.54 (59.69), 313.37 (55.63), 298.16 (48.14), 297.22 (52.83), 253.13 (44.02), 233.13 (30.90), 206.22 (30.80), 165.38 (85.57), 86.55 (63.81), 75.22 (100.00), 66.44 (83.76). Analysis calculated for C_27_H_24_N_4_O_3_S (484.16): C, 66.92; H, 4.99; N, 11.56%. Found: C, 66.75; H, 5.06; N, 11.67%.

### *N*-(4-Acetylphenyl)-4-((4-chlorophenyl)azo)-3-methyl-5-phenylamino-thiophene-2-carboxamide (5c)

Orange crystals, yield = 81%, m.p. = 270–271 °C. IR (ν/cm^−1^): 3448, 3277 (N–H stretch), 1674, 1638 (C = O). ^1^H NMR (δ/ppm): 2.45 (s, 3H, CH_3_), 2.52 (s, 3H, COCH_3_), 7.28 (t, *J* = 7.5 Hz, 1H, Ar–H), 7.39 (d, *J* = 8.0 Hz, 2H, Ar–H), 7.47–7.52 (m, 4H, Ar–H), 7.63 (d, *J* = 8.5 Hz, 2H, Ar–H), 7.78 (d, *J* = 8.5 Hz, 2H, Ar–H), 7.92 (d, *J* = 9.0 Hz, 2H, Ar–H), 8.50 (s, 1H, NH), 10.27 (s, 1H, NH). Analysis calculated for C_26_H_21_ClN_4_O_2_S (488.11): C, 63.86; H, 4.33; N, 11.46%. Found: C, 63.64; H, 4.28; N, 11.61%.

### Synthesis of 3-aminothiophene compounds 7a-c

A mixture of each 2-cyano-3-mercapto-*N*-phenyl-3-(phenylamino)acrylamide derivative **6a**, **6b** or **6c** (0.002 mol), *N*-(4-acetylphenyl)-2-chloroacetamide (0.44 g, 0.002 mol) and sodium methoxide (0.11 g, 0.002 mol) was refluxed in 20 mL dioxane for 2 h. The mixture was poured into ice-water. The formed solid was collected and recrystallized from ethanol to produce the corresponding 3-aminothiophene compounds **7a-c**.

### ***N***^2^-(4-Acetylphenyl)-3-amino-***N***^4^-phenyl-5-phenylaminothiophene-2,4-dicarboxamide (7a)

White crystals, yield = 47%, m.p. = 190–191 °C. IR (ν/cm^−1^): 3346, 3276, 3192 (NH_2_ & NH), 1654 (broad, C = O stretch). ^1^H NMR (δ/ppm): 2.51 (s, 3H, COCH_3_), 5.12 (s, 1H, NH), 7.03–7.08 (m, 4H, Ar–H), 7.29–7.38 (m, 4H, Ar–H), 7.57 (d, *J* = 7.5 Hz, 2H, Ar–H), 7.68 (d, *J* = 8.5 Hz, 2H, Ar–H), 7.94 (d, *J* = 8.5 Hz, 2H, Ar–H), 8.80 (s, 1H, NH), 9.31 (s, 1H, NH), 10.71 (s, 1H, NH), 11.85 (s, 1H, NH). ^13^C NMR (δ/ppm): 26.50, 118.54 (2C), 119.44 (2C), 119.94, 120.29, 121.21 (2C), 123.21, 124.50, 128.49, 129.01 (2C), 129.28 (2C), 129.63 (2C), 132.20, 138.58, 142.88, 149.95, 163.38, 165.85, 167.48, 196.57. MS: m/z (%) = 470 (M^+^, 25.44), 453.06 (58.69), 449 (63.16), 44.27 (52.26), 429.94 (52.39), 354.88 (52.44), 174.09 (62.15), 147.41 (75.00), 117.02 (57.18), 104.56 (100), 43.95 (72.07). Analysis calculated for C_26_H_22_N_4_O_3_S (470.14): C, 66.37; H, 4.71; N, 11.91%. Found: C, 66.48; H, 4.65; N, 11.82%.

### ***N***^2^-(4-Acetylphenyl)-3-amino-5-phenylamino-***N***^4^-(***p***-tolyl)thiophene-2,4-dicarboxamide (7b)

Yellow solid, yield = 54%, m.p. = 230–231 °C. IR (ν/cm^−1^): 3390, 3252 (NH_2_ & NH), 1670, 1649 (C = O stretch). ^1^H NMR (δ/ppm): 2.24 (s, 3H, CH_3_), 2.51 (s, 3H, COCH_3_), 5.10 (s, 1H, NH), 7.06–7.14 (m, 3H, Ar–H), 7.36–7.52 (m, 6H, Ar–H), 7.67 (d, *J* = 9.0 Hz, 2H, Ar–H), 7.94 (d, *J* = 9.0 Hz, 2H, Ar–H), 8.76 (s, 1H, NH), 9.33 (s, 1H, NH), 10.71 (s, 1H, NH), 11.75 (s, 1H, NH). Analysis calculated for C_27_H_24_N_4_O_3_S (484.16): C, 66.92; H, 4.99; N, 11.56%. Found: C, 67.08; H, 5.06; N, 11.45%.

### ***N***^2^-(4-Acetylphenyl)-3-amino-***N***^4^-(***p***-anisyl)-5-phenylamino-thiophene-2,4-dicarboxamide (7c)

Orange solid, yield = 62%, m.p. = 244–245 °C. IR (ν/cm^−1^): 3348, 3234 (NH_2_ & NH), 1646 (broad, C = O stretch). ^1^H NMR (δ/ppm): 2.52 (s, 3H, COCH_3_), 3.77 (s, 3H, OCH_3_), 5.12 (s, 1H, NH), 6.94 (d, *J* = 9.0 Hz, 2H, Ar–H), 7.38–7.43 (m, 5H, Ar–H), 7.68 (d, *J* = 9.0 Hz, 2H, Ar–H), 7.74 (d, *J* = 9.0 Hz, 2H, Ar–H), 7.98 (d, *J* = 9.0 Hz, 2H, Ar–H), 8.78 (s, 1H, NH), 9.25 (s, 1H, NH), 10.70 (s, 1H, NH), 11.81 (s, 1H, NH). Analysis calculated for C_27_H_24_N_4_O_4_S (500.15): C, 64.79; H, 4.83; N, 11.19%. Found: C, 64.84; H, 4.89; N, 11.31%.

### Computational details

Quantum chemical calculations for the synthesized compounds were used to optimize the geometry by Gaussian 09W suite program [66] using the Becke3–Lee–Yang–Parr (B3LYP) exchange–correlation functional [67–69] with standard 6–311 +  + G (d,p) basis set. The HOMO–LUMO plots and Mulliken’s atomic charges data were obtained using the GaussView program [70]. The Fukui indices were determined by Materials studio package DMol3 module [71] utilizing the GGA and B3LYP functional with DNP (version 3.5) [72].

### Docking method

All the molecular modeling studies were carried out using Molecular Operating Environment (MOE, 2015.01) software.The three-dimensional structure (3D) of the selected proteins (PDB ID 2AS1), (PDB ID 1DD6), (PDB ID 2MLM), (PDB ID 3ZMI), (PDB ID 4D3V) were downloaded from the PDB website. The water molecules and repeated chains were removed. Protons were added and the energy of the protein was minimized. The preparation of thiophene 2-carboxamide derivatives **3a-c**, **5a-c** and **7a-c** for docking were carried out by energy minimization and potential energy calculation inside MOE propgram. MOE conducted the docking of the newly synthesized compounds, calculated the binding energies, and provided their binding modes.

## Supplementary Information

**Additional file 1: **Experimental general remarks. **Table S1.** Bond length, bond angle, dihedral angle for compounds **3a-c**. **Table S2.** Bond length, bond angle, dihedral angle for compounds **5a-c**. **Table S3.** Bond length, bond angle, dihedral angle for compounds **7a-c**. **Table S4.** The atomic Mulliken’s charges and Fukui’s indices of investigated compounds. **Fig. S1**. DFT optimized structures for compounds **3a-c**, **5a-c**, and **7a-c**. **Fig. S2**. The binding interaction of 3-hydroxythiophene **3a** with (PDB ID: 2AS1). **Fig. S3**. The binding interaction of 3-hydroxythiophene **5a** with (PDB ID: 2AS1). **Fig. S4.** The binding interaction of 3-hydroxythiophene **5b** with (PDB ID: 2AS1). **Fig. S5**. The binding interaction of 3-hydroxythiophene **5c** with (PDB ID: 2AS1). **Fig. S6.** The binding interaction of 3-hydroxythiophene **7a** with (PDB ID: 2AS1). **Fig. S7**. The binding interaction of 3-hydroxythiophene **7b** with (PDB ID: 2AS1). **Fig. S8.** The binding interaction of 3-hydroxythiophene **7c** with (PDB ID: 2AS1). **Fig. S9.** The binding interaction ascorbic acid with (PDB ID: 2AS1). **Fig. S10.** The binding interaction of 3-hydroxythiophene **3a** with (PDB ID: 1DD6). **Fig. S11.** The binding interaction of 3-hydroxythiophene **3b** with (PDB ID: 1DD6). **Fig. S12.** The binding interaction of 3-hydroxythiophene **3c** with (PDB ID: 1DD6). **Fig. S13.** The binding interaction of 3-methylthiophene **5b** with (PDB ID: 1DD6). **Fig. S14.** The binding interaction of 3-methylthiophene **5c** with (PDB ID: 1DD6). **Fig. S15.** The binding interaction of 3-aminothiophene **7a** with (PDB ID: 1DD6). **Fig. S16.** The binding interaction of 3-aminothiophene **7b** with (PDB ID: 1DD6). **Fig. S17**. The binding interaction of 3-aminothiophene **7c** with (PDB ID: 1DD6). **Fig. S18.** The binding interaction of with ampicillin (PDB ID: 1DD6). **Fig. S19**. The binding interaction of 3-hydroxythiophene **3a** with (PDB ID: 2MLM). **Fig. S20.** The binding interaction of 3-hydroxythiophene **3b** with (PDB ID: 2MLM). **Fig. S21.** The binding interaction of 3-hydroxythiophene **3c** with (PDB ID: 2MLM). **Fig. S22.** The binding interaction of 3-methylthiophene **5a** with (PDB ID: 2MLM). **Fig. S23.** The binding interaction of 3-methylthiophene **5b** with (PDB ID: 2MLM). **Fig. S24.** The binding interaction of 3-methylthiophene **5c** with (PDB ID: 2MLM). **Fig. S25.** The binding interaction of 3-methylthiophene **7b** with (PDB ID: 2MLM). **Fig. S26.** The binding interaction of 3-methylthiophene **7c** with (PDB ID: 2MLM). **Fig. S27.** The binding interaction of ampicillin with (PDB ID: 2MLM). **Fig. S28.** The binding interaction of 3-hydroxythiophene **3a** with (PDB ID: 3ZMI). **Fig. S29.** The binding interaction of 3-hydroxythiophene **3b** with (PDB ID: 3ZMI). **Fig. S30.** The binding interaction of 3-hydroxythiophene **3c** with (PDB ID: 3ZMI). **Fig. S31.** The binding interaction of 3-methylthiophene **5a** with (PDB ID: 3ZMI). **Fig. S32.** The binding interaction of 3-methylthiophene **5b** with (PDB ID: 3ZMI). **Fig. S33.** The binding interaction of 3-methylthiophene **5c** with (PDB ID: 3ZMI). **Fig. S34.** The binding interaction of 3-aminothiophene **7a** with (PDB ID: 3ZMI). **Fig. S35.** The binding interaction of 3-aminothiophene **7b** with (PDB ID: 3ZMI). **Fig. S36.** The binding interaction of with ampicillin (PDB ID: 3ZMI). **Fig. S37.** The binding interaction of 3-hydroxythiophene **3b** with (PDB ID: 4d3v). **Fig. S38.** The binding interaction of 3-hydroxythiophene **3c** with (PDB ID: 4d3v). **Fig. S39.** The binding interaction of 3-methylthiophene **5a** with (PDB ID: 4d3v). **Fig. S40.** The binding interaction of 3-methylthiophene **5b** with (PDB ID: 4d3v). **Fig. S41**. The binding interaction of 3-methylthiophene **5c** with (PDB ID: 4d3v). **Fig. S42.** The binding interaction of 3-aminothiophene **7a** with (PDB ID: 4d3v). **Fig. S43.** The binding interaction of 3-aminothiophene **7b** with (PDB ID: 4d3v). **Fig. S44**. The binding interaction of 3-aminothiophene **7c** with (PDB ID: 4d3v). **Fig. S45.** The binding interaction of with ampicillin (PDB ID: 4d3v). **Fig. S46.** IR spectrum of compound **3a**. **Fig. S47.** ^1^H NMR spectrum of compound **3a. Fig. S48.** ^13^C NMR spectrum of compound **3a. Fig. S49**. IR spectrum of compound **3b. Fig. S50.** ^1^H NMR spectrum of compound **3b. Fig. S51.** ^13^C NMR spectrum of compound **3b. Fig. S52.** IR spectrum of compound **3c. Fig. S53.** ^1^H NMR spectrum of compound **3c. Fig. S54.** IR spectrum of compound **5a. Fig. S55.** ^1^H NMR spectrum of compound **5a. Fig. S56.** ^13^C NMR spectrum of compound **5a. Fig. S57.** IR spectrum of compound **5b. Fig. S58.** ^1^H NMR spectrum of compound **5b. Fig. S59.** ^13^C NMR spectrum of compound **5b. Fig. S60.** IR spectrum of compound **5c. Fig. S61.** ^1^H NMR spectrum of compound **5c. Fig. S62**. IR spectrum of compound **7a. Fig. S63.** ^1^H NMR spectrum of compound **7a. Fig. S64.** ^13^C NMR spectrum of compound **7a. Fig. S65.** IR spectrum of compound **7b. Fig. S66.** ^1^H NMR spectrum of compound **7b. Fig. S67.** IR spectrum of compound **7c. Fig. S68.** ^1^H NMR spectrum of compound** 7c.**

## Data Availability

The datasets used and/or analyzed during the current study available from the corresponding author on reasonable request.
