# Supplementary material for: Synthesis, DFT investigations, antioxidant, antibacterial activity and SAR-study of novel thiophene-2-carboxamide derivatives
Source: BMC Chem. 2023 Feb 20;17(1):6. doi: 10.1186/s13065-023-00917-2 (PMC9940361; doi:10.1186/s13065-023-00917-2)
Supplement: Supplementary file 1 — Additional file 1: Experimental general remarks. Table S1. Bond length, bond angle, dihedral angle for compounds 3a-c. Table S2. Bond length, bond angle, dihedral angle for compounds 5a-c. Table S3. Bond length, bond angle, dihedral angle for compounds 7a-c. Table S4. The atomic Mulliken’s charges and Fukui’s indices of investigated compounds. Fig. S1. DFT optimized structures for compounds 3a-c, 5a-c, and 7a-c. Fig. S2. The binding interaction of 3-hydroxythiophene 3a with (PDB ID: 2AS1). Fig. S3. The binding interaction of 3-hydroxythiophene 5a with (PDB ID: 2AS1). Fig. S4. The binding interaction of 3-hydroxythiophene 5b with (PDB ID: 2AS1). Fig. S5. The binding interaction of 3-hydroxythiophene 5c with (PDB ID: 2AS1). Fig. S6. The binding interaction of 3-hydroxythiophene 7a with (PDB ID: 2AS1). Fig. S7. The binding interaction of 3-hydroxythiophene 7b with (PDB ID: 2AS1). Fig. S8. The binding interaction of 3-hydroxythiophene 7c with (PDB ID: 2AS1). Fig. S9. The binding interaction ascorbic acid with (PDB ID: 2AS1). Fig. S10. The binding interaction of 3-hydroxythiophene 3a with (PDB ID: 1DD6). Fig. S11. The binding interaction of 3-hydroxythiophene 3b with (PDB ID: 1DD6). Fig. S12. The binding interaction of 3-hydroxythiophene 3c with (PDB ID: 1DD6). Fig. S13. The binding interaction of 3-methylthiophene 5b with (PDB ID: 1DD6). Fig. S14. The binding interaction of 3-methylthiophene 5c with (PDB ID: 1DD6). Fig. S15. The binding interaction of 3-aminothiophene 7a with (PDB ID: 1DD6). Fig. S16. The binding interaction of 3-aminothiophene 7b with (PDB ID: 1DD6). Fig. S17. The binding interaction of 3-aminothiophene 7c with (PDB ID: 1DD6). Fig. S18. The binding interaction of with ampicillin (PDB ID: 1DD6). Fig. S19. The binding interaction of 3-hydroxythiophene 3a with (PDB ID: 2MLM). Fig. S20. The binding interaction of 3-hydroxythiophene 3b with (PDB ID: 2MLM). Fig. S21. The binding interaction of 3-hydroxythiophene 3c with (PDB ID: 2MLM). Fig. S22. The binding [file 13065_2023_917_MOESM1_ESM.docx]

**Supporting Information**

**Synthesis, DFT investigations, antioxidant, antibacterial activity and SAR-study of novel** **thiophene-2-carboxamide derivatives**

**Heba M. Metwally*,** **Ehab Abdel-Latif, Norhan A. Khalaf, Mohamed A. Ismail**

Department of Chemistry, Faculty of Science, Mansoura University, 35516 Mansoura, Egypt

**Corresponding: hebama@mans.edu.eg*

**Chemistry**

**Experimental general remarks:** Melting points were determined with Gallenkamp melting point apparatus and are uncorrected. The infrared (IR) spectra were recorded on Thermo Scientific Nicolet iS10 FTIR. ^1^H NMR and ^13^C NMR spectra were recorded DMSO-*d*_6_ as a solvent using JEOL’s spectrometer at 500 MHz using tetramethylsilane (TMS) as internal standard. Chemical shifts are expressed in δ, ppm. ^1^H NMR data are reported in order: multiplicity (br, broad; s, singlet; d, doublet; t, triplet; dd, doublet of doublet; m, multiplet), approximate coupling constant in Hertz, number of protons and type of protons. The purity of the compounds was checked by ^1^H NMR and thin layer chromatography (TLC) on silica gel plates using a mixture of dichloromethane and methanol or petroleum ether and ethyl acetate as eluent. UV lamp was used as a visualizing agent. Mass analyses and elemental analyses were recorded on Thermo DSQ II spectrometer at Faculty of Science, Alazhar University.

**Computational Study**

**Molecular Modelling**

**Table S1** Bond length, bond angle, dihedral angle for compounds **3a-c**

| **3a Bond Length Bond Angle Dihedral Angle** | | | | | | | | |
| --- | --- | --- | --- | --- | --- | --- | --- | --- |
| **Atoms** | **Actual (°/A°)** | **Optimal (°/A°)** | **Atoms** | **Actual (°/A°)** | **Optimal (°/A°)** |  | **Atoms** | **Actual (°/A°)** |
| C(34)-H(56) | 1.08 | 1.10 | H(56)-C(34)-C(33) | 119.35 | 120 |  | C(32)-C(33)-C(34)-C(29) | -0.81 |
| C(33)-H(55) | 1.08 | 1.10 | H(56)-C(34)-C(29) | 120.78 | 120 |  | C(32)-C(33)-C(34)-H(56) | 177.07 |
| C(33)-C(34) | 1.39 | 1.42 | C(33)-C(34)-C(29) | 119.83 |  |  | H(55)-C(33)-C(34)-C(29) | -179.91 |
| C(32)-H(54) | 1.08 | 1.10 | H(55)-C(33)-C(34) | 119.00 | 120 |  | H(55)-C(33)-C(34)-H(56) | -2.03 |
| C(32)-C(33) | 1.39 | 1.42 | H(55)-C(33)-C(32) | 120.03 | 120 |  | C(31)-C(32)-C(33)-C(34) | -0.15 |
| C(31)-H(53) | 1.08 | 1.10 | C(34)-C(33)-C(32) | 120.96 |  |  | C(31)-C(32)-C(33)-H(55) | 178.95 |
| C(31)-C(32) | 1.40 | 1.42 | H(54)-C(32)-C(33) | 120.43 | 120 |  | H(54)-C(32)-C(33)-C(34) | -179.80 |
| C(30)-H(52) | 1.09 | 1.10 | H(54)-C(32)-C(31) | 120.41 | 120 |  | H(54)-C(32)-C(33)-H(55) | -0.71 |
| C(30)-C(31) | 1.39 | 1.42 | C(33)-C(32)-C(31) | 119.16 |  |  | C(30)-C(31)-C(32)-C(33) | 0.76 |
| C(29)-C(34) | 1.40 | 1.42 | H(53)-C(31)-C(32) | 120.19 | 120 |  | C(30)-C(31)-C(32)-H(54) | -179.58 |
| C(29)-C(30) | 1.40 | 1.42 | H(53)-C(31)-C(30) | 119.36 | 120 |  | H(53)-C(31)-C(32)-C(33) | -179.76 |
| N(28)-H(51) | 1.02 | 1.05 | C(32)-C(31)-C(30) | 120.45 |  |  | H(53)-C(31)-C(32)-H(54) | -0.11 |
| N(28)-C(29) | 1.40 | 1.46 | H(52)-C(30)-C(31) | 120.13 | 120 |  | C(29)-C(30)-C(31)-C(32) | -0.42 |
| C(27)-H(50) | 1.09 | 1.11 | H(52)-C(30)-C(29) | 119.46 | 120 |  | C(29)-C(30)-C(31)-H(53) | -179.90 |
| C(27)-H(49) | 1.10 | 1.11 | C(31)-C(30)-C(29) | 120.41 |  |  | H(52)-C(30)-C(31)-C(32) | 178.98 |
| C(27)-H(48) | 1.09 | 1.11 | C(34)-C(29)-C(30) | 119.17 | 120 |  | H(52)-C(30)-C(31)-H(53) | -0.50 |
| C(26)-H(47) | 1.09 | 1.11 | C(34)-C(29)-N(28) | 123.44 | 120 |  | N(28)-C(29)-C(34)-C(33) | 179.40 |
| C(26)-H(46) | 1.09 | 1.11 | C(30)-C(29)-N(28) | 117.37 | 120 |  | N(28)-C(29)-C(34)-H(56) | 1.55 |
| C(26)-H(45) | 1.09 | 1.11 | H(51)-N(28)-C(29) | 116.89 | 118 |  | C(30)-C(29)-C(34)-C(33) | 1.13 |
| C(24)-H(44) | 1.08 | 1.10 | H(51)-N(28)-C(3) | 111.05 | 118 |  | C(30)-C(29)-C(34)-H(56) | -176.71 |
| C(23)-H(43) | 1.09 | 1.10 | C(29)-N(28)-C(3) | 132.03 | 124 |  | N(28)-C(29)-C(30)-C(31) | -178.90 |
| C(23)-C(24) | 1.39 | 1.34 | H(50)-C(27)-H(49) | 107.14 | 109 |  | N(28)-C(29)-C(30)-H(52) | 1.69 |
| C(22)-C(27) | 1.51 | 1.52 | H(50)-C(27)-H(48) | 107.97 | 109 |  | C(34)-C(29)-C(30)-C(31) | -0.53 |
| C(22)-C(23) | 1.41 | 1.50 | H(50)-C(27)-C(22) | 111.24 | 110 |  | C(34)-C(29)-C(30)-H(52) | -179.94 |
| C(21)-H(42) | 1.08 | 1.11 | H(49)-C(27)-H(48) | 107.79 | 109 |  | C(3)-N(28)-C(29)-C(30) | -158.16 |
| C(21)-C(22) | 1.40 | 1.52 | H(49)-C(27)-C(22) | 111.10 | 110 |  | C(3)-N(28)-C(29)-C(34) | 23.55 |
| C(20)-H(41) | 1.08 | 1.11 | H(48)-C(27)-C(22) | 111.42 | 110 |  | H(51)-N(28)-C(29)-C(30) | 19.63 |
| C(20)-C(21) | 1.39 | 1.52 | H(47)-C(26)-H(46) | 109.31 | 109 |  | H(51)-N(28)-C(29)-C(34) | -158.66 |
| O(19)-H(40) | 0.99 | 0.97 | H(47)-C(26)-H(45) | 107.53 | 109 |  | C(22)-C(23)-C(24)-C(6) | 0.04 |
| C(18)-O(19) | 1.33 | 1.36 | H(47)-C(26)-C(17) | 111.00 | 109.47 |  | C(22)-C(23)-C(24)-H(44) | 179.29 |
| C(17)-C(26) | 1.52 | 1.51 | H(46)-C(26)-H(45) | 109.31 | 109 |  | H(43)-C(23)-C(24)-C(6) | -179.59 |
| C(17)-O(25) | 1.22 | 1.21 | H(46)-C(26)-C(17) | 108.66 | 109.47 |  | H(43)-C(23)-C(24)-H(44) | -0.35 |
| C(16)-H(39) | 1.08 | 1.11 | H(45)-C(26)-C(17) | 111.00 | 109.47 |  | C(21)-C(22)-C(27)-H(48) | -4.74 |
| C(15)-H(38) | 1.08 | 1.11 | H(44)-C(24)-C(23) | 120.54 | 120 |  | C(21)-C(22)-C(27)-H(49) | 115.46 |
| C(15)-C(16) | 1.39 | 1.52 | H(44)-C(24)-C(6) | 119.52 | 118.2 |  | C(21)-C(22)-C(27)-H(50) | -125.28 |
| C(14)-C(17) | 1.49 | 1.51 | C(23)-C(24)-C(6) | 119.94 | 122 |  | C(23)-C(22)-C(27)-H(48) | 175.24 |
| C(14)-C(15) | 1.40 | 1.52 | H(43)-C(23)-C(24) | 119.34 | 120 |  | C(23)-C(22)-C(27)-H(49) | -64.55 |
| C(13)-H(37) | 1.08 | 1.10 | H(43)-C(23)-C(22) | 119.19 | 118.2 |  | C(23)-C(22)-C(27)-H(50) | 54.71 |
| C(13)-C(14) | 1.40 | 1.50 | C(24)-C(23)-C(22) | 121.47 | 122 |  | C(21)-C(22)-C(23)-C(24) | -0.36 |
| C(12)-H(36) | 1.09 | 1.10 | C(27)-C(22)-C(23) | 120.51 | 109.51 |  | C(21)-C(22)-C(23)-H(43) | 179.27 |
| C(12)-C(13) | 1.39 | 1.34 | C(27)-C(22)-C(21) | 121.43 | 109.51 |  | C(27)-C(22)-C(23)-C(24) | 179.65 |
| C(11)-C(16) | 1.40 | 1.52 | C(23)-C(22)-C(21) | 118.06 | 109.51 |  | C(27)-C(22)-C(23)-H(43) | -0.72 |
| C(11)-C(12) | 1.40 | 1.50 | H(42)-C(21)-C(22) | 119.53 | 109.41 |  | C(20)-C(21)-C(22)-C(23) | 0.10 |
| N(9)-H(35) | 1.01 | 1.02 | H(42)-C(21)-C(20) | 119.47 | 109.41 |  | C(20)-C(21)-C(22)-C(27) | -179.91 |
| N(9)-C(11) | 1.40 | 1.46 | C(22)-C(21)-C(20) | 121.01 | 109.5 |  | H(42)-C(21)-C(22)-C(23) | -179.62 |
| C(8)-O(10) | 1.24 | 1.21 | H(41)-C(20)-C(21) | 121.27 | 109.41 |  | H(42)-C(21)-C(22)-C(27) | 0.37 |
| C(8)-N(9) | 1.38 | 1.37 | H(41)-C(20)-C(6) | 118.26 | 109.41 |  | C(6)-C(20)-C(21)-C(22) | 0.47 |
| C(7)-C(18) | 1.39 | 1.42 | C(21)-C(20)-C(6) | 120.47 | 109.5 |  | C(6)-C(20)-C(21)-H(42) | -179.80 |
| C(7)-C(8) | 1.45 | 1.52 | H(40)-O(19)-C(18) | 105.76 | 108 |  | H(41)-C(20)-C(21)-C(22) | -179.65 |
| C(6)-C(24) | 1.41 | 1.50 | O(19)-C(18)-C(7) | 123.47 | 124.3 |  | H(41)-C(20)-C(21)-H(42) | 0.07 |
| C(6)-C(20) | 1.40 | 1.52 | O(19)-C(18)-C(2) | 123.29 | 124.3 |  | C(2)-C(18)-O(19)-H(40) | 179.08 |
| N(5)-C(6) | 1.41 | 1.47 | C(7)-C(18)-C(2) | 113.24 | 120 |  | C(7)-C(18)-O(19)-H(40) | -0.47 |
| N(4)-N(5) | 1.26 | 1.25 | C(26)-C(17)-O(25) | 120.33 | 122.5 |  | C(14)-C(17)-C(26)-H(45) | 59.76 |
| C(3)-N(28) | 1.36 | 1.46 | C(26)-C(17)-C(14) | 118.80 | 116.6 |  | C(14)-C(17)-C(26)-H(46) | 180.00 |
| C(2)-C(18) | 1.44 | 1.42 | O(25)-C(17)-C(14) | 120.87 | 122.5 |  | C(14)-C(17)-C(26)-H(47) | -59.77 |
| C(2)-N(4) | 1.38 | 1.46 | H(39)-C(16)-C(15) | 120.93 | 109.41 |  | O(25)-C(17)-C(26)-H(45) | -120.23 |
| C(2)-C(3) | 1.40 | 1.42 | H(39)-C(16)-C(11) | 119.65 | 109.41 |  | O(25)-C(17)-C(26)-H(46) | 0.00 |
| S(1)-C(7) | 1.77 | 1.66 | C(15)-C(16)-C(11) | 119.42 | 109.5 |  | O(25)-C(17)-C(26)-H(47) | 120.24 |
| S(1)-C(3) | 1.74 | 1.66 | H(38)-C(15)-C(16) | 119.85 | 109.41 |  | C(14)-C(15)-C(16)-C(11) | -0.01 |
|  |  |  | H(38)-C(15)-C(14) | 118.18 | 109.41 |  | C(14)-C(15)-C(16)-H(39) | -179.97 |
|  |  |  | C(16)-C(15)-C(14) | 121.97 | 109.5 |  | H(38)-C(15)-C(16)-C(11) | 180.00 |
|  |  |  | C(17)-C(14)-C(15) | 119.01 | 109.9 |  | H(38)-C(15)-C(16)-H(39) | 0.03 |
|  |  |  | C(17)-C(14)-C(13) | 122.94 | 110.51 |  | C(13)-C(14)-C(17)-O(25) | -179.98 |
|  |  |  | C(15)-C(14)-C(13) | 118.05 | 109.51 |  | C(13)-C(14)-C(17)-C(26) | 0.03 |
|  |  |  | H(37)-C(13)-C(14) | 120.62 | 118.2 |  | C(15)-C(14)-C(17)-O(25) | 0.05 |
|  |  |  | H(37)-C(13)-C(12) | 118.62 | 120 |  | C(15)-C(14)-C(17)-C(26) | -179.95 |
|  |  |  | C(14)-C(13)-C(12) | 120.76 | 122 |  | C(13)-C(14)-C(15)-C(16) | 0.01 |
|  |  |  | H(36)-C(12)-C(13) | 119.62 | 120 |  | C(13)-C(14)-C(15)-H(38) | -179.99 |
|  |  |  | H(36)-C(12)-C(11) | 119.71 | 118.2 |  | C(17)-C(14)-C(15)-C(16) | 179.99 |
|  |  |  | C(13)-C(12)-C(11) | 120.67 | 122 |  | C(17)-C(14)-C(15)-H(38) | -0.02 |
|  |  |  | C(16)-C(11)-C(12) | 119.13 | 109.51 |  | C(12)-C(13)-C(14)-C(15) | -0.01 |
|  |  |  | C(16)-C(11)-N(9) | 123.98 | 110.78 |  | C(12)-C(13)-C(14)-C(17) | -179.99 |
|  |  |  | C(12)-C(11)-N(9) | 116.89 |  |  | H(37)-C(13)-C(14)-C(15) | 179.98 |
|  |  |  | H(35)-N(9)-C(11) | 114.22 | 118 |  | H(37)-C(13)-C(14)-C(17) | 0.00 |
|  |  |  | H(35)-N(9)-C(8) | 116.26 | 117.4 |  | C(11)-C(12)-C(13)-C(14) | 0.00 |
|  |  |  | C(11)-N(9)-C(8) | 129.53 |  |  | C(11)-C(12)-C(13)-H(37) | -179.99 |
|  |  |  | O(10)-C(8)-N(9) | 122.23 | 122.6 |  | H(36)-C(12)-C(13)-C(14) | 179.98 |
|  |  |  | O(10)-C(8)-C(7) | 119.95 | 123 |  | H(36)-C(12)-C(13)-H(37) | -0.01 |
|  |  |  | N(9)-C(8)-C(7) | 117.82 | 112.74 |  | N(9)-C(11)-C(16)-C(15) | 179.96 |
|  |  |  | C(18)-C(7)-C(8) | 122.30 | 117.6 |  | N(9)-C(11)-C(16)-H(39) | -0.07 |
|  |  |  | C(18)-C(7)-S(1) | 111.26 | 119 |  | C(12)-C(11)-C(16)-C(15) | 0.00 |
|  |  |  | C(8)-C(7)-S(1) | 126.42 |  |  | C(12)-C(11)-C(16)-H(39) | 179.96 |
|  |  |  | C(24)-C(6)-C(20) | 119.04 | 109.51 |  | N(9)-C(11)-C(12)-C(13) | -179.96 |
|  |  |  | C(24)-C(6)-N(5) | 125.63 |  |  | N(9)-C(11)-C(12)-H(36) | 0.06 |
|  |  |  | C(20)-C(6)-N(5) | 115.32 |  |  | C(16)-C(11)-C(12)-C(13) | 0.01 |
|  |  |  | C(6)-N(5)-N(4) | 115.45 | 106.5 |  | C(16)-C(11)-C(12)-H(36) | -179.97 |
|  |  |  | N(5)-N(4)-C(2) | 117.44 | 107.5 |  | C(8)-N(9)-C(11)-C(12) | -179.74 |
|  |  |  | N(28)-C(3)-C(2) | 122.05 | 120 |  | C(8)-N(9)-C(11)-C(16) | 0.30 |
|  |  |  | N(28)-C(3)-S(1) | 125.31 |  |  | H(35)-N(9)-C(11)-C(12) | 0.06 |
|  |  |  | C(2)-C(3)-S(1) | 112.55 | 119 |  | H(35)-N(9)-C(11)-C(16) | -179.90 |
|  |  |  | C(18)-C(2)-N(4) | 131.81 | 120 |  | C(7)-C(8)-N(9)-C(11) | -179.91 |
|  |  |  | C(18)-C(2)-C(3) | 111.83 | 120 |  | C(7)-C(8)-N(9)-H(35) | 0.30 |
|  |  |  | N(4)-C(2)-C(3) | 116.35 | 120 |  | O(10)-C(8)-N(9)-C(11) | 0.18 |
|  |  |  | C(7)-S(1)-C(3) | 91.12 | 98.5 |  | O(10)-C(8)-N(9)-H(35) | -179.62 |
|  |  |  |  |  |  |  | S(1)-C(7)-C(18)-C(2) | 0.14 |
|  |  |  |  |  |  |  | S(1)-C(7)-C(18)-O(19) | 179.73 |
|  |  |  |  |  |  |  | C(8)-C(7)-C(18)-C(2) | -178.33 |
|  |  |  |  |  |  |  | C(8)-C(7)-C(18)-O(19) | 1.26 |
|  |  |  |  |  |  |  | S(1)-C(7)-C(8)-N(9) | 0.79 |
|  |  |  |  |  |  |  | S(1)-C(7)-C(8)-O(10) | -179.30 |
|  |  |  |  |  |  |  | C(18)-C(7)-C(8)-N(9) | 179.01 |
|  |  |  |  |  |  |  | C(18)-C(7)-C(8)-O(10) | -1.07 |
|  |  |  |  |  |  |  | N(5)-C(6)-C(24)-C(23) | 179.58 |
|  |  |  |  |  |  |  | N(5)-C(6)-C(24)-H(44) | 0.33 |
|  |  |  |  |  |  |  | C(20)-C(6)-C(24)-C(23) | 0.53 |
|  |  |  |  |  |  |  | C(20)-C(6)-C(24)-H(44) | -178.72 |
|  |  |  |  |  |  |  | N(5)-C(6)-C(20)-C(21) | -179.93 |
|  |  |  |  |  |  |  | N(5)-C(6)-C(20)-H(41) | 0.19 |
|  |  |  |  |  |  |  | C(24)-C(6)-C(20)-C(21) | -0.79 |
|  |  |  |  |  |  |  | C(24)-C(6)-C(20)-H(41) | 179.34 |
|  |  |  |  |  |  |  | N(4)-N(5)-C(6)-C(20) | -174.14 |
|  |  |  |  |  |  |  | N(4)-N(5)-C(6)-C(24) | 6.78 |
|  |  |  |  |  |  |  | C(2)-N(4)-N(5)-C(6) | -179.41 |
|  |  |  |  |  |  |  | S(1)-C(3)-N(28)-C(29) | 6.19 |
|  |  |  |  |  |  |  | S(1)-C(3)-N(28)-H(51) | -171.70 |
|  |  |  |  |  |  |  | C(2)-C(3)-N(28)-C(29) | -177.58 |
|  |  |  |  |  |  |  | C(2)-C(3)-N(28)-H(51) | 4.53 |
|  |  |  |  |  |  |  | C(3)-C(2)-C(18)-C(7) | 0.00 |
|  |  |  |  |  |  |  | C(3)-C(2)-C(18)-O(19) | -179.59 |
|  |  |  |  |  |  |  | N(4)-C(2)-C(18)-C(7) | -178.93 |
|  |  |  |  |  |  |  | N(4)-C(2)-C(18)-O(19) | 1.48 |
|  |  |  |  |  |  |  | C(3)-C(2)-N(4)-N(5) | -178.45 |
|  |  |  |  |  |  |  | C(18)-C(2)-N(4)-N(5) | 0.44 |
|  |  |  |  |  |  |  | N(4)-C(2)-C(3)-S(1) | 178.97 |
|  |  |  |  |  |  |  | N(4)-C(2)-C(3)-N(28) | 2.29 |
|  |  |  |  |  |  |  | C(18)-C(2)-C(3)-S(1) | -0.15 |
|  |  |  |  |  |  |  | C(18)-C(2)-C(3)-N(28) | -176.82 |
|  |  |  |  |  |  |  | C(3)-S(1)-C(7)-C(8) | 178.20 |
|  |  |  |  |  |  |  | C(3)-S(1)-C(7)-C(18) | -0.19 |
|  |  |  |  |  |  |  | C(7)-S(1)-C(3)-C(2) | 0.19 |
|  |  |  |  |  |  |  | C(7)-S(1)-C(3)-N(28) | 176.74 |

| **3b** |  |  |  |  |  |  |  |
| --- | --- | --- | --- | --- | --- | --- | --- |
| **Atoms** | **Actual (°/A°)** | **Optimal (°/A°)** | **Atoms** | **Actual (°/A°)** | **Optimal (°/A°)** | **Atoms** | **Actual (°/A°)** |
| C(35)-H(57) | 1.09 | 1.11 | H(57)-C(35)-H(56) | 109.30 | 109 | C(22)-O(33)-C(34)-H(52) | -179.78 |
| C(35)-H(56) | 1.09 | 1.11 | H(57)-C(35)-H(55) | 107.53 | 109 | C(22)-O(33)-C(34)-H(53) | -61.10 |
| C(35)-H(55) | 1.09 | 1.11 | H(57)-C(35)-C(17) | 111.03 | 109.47 | C(22)-O(33)-C(34)-H(54) | 61.55 |
| C(34)-H(54) | 1.10 | 1.11 | H(56)-C(35)-H(55) | 109.30 | 109 | C(30)-C(31)-C(32)-C(27) | -0.80 |
| C(34)-H(53) | 1.09 | 1.11 | H(56)-C(35)-C(17) | 108.65 | 109.47 | C(30)-C(31)-C(32)-H(51) | 177.09 |
| C(34)-H(52) | 1.09 | 1.11 | H(55)-C(35)-C(17) | 111.01 | 109.47 | H(50)-C(31)-C(32)-C(27) | -179.91 |
| O(33)-C(34) | 1.42 | 1.40 | H(54)-C(34)-H(53) | 109.60 | 109 | H(50)-C(31)-C(32)-H(51) | -2.02 |
| C(32)-H(51) | 1.08 | 1.10 | H(54)-C(34)-H(52) | 109.34 | 109 | C(29)-C(30)-C(31)-C(32) | -0.13 |
| C(31)-H(50) | 1.08 | 1.10 | H(54)-C(34)-O(33) | 111.35 | 106.7 | C(29)-C(30)-C(31)-H(50) | 178.97 |
| C(31)-C(32) | 1.39 | 1.42 | H(53)-C(34)-H(52) | 109.36 | 109 | H(49)-C(30)-C(31)-C(32) | -179.81 |
| C(30)-H(49) | 1.08 | 1.10 | H(53)-C(34)-O(33) | 111.33 | 106.7 | H(49)-C(30)-C(31)-H(50) | -0.71 |
| C(30)-C(31) | 1.39 | 1.42 | H(52)-C(34)-O(33) | 105.76 | 106.7 | C(28)-C(29)-C(30)-C(31) | 0.75 |
| C(29)-H(48) | 1.08 | 1.10 | C(34)-O(33)-C(22) | 118.96 | 106.8 | C(28)-C(29)-C(30)-H(49) | -179.57 |
| C(29)-C(30) | 1.40 | 1.42 | H(51)-C(32)-C(31) | 119.34 | 120 | H(48)-C(29)-C(30)-C(31) | -179.80 |
| C(28)-H(47) | 1.09 | 1.10 | H(51)-C(32)-C(27) | 120.77 | 120 | H(48)-C(29)-C(30)-H(49) | -0.12 |
| C(28)-C(29) | 1.39 | 1.42 | C(31)-C(32)-C(27) | 119.85 |  | C(27)-C(28)-C(29)-C(30) | -0.43 |
| C(27)-C(32) | 1.40 | 1.42 | H(50)-C(31)-C(32) | 118.98 | 120 | C(27)-C(28)-C(29)-H(48) | -179.89 |
| C(27)-C(28) | 1.40 | 1.42 | H(50)-C(31)-C(30) | 120.03 | 120 | H(47)-C(28)-C(29)-C(30) | 178.95 |
| N(26)-H(46) | 1.02 | 1.05 | C(32)-C(31)-C(30) | 120.98 |  | H(47)-C(28)-C(29)-H(48) | -0.51 |
| N(26)-C(27) | 1.40 | 1.46 | H(49)-C(30)-C(31) | 120.44 | 120 | N(26)-C(27)-C(32)-C(31) | 179.37 |
| C(24)-H(45) | 1.08 | 1.11 | H(49)-C(30)-C(29) | 120.42 | 120 | N(26)-C(27)-C(32)-H(51) | 1.51 |
| C(23)-H(44) | 1.08 | 1.11 | C(31)-C(30)-C(29) | 119.14 |  | C(28)-C(27)-C(32)-C(31) | 1.10 |
| C(23)-C(24) | 1.39 | 1.52 | H(48)-C(29)-C(30) | 120.18 | 120 | C(28)-C(27)-C(32)-H(51) | -176.76 |
| C(22)-O(33) | 1.36 | 1.39 | H(48)-C(29)-C(28) | 119.36 | 120 | N(26)-C(27)-C(28)-C(29) | -178.87 |
| C(22)-C(23) | 1.40 | 1.51 | C(30)-C(29)-C(28) | 120.46 |  | N(26)-C(27)-C(28)-H(47) | 1.75 |
| C(21)-H(43) | 1.08 | 1.10 | H(47)-C(28)-C(29) | 120.12 | 120 | C(32)-C(27)-C(28)-C(29) | -0.49 |
| C(21)-C(22) | 1.40 | 1.50 | H(47)-C(28)-C(27) | 119.44 | 120 | C(32)-C(27)-C(28)-H(47) | -179.88 |
| C(20)-H(42) | 1.08 | 1.10 | C(29)-C(28)-C(27) | 120.44 |  | C(3)-N(26)-C(27)-C(28) | -158.58 |
| C(20)-C(21) | 1.38 | 1.42 | C(32)-C(27)-C(28) | 119.12 | 120 | C(3)-N(26)-C(27)-C(32) | 23.13 |
| O(19)-H(41) | 0.99 | 0.97 | C(32)-C(27)-N(26) | 123.47 | 120 | H(46)-N(26)-C(27)-C(28) | 19.56 |
| C(18)-O(19) | 1.33 | 1.36 | C(28)-C(27)-N(26) | 117.39 | 120 | H(46)-N(26)-C(27)-C(32) | -158.73 |
| C(17)-C(35) | 1.52 | 1.51 | H(46)-N(26)-C(27) | 116.91 | 118 | C(22)-C(23)-C(24)-C(6) | 0.01 |
| C(17)-O(25) | 1.22 | 1.21 | H(46)-N(26)-C(3) | 111.01 | 118 | C(22)-C(23)-C(24)-H(45) | 179.49 |
| C(16)-H(40) | 1.08 | 1.11 | C(27)-N(26)-C(3) | 132.06 | 124 | H(44)-C(23)-C(24)-C(6) | -179.73 |
| C(15)-H(39) | 1.08 | 1.11 | H(45)-C(24)-C(23) | 119.83 | 109.41 | H(44)-C(23)-C(24)-H(45) | -0.25 |
| C(15)-C(16) | 1.39 | 1.52 | H(45)-C(24)-C(6) | 119.43 | 109.41 | C(21)-C(22)-O(33)-C(34) | 179.91 |
| C(14)-C(17) | 1.49 | 1.51 | C(23)-C(24)-C(6) | 120.73 | 109.5 | C(23)-C(22)-O(33)-C(34) | -0.29 |
| C(14)-C(15) | 1.40 | 1.52 | H(44)-C(23)-C(24) | 119.32 | 109.41 | C(21)-C(22)-C(23)-C(24) | -0.21 |
| C(13)-H(38) | 1.08 | 1.10 | H(44)-C(23)-C(22) | 120.81 | 109.41 | C(21)-C(22)-C(23)-H(44) | 179.53 |
| C(13)-C(14) | 1.40 | 1.50 | C(24)-C(23)-C(22) | 119.87 | 109.5 | O(33)-C(22)-C(23)-C(24) | -179.99 |
| C(12)-H(37) | 1.09 | 1.10 | O(33)-C(22)-C(23) | 124.35 | 107.7 | O(33)-C(22)-C(23)-H(44) | -0.26 |
| C(12)-C(13) | 1.39 | 1.42 | O(33)-C(22)-C(21) | 115.90 |  | C(20)-C(21)-C(22)-C(23) | 0.07 |
| C(11)-C(16) | 1.40 | 1.52 | C(23)-C(22)-C(21) | 119.74 | 109.51 | C(20)-C(21)-C(22)-O(33) | 179.88 |
| C(11)-C(12) | 1.40 | 1.50 | H(43)-C(21)-C(22) | 118.65 | 118.2 | H(43)-C(21)-C(22)-C(23) | -179.80 |
| N(9)-H(36) | 1.01 | 1.02 | H(43)-C(21)-C(20) | 121.39 | 120 | H(43)-C(21)-C(22)-O(33) | 0.01 |
| N(9)-C(11) | 1.40 | 1.46 | C(22)-C(21)-C(20) | 119.96 | 122 | C(6)-C(20)-C(21)-C(22) | 0.26 |
| C(8)-O(10) | 1.24 | 1.21 | H(42)-C(20)-C(21) | 120.94 | 120 | C(6)-C(20)-C(21)-H(43) | -179.87 |
| C(8)-N(9) | 1.38 | 1.37 | H(42)-C(20)-C(6) | 118.16 | 118.2 | H(42)-C(20)-C(21)-C(22) | -179.83 |
| C(7)-C(18) | 1.39 | 1.42 | C(21)-C(20)-C(6) | 120.89 | 122 | H(42)-C(20)-C(21)-H(43) | 0.04 |
| C(7)-C(8) | 1.44 | 1.52 | H(41)-O(19)-C(18) | 105.77 | 108 | C(2)-C(18)-O(19)-H(41) | 179.22 |
| C(6)-C(24) | 1.40 | 1.52 | O(19)-C(18)-C(7) | 123.40 | 124.3 | C(7)-C(18)-O(19)-H(41) | -0.43 |
| C(6)-C(20) | 1.40 | 1.50 | O(19)-C(18)-C(2) | 123.33 | 124.3 | C(14)-C(17)-C(35)-H(55) | 59.79 |
| N(5)-C(6) | 1.41 | 1.47 | C(7)-C(18)-C(2) | 113.27 | 120 | C(14)-C(17)-C(35)-H(56) | -180.00 |
| N(4)-N(5) | 1.26 | 1.25 | C(35)-C(17)-O(25) | 120.31 | 122.5 | C(14)-C(17)-C(35)-H(57) | -59.77 |
| C(3)-N(26) | 1.36 | 1.46 | C(35)-C(17)-C(14) | 118.82 | 116.6 | O(25)-C(17)-C(35)-H(55) | -120.20 |
| C(2)-C(18) | 1.44 | 1.42 | O(25)-C(17)-C(14) | 120.87 | 122.5 | O(25)-C(17)-C(35)-H(56) | 0.02 |
| C(2)-N(4) | 1.39 | 1.46 | H(40)-C(16)-C(15) | 120.93 | 109.41 | O(25)-C(17)-C(35)-H(57) | 120.24 |
| C(2)-C(3) | 1.40 | 1.42 | H(40)-C(16)-C(11) | 119.66 | 109.41 | C(14)-C(15)-C(16)-C(11) | -0.02 |
| S(1)-C(7) | 1.77 | 1.66 | C(15)-C(16)-C(11) | 119.41 | 109.5 | C(14)-C(15)-C(16)-H(40) | -179.98 |
| S(1)-C(3) | 1.74 | 1.66 | H(39)-C(15)-C(16) | 119.86 | 109.41 | H(39)-C(15)-C(16)-C(11) | 179.98 |
|  |  |  | H(39)-C(15)-C(14) | 118.16 | 109.41 | H(39)-C(15)-C(16)-H(40) | 0.02 |
|  |  |  | C(16)-C(15)-C(14) | 121.99 | 109.5 | C(13)-C(14)-C(17)-O(25) | -179.96 |
|  |  |  | C(17)-C(14)-C(15) | 119.00 | 109.9 | C(13)-C(14)-C(17)-C(35) | 0.06 |
|  |  |  | C(17)-C(14)-C(13) | 122.96 | 110.51 | C(15)-C(14)-C(17)-O(25) | 0.06 |
|  |  |  | C(15)-C(14)-C(13) | 118.04 | 109.51 | C(15)-C(14)-C(17)-C(35) | -179.93 |
|  |  |  | H(38)-C(13)-C(14) | 120.65 | 118.2 | C(13)-C(14)-C(15)-C(16) | 0.01 |
|  |  |  | H(38)-C(13)-C(12) | 118.60 | 120 | C(13)-C(14)-C(15)-H(39) | -179.99 |
|  |  |  | C(14)-C(13)-C(12) | 120.75 | 122 | C(17)-C(14)-C(15)-C(16) | 180.00 |
|  |  |  | H(37)-C(12)-C(13) | 119.63 | 120 | C(17)-C(14)-C(15)-H(39) | 0.00 |
|  |  |  | H(37)-C(12)-C(11) | 119.67 | 118.2 | C(12)-C(13)-C(14)-C(15) | 0.00 |
|  |  |  | C(13)-C(12)-C(11) | 120.69 | 122 | C(12)-C(13)-C(14)-C(17) | -179.99 |
|  |  |  | C(16)-C(11)-C(12) | 119.12 | 109.51 | H(38)-C(13)-C(14)-C(15) | 179.98 |
|  |  |  | C(16)-C(11)-N(9) | 123.98 | 110.78 | H(38)-C(13)-C(14)-C(17) | 0.00 |
|  |  |  | C(12)-C(11)-N(9) | 116.90 |  | C(11)-C(12)-C(13)-C(14) | 0.01 |
|  |  |  | H(36)-N(9)-C(11) | 114.21 | 118 | C(11)-C(12)-C(13)-H(38) | -179.98 |
|  |  |  | H(36)-N(9)-C(8) | 116.23 | 117.4 | H(37)-C(12)-C(13)-C(14) | 179.98 |
|  |  |  | C(11)-N(9)-C(8) | 129.55 |  | H(37)-C(12)-C(13)-H(38) | -0.01 |
|  |  |  | O(10)-C(8)-N(9) | 122.20 | 122.6 | N(9)-C(11)-C(16)-C(15) | -179.99 |
|  |  |  | O(10)-C(8)-C(7) | 120.01 | 123 | N(9)-C(11)-C(16)-H(40) | -0.03 |
|  |  |  | N(9)-C(8)-C(7) | 117.79 | 112.74 | C(12)-C(11)-C(16)-C(15) | 0.02 |
|  |  |  | C(18)-C(7)-C(8) | 122.31 | 117.6 | C(12)-C(11)-C(16)-H(40) | 179.98 |
|  |  |  | C(18)-C(7)-S(1) | 111.25 | 119 | N(9)-C(11)-C(12)-C(13) | 179.99 |
|  |  |  | C(8)-C(7)-S(1) | 126.42 |  | N(9)-C(11)-C(12)-H(37) | 0.02 |
|  |  |  | C(24)-C(6)-C(20) | 118.80 | 109.51 | C(16)-C(11)-C(12)-C(13) | -0.01 |
|  |  |  | C(24)-C(6)-N(5) | 125.85 |  | C(16)-C(11)-C(12)-H(37) | -179.98 |
|  |  |  | C(20)-C(6)-N(5) | 115.35 |  | C(8)-N(9)-C(11)-C(12) | -179.85 |
|  |  |  | C(6)-N(5)-N(4) | 115.78 | 106.5 | C(8)-N(9)-C(11)-C(16) | 0.16 |
|  |  |  | N(5)-N(4)-C(2) | 117.30 | 107.5 | H(36)-N(9)-C(11)-C(12) | -0.05 |
|  |  |  | N(26)-C(3)-C(2) | 122.01 | 120 | H(36)-N(9)-C(11)-C(16) | 179.96 |
|  |  |  | N(26)-C(3)-S(1) | 125.28 |  | C(7)-C(8)-N(9)-C(11) | 179.90 |
|  |  |  | C(2)-C(3)-S(1) | 112.61 | 119 | C(7)-C(8)-N(9)-H(36) | 0.10 |
|  |  |  | C(18)-C(2)-N(4) | 131.79 | 120 | O(10)-C(8)-N(9)-C(11) | 0.00 |
|  |  |  | C(18)-C(2)-C(3) | 111.78 | 120 | O(10)-C(8)-N(9)-H(36) | -179.80 |
|  |  |  | N(4)-C(2)-C(3) | 116.41 | 120 | S(1)-C(7)-C(18)-C(2) | 0.13 |
|  |  |  | C(7)-S(1)-C(3) | 91.10 | 98.5 | S(1)-C(7)-C(18)-O(19) | 179.81 |
|  |  |  |  |  |  | C(8)-C(7)-C(18)-C(2) | -178.40 |
|  |  |  |  |  |  | C(8)-C(7)-C(18)-O(19) | 1.28 |
|  |  |  |  |  |  | S(1)-C(7)-C(8)-N(9) | 0.67 |
|  |  |  |  |  |  | S(1)-C(7)-C(8)-O(10) | -179.43 |
|  |  |  |  |  |  | C(18)-C(7)-C(8)-N(9) | 178.96 |
|  |  |  |  |  |  | C(18)-C(7)-C(8)-O(10) | -1.13 |
|  |  |  |  |  |  | N(5)-C(6)-C(24)-C(23) | 179.70 |
|  |  |  |  |  |  | N(5)-C(6)-C(24)-H(45) | 0.21 |
|  |  |  |  |  |  | C(20)-C(6)-C(24)-C(23) | 0.32 |
|  |  |  |  |  |  | C(20)-C(6)-C(24)-H(45) | -179.17 |
|  |  |  |  |  |  | N(5)-C(6)-C(20)-C(21) | -179.90 |
|  |  |  |  |  |  | N(5)-C(6)-C(20)-H(42) | 0.19 |
|  |  |  |  |  |  | C(24)-C(6)-C(20)-C(21) | -0.46 |
|  |  |  |  |  |  | C(24)-C(6)-C(20)-H(42) | 179.63 |
|  |  |  |  |  |  | N(4)-N(5)-C(6)-C(20) | -176.75 |
|  |  |  |  |  |  | N(4)-N(5)-C(6)-C(24) | 3.85 |
|  |  |  |  |  |  | C(2)-N(4)-N(5)-C(6) | -179.65 |
|  |  |  |  |  |  | S(1)-C(3)-N(26)-C(27) | 6.64 |
|  |  |  |  |  |  | S(1)-C(3)-N(26)-H(46) | -171.59 |
|  |  |  |  |  |  | C(2)-C(3)-N(26)-C(27) | -177.21 |
|  |  |  |  |  |  | C(2)-C(3)-N(26)-H(46) | 4.57 |
|  |  |  |  |  |  | C(3)-C(2)-C(18)-C(7) | 0.02 |
|  |  |  |  |  |  | C(3)-C(2)-C(18)-O(19) | -179.66 |
|  |  |  |  |  |  | N(4)-C(2)-C(18)-C(7) | -178.68 |
|  |  |  |  |  |  | N(4)-C(2)-C(18)-O(19) | 1.64 |
|  |  |  |  |  |  | C(3)-C(2)-N(4)-N(5) | -179.28 |
|  |  |  |  |  |  | C(18)-C(2)-N(4)-N(5) | -0.62 |
|  |  |  |  |  |  | N(4)-C(2)-C(3)-S(1) | 178.75 |
|  |  |  |  |  |  | N(4)-C(2)-C(3)-N(26) | 2.15 |
|  |  |  |  |  |  | C(18)-C(2)-C(3)-S(1) | -0.17 |
|  |  |  |  |  |  | C(18)-C(2)-C(3)-N(26) | -176.77 |
|  |  |  |  |  |  | C(3)-S(1)-C(7)-C(8) | 178.26 |
|  |  |  |  |  |  | C(3)-S(1)-C(7)-C(18) | -0.19 |
|  |  |  |  |  |  | C(7)-S(1)-C(3)-C(2) | 0.21 |
|  |  |  |  |  |  | C(7)-S(1)-C(3)-N(26) | 176.68 |

| **3c** |  |  |  |  |  |  |  |
| --- | --- | --- | --- | --- | --- | --- | --- |
| **Atoms** | **Actual (°/A°)** | **Optimal (°/A°)** | **Atoms** | **Actual (°/A°)** | **Optimal (°/A°)** | **Atoms** | **Actual (°/A°)** |
| C(34)-H(53) | 1.09 | 1.11 | H(53)-C(34)-H(52) | 109.30 | 109 | C(31)-C(32)-C(33)-C(28) | -0.84 |
| C(34)-H(52) | 1.09 | 1.11 | H(53)-C(34)-H(51) | 107.53 | 109 | C(31)-C(32)-C(33)-H(50) | 177.02 |
| C(34)-H(51) | 1.09 | 1.11 | H(53)-C(34)-C(17) | 111.02 | 109.47 | H(49)-C(32)-C(33)-C(28) | -179.92 |
| C(33)-H(50) | 1.08 | 1.10 | H(52)-C(34)-H(51) | 109.29 | 109 | H(49)-C(32)-C(33)-H(50) | -2.06 |
| C(32)-H(49) | 1.08 | 1.10 | H(52)-C(34)-C(17) | 108.65 | 109.47 | C(30)-C(31)-C(32)-C(33) | -0.14 |
| C(32)-C(33) | 1.39 | 1.42 | H(51)-C(34)-C(17) | 111.02 | 109.47 | C(30)-C(31)-C(32)-H(49) | 178.92 |
| C(31)-H(48) | 1.08 | 1.10 | H(50)-C(33)-C(32) | 119.39 | 120 | H(48)-C(31)-C(32)-C(33) | -179.77 |
| C(31)-C(32) | 1.39 | 1.42 | H(50)-C(33)-C(28) | 120.77 | 120 | H(48)-C(31)-C(32)-H(49) | -0.71 |
| C(30)-H(47) | 1.08 | 1.10 | C(32)-C(33)-C(28) | 119.81 |  | C(29)-C(30)-C(31)-C(32) | 0.78 |
| C(30)-C(31) | 1.40 | 1.42 | H(49)-C(32)-C(33) | 119.03 | 120 | C(29)-C(30)-C(31)-H(48) | -179.58 |
| C(29)-H(46) | 1.09 | 1.10 | H(49)-C(32)-C(31) | 120.04 | 120 | H(47)-C(30)-C(31)-C(32) | -179.75 |
| C(29)-C(30) | 1.39 | 1.42 | C(33)-C(32)-C(31) | 120.92 |  | H(47)-C(30)-C(31)-H(48) | -0.12 |
| C(28)-C(33) | 1.40 | 1.42 | H(48)-C(31)-C(32) | 120.40 | 120 | C(28)-C(29)-C(30)-C(31) | -0.44 |
| C(28)-C(29) | 1.40 | 1.42 | H(48)-C(31)-C(30) | 120.38 | 120 | C(28)-C(29)-C(30)-H(47) | -179.91 |
| N(27)-H(45) | 1.02 | 1.05 | C(32)-C(31)-C(30) | 119.21 |  | H(46)-C(29)-C(30)-C(31) | 178.95 |
| N(27)-C(28) | 1.41 | 1.46 | H(47)-C(30)-C(31) | 120.19 | 120 | H(46)-C(29)-C(30)-H(47) | -0.52 |
| C(24)-H(44) | 1.08 | 1.10 | H(47)-C(30)-C(29) | 119.37 | 120 | N(27)-C(28)-C(33)-C(32) | 179.37 |
| C(23)-H(43) | 1.08 | 1.10 | C(31)-C(30)-C(29) | 120.43 |  | N(27)-C(28)-C(33)-H(50) | 1.54 |
| C(23)-C(24) | 1.39 | 1.42 | H(46)-C(29)-C(30) | 120.14 | 120 | C(29)-C(28)-C(33)-C(32) | 1.18 |
| C(22)-Cl(25) | 1.76 | 1.72 | H(46)-C(29)-C(28) | 119.51 | 120 | C(29)-C(28)-C(33)-H(50) | -176.65 |
| C(22)-C(23) | 1.40 | 1.42 | C(30)-C(29)-C(28) | 120.35 |  | N(27)-C(28)-C(29)-C(30) | -178.84 |
| C(21)-H(42) | 1.08 | 1.10 | C(33)-C(28)-C(29) | 119.26 | 120 | N(27)-C(28)-C(29)-H(46) | 1.76 |
| C(21)-C(22) | 1.39 | 1.42 | C(33)-C(28)-N(27) | 123.33 | 120 | C(33)-C(28)-C(29)-C(30) | -0.55 |
| C(20)-H(41) | 1.08 | 1.10 | C(29)-C(28)-N(27) | 117.39 | 120 | C(33)-C(28)-C(29)-H(46) | -179.94 |
| C(20)-C(21) | 1.39 | 1.42 | H(45)-N(27)-C(28) | 116.83 | 118 | C(3)-N(27)-C(28)-C(29) | -156.68 |
| O(19)-H(40) | 0.99 | 0.97 | H(45)-N(27)-C(3) | 111.30 | 118 | C(3)-N(27)-C(28)-C(33) | 25.10 |
| C(18)-O(19) | 1.33 | 1.36 | C(28)-N(27)-C(3) | 131.83 | 124 | H(45)-N(27)-C(28)-C(29) | 20.70 |
| C(17)-C(34) | 1.52 | 1.51 | H(44)-C(24)-C(23) | 120.17 | 120 | H(45)-N(27)-C(28)-C(33) | -157.52 |
| C(17)-O(26) | 1.22 | 1.21 | H(44)-C(24)-C(6) | 119.56 | 120 | C(22)-C(23)-C(24)-C(6) | 0.05 |
| C(16)-H(39) | 1.08 | 1.10 | C(23)-C(24)-C(6) | 120.26 |  | C(22)-C(23)-C(24)-H(44) | 179.04 |
| C(15)-H(38) | 1.08 | 1.10 | H(43)-C(23)-C(24) | 120.72 | 120 | H(43)-C(23)-C(24)-C(6) | -179.45 |
| C(15)-C(16) | 1.39 | 1.42 | H(43)-C(23)-C(22) | 119.84 | 120 | H(43)-C(23)-C(24)-H(44) | -0.47 |
| C(14)-C(17) | 1.49 | 1.52 | C(24)-C(23)-C(22) | 119.44 |  | C(21)-C(22)-C(23)-C(24) | -0.58 |
| C(14)-C(15) | 1.40 | 1.42 | Cl(25)-C(22)-C(23) | 119.23 | 118.8 | C(21)-C(22)-C(23)-H(43) | 178.93 |
| C(13)-H(37) | 1.08 | 1.10 | Cl(25)-C(22)-C(21) | 119.54 | 118.8 | Cl(25)-C(22)-C(23)-C(24) | 179.95 |
| C(13)-C(14) | 1.40 | 1.42 | C(23)-C(22)-C(21) | 121.22 | 120 | Cl(25)-C(22)-C(23)-H(43) | -0.54 |
| C(12)-H(36) | 1.09 | 1.10 | H(42)-C(21)-C(22) | 120.19 | 120 | C(20)-C(21)-C(22)-C(23) | 0.13 |
| C(12)-C(13) | 1.39 | 1.42 | H(42)-C(21)-C(20) | 120.82 | 120 | C(20)-C(21)-C(22)-Cl(25) | 179.60 |
| C(11)-C(16) | 1.40 | 1.42 | C(22)-C(21)-C(20) | 118.99 |  | H(42)-C(21)-C(22)-C(23) | -179.51 |
| C(11)-C(12) | 1.40 | 1.42 | H(41)-C(20)-C(21) | 120.87 | 120 | H(42)-C(21)-C(22)-Cl(25) | -0.04 |
| N(9)-H(35) | 1.01 | 1.01 | H(41)-C(20)-C(6) | 118.39 | 120 | C(6)-C(20)-C(21)-C(22) | 0.84 |
| N(9)-C(11) | 1.41 | 1.35 | C(21)-C(20)-C(6) | 120.75 |  | C(6)-C(20)-C(21)-H(42) | -179.52 |
| C(8)-O(10) | 1.24 | 1.21 | H(40)-O(19)-C(18) | 105.74 | 108 | H(41)-C(20)-C(21)-C(22) | -179.47 |
| C(8)-N(9) | 1.38 | 1.37 | O(19)-C(18)-C(7) | 123.59 | 124.3 | H(41)-C(20)-C(21)-H(42) | 0.16 |
| C(7)-C(18) | 1.39 | 1.42 | O(19)-C(18)-C(2) | 123.20 | 124.3 | C(2)-C(18)-O(19)-H(40) | 178.73 |
| C(7)-C(8) | 1.45 | 1.52 | C(7)-C(18)-C(2) | 113.21 | 120 | C(7)-C(18)-O(19)-H(40) | -0.55 |
| C(6)-C(24) | 1.40 | 1.42 | C(34)-C(17)-O(26) | 120.35 | 122.5 | C(14)-C(17)-C(34)-H(51) | 59.87 |
| C(6)-C(20) | 1.40 | 1.42 | C(34)-C(17)-C(14) | 118.84 | 115 | C(14)-C(17)-C(34)-H(52) | -179.92 |
| N(5)-C(6) | 1.41 | 1.46 | O(26)-C(17)-C(14) | 120.81 | 123 | C(14)-C(17)-C(34)-H(53) | -59.69 |
| N(4)-N(5) | 1.26 | 1.25 | H(39)-C(16)-C(15) | 120.91 | 120 | O(26)-C(17)-C(34)-H(51) | -120.14 |
| C(3)-N(27) | 1.35 | 1.46 | H(39)-C(16)-C(11) | 119.70 | 120 | O(26)-C(17)-C(34)-H(52) | 0.07 |
| C(2)-C(18) | 1.44 | 1.42 | C(15)-C(16)-C(11) | 119.39 |  | O(26)-C(17)-C(34)-H(53) | 120.30 |
| C(2)-N(4) | 1.38 | 1.46 | H(38)-C(15)-C(16) | 119.87 | 120 | C(14)-C(15)-C(16)-C(11) | -0.02 |
| C(2)-C(3) | 1.40 | 1.42 | H(38)-C(15)-C(14) | 118.16 | 120 | C(14)-C(15)-C(16)-H(39) | -179.99 |
| S(1)-C(7) | 1.77 | 1.66 | C(16)-C(15)-C(14) | 121.98 |  | H(38)-C(15)-C(16)-C(11) | 180.00 |
| S(1)-C(3) | 1.74 | 1.66 | C(17)-C(14)-C(15) | 118.97 | 117.6 | H(38)-C(15)-C(16)-H(39) | 0.02 |
|  |  |  | C(17)-C(14)-C(13) | 122.97 | 117.6 | C(13)-C(14)-C(17)-O(26) | 179.95 |
|  |  |  | C(15)-C(14)-C(13) | 118.06 | 120 | C(13)-C(14)-C(17)-C(34) | -0.06 |
|  |  |  | H(37)-C(13)-C(14) | 120.66 | 120 | C(15)-C(14)-C(17)-O(26) | -0.04 |
|  |  |  | H(37)-C(13)-C(12) | 118.60 | 120 | C(15)-C(14)-C(17)-C(34) | 179.95 |
|  |  |  | C(14)-C(13)-C(12) | 120.74 |  | C(13)-C(14)-C(15)-C(16) | 0.03 |
|  |  |  | H(36)-C(12)-C(13) | 119.64 | 120 | C(13)-C(14)-C(15)-H(38) | -179.99 |
|  |  |  | H(36)-C(12)-C(11) | 119.70 | 120 | C(17)-C(14)-C(15)-C(16) | -179.98 |
|  |  |  | C(13)-C(12)-C(11) | 120.66 |  | C(17)-C(14)-C(15)-H(38) | 0.00 |
|  |  |  | C(16)-C(11)-C(12) | 119.17 | 120 | C(12)-C(13)-C(14)-C(15) | -0.02 |
|  |  |  | C(16)-C(11)-N(9) | 123.96 | 120 | C(12)-C(13)-C(14)-C(17) | 179.99 |
|  |  |  | C(12)-C(11)-N(9) | 116.87 | 120 | H(37)-C(13)-C(14)-C(15) | 179.97 |
|  |  |  | H(35)-N(9)-C(11) | 114.18 | 110 | H(37)-C(13)-C(14)-C(17) | -0.02 |
|  |  |  | H(35)-N(9)-C(8) | 116.29 | 117.4 | C(11)-C(12)-C(13)-C(14) | 0.00 |
|  |  |  | C(11)-N(9)-C(8) | 129.53 |  | C(11)-C(12)-C(13)-H(37) | -179.99 |
|  |  |  | O(10)-C(8)-N(9) | 122.32 | 122.6 | H(36)-C(12)-C(13)-C(14) | 179.98 |
|  |  |  | O(10)-C(8)-C(7) | 119.85 | 123 | H(36)-C(12)-C(13)-H(37) | -0.01 |
|  |  |  | N(9)-C(8)-C(7) | 117.83 | 112.74 | N(9)-C(11)-C(16)-C(15) | -179.99 |
|  |  |  | C(18)-C(7)-C(8) | 122.24 | 117.6 | N(9)-C(11)-C(16)-H(39) | -0.02 |
|  |  |  | C(18)-C(7)-S(1) | 111.29 | 119 | C(12)-C(11)-C(16)-C(15) | 0.00 |
|  |  |  | C(8)-C(7)-S(1) | 126.44 |  | C(12)-C(11)-C(16)-H(39) | 179.98 |
|  |  |  | C(24)-C(6)-C(20) | 119.33 | 120 | N(9)-C(11)-C(12)-C(13) | -180.00 |
|  |  |  | C(24)-C(6)-N(5) | 125.27 | 120 | N(9)-C(11)-C(12)-H(36) | 0.03 |
|  |  |  | C(20)-C(6)-N(5) | 115.38 | 120 | C(16)-C(11)-C(12)-C(13) | 0.01 |
|  |  |  | C(6)-N(5)-N(4) | 115.09 | 107.5 | C(16)-C(11)-C(12)-H(36) | -179.97 |
|  |  |  | N(5)-N(4)-C(2) | 117.64 | 107.5 | C(8)-N(9)-C(11)-C(12) | 179.85 |
|  |  |  | N(27)-C(3)-C(2) | 122.21 | 120 | C(8)-N(9)-C(11)-C(16) | -0.15 |
|  |  |  | N(27)-C(3)-S(1) | 125.22 |  | H(35)-N(9)-C(11)-C(12) | -0.15 |
|  |  |  | C(2)-C(3)-S(1) | 112.47 | 119 | H(35)-N(9)-C(11)-C(16) | 179.85 |
|  |  |  | C(18)-C(2)-N(4) | 131.73 | 120 | C(7)-C(8)-N(9)-C(11) | -179.83 |
|  |  |  | C(18)-C(2)-C(3) | 111.86 | 120 | C(7)-C(8)-N(9)-H(35) | 0.17 |
|  |  |  | N(4)-C(2)-C(3) | 116.40 | 120 | O(10)-C(8)-N(9)-C(11) | 0.23 |
|  |  |  | C(7)-S(1)-C(3) | 91.17 | 98.5 | O(10)-C(8)-N(9)-H(35) | -179.77 |
|  |  |  |  |  |  | S(1)-C(7)-C(18)-C(2) | 0.23 |
|  |  |  |  |  |  | S(1)-C(7)-C(18)-O(19) | 179.57 |
|  |  |  |  |  |  | C(8)-C(7)-C(18)-C(2) | -177.98 |
|  |  |  |  |  |  | C(8)-C(7)-C(18)-O(19) | 1.37 |
|  |  |  |  |  |  | S(1)-C(7)-C(8)-N(9) | 0.90 |
|  |  |  |  |  |  | S(1)-C(7)-C(8)-O(10) | -179.16 |
|  |  |  |  |  |  | C(18)-C(7)-C(8)-N(9) | 178.82 |
|  |  |  |  |  |  | C(18)-C(7)-C(8)-O(10) | -1.24 |
|  |  |  |  |  |  | N(5)-C(6)-C(24)-C(23) | 179.32 |
|  |  |  |  |  |  | N(5)-C(6)-C(24)-H(44) | 0.33 |
|  |  |  |  |  |  | C(20)-C(6)-C(24)-C(23) | 0.89 |
|  |  |  |  |  |  | C(20)-C(6)-C(24)-H(44) | -178.09 |
|  |  |  |  |  |  | N(5)-C(6)-C(20)-C(21) | -179.93 |
|  |  |  |  |  |  | N(5)-C(6)-C(20)-H(41) | 0.38 |
|  |  |  |  |  |  | C(24)-C(6)-C(20)-C(21) | -1.35 |
|  |  |  |  |  |  | C(24)-C(6)-C(20)-H(41) | 178.96 |
|  |  |  |  |  |  | N(4)-N(5)-C(6)-C(20) | -168.97 |
|  |  |  |  |  |  | N(4)-N(5)-C(6)-C(24) | 12.55 |
|  |  |  |  |  |  | C(2)-N(4)-N(5)-C(6) | -178.67 |
|  |  |  |  |  |  | S(1)-C(3)-N(27)-C(28) | 6.06 |
|  |  |  |  |  |  | S(1)-C(3)-N(27)-H(45) | -171.43 |
|  |  |  |  |  |  | C(2)-C(3)-N(27)-C(28) | -177.90 |
|  |  |  |  |  |  | C(2)-C(3)-N(27)-H(45) | 4.61 |
|  |  |  |  |  |  | C(3)-C(2)-C(18)-C(7) | -0.09 |
|  |  |  |  |  |  | C(3)-C(2)-C(18)-O(19) | -179.44 |
|  |  |  |  |  |  | N(4)-C(2)-C(18)-C(7) | -179.44 |
|  |  |  |  |  |  | N(4)-C(2)-C(18)-O(19) | 1.21 |
|  |  |  |  |  |  | C(3)-C(2)-N(4)-N(5) | -176.65 |
|  |  |  |  |  |  | C(18)-C(2)-N(4)-N(5) | 2.68 |
|  |  |  |  |  |  | N(4)-C(2)-C(3)-S(1) | 179.37 |
|  |  |  |  |  |  | N(4)-C(2)-C(3)-N(27) | 2.87 |
|  |  |  |  |  |  | C(18)-C(2)-C(3)-S(1) | -0.09 |
|  |  |  |  |  |  | C(18)-C(2)-C(3)-N(27) | -176.59 |
|  |  |  |  |  |  | C(3)-S(1)-C(7)-C(8) | 177.88 |
|  |  |  |  |  |  | C(3)-S(1)-C(7)-C(18) | -0.23 |
|  |  |  |  |  |  | C(7)-S(1)-C(3)-C(2) | 0.18 |
|  |  |  |  |  |  | C(7)-S(1)-C(3)-N(27) | 176.56 |

**Table S2** Bond length, bond angle, dihedral angle for compounds **5a-c**

| **5a** |  |  |  |  |  |  |  |
| --- | --- | --- | --- | --- | --- | --- | --- |
| **Atoms** | **Actual (°/A°)** | **Optimal (°/A°)** | **Atoms** | **Actual (°/A°)** | **Optimal (°/A°)** | **Atoms** | **Actual (°/A°)** |
| C(34)-H(58) | 1.08 | 1.10 | H(58)-C(34)-C(33) | 119.14 | 120 | C(32)-C(33)-C(34)-C(29) | -0.69 |
| C(33)-H(57) | 1.08 | 1.10 | H(58)-C(34)-C(29) | 120.96 | 120 | C(32)-C(33)-C(34)-H(58) | 177.21 |
| C(33)-C(34) | 1.39 | 1.42 | C(33)-C(34)-C(29) | 119.87 |  | H(57)-C(33)-C(34)-C(29) | -179.89 |
| C(32)-H(56) | 1.08 | 1.10 | H(57)-C(33)-C(34) | 118.93 | 120 | H(57)-C(33)-C(34)-H(58) | -1.98 |
| C(32)-C(33) | 1.39 | 1.42 | H(57)-C(33)-C(32) | 120.04 | 120 | C(31)-C(32)-C(33)-C(34) | -0.16 |
| C(31)-H(55) | 1.08 | 1.10 | C(34)-C(33)-C(32) | 121.03 |  | C(31)-C(32)-C(33)-H(57) | 179.03 |
| C(31)-C(32) | 1.40 | 1.42 | H(56)-C(32)-C(33) | 120.46 | 120 | H(56)-C(32)-C(33)-C(34) | -179.82 |
| C(30)-H(54) | 1.09 | 1.10 | H(56)-C(32)-C(31) | 120.44 | 120 | H(56)-C(32)-C(33)-H(57) | -0.63 |
| C(30)-C(31) | 1.39 | 1.42 | C(33)-C(32)-C(31) | 119.11 |  | C(30)-C(31)-C(32)-C(33) | 0.67 |
| C(29)-C(34) | 1.40 | 1.42 | H(55)-C(31)-C(32) | 120.19 | 120 | C(30)-C(31)-C(32)-H(56) | -179.67 |
| C(29)-C(30) | 1.40 | 1.42 | H(55)-C(31)-C(30) | 119.35 | 120 | H(55)-C(31)-C(32)-C(33) | -179.76 |
| N(28)-H(53) | 1.02 | 1.05 | C(32)-C(31)-C(30) | 120.46 |  | H(55)-C(31)-C(32)-H(56) | -0.09 |
| N(28)-C(29) | 1.40 | 1.46 | H(54)-C(30)-C(31) | 120.07 | 120 | C(29)-C(30)-C(31)-C(32) | -0.33 |
| C(27)-H(52) | 1.09 | 1.11 | H(54)-C(30)-C(29) | 119.42 | 120 | C(29)-C(30)-C(31)-H(55) | -179.91 |
| C(27)-H(51) | 1.09 | 1.11 | C(31)-C(30)-C(29) | 120.51 |  | H(54)-C(30)-C(31)-C(32) | 179.10 |
| C(27)-H(50) | 1.09 | 1.11 | C(34)-C(29)-C(30) | 119.02 | 120 | H(54)-C(30)-C(31)-H(55) | -0.48 |
| C(26)-H(49) | 1.09 | 1.11 | C(34)-C(29)-N(28) | 123.76 | 120 | N(28)-C(29)-C(34)-C(33) | 179.49 |
| C(26)-H(48) | 1.09 | 1.11 | C(30)-C(29)-N(28) | 117.20 | 120 | N(28)-C(29)-C(34)-H(58) | 1.62 |
| C(26)-H(47) | 1.09 | 1.11 | H(53)-N(28)-C(29) | 116.72 | 118 | C(30)-C(29)-C(34)-C(33) | 1.02 |
| C(25)-H(46) | 1.10 | 1.11 | H(53)-N(28)-C(3) | 110.82 |  | C(30)-C(29)-C(34)-H(58) | -176.85 |
| C(25)-H(45) | 1.09 | 1.11 | C(29)-N(28)-C(3) | 132.40 |  | N(28)-C(29)-C(30)-C(31) | -179.09 |
| C(25)-H(44) | 1.09 | 1.11 | H(52)-C(27)-H(51) | 108.90 | 109 | N(28)-C(29)-C(30)-H(54) | 1.47 |
| C(23)-H(43) | 1.08 | 1.10 | H(52)-C(27)-H(50) | 110.65 | 109 | C(34)-C(29)-C(30)-C(31) | -0.52 |
| C(22)-H(42) | 1.09 | 1.10 | H(52)-C(27)-C(18) | 110.92 | 110 | C(34)-C(29)-C(30)-H(54) | -179.95 |
| C(22)-C(23) | 1.39 | 1.42 | H(51)-C(27)-H(50) | 105.99 | 109 | C(3)-N(28)-C(29)-C(30) | -160.50 |
| C(21)-C(25) | 1.51 | 1.50 | H(51)-C(27)-C(18) | 110.07 | 110 | C(3)-N(28)-C(29)-C(34) | 21.00 |
| C(21)-C(22) | 1.40 | 1.42 | H(50)-C(27)-C(18) | 110.19 | 110 | H(53)-N(28)-C(29)-C(30) | 16.30 |
| C(20)-H(41) | 1.09 | 1.10 | H(49)-C(26)-H(48) | 109.29 | 109 | H(53)-N(28)-C(29)-C(34) | -162.20 |
| C(20)-C(21) | 1.40 | 1.42 | H(49)-C(26)-H(47) | 107.53 | 109 | C(21)-C(22)-C(23)-C(6) | 0.00 |
| C(19)-H(40) | 1.08 | 1.10 | H(49)-C(26)-C(17) | 111.01 | 109.47 | C(21)-C(22)-C(23)-H(43) | 179.00 |
| C(19)-C(20) | 1.39 | 1.42 | H(48)-C(26)-H(47) | 109.31 | 109 | H(42)-C(22)-C(23)-C(6) | -179.52 |
| C(18)-C(27) | 1.50 | 1.50 | H(48)-C(26)-C(17) | 108.65 | 109.47 | H(42)-C(22)-C(23)-H(43) | -0.53 |
| C(17)-C(26) | 1.52 | 1.51 | H(47)-C(26)-C(17) | 111.02 | 109.47 | C(20)-C(21)-C(25)-H(44) | 10.83 |
| C(17)-O(24) | 1.22 | 1.21 | H(46)-C(25)-H(45) | 107.17 | 109 | C(20)-C(21)-C(25)-H(45) | 131.50 |
| C(16)-H(39) | 1.08 | 1.11 | H(46)-C(25)-H(44) | 107.66 | 109 | C(20)-C(21)-C(25)-H(46) | -109.18 |
| C(15)-H(38) | 1.08 | 1.11 | H(46)-C(25)-C(21) | 111.04 | 110 | C(22)-C(21)-C(25)-H(44) | -170.42 |
| C(15)-C(16) | 1.39 | 1.52 | H(45)-C(25)-H(44) | 108.04 | 109 | C(22)-C(21)-C(25)-H(45) | -49.74 |
| C(14)-C(17) | 1.49 | 1.51 | H(45)-C(25)-C(21) | 111.32 | 110 | C(22)-C(21)-C(25)-H(46) | 69.58 |
| C(14)-C(15) | 1.40 | 1.52 | H(44)-C(25)-C(21) | 111.42 | 110 | C(20)-C(21)-C(22)-C(23) | -0.34 |
| C(13)-H(37) | 1.08 | 1.10 | H(43)-C(23)-C(22) | 120.55 | 120 | C(20)-C(21)-C(22)-H(42) | 179.19 |
| C(13)-C(14) | 1.40 | 1.50 | H(43)-C(23)-C(6) | 119.44 | 120 | C(25)-C(21)-C(22)-C(23) | -179.13 |
| C(12)-H(36) | 1.09 | 1.10 | C(22)-C(23)-C(6) | 120.00 |  | C(25)-C(21)-C(22)-H(42) | 0.39 |
| C(12)-C(13) | 1.39 | 1.42 | H(42)-C(22)-C(23) | 119.30 | 120 | C(19)-C(20)-C(21)-C(22) | -0.13 |
| C(11)-C(16) | 1.41 | 1.52 | H(42)-C(22)-C(21) | 119.20 | 120 | C(19)-C(20)-C(21)-C(25) | 178.66 |
| C(11)-C(12) | 1.40 | 1.50 | C(23)-C(22)-C(21) | 121.50 |  | H(41)-C(20)-C(21)-C(22) | -179.82 |
| N(9)-H(35) | 1.01 | 1.02 | C(25)-C(21)-C(22) | 120.60 | 121.4 | H(41)-C(20)-C(21)-C(25) | -1.03 |
| N(9)-C(11) | 1.40 | 1.46 | C(25)-C(21)-C(20) | 121.41 | 121.4 | C(6)-C(19)-C(20)-C(21) | 0.93 |
| C(8)-O(10) | 1.22 | 1.21 | C(22)-C(21)-C(20) | 117.98 | 120 | C(6)-C(19)-C(20)-H(41) | -179.38 |
| C(8)-N(9) | 1.39 | 1.37 | H(41)-C(20)-C(21) | 119.51 | 120 | H(40)-C(19)-C(20)-C(21) | -179.56 |
| C(7)-C(18) | 1.37 | 1.42 | H(41)-C(20)-C(19) | 119.44 | 120 | H(40)-C(19)-C(20)-H(41) | 0.13 |
| C(7)-C(8) | 1.48 | 1.52 | C(21)-C(20)-C(19) | 121.05 |  | C(2)-C(18)-C(27)-H(50) | -55.28 |
| C(6)-C(23) | 1.40 | 1.42 | H(40)-C(19)-C(20) | 120.98 | 120 | C(2)-C(18)-C(27)-H(51) | 61.26 |
| C(6)-C(19) | 1.40 | 1.42 | H(40)-C(19)-C(6) | 118.51 | 120 | C(2)-C(18)-C(27)-H(52) | -178.16 |
| N(5)-C(6) | 1.41 | 1.46 | C(20)-C(19)-C(6) | 120.50 |  | C(7)-C(18)-C(27)-H(50) | 123.32 |
| N(4)-N(5) | 1.26 | 1.25 | C(27)-C(18)-C(7) | 124.36 | 121.4 | C(7)-C(18)-C(27)-H(51) | -120.14 |
| C(3)-N(28) | 1.36 | 1.47 | C(27)-C(18)-C(2) | 123.82 | 117.2 | C(7)-C(18)-C(27)-H(52) | 0.44 |
| C(2)-C(18) | 1.44 | 1.50 | C(7)-C(18)-C(2) | 111.81 | 121.4 | C(14)-C(17)-C(26)-H(47) | 59.58 |
| C(2)-N(4) | 1.38 | 1.47 | C(26)-C(17)-O(24) | 120.28 | 122.5 | C(14)-C(17)-C(26)-H(48) | 179.81 |
| C(2)-C(3) | 1.41 | 1.52 | C(26)-C(17)-C(14) | 118.83 | 116.6 | C(14)-C(17)-C(26)-H(49) | -59.98 |
| S(1)-C(7) | 1.78 | 1.66 | O(24)-C(17)-C(14) | 120.90 | 122.5 | O(24)-C(17)-C(26)-H(47) | -120.42 |
| S(1)-C(3) | 1.73 |  | H(39)-C(16)-C(15) | 120.88 | 109.41 | O(24)-C(17)-C(26)-H(48) | -0.18 |
|  |  |  | H(39)-C(16)-C(11) | 119.68 | 109.41 | O(24)-C(17)-C(26)-H(49) | 120.03 |
|  |  |  | C(15)-C(16)-C(11) | 119.45 | 109.5 | C(14)-C(15)-C(16)-C(11) | 0.19 |
|  |  |  | H(38)-C(15)-C(16) | 119.89 | 109.41 | C(14)-C(15)-C(16)-H(39) | -179.24 |
|  |  |  | H(38)-C(15)-C(14) | 118.15 | 109.41 | H(38)-C(15)-C(16)-C(11) | -179.98 |
|  |  |  | C(16)-C(15)-C(14) | 121.96 | 109.5 | H(38)-C(15)-C(16)-H(39) | 0.60 |
|  |  |  | C(17)-C(14)-C(15) | 118.99 | 109.9 | C(13)-C(14)-C(17)-O(24) | -179.81 |
|  |  |  | C(17)-C(14)-C(13) | 122.95 | 110.51 | C(13)-C(14)-C(17)-C(26) | 0.20 |
|  |  |  | C(15)-C(14)-C(13) | 118.06 | 109.51 | C(15)-C(14)-C(17)-O(24) | 0.27 |
|  |  |  | H(37)-C(13)-C(14) | 120.64 | 118.2 | C(15)-C(14)-C(17)-C(26) | -179.72 |
|  |  |  | H(37)-C(13)-C(12) | 118.61 | 120 | C(13)-C(14)-C(15)-C(16) | -0.08 |
|  |  |  | C(14)-C(13)-C(12) | 120.75 | 122 | C(13)-C(14)-C(15)-H(38) | -179.92 |
|  |  |  | H(36)-C(12)-C(13) | 119.66 | 120 | C(17)-C(14)-C(15)-C(16) | 179.84 |
|  |  |  | H(36)-C(12)-C(11) | 119.65 | 118.2 | C(17)-C(14)-C(15)-H(38) | 0.00 |
|  |  |  | C(13)-C(12)-C(11) | 120.69 | 122 | C(12)-C(13)-C(14)-C(15) | -0.06 |
|  |  |  | C(16)-C(11)-C(12) | 119.10 | 109.51 | C(12)-C(13)-C(14)-C(17) | -179.97 |
|  |  |  | C(16)-C(11)-N(9) | 123.84 | 110.78 | H(37)-C(13)-C(14)-C(15) | 179.97 |
|  |  |  | C(12)-C(11)-N(9) | 117.06 |  | H(37)-C(13)-C(14)-C(17) | 0.06 |
|  |  |  | H(35)-N(9)-C(11) | 114.56 | 118 | C(11)-C(12)-C(13)-C(14) | 0.09 |
|  |  |  | H(35)-N(9)-C(8) | 116.15 | 117.4 | C(11)-C(12)-C(13)-H(37) | -179.94 |
|  |  |  | C(11)-N(9)-C(8) | 128.95 |  | H(36)-C(12)-C(13)-C(14) | -179.84 |
|  |  |  | O(10)-C(8)-N(9) | 122.79 | 122.6 | H(36)-C(12)-C(13)-H(37) | 0.13 |
|  |  |  | O(10)-C(8)-C(7) | 122.97 | 123 | N(9)-C(11)-C(16)-C(15) | 179.13 |
|  |  |  | N(9)-C(8)-C(7) | 114.23 | 112.74 | N(9)-C(11)-C(16)-H(39) | -1.44 |
|  |  |  | C(18)-C(7)-C(8) | 128.31 | 117.6 | C(12)-C(11)-C(16)-C(15) | -0.15 |
|  |  |  | C(18)-C(7)-S(1) | 112.15 | 119 | C(12)-C(11)-C(16)-H(39) | 179.28 |
|  |  |  | C(8)-C(7)-S(1) | 119.53 |  | N(9)-C(11)-C(12)-C(13) | -179.31 |
|  |  |  | C(23)-C(6)-C(19) | 118.94 | 120 | N(9)-C(11)-C(12)-H(36) | 0.62 |
|  |  |  | C(23)-C(6)-N(5) | 125.19 | 120 | C(16)-C(11)-C(12)-C(13) | 0.02 |
|  |  |  | C(19)-C(6)-N(5) | 115.85 | 120 | C(16)-C(11)-C(12)-H(36) | 179.95 |
|  |  |  | C(6)-N(5)-N(4) | 115.25 | 107.5 | C(8)-N(9)-C(11)-C(12) | -174.73 |
|  |  |  | N(5)-N(4)-C(2) | 118.42 | 106.5 | C(8)-N(9)-C(11)-C(16) | 5.98 |
|  |  |  | N(28)-C(3)-C(2) | 122.54 |  | H(35)-N(9)-C(11)-C(12) | -1.74 |
|  |  |  | N(28)-C(3)-S(1) | 126.08 |  | H(35)-N(9)-C(11)-C(16) | 178.97 |
|  |  |  | C(2)-C(3)-S(1) | 111.36 |  | C(7)-C(8)-N(9)-C(11) | -175.41 |
|  |  |  | C(18)-C(2)-N(4) | 131.73 |  | C(7)-C(8)-N(9)-H(35) | 11.70 |
|  |  |  | C(18)-C(2)-C(3) | 113.35 | 109.51 | O(10)-C(8)-N(9)-C(11) | 4.59 |
|  |  |  | N(4)-C(2)-C(3) | 114.93 |  | O(10)-C(8)-N(9)-H(35) | -168.30 |
|  |  |  | C(7)-S(1)-C(3) | 91.33 |  | S(1)-C(7)-C(18)-C(2) | -0.60 |
|  |  |  |  |  |  | S(1)-C(7)-C(18)-C(27) | -179.35 |
|  |  |  |  |  |  | C(8)-C(7)-C(18)-C(2) | -179.55 |
|  |  |  |  |  |  | C(8)-C(7)-C(18)-C(27) | 1.70 |
|  |  |  |  |  |  | S(1)-C(7)-C(8)-N(9) | 27.69 |
|  |  |  |  |  |  | S(1)-C(7)-C(8)-O(10) | -152.31 |
|  |  |  |  |  |  | C(18)-C(7)-C(8)-N(9) | -153.43 |
|  |  |  |  |  |  | C(18)-C(7)-C(8)-O(10) | 26.57 |
|  |  |  |  |  |  | N(5)-C(6)-C(23)-C(22) | 179.35 |
|  |  |  |  |  |  | N(5)-C(6)-C(23)-H(43) | 0.35 |
|  |  |  |  |  |  | C(19)-C(6)-C(23)-C(22) | 0.79 |
|  |  |  |  |  |  | C(19)-C(6)-C(23)-H(43) | -178.22 |
|  |  |  |  |  |  | N(5)-C(6)-C(19)-C(20) | -179.94 |
|  |  |  |  |  |  | N(5)-C(6)-C(19)-H(40) | 0.53 |
|  |  |  |  |  |  | C(23)-C(6)-C(19)-C(20) | -1.25 |
|  |  |  |  |  |  | C(23)-C(6)-C(19)-H(40) | 179.23 |
|  |  |  |  |  |  | N(4)-N(5)-C(6)-C(19) | -168.25 |
|  |  |  |  |  |  | N(4)-N(5)-C(6)-C(23) | 13.14 |
|  |  |  |  |  |  | C(2)-N(4)-N(5)-C(6) | -178.30 |
|  |  |  |  |  |  | S(1)-C(3)-N(28)-C(29) | 2.21 |
|  |  |  |  |  |  | S(1)-C(3)-N(28)-H(53) | -174.74 |
|  |  |  |  |  |  | C(2)-C(3)-N(28)-C(29) | -179.23 |
|  |  |  |  |  |  | C(2)-C(3)-N(28)-H(53) | 3.82 |
|  |  |  |  |  |  | C(3)-C(2)-C(18)-C(7) | 0.00 |
|  |  |  |  |  |  | C(3)-C(2)-C(18)-C(27) | 178.75 |
|  |  |  |  |  |  | N(4)-C(2)-C(18)-C(7) | -179.89 |
|  |  |  |  |  |  | N(4)-C(2)-C(18)-C(27) | -1.14 |
|  |  |  |  |  |  | C(3)-C(2)-N(4)-N(5) | -177.13 |
|  |  |  |  |  |  | C(18)-C(2)-N(4)-N(5) | 2.76 |
|  |  |  |  |  |  | N(4)-C(2)-C(3)-S(1) | -179.48 |
|  |  |  |  |  |  | N(4)-C(2)-C(3)-N(28) | 1.77 |
|  |  |  |  |  |  | C(18)-C(2)-C(3)-S(1) | 0.62 |
|  |  |  |  |  |  | C(18)-C(2)-C(3)-N(28) | -178.14 |
|  |  |  |  |  |  | C(3)-S(1)-C(7)-C(8) | 179.87 |
|  |  |  |  |  |  | C(3)-S(1)-C(7)-C(18) | 0.81 |
|  |  |  |  |  |  | C(7)-S(1)-C(3)-C(2) | -0.80 |
|  |  |  |  |  |  | C(7)-S(1)-C(3)-N(28) | 177.90 |

| **5b** |  |  |  |  |  |  |  |
| --- | --- | --- | --- | --- | --- | --- | --- |
| **Atoms** | **Actual (°/A°)** | **Optimal (°/A°)** | **Atoms** | **Actual (°/A°)** | **Optimal (°/A°)** | **Atoms** | **Actual (°/A°)** |
| C(35)-H(59) | 1.09 | 1.11 | S(1)-C(3) | 1.73 |  | C(32)-C(33)-C(34)-C(29) | -0.69 |
| C(35)-H(58) | 1.09 | 1.11 | H(59)-C(35)-H(58) | 110.63 | 109 | C(32)-C(33)-C(34)-H(56) | 177.23 |
| C(35)-H(57) | 1.09 | 1.11 | H(59)-C(35)-H(57) | 106.02 | 109 | H(55)-C(33)-C(34)-C(29) | -179.88 |
| C(34)-H(56) | 1.08 | 1.10 | H(59)-C(35)-C(18) | 110.19 | 110 | H(55)-C(33)-C(34)-H(56) | -1.96 |
| C(33)-H(55) | 1.08 | 1.10 | H(58)-C(35)-H(57) | 108.89 | 109 | C(31)-C(32)-C(33)-C(34) | -0.15 |
| C(33)-C(34) | 1.39 | 1.42 | H(58)-C(35)-C(18) | 110.91 | 110 | C(31)-C(32)-C(33)-H(55) | 179.03 |
| C(32)-H(54) | 1.08 | 1.10 | H(57)-C(35)-C(18) | 110.08 | 110 | H(54)-C(32)-C(33)-C(34) | -179.83 |
| C(32)-C(33) | 1.39 | 1.42 | H(56)-C(34)-C(33) | 119.14 | 120 | H(54)-C(32)-C(33)-H(55) | -0.64 |
| C(31)-H(53) | 1.08 | 1.10 | H(56)-C(34)-C(29) | 120.95 | 120 | C(30)-C(31)-C(32)-C(33) | 0.67 |
| C(31)-C(32) | 1.40 | 1.42 | C(33)-C(34)-C(29) | 119.88 |  | C(30)-C(31)-C(32)-H(54) | -179.66 |
| C(30)-H(52) | 1.09 | 1.10 | H(55)-C(33)-C(34) | 118.92 | 120 | H(53)-C(31)-C(32)-C(33) | -179.79 |
| C(30)-C(31) | 1.39 | 1.42 | H(55)-C(33)-C(32) | 120.03 | 120 | H(53)-C(31)-C(32)-H(54) | -0.11 |
| C(29)-C(34) | 1.40 | 1.42 | C(34)-C(33)-C(32) | 121.05 |  | C(29)-C(30)-C(31)-C(32) | -0.34 |
| C(29)-C(30) | 1.41 | 1.42 | H(54)-C(32)-C(33) | 120.46 | 120 | C(29)-C(30)-C(31)-H(53) | -179.89 |
| N(28)-H(51) | 1.02 | 1.05 | H(54)-C(32)-C(31) | 120.45 | 120 | H(52)-C(30)-C(31)-C(32) | 179.05 |
| N(28)-C(29) | 1.40 | 1.46 | C(33)-C(32)-C(31) | 119.08 |  | H(52)-C(30)-C(31)-H(53) | -0.50 |
| C(27)-H(50) | 1.10 | 1.11 | H(53)-C(31)-C(32) | 120.18 | 120 | N(28)-C(29)-C(34)-C(33) | 179.50 |
| C(27)-H(49) | 1.10 | 1.11 | H(53)-C(31)-C(30) | 119.35 | 120 | N(28)-C(29)-C(34)-H(56) | 1.62 |
| C(27)-H(48) | 1.09 | 1.11 | C(32)-C(31)-C(30) | 120.47 |  | C(30)-C(29)-C(34)-C(33) | 1.00 |
| O(26)-C(27) | 1.42 | 1.40 | H(52)-C(30)-C(31) | 120.07 | 120 | C(30)-C(29)-C(34)-H(56) | -176.88 |
| C(25)-H(47) | 1.09 | 1.11 | H(52)-C(30)-C(29) | 119.40 | 120 | N(28)-C(29)-C(30)-C(31) | -179.09 |
| C(25)-H(46) | 1.09 | 1.11 | C(31)-C(30)-C(29) | 120.53 |  | N(28)-C(29)-C(30)-H(52) | 1.50 |
| C(25)-H(45) | 1.09 | 1.11 | C(34)-C(29)-C(30) | 118.98 | 120 | C(34)-C(29)-C(30)-C(31) | -0.50 |
| C(23)-H(44) | 1.08 | 1.11 | C(34)-C(29)-N(28) | 123.77 | 120 | C(34)-C(29)-C(30)-H(52) | -179.90 |
| C(22)-H(43) | 1.08 | 1.11 | C(30)-C(29)-N(28) | 117.23 | 120 | C(3)-N(28)-C(29)-C(30) | -160.69 |
| C(22)-C(23) | 1.39 | 1.52 | H(51)-N(28)-C(29) | 116.75 | 118 | C(3)-N(28)-C(29)-C(34) | 20.78 |
| C(21)-O(26) | 1.36 | 1.39 | H(51)-N(28)-C(3) | 110.79 |  | H(51)-N(28)-C(29)-C(30) | 16.21 |
| C(21)-C(22) | 1.40 | 1.51 | C(29)-N(28)-C(3) | 132.40 |  | H(51)-N(28)-C(29)-C(34) | -162.32 |
| C(20)-H(42) | 1.08 | 1.10 | H(50)-C(27)-H(49) | 109.60 | 109 | C(21)-O(26)-C(27)-H(48) | -179.70 |
| C(20)-C(21) | 1.40 | 1.50 | H(50)-C(27)-H(48) | 109.35 | 109 | C(21)-O(26)-C(27)-H(49) | -61.01 |
| C(19)-H(41) | 1.08 | 1.10 | H(50)-C(27)-O(26) | 111.35 | 106.7 | C(21)-O(26)-C(27)-H(50) | 61.62 |
| C(19)-C(20) | 1.38 | 1.42 | H(49)-C(27)-H(48) | 109.36 | 109 | C(21)-C(22)-C(23)-C(6) | 0.07 |
| C(18)-C(35) | 1.50 | 1.50 | H(49)-C(27)-O(26) | 111.33 | 106.7 | C(21)-C(22)-C(23)-H(44) | 179.05 |
| C(17)-C(25) | 1.52 | 1.51 | H(48)-C(27)-O(26) | 105.77 | 106.7 | H(43)-C(22)-C(23)-C(6) | -179.35 |
| C(17)-O(24) | 1.22 | 1.21 | C(27)-O(26)-C(21) | 118.93 | 106.8 | H(43)-C(22)-C(23)-H(44) | -0.36 |
| C(16)-H(40) | 1.08 | 1.11 | H(47)-C(25)-H(46) | 109.28 | 109 | C(20)-C(21)-O(26)-C(27) | -179.68 |
| C(15)-H(39) | 1.08 | 1.11 | H(47)-C(25)-H(45) | 107.53 | 109 | C(22)-C(21)-O(26)-C(27) | -0.21 |
| C(15)-C(16) | 1.39 | 1.52 | H(47)-C(25)-C(17) | 111.00 | 109.47 | C(20)-C(21)-C(22)-C(23) | -0.55 |
| C(14)-C(17) | 1.49 | 1.51 | H(46)-C(25)-H(45) | 109.32 | 109 | C(20)-C(21)-C(22)-H(43) | 178.86 |
| C(14)-C(15) | 1.40 | 1.52 | H(46)-C(25)-C(17) | 108.65 | 109.47 | O(26)-C(21)-C(22)-C(23) | 179.99 |
| C(13)-H(38) | 1.08 | 1.10 | H(45)-C(25)-C(17) | 111.04 | 109.47 | O(26)-C(21)-C(22)-H(43) | -0.60 |
| C(13)-C(14) | 1.40 | 1.50 | H(44)-C(23)-C(22) | 119.85 | 109.41 | C(19)-C(20)-C(21)-C(22) | 0.13 |
| C(12)-H(37) | 1.09 | 1.10 | H(44)-C(23)-C(6) | 119.36 | 109.41 | C(19)-C(20)-C(21)-O(26) | 179.63 |
| C(12)-C(13) | 1.39 | 1.42 | C(22)-C(23)-C(6) | 120.78 | 109.5 | H(42)-C(20)-C(21)-C(22) | -179.53 |
| C(11)-C(16) | 1.41 | 1.52 | H(43)-C(22)-C(23) | 119.29 | 109.41 | H(42)-C(20)-C(21)-O(26) | -0.02 |
| C(11)-C(12) | 1.40 | 1.50 | H(43)-C(22)-C(21) | 120.80 | 109.41 | C(6)-C(19)-C(20)-C(21) | 0.77 |
| N(9)-H(36) | 1.01 | 1.02 | C(23)-C(22)-C(21) | 119.90 | 109.5 | C(6)-C(19)-C(20)-H(42) | -179.58 |
| N(9)-C(11) | 1.40 | 1.46 | O(26)-C(21)-C(22) | 124.43 | 107.7 | H(41)-C(19)-C(20)-C(21) | -179.61 |
| C(8)-O(10) | 1.22 | 1.21 | O(26)-C(21)-C(20) | 115.89 |  | H(41)-C(19)-C(20)-H(42) | 0.04 |
| C(8)-N(9) | 1.39 | 1.37 | C(22)-C(21)-C(20) | 119.68 | 109.51 | C(2)-C(18)-C(35)-H(57) | 61.40 |
| C(7)-C(18) | 1.37 | 1.42 | H(42)-C(20)-C(21) | 118.65 | 118.2 | C(2)-C(18)-C(35)-H(58) | -178.01 |
| C(7)-C(8) | 1.48 | 1.52 | H(42)-C(20)-C(19) | 121.37 | 120 | C(2)-C(18)-C(35)-H(59) | -55.18 |
| C(6)-C(23) | 1.40 | 1.52 | C(21)-C(20)-C(19) | 119.98 | 122 | C(7)-C(18)-C(35)-H(57) | -120.08 |
| C(6)-C(19) | 1.40 | 1.50 | H(41)-C(19)-C(20) | 120.64 | 120 | C(7)-C(18)-C(35)-H(58) | 0.51 |
| N(5)-C(6) | 1.41 | 1.47 | H(41)-C(19)-C(6) | 118.42 | 118.2 | C(7)-C(18)-C(35)-H(59) | 123.34 |
| N(4)-N(5) | 1.26 | 1.25 | C(20)-C(19)-C(6) | 120.94 | 122 | C(14)-C(17)-C(25)-H(45) | 59.51 |
| C(3)-N(28) | 1.36 | 1.47 | C(35)-C(18)-C(7) | 124.27 | 121.4 | C(14)-C(17)-C(25)-H(46) | 179.76 |
| C(2)-C(18) | 1.44 | 1.50 | C(35)-C(18)-C(2) | 123.88 | 117.2 | C(14)-C(17)-C(25)-H(47) | -60.05 |
| C(2)-N(4) | 1.38 | 1.47 | C(7)-C(18)-C(2) | 111.84 | 121.4 | O(24)-C(17)-C(25)-H(45) | -120.50 |
| C(2)-C(3) | 1.41 | 1.52 | C(25)-C(17)-O(24) | 120.27 | 122.5 | O(24)-C(17)-C(25)-H(46) | -0.24 |
| S(1)-C(7) | 1.78 | 1.66 | C(25)-C(17)-C(14) | 118.83 | 116.6 | O(24)-C(17)-C(25)-H(47) | 119.95 |
| S(1)-C(3) | 1.73 |  | O(24)-C(17)-C(14) | 120.90 | 122.5 | C(14)-C(15)-C(16)-C(11) | 0.18 |
|  |  |  | H(40)-C(16)-C(15) | 120.87 | 109.41 | C(14)-C(15)-C(16)-H(40) | -179.24 |
|  |  |  | H(40)-C(16)-C(11) | 119.67 | 109.41 | H(39)-C(15)-C(16)-C(11) | -179.97 |
|  |  |  | C(15)-C(16)-C(11) | 119.45 | 109.5 | H(39)-C(15)-C(16)-H(40) | 0.61 |
|  |  |  | H(39)-C(15)-C(16) | 119.89 | 109.41 | C(13)-C(14)-C(17)-O(24) | -179.82 |
|  |  |  | H(39)-C(15)-C(14) | 118.15 | 109.41 | C(13)-C(14)-C(17)-C(25) | 0.18 |
|  |  |  | C(16)-C(15)-C(14) | 121.96 | 109.5 | C(15)-C(14)-C(17)-O(24) | 0.26 |
|  |  |  | C(17)-C(14)-C(15) | 119.01 | 109.9 | C(15)-C(14)-C(17)-C(25) | -179.75 |
|  |  |  | C(17)-C(14)-C(13) | 122.95 | 110.51 | C(13)-C(14)-C(15)-C(16) | -0.07 |
|  |  |  | C(15)-C(14)-C(13) | 118.04 | 109.51 | C(13)-C(14)-C(15)-H(39) | -179.92 |
|  |  |  | H(38)-C(13)-C(14) | 120.63 | 118.2 | C(17)-C(14)-C(15)-C(16) | 179.86 |
|  |  |  | H(38)-C(13)-C(12) | 118.61 | 120 | C(17)-C(14)-C(15)-H(39) | 0.01 |
|  |  |  | C(14)-C(13)-C(12) | 120.76 | 122 | C(12)-C(13)-C(14)-C(15) | -0.07 |
|  |  |  | H(37)-C(12)-C(13) | 119.66 | 120 | C(12)-C(13)-C(14)-C(17) | -180.00 |
|  |  |  | H(37)-C(12)-C(11) | 119.64 | 118.2 | H(38)-C(13)-C(14)-C(15) | 179.96 |
|  |  |  | C(13)-C(12)-C(11) | 120.70 | 122 | H(38)-C(13)-C(14)-C(17) | 0.03 |
|  |  |  | C(16)-C(11)-C(12) | 119.08 | 109.51 | C(11)-C(12)-C(13)-C(14) | 0.10 |
|  |  |  | C(16)-C(11)-N(9) | 123.85 | 110.78 | C(11)-C(12)-C(13)-H(38) | -179.93 |
|  |  |  | C(12)-C(11)-N(9) | 117.06 |  | H(37)-C(12)-C(13)-C(14) | -179.84 |
|  |  |  | H(36)-N(9)-C(11) | 114.57 | 118 | H(37)-C(12)-C(13)-H(38) | 0.14 |
|  |  |  | H(36)-N(9)-C(8) | 116.13 | 117.4 | N(9)-C(11)-C(16)-C(15) | 179.12 |
|  |  |  | C(11)-N(9)-C(8) | 128.96 |  | N(9)-C(11)-C(16)-H(40) | -1.46 |
|  |  |  | O(10)-C(8)-N(9) | 122.76 | 122.6 | C(12)-C(11)-C(16)-C(15) | -0.15 |
|  |  |  | O(10)-C(8)-C(7) | 123.03 | 123 | C(12)-C(11)-C(16)-H(40) | 179.28 |
|  |  |  | N(9)-C(8)-C(7) | 114.21 | 112.74 | N(9)-C(11)-C(12)-C(13) | -179.30 |
|  |  |  | C(18)-C(7)-C(8) | 128.34 | 117.6 | N(9)-C(11)-C(12)-H(37) | 0.63 |
|  |  |  | C(18)-C(7)-S(1) | 112.13 | 119 | C(16)-C(11)-C(12)-C(13) | 0.01 |
|  |  |  | C(8)-C(7)-S(1) | 119.52 |  | C(16)-C(11)-C(12)-H(37) | 179.95 |
|  |  |  | C(23)-C(6)-C(19) | 118.71 | 109.51 | C(8)-N(9)-C(11)-C(12) | -174.72 |
|  |  |  | C(23)-C(6)-N(5) | 125.45 |  | C(8)-N(9)-C(11)-C(16) | 6.00 |
|  |  |  | C(19)-C(6)-N(5) | 115.83 |  | H(36)-N(9)-C(11)-C(12) | -1.72 |
|  |  |  | C(6)-N(5)-N(4) | 115.57 | 106.5 | H(36)-N(9)-C(11)-C(16) | 179.00 |
|  |  |  | N(5)-N(4)-C(2) | 118.27 | 106.5 | C(7)-C(8)-N(9)-C(11) | -175.30 |
|  |  |  | N(28)-C(3)-C(2) | 122.51 |  | C(7)-C(8)-N(9)-H(36) | 11.79 |
|  |  |  | N(28)-C(3)-S(1) | 126.06 |  | O(10)-C(8)-N(9)-C(11) | 4.71 |
|  |  |  | C(2)-C(3)-S(1) | 111.41 |  | O(10)-C(8)-N(9)-H(36) | -168.20 |
|  |  |  | C(18)-C(2)-N(4) | 131.71 |  | S(1)-C(7)-C(18)-C(2) | -0.61 |
|  |  |  | C(18)-C(2)-C(3) | 113.30 | 109.51 | S(1)-C(7)-C(18)-C(35) | -179.28 |
|  |  |  | N(4)-C(2)-C(3) | 114.99 |  | C(8)-C(7)-C(18)-C(2) | -179.54 |
|  |  |  | C(7)-S(1)-C(3) | 91.32 |  | C(8)-C(7)-C(18)-C(35) | 1.78 |
|  |  |  |  |  |  | S(1)-C(7)-C(8)-N(9) | 27.73 |
|  |  |  |  |  |  | S(1)-C(7)-C(8)-O(10) | -152.28 |
|  |  |  |  |  |  | C(18)-C(7)-C(8)-N(9) | -153.40 |
|  |  |  |  |  |  | C(18)-C(7)-C(8)-O(10) | 26.59 |
|  |  |  |  |  |  | N(5)-C(6)-C(23)-C(22) | 179.40 |
|  |  |  |  |  |  | N(5)-C(6)-C(23)-H(44) | 0.41 |
|  |  |  |  |  |  | C(19)-C(6)-C(23)-C(22) | 0.81 |
|  |  |  |  |  |  | C(19)-C(6)-C(23)-H(44) | -178.18 |
|  |  |  |  |  |  | N(5)-C(6)-C(19)-C(20) | -179.96 |
|  |  |  |  |  |  | N(5)-C(6)-C(19)-H(41) | 0.41 |
|  |  |  |  |  |  | C(23)-C(6)-C(19)-C(20) | -1.23 |
|  |  |  |  |  |  | C(23)-C(6)-C(19)-H(41) | 179.13 |
|  |  |  |  |  |  | N(4)-N(5)-C(6)-C(19) | -170.24 |
|  |  |  |  |  |  | N(4)-N(5)-C(6)-C(23) | 11.13 |
|  |  |  |  |  |  | C(2)-N(4)-N(5)-C(6) | -178.39 |
|  |  |  |  |  |  | S(1)-C(3)-N(28)-C(29) | 2.46 |
|  |  |  |  |  |  | S(1)-C(3)-N(28)-H(51) | -174.58 |
|  |  |  |  |  |  | C(2)-C(3)-N(28)-C(29) | -179.06 |
|  |  |  |  |  |  | C(2)-C(3)-N(28)-H(51) | 3.90 |
|  |  |  |  |  |  | C(3)-C(2)-C(18)-C(7) | 0.03 |
|  |  |  |  |  |  | C(3)-C(2)-C(18)-C(35) | 178.72 |
|  |  |  |  |  |  | N(4)-C(2)-C(18)-C(7) | -179.79 |
|  |  |  |  |  |  | N(4)-C(2)-C(18)-C(35) | -1.11 |
|  |  |  |  |  |  | C(3)-C(2)-N(4)-N(5) | -177.32 |
|  |  |  |  |  |  | C(18)-C(2)-N(4)-N(5) | 2.50 |
|  |  |  |  |  |  | N(4)-C(2)-C(3)-S(1) | -179.58 |
|  |  |  |  |  |  | N(4)-C(2)-C(3)-N(28) | 1.74 |
|  |  |  |  |  |  | C(18)-C(2)-C(3)-S(1) | 0.57 |
|  |  |  |  |  |  | C(18)-C(2)-C(3)-N(28) | -178.12 |
|  |  |  |  |  |  | C(3)-S(1)-C(7)-C(8) | 179.83 |
|  |  |  |  |  |  | C(3)-S(1)-C(7)-C(18) | 0.79 |
|  |  |  |  |  |  | C(7)-S(1)-C(3)-C(2) | -0.76 |
|  |  |  |  |  |  | C(7)-S(1)-C(3)-N(28) | 177.87 |

| **5c** |  |  |  |  |  |  |  |
| --- | --- | --- | --- | --- | --- | --- | --- |
| **Atoms** | **Actual (°/A°)** | **Optimal (°/A°)** | **Atoms** | **Actual (°/A°)** | **Optimal (°/A°)** | **Atoms** | **Actual (°/A°)** |
| C(34)-H(55) | 1.09 | 1.11 | H(55)-C(34)-H(54) | 109.29 | 109 | C(30)-C(31)-C(32)-C(27) | -0.75 |
| C(34)-H(54) | 1.09 | 1.11 | H(55)-C(34)-H(53) | 107.53 | 109 | C(30)-C(31)-C(32)-H(49) | 177.11 |
| C(34)-H(53) | 1.09 | 1.11 | H(55)-C(34)-C(17) | 111.00 | 109.47 | H(48)-C(31)-C(32)-C(27) | -179.89 |
| C(33)-H(52) | 1.09 | 1.11 | H(54)-C(34)-H(53) | 109.31 | 109 | H(48)-C(31)-C(32)-H(49) | -2.02 |
| C(33)-H(51) | 1.09 | 1.11 | H(54)-C(34)-C(17) | 108.65 | 109.47 | C(29)-C(30)-C(31)-C(32) | -0.16 |
| C(33)-H(50) | 1.09 | 1.11 | H(53)-C(34)-C(17) | 111.03 | 109.47 | C(29)-C(30)-C(31)-H(48) | 178.97 |
| C(32)-H(49) | 1.08 | 1.10 | H(52)-C(33)-H(51) | 108.88 | 109 | H(47)-C(30)-C(31)-C(32) | -179.79 |
| C(31)-H(48) | 1.08 | 1.10 | H(52)-C(33)-H(50) | 110.60 | 109 | H(47)-C(30)-C(31)-H(48) | -0.66 |
| C(31)-C(32) | 1.39 | 1.42 | H(52)-C(33)-C(18) | 110.90 | 110 | C(28)-C(29)-C(30)-C(31) | 0.71 |
| C(30)-H(47) | 1.08 | 1.10 | H(51)-C(33)-H(50) | 106.06 | 109 | C(28)-C(29)-C(30)-H(47) | -179.66 |
| C(30)-C(31) | 1.39 | 1.42 | H(51)-C(33)-C(18) | 110.08 | 110 | H(46)-C(29)-C(30)-C(31) | -179.74 |
| C(29)-H(46) | 1.08 | 1.10 | H(50)-C(33)-C(18) | 110.20 | 110 | H(46)-C(29)-C(30)-H(47) | -0.11 |
| C(29)-C(30) | 1.40 | 1.42 | H(49)-C(32)-C(31) | 119.21 | 120 | C(27)-C(28)-C(29)-C(30) | -0.35 |
| C(28)-H(45) | 1.09 | 1.10 | H(49)-C(32)-C(27) | 120.93 | 120 | C(27)-C(28)-C(29)-H(46) | -179.90 |
| C(28)-C(29) | 1.39 | 1.42 | C(31)-C(32)-C(27) | 119.83 |  | H(45)-C(28)-C(29)-C(30) | 179.06 |
| C(27)-C(32) | 1.40 | 1.42 | H(48)-C(31)-C(32) | 118.96 | 120 | H(45)-C(28)-C(29)-H(46) | -0.50 |
| C(27)-C(28) | 1.40 | 1.42 | H(48)-C(31)-C(30) | 120.05 | 120 | N(26)-C(27)-C(32)-C(31) | 179.48 |
| N(26)-H(44) | 1.02 | 1.05 | C(32)-C(31)-C(30) | 120.99 |  | N(26)-C(27)-C(32)-H(49) | 1.65 |
| N(26)-C(27) | 1.40 | 1.46 | H(47)-C(30)-C(31) | 120.42 | 120 | C(28)-C(27)-C(32)-C(31) | 1.11 |
| C(23)-H(43) | 1.08 | 1.10 | H(47)-C(30)-C(29) | 120.42 | 120 | C(28)-C(27)-C(32)-H(49) | -176.72 |
| C(22)-H(42) | 1.08 | 1.10 | C(31)-C(30)-C(29) | 119.16 |  | N(26)-C(27)-C(28)-C(29) | -179.04 |
| C(22)-C(23) | 1.39 | 1.42 | H(46)-C(29)-C(30) | 120.20 | 120 | N(26)-C(27)-C(28)-H(45) | 1.55 |
| C(21)-Cl(24) | 1.76 | 1.72 | H(46)-C(29)-C(28) | 119.36 | 120 | C(32)-C(27)-C(28)-C(29) | -0.57 |
| C(21)-C(22) | 1.40 | 1.42 | C(30)-C(29)-C(28) | 120.44 |  | C(32)-C(27)-C(28)-H(45) | -179.97 |
| C(20)-H(41) | 1.08 | 1.10 | H(45)-C(28)-C(29) | 120.08 | 120 | C(3)-N(26)-C(27)-C(28) | -158.52 |
| C(20)-C(21) | 1.39 | 1.42 | H(45)-C(28)-C(27) | 119.47 | 120 | C(3)-N(26)-C(27)-C(32) | 23.08 |
| C(19)-H(40) | 1.08 | 1.10 | C(29)-C(28)-C(27) | 120.45 |  | H(44)-N(26)-C(27)-C(28) | 17.86 |
| C(19)-C(20) | 1.39 | 1.42 | C(32)-C(27)-C(28) | 119.13 | 120 | H(44)-N(26)-C(27)-C(32) | -160.54 |
| C(18)-C(33) | 1.50 | 1.50 | C(32)-C(27)-N(26) | 123.58 | 120 | C(21)-C(22)-C(23)-C(6) | 0.04 |
| C(17)-C(34) | 1.52 | 1.51 | C(28)-C(27)-N(26) | 117.27 | 120 | C(21)-C(22)-C(23)-H(43) | 178.89 |
| C(17)-O(25) | 1.22 | 1.21 | H(44)-N(26)-C(27) | 116.71 | 118 | H(42)-C(22)-C(23)-C(6) | -179.39 |
| C(16)-H(39) | 1.08 | 1.10 | H(44)-N(26)-C(3) | 111.07 |  | H(42)-C(22)-C(23)-H(43) | -0.54 |
| C(15)-H(38) | 1.08 | 1.10 | C(27)-N(26)-C(3) | 132.14 |  | C(20)-C(21)-C(22)-C(23) | -0.61 |
| C(15)-C(16) | 1.39 | 1.42 | H(43)-C(23)-C(22) | 120.14 | 120 | C(20)-C(21)-C(22)-H(42) | 178.83 |
| C(14)-C(17) | 1.49 | 1.52 | H(43)-C(23)-C(6) | 119.51 | 120 | Cl(24)-C(21)-C(22)-C(23) | -179.96 |
| C(14)-C(15) | 1.40 | 1.42 | C(22)-C(23)-C(6) | 120.34 |  | Cl(24)-C(21)-C(22)-H(42) | -0.52 |
| C(13)-H(37) | 1.08 | 1.10 | H(42)-C(22)-C(23) | 120.68 | 120 | C(19)-C(20)-C(21)-C(22) | 0.16 |
| C(13)-C(14) | 1.40 | 1.42 | H(42)-C(22)-C(21) | 119.85 | 120 | C(19)-C(20)-C(21)-Cl(24) | 179.51 |
| C(12)-H(36) | 1.09 | 1.10 | C(23)-C(22)-C(21) | 119.47 |  | H(41)-C(20)-C(21)-C(22) | -179.52 |
| C(12)-C(13) | 1.39 | 1.42 | Cl(24)-C(21)-C(22) | 119.32 | 118.8 | H(41)-C(20)-C(21)-Cl(24) | -0.17 |
| C(11)-C(16) | 1.40 | 1.42 | Cl(24)-C(21)-C(20) | 119.54 | 118.8 | C(6)-C(19)-C(20)-C(21) | 0.86 |
| C(11)-C(12) | 1.40 | 1.42 | C(22)-C(21)-C(20) | 121.14 | 120 | C(6)-C(19)-C(20)-H(41) | -179.47 |
| N(9)-H(35) | 1.01 | 1.01 | H(41)-C(20)-C(21) | 120.17 | 120 | H(40)-C(19)-C(20)-C(21) | -179.55 |
| N(9)-C(11) | 1.40 | 1.35 | H(41)-C(20)-C(19) | 120.79 | 120 | H(40)-C(19)-C(20)-H(41) | 0.13 |
| C(8)-O(10) | 1.22 | 1.21 | C(21)-C(20)-C(19) | 119.04 |  | C(2)-C(18)-C(33)-H(50) | -55.55 |
| C(8)-N(9) | 1.39 | 1.37 | H(40)-C(19)-C(20) | 120.58 | 120 | C(2)-C(18)-C(33)-H(51) | 61.08 |
| C(7)-C(18) | 1.37 | 1.42 | H(40)-C(19)-C(6) | 118.61 | 120 | C(2)-C(18)-C(33)-H(52) | -178.36 |
| C(7)-C(8) | 1.48 | 1.52 | C(20)-C(19)-C(6) | 120.81 |  | C(7)-C(18)-C(33)-H(50) | 123.08 |
| C(6)-C(23) | 1.40 | 1.42 | C(33)-C(18)-C(7) | 124.39 | 121.4 | C(7)-C(18)-C(33)-H(51) | -120.28 |
| C(6)-C(19) | 1.40 | 1.42 | C(33)-C(18)-C(2) | 123.84 | 117.2 | C(7)-C(18)-C(33)-H(52) | 0.27 |
| N(5)-C(6) | 1.41 | 1.46 | C(7)-C(18)-C(2) | 111.77 | 121.4 | C(14)-C(17)-C(34)-H(53) | 59.58 |
| N(4)-N(5) | 1.26 |  | C(34)-C(17)-O(25) | 120.32 | 122.5 | C(14)-C(17)-C(34)-H(54) | 179.82 |
| C(3)-N(26) | 1.36 | 1.47 | C(34)-C(17)-C(14) | 118.83 | 115 | C(14)-C(17)-C(34)-H(55) | -59.98 |
| C(2)-C(18) | 1.45 | 1.50 | O(25)-C(17)-C(14) | 120.85 | 123 | O(25)-C(17)-C(34)-H(53) | -120.44 |
| C(2)-N(4) | 1.38 | 1.44 | H(39)-C(16)-C(15) | 120.85 | 120 | O(25)-C(17)-C(34)-H(54) | -0.20 |
| C(2)-C(3) | 1.41 | 1.52 | H(39)-C(16)-C(11) | 119.71 | 120 | O(25)-C(17)-C(34)-H(55) | 120.00 |
| S(1)-C(7) | 1.78 | 1.66 | C(15)-C(16)-C(11) | 119.43 |  | C(14)-C(15)-C(16)-C(11) | 0.18 |
| S(1)-C(3) | 1.73 |  | H(38)-C(15)-C(16) | 119.90 | 120 | C(14)-C(15)-C(16)-H(39) | -179.28 |
|  |  |  | H(38)-C(15)-C(14) | 118.16 | 120 | H(38)-C(15)-C(16)-C(11) | -179.96 |
|  |  |  | C(16)-C(15)-C(14) | 121.94 |  | H(38)-C(15)-C(16)-H(39) | 0.58 |
|  |  |  | C(17)-C(14)-C(15) | 118.98 | 117.6 | C(13)-C(14)-C(17)-O(25) | -179.89 |
|  |  |  | C(17)-C(14)-C(13) | 122.95 | 117.6 | C(13)-C(14)-C(17)-C(34) | 0.08 |
|  |  |  | C(15)-C(14)-C(13) | 118.07 | 120 | C(15)-C(14)-C(17)-O(25) | 0.17 |
|  |  |  | H(37)-C(13)-C(14) | 120.63 | 120 | C(15)-C(14)-C(17)-C(34) | -179.85 |
|  |  |  | H(37)-C(13)-C(12) | 118.62 | 120 | C(13)-C(14)-C(15)-C(16) | -0.06 |
|  |  |  | C(14)-C(13)-C(12) | 120.75 |  | C(13)-C(14)-C(15)-H(38) | -179.93 |
|  |  |  | H(36)-C(12)-C(13) | 119.65 | 120 | C(17)-C(14)-C(15)-C(16) | 179.87 |
|  |  |  | H(36)-C(12)-C(11) | 119.67 | 120 | C(17)-C(14)-C(15)-H(38) | 0.01 |
|  |  |  | C(13)-C(12)-C(11) | 120.67 |  | C(12)-C(13)-C(14)-C(15) | -0.07 |
|  |  |  | C(16)-C(11)-C(12) | 119.13 | 120 | C(12)-C(13)-C(14)-C(17) | 179.99 |
|  |  |  | C(16)-C(11)-N(9) | 123.83 | 120 | H(37)-C(13)-C(14)-C(15) | 179.96 |
|  |  |  | C(12)-C(11)-N(9) | 117.03 | 120 | H(37)-C(13)-C(14)-C(17) | 0.03 |
|  |  |  | H(35)-N(9)-C(11) | 114.53 | 110 | C(11)-C(12)-C(13)-C(14) | 0.09 |
|  |  |  | H(35)-N(9)-C(8) | 116.17 | 117.4 | C(11)-C(12)-C(13)-H(37) | -179.94 |
|  |  |  | C(11)-N(9)-C(8) | 128.95 |  | H(36)-C(12)-C(13)-C(14) | -179.85 |
|  |  |  | O(10)-C(8)-N(9) | 122.92 | 122.6 | H(36)-C(12)-C(13)-H(37) | 0.12 |
|  |  |  | O(10)-C(8)-C(7) | 122.89 | 123 | N(9)-C(11)-C(16)-C(15) | 179.13 |
|  |  |  | N(9)-C(8)-C(7) | 114.19 | 112.74 | N(9)-C(11)-C(16)-H(39) | -1.41 |
|  |  |  | C(18)-C(7)-C(8) | 128.30 | 117.6 | C(12)-C(11)-C(16)-C(15) | -0.15 |
|  |  |  | C(18)-C(7)-S(1) | 112.22 | 119 | C(12)-C(11)-C(16)-H(39) | 179.31 |
|  |  |  | C(8)-C(7)-S(1) | 119.46 |  | N(9)-C(11)-C(12)-C(13) | -179.31 |
|  |  |  | C(23)-C(6)-C(19) | 119.19 | 120 | N(9)-C(11)-C(12)-H(36) | 0.63 |
|  |  |  | C(23)-C(6)-N(5) | 125.04 | 120 | C(16)-C(11)-C(12)-C(13) | 0.02 |
|  |  |  | C(19)-C(6)-N(5) | 115.75 | 120 | C(16)-C(11)-C(12)-H(36) | 179.96 |
|  |  |  | C(6)-N(5)-N(4) | 115.03 |  | C(8)-N(9)-C(11)-C(12) | -174.71 |
|  |  |  | N(5)-N(4)-C(2) | 118.53 |  | C(8)-N(9)-C(11)-C(16) | 6.00 |
|  |  |  | N(26)-C(3)-C(2) | 122.72 |  | H(35)-N(9)-C(11)-C(12) | -1.80 |
|  |  |  | N(26)-C(3)-S(1) | 125.93 |  | H(35)-N(9)-C(11)-C(16) | 178.91 |
|  |  |  | C(2)-C(3)-S(1) | 111.34 |  | C(7)-C(8)-N(9)-C(11) | -175.51 |
|  |  |  | C(18)-C(2)-N(4) | 131.68 |  | C(7)-C(8)-N(9)-H(35) | 11.68 |
|  |  |  | C(18)-C(2)-C(3) | 113.34 | 109.51 | O(10)-C(8)-N(9)-C(11) | 4.47 |
|  |  |  | N(4)-C(2)-C(3) | 114.98 | 108.8 | O(10)-C(8)-N(9)-H(35) | -168.34 |
|  |  |  | C(7)-S(1)-C(3) | 91.33 |  | S(1)-C(7)-C(18)-C(2) | -0.62 |
|  |  |  |  |  |  | S(1)-C(7)-C(18)-C(33) | -179.40 |
|  |  |  |  |  |  | C(8)-C(7)-C(18)-C(2) | -179.45 |
|  |  |  |  |  |  | C(8)-C(7)-C(18)-C(33) | 1.77 |
|  |  |  |  |  |  | S(1)-C(7)-C(8)-N(9) | 28.26 |
|  |  |  |  |  |  | S(1)-C(7)-C(8)-O(10) | -151.72 |
|  |  |  |  |  |  | C(18)-C(7)-C(8)-N(9) | -152.98 |
|  |  |  |  |  |  | C(18)-C(7)-C(8)-O(10) | 27.04 |
|  |  |  |  |  |  | N(5)-C(6)-C(23)-C(22) | 179.19 |
|  |  |  |  |  |  | N(5)-C(6)-C(23)-H(43) | 0.34 |
|  |  |  |  |  |  | C(19)-C(6)-C(23)-C(22) | 0.94 |
|  |  |  |  |  |  | C(19)-C(6)-C(23)-H(43) | -177.91 |
|  |  |  |  |  |  | N(5)-C(6)-C(19)-C(20) | -179.81 |
|  |  |  |  |  |  | N(5)-C(6)-C(19)-H(40) | 0.59 |
|  |  |  |  |  |  | C(23)-C(6)-C(19)-C(20) | -1.40 |
|  |  |  |  |  |  | C(23)-C(6)-C(19)-H(40) | 178.99 |
|  |  |  |  |  |  | N(4)-N(5)-C(6)-C(19) | -167.32 |
|  |  |  |  |  |  | N(4)-N(5)-C(6)-C(23) | 14.38 |
|  |  |  |  |  |  | C(2)-N(4)-N(5)-C(6) | -178.16 |
|  |  |  |  |  |  | S(1)-C(3)-N(26)-C(27) | 2.35 |
|  |  |  |  |  |  | S(1)-C(3)-N(26)-H(44) | -174.18 |
|  |  |  |  |  |  | C(2)-C(3)-N(26)-C(27) | -179.31 |
|  |  |  |  |  |  | C(2)-C(3)-N(26)-H(44) | 4.15 |
|  |  |  |  |  |  | C(3)-C(2)-C(18)-C(7) | -0.02 |
|  |  |  |  |  |  | C(3)-C(2)-C(18)-C(33) | 178.77 |
|  |  |  |  |  |  | N(4)-C(2)-C(18)-C(7) | -179.86 |
|  |  |  |  |  |  | N(4)-C(2)-C(18)-C(33) | -1.07 |
|  |  |  |  |  |  | C(3)-C(2)-N(4)-N(5) | -176.87 |
|  |  |  |  |  |  | C(18)-C(2)-N(4)-N(5) | 2.97 |
|  |  |  |  |  |  | N(4)-C(2)-C(3)-S(1) | -179.46 |
|  |  |  |  |  |  | N(4)-C(2)-C(3)-N(26) | 1.99 |
|  |  |  |  |  |  | C(18)-C(2)-C(3)-S(1) | 0.67 |
|  |  |  |  |  |  | C(18)-C(2)-C(3)-N(26) | -177.88 |
|  |  |  |  |  |  | C(3)-S(1)-C(7)-C(8) | 179.80 |
|  |  |  |  |  |  | C(3)-S(1)-C(7)-C(18) | 0.86 |
|  |  |  |  |  |  | C(7)-S(1)-C(3)-C(2) | -0.85 |
|  |  |  |  |  |  | C(7)-S(1)-C(3)-N(26) | 177.65 |

**Table S3** Bond length, bond angle, dihedral angle for compounds **7a-c**

| **7a** |  |  |  |  |  |  |  |
| --- | --- | --- | --- | --- | --- | --- | --- |
| **Atoms** | **Actual (°/A°)** | **Optimal (°/A°)** | **Atoms** | **Actual (°/A°)** | **Optimal (°/A°)** | **Atoms** | **Actual (°/A°)** |
| C(35)-H(59) | 1.10 | 1.11 | H(59)-C(35)-H(58) | 107.44 | 109 | C(31)-C(32)-C(33)-C(28) | -0.90 |
| C(35)-H(58) | 1.09 | 1.11 | H(59)-C(35)-H(57) | 107.28 | 109 | C(31)-C(32)-C(33)-H(53) | 176.82 |
| C(35)-H(57) | 1.09 | 1.11 | H(59)-C(35)-C(23) | 111.17 | 110 | H(52)-C(32)-C(33)-C(28) | -179.90 |
| C(34)-H(56) | 1.09 | 1.11 | H(58)-C(35)-H(57) | 107.99 | 109 | H(52)-C(32)-C(33)-H(53) | -2.18 |
| C(34)-H(55) | 1.09 | 1.11 | H(58)-C(35)-C(23) | 111.33 | 110 | C(30)-C(31)-C(32)-C(33) | -0.25 |
| C(34)-H(54) | 1.09 | 1.11 | H(57)-C(35)-C(23) | 111.43 | 110 | C(30)-C(31)-C(32)-H(52) | 178.73 |
| C(33)-H(53) | 1.08 | 1.10 | H(56)-C(34)-H(55) | 109.30 | 109 | H(51)-C(31)-C(32)-C(33) | -179.79 |
| C(32)-H(52) | 1.08 | 1.10 | H(56)-C(34)-H(54) | 107.52 | 109 | H(51)-C(31)-C(32)-H(52) | -0.80 |
| C(32)-C(33) | 1.39 | 1.42 | H(56)-C(34)-C(17) | 111.04 | 109.47 | C(29)-C(30)-C(31)-C(32) | 0.86 |
| C(31)-H(51) | 1.08 | 1.10 | H(55)-C(34)-H(54) | 109.28 | 109 | C(29)-C(30)-C(31)-H(51) | -179.60 |
| C(31)-C(32) | 1.39 | 1.42 | H(55)-C(34)-C(17) | 108.64 | 109.47 | H(50)-C(30)-C(31)-C(32) | -179.59 |
| C(30)-H(50) | 1.08 | 1.10 | H(54)-C(34)-C(17) | 111.03 | 109.47 | H(50)-C(30)-C(31)-H(51) | -0.06 |
| C(30)-C(31) | 1.40 | 1.42 | H(53)-C(33)-C(32) | 119.35 | 120 | C(28)-C(29)-C(30)-C(31) | -0.32 |
| C(29)-H(49) | 1.08 | 1.10 | H(53)-C(33)-C(28) | 120.74 | 120 | C(28)-C(29)-C(30)-H(50) | -179.87 |
| C(29)-C(30) | 1.39 | 1.42 | C(32)-C(33)-C(28) | 119.88 |  | H(49)-C(29)-C(30)-C(31) | 179.28 |
| C(28)-C(33) | 1.40 | 1.42 | H(52)-C(32)-C(33) | 119.05 | 120 | H(49)-C(29)-C(30)-H(50) | -0.27 |
| C(28)-C(29) | 1.40 | 1.42 | H(52)-C(32)-C(31) | 120.05 | 120 | N(27)-C(28)-C(33)-C(32) | 179.30 |
| N(27)-H(48) | 1.03 | 1.05 | C(33)-C(32)-C(31) | 120.89 |  | N(27)-C(28)-C(33)-H(53) | 1.61 |
| N(27)-C(28) | 1.41 | 1.46 | H(51)-C(31)-C(32) | 120.40 | 120 | C(29)-C(28)-C(33)-C(32) | 1.43 |
| C(25)-H(47) | 1.08 | 1.10 | H(51)-C(31)-C(30) | 120.39 | 120 | C(29)-C(28)-C(33)-H(53) | -176.25 |
| C(24)-H(46) | 1.09 | 1.10 | C(32)-C(31)-C(30) | 119.21 |  | N(27)-C(28)-C(29)-C(30) | -178.84 |
| C(24)-C(25) | 1.39 | 1.42 | H(50)-C(30)-C(31) | 120.17 | 120 | N(27)-C(28)-C(29)-H(49) | 1.56 |
| C(23)-C(35) | 1.51 | 1.50 | H(50)-C(30)-C(29) | 119.38 | 120 | C(33)-C(28)-C(29)-C(30) | -0.83 |
| C(23)-C(24) | 1.40 | 1.42 | C(31)-C(30)-C(29) | 120.45 |  | C(33)-C(28)-C(29)-H(49) | 179.56 |
| C(22)-H(45) | 1.09 | 1.10 | H(49)-C(29)-C(30) | 120.30 | 120 | C(3)-N(27)-C(28)-C(29) | -153.44 |
| C(22)-C(23) | 1.40 | 1.42 | H(49)-C(29)-C(28) | 119.31 | 120 | C(3)-N(27)-C(28)-C(33) | 28.65 |
| C(21)-H(44) | 1.09 | 1.10 | C(30)-C(29)-C(28) | 120.38 |  | H(48)-N(27)-C(28)-C(29) | 22.44 |
| C(21)-C(22) | 1.39 | 1.42 | C(33)-C(28)-C(29) | 119.18 | 120 | H(48)-N(27)-C(28)-C(33) | -155.47 |
| N(19)-H(43) | 1.01 | 1.05 | C(33)-C(28)-N(27) | 123.66 | 120 | C(23)-C(24)-C(25)-C(6) | -0.16 |
| N(19)-H(42) | 1.02 | 1.05 | C(29)-C(28)-N(27) | 117.13 | 120 | C(23)-C(24)-C(25)-H(47) | 179.63 |
| C(18)-N(19) | 1.39 | 1.46 | H(48)-N(27)-C(28) | 117.98 | 118 | H(46)-C(24)-C(25)-C(6) | 179.85 |
| C(17)-C(34) | 1.52 | 1.51 | H(48)-N(27)-C(3) | 110.69 | 118 | H(46)-C(24)-C(25)-H(47) | -0.36 |
| C(17)-O(26) | 1.22 | 1.21 | C(28)-N(27)-C(3) | 131.22 | 124 | C(22)-C(23)-C(35)-H(57) | 38.04 |
| C(16)-H(41) | 1.08 | 1.11 | H(47)-C(25)-C(24) | 120.71 | 120 | C(22)-C(23)-C(35)-H(58) | 158.66 |
| C(15)-H(40) | 1.08 | 1.11 | H(47)-C(25)-C(6) | 119.81 | 120 | C(22)-C(23)-C(35)-H(59) | -81.58 |
| C(15)-C(16) | 1.39 | 1.52 | C(24)-C(25)-C(6) | 119.48 |  | C(24)-C(23)-C(35)-H(57) | -143.20 |
| C(14)-C(17) | 1.49 | 1.51 | H(46)-C(24)-C(25) | 118.37 | 120 | C(24)-C(23)-C(35)-H(58) | -22.57 |
| C(14)-C(15) | 1.40 | 1.52 | H(46)-C(24)-C(23) | 119.30 | 120 | C(24)-C(23)-C(35)-H(59) | 97.18 |
| C(13)-H(39) | 1.08 | 1.10 | C(25)-C(24)-C(23) | 122.33 |  | C(22)-C(23)-C(24)-C(25) | 0.24 |
| C(13)-C(14) | 1.40 | 1.50 | C(35)-C(23)-C(24) | 121.50 | 121.4 | C(22)-C(23)-C(24)-H(46) | -179.77 |
| C(12)-H(38) | 1.09 | 1.10 | C(35)-C(23)-C(22) | 121.11 | 121.4 | C(35)-C(23)-C(24)-C(25) | -178.58 |
| C(12)-C(13) | 1.39 | 1.42 | C(24)-C(23)-C(22) | 117.39 | 120 | C(35)-C(23)-C(24)-H(46) | 1.42 |
| C(11)-C(16) | 1.41 | 1.52 | H(45)-C(22)-C(23) | 119.73 | 120 | C(21)-C(22)-C(23)-C(24) | -0.21 |
| C(11)-C(12) | 1.41 | 1.50 | H(45)-C(22)-C(21) | 119.01 | 120 | C(21)-C(22)-C(23)-C(35) | 178.60 |
| N(9)-H(37) | 1.01 | 1.02 | C(23)-C(22)-C(21) | 121.25 |  | H(45)-C(22)-C(23)-C(24) | 179.76 |
| N(9)-C(11) | 1.40 | 1.46 | H(44)-C(21)-C(22) | 119.66 | 120 | H(45)-C(22)-C(23)-C(35) | -1.42 |
| C(8)-O(10) | 1.24 | 1.21 | H(44)-C(21)-C(6) | 119.68 | 120 | C(6)-C(21)-C(22)-C(23) | 0.12 |
| C(8)-N(9) | 1.39 | 1.37 | C(22)-C(21)-C(6) | 120.66 |  | C(6)-C(21)-C(22)-H(45) | -179.86 |
| C(7)-C(18) | 1.39 | 1.42 | H(43)-N(19)-H(42) | 113.10 | 118.8 | H(44)-C(21)-C(22)-C(23) | -179.92 |
| C(7)-C(8) | 1.46 | 1.52 | H(43)-N(19)-C(18) | 114.57 |  | H(44)-C(21)-C(22)-H(45) | 0.11 |
| C(6)-C(25) | 1.40 | 1.42 | H(42)-N(19)-C(18) | 111.43 |  | C(2)-C(18)-N(19)-H(42) | -177.38 |
| C(6)-C(21) | 1.40 | 1.42 | N(19)-C(18)-C(7) | 123.01 | 120 | C(2)-C(18)-N(19)-H(43) | -47.38 |
| N(5)-H(36) | 1.01 | 1.01 | N(19)-C(18)-C(2) | 123.17 | 120 | C(7)-C(18)-N(19)-H(42) | 3.93 |
| N(5)-C(6) | 1.41 | 1.35 | C(7)-C(18)-C(2) | 113.82 | 120 | C(7)-C(18)-N(19)-H(43) | 133.93 |
| C(4)-O(20) | 1.24 | 1.21 | C(34)-C(17)-O(26) | 120.26 | 122.5 | C(14)-C(17)-C(34)-H(54) | 59.95 |
| C(4)-N(5) | 1.37 | 1.37 | C(34)-C(17)-C(14) | 118.84 | 116.6 | C(14)-C(17)-C(34)-H(55) | -179.85 |
| C(3)-N(27) | 1.35 | 1.46 | O(26)-C(17)-C(14) | 120.90 | 122.5 | C(14)-C(17)-C(34)-H(56) | -59.62 |
| C(2)-C(18) | 1.44 | 1.42 | H(41)-C(16)-C(15) | 120.96 | 109.41 | O(26)-C(17)-C(34)-H(54) | -120.08 |
| C(2)-C(4) | 1.48 | 1.52 | H(41)-C(16)-C(11) | 119.59 | 109.41 | O(26)-C(17)-C(34)-H(55) | 0.12 |
| C(2)-C(3) | 1.40 | 1.42 | C(15)-C(16)-C(11) | 119.45 | 109.5 | O(26)-C(17)-C(34)-H(56) | 120.35 |
| S(1)-C(7) | 1.77 | 1.66 | H(40)-C(15)-C(16) | 119.87 | 109.41 | C(14)-C(15)-C(16)-C(11) | 0.09 |
| S(1)-C(3) | 1.75 | 1.66 | H(40)-C(15)-C(14) | 118.13 | 109.41 | C(14)-C(15)-C(16)-H(41) | -179.68 |
|  |  |  | C(16)-C(15)-C(14) | 122.01 | 109.5 | H(40)-C(15)-C(16)-C(11) | -179.96 |
|  |  |  | C(17)-C(14)-C(15) | 119.00 | 109.9 | H(40)-C(15)-C(16)-H(41) | 0.26 |
|  |  |  | C(17)-C(14)-C(13) | 122.99 | 110.51 | C(13)-C(14)-C(17)-O(26) | 179.86 |
|  |  |  | C(15)-C(14)-C(13) | 118.01 | 109.51 | C(13)-C(14)-C(17)-C(34) | -0.17 |
|  |  |  | H(39)-C(13)-C(14) | 120.66 | 118.2 | C(15)-C(14)-C(17)-O(26) | -0.14 |
|  |  |  | H(39)-C(13)-C(12) | 118.59 | 120 | C(15)-C(14)-C(17)-C(34) | 179.83 |
|  |  |  | C(14)-C(13)-C(12) | 120.76 | 122 | C(13)-C(14)-C(15)-C(16) | -0.02 |
|  |  |  | H(38)-C(12)-C(13) | 119.64 | 120 | C(13)-C(14)-C(15)-H(40) | -179.97 |
|  |  |  | H(38)-C(12)-C(11) | 119.63 | 118.2 | C(17)-C(14)-C(15)-C(16) | 179.98 |
|  |  |  | C(13)-C(12)-C(11) | 120.74 | 122 | C(17)-C(14)-C(15)-H(40) | 0.04 |
|  |  |  | C(16)-C(11)-C(12) | 119.04 | 109.51 | C(12)-C(13)-C(14)-C(15) | -0.04 |
|  |  |  | C(16)-C(11)-N(9) | 124.05 | 110.78 | C(12)-C(13)-C(14)-C(17) | 179.96 |
|  |  |  | C(12)-C(11)-N(9) | 116.91 |  | H(39)-C(13)-C(14)-C(15) | 179.97 |
|  |  |  | H(37)-N(9)-C(11) | 114.09 | 118 | H(39)-C(13)-C(14)-C(17) | -0.03 |
|  |  |  | H(37)-N(9)-C(8) | 116.49 | 117.4 | C(11)-C(12)-C(13)-C(14) | 0.03 |
|  |  |  | C(11)-N(9)-C(8) | 129.37 |  | C(11)-C(12)-C(13)-H(39) | -179.98 |
|  |  |  | O(10)-C(8)-N(9) | 122.06 | 122.6 | H(38)-C(12)-C(13)-C(14) | -179.95 |
|  |  |  | O(10)-C(8)-C(7) | 121.58 | 123 | H(38)-C(12)-C(13)-H(39) | 0.04 |
|  |  |  | N(9)-C(8)-C(7) | 116.36 | 112.74 | N(9)-C(11)-C(16)-C(15) | 179.55 |
|  |  |  | C(18)-C(7)-C(8) | 125.09 | 117.6 | N(9)-C(11)-C(16)-H(41) | -0.67 |
|  |  |  | C(18)-C(7)-S(1) | 110.66 | 119 | C(12)-C(11)-C(16)-C(15) | -0.11 |
|  |  |  | C(8)-C(7)-S(1) | 124.11 |  | C(12)-C(11)-C(16)-H(41) | 179.68 |
|  |  |  | C(25)-C(6)-C(21) | 118.89 | 120 | N(9)-C(11)-C(12)-C(13) | -179.63 |
|  |  |  | C(25)-C(6)-N(5) | 124.26 | 120 | N(9)-C(11)-C(12)-H(38) | 0.35 |
|  |  |  | C(21)-C(6)-N(5) | 116.85 | 120 | C(16)-C(11)-C(12)-C(13) | 0.05 |
|  |  |  | H(36)-N(5)-C(6) | 115.16 | 110 | C(16)-C(11)-C(12)-H(38) | -179.98 |
|  |  |  | H(36)-N(5)-C(4) | 115.38 | 117.4 | C(8)-N(9)-C(11)-C(12) | -178.11 |
|  |  |  | C(6)-N(5)-C(4) | 129.21 |  | C(8)-N(9)-C(11)-C(16) | 2.23 |
|  |  |  | O(20)-C(4)-N(5) | 122.73 | 122.6 | H(37)-N(9)-C(11)-C(12) | -1.01 |
|  |  |  | O(20)-C(4)-C(2) | 121.25 | 123 | H(37)-N(9)-C(11)-C(16) | 179.33 |
|  |  |  | N(5)-C(4)-C(2) | 115.98 | 112.74 | C(7)-C(8)-N(9)-C(11) | -177.86 |
|  |  |  | N(27)-C(3)-C(2) | 124.20 | 120 | C(7)-C(8)-N(9)-H(37) | 5.10 |
|  |  |  | N(27)-C(3)-S(1) | 124.09 |  | O(10)-C(8)-N(9)-C(11) | 2.53 |
|  |  |  | C(2)-C(3)-S(1) | 111.71 | 119 | O(10)-C(8)-N(9)-H(37) | -174.51 |
|  |  |  | C(18)-C(2)-C(4) | 127.93 | 117.6 | S(1)-C(7)-C(18)-C(2) | 0.50 |
|  |  |  | C(18)-C(2)-C(3) | 112.02 | 120 | S(1)-C(7)-C(18)-N(19) | 179.30 |
|  |  |  | C(4)-C(2)-C(3) | 119.98 | 117.6 | C(8)-C(7)-C(18)-C(2) | 176.31 |
|  |  |  | C(7)-S(1)-C(3) | 91.65 | 98.5 | C(8)-C(7)-C(18)-N(19) | -4.89 |
|  |  |  |  |  |  | S(1)-C(7)-C(8)-N(9) | 3.80 |
|  |  |  |  |  |  | S(1)-C(7)-C(8)-O(10) | -176.59 |
|  |  |  |  |  |  | C(18)-C(7)-C(8)-N(9) | -171.46 |
|  |  |  |  |  |  | C(18)-C(7)-C(8)-O(10) | 8.15 |
|  |  |  |  |  |  | N(5)-C(6)-C(25)-C(24) | -178.89 |
|  |  |  |  |  |  | N(5)-C(6)-C(25)-H(47) | 1.32 |
|  |  |  |  |  |  | C(21)-C(6)-C(25)-C(24) | 0.05 |
|  |  |  |  |  |  | C(21)-C(6)-C(25)-H(47) | -179.74 |
|  |  |  |  |  |  | N(5)-C(6)-C(21)-C(22) | 178.98 |
|  |  |  |  |  |  | N(5)-C(6)-C(21)-H(44) | -0.98 |
|  |  |  |  |  |  | C(25)-C(6)-C(21)-C(22) | -0.03 |
|  |  |  |  |  |  | C(25)-C(6)-C(21)-H(44) | -179.99 |
|  |  |  |  |  |  | C(4)-N(5)-C(6)-C(21) | 173.80 |
|  |  |  |  |  |  | C(4)-N(5)-C(6)-C(25) | -7.25 |
|  |  |  |  |  |  | H(36)-N(5)-C(6)-C(21) | -0.21 |
|  |  |  |  |  |  | H(36)-N(5)-C(6)-C(25) | 178.74 |
|  |  |  |  |  |  | C(2)-C(4)-N(5)-C(6) | -179.22 |
|  |  |  |  |  |  | C(2)-C(4)-N(5)-H(36) | -5.22 |
|  |  |  |  |  |  | O(20)-C(4)-N(5)-C(6) | -1.28 |
|  |  |  |  |  |  | O(20)-C(4)-N(5)-H(36) | 172.71 |
|  |  |  |  |  |  | S(1)-C(3)-N(27)-C(28) | 3.64 |
|  |  |  |  |  |  | S(1)-C(3)-N(27)-H(48) | -172.47 |
|  |  |  |  |  |  | C(2)-C(3)-N(27)-C(28) | -176.85 |
|  |  |  |  |  |  | C(2)-C(3)-N(27)-H(48) | 7.04 |
|  |  |  |  |  |  | C(3)-C(2)-C(18)-C(7) | -2.93 |
|  |  |  |  |  |  | C(3)-C(2)-C(18)-N(19) | 178.27 |
|  |  |  |  |  |  | C(4)-C(2)-C(18)-C(7) | 174.01 |
|  |  |  |  |  |  | C(4)-C(2)-C(18)-N(19) | -4.79 |
|  |  |  |  |  |  | C(3)-C(2)-C(4)-N(5) | 158.32 |
|  |  |  |  |  |  | C(3)-C(2)-C(4)-O(20) | -19.65 |
|  |  |  |  |  |  | C(18)-C(2)-C(4)-N(5) | -18.40 |
|  |  |  |  |  |  | C(18)-C(2)-C(4)-O(20) | 163.62 |
|  |  |  |  |  |  | C(4)-C(2)-C(3)-S(1) | -173.20 |
|  |  |  |  |  |  | C(4)-C(2)-C(3)-N(27) | 7.24 |
|  |  |  |  |  |  | C(18)-C(2)-C(3)-S(1) | 4.01 |
|  |  |  |  |  |  | C(18)-C(2)-C(3)-N(27) | -175.55 |
|  |  |  |  |  |  | C(3)-S(1)-C(7)-C(8) | -174.34 |
|  |  |  |  |  |  | C(3)-S(1)-C(7)-C(18) | 1.51 |
|  |  |  |  |  |  | C(7)-S(1)-C(3)-C(2) | -3.18 |
|  |  |  |  |  |  | C(7)-S(1)-C(3)-N(27) | 176.38 |

| **7b** |  |  |  |  |  |  |  |
| --- | --- | --- | --- | --- | --- | --- | --- |
| **Atoms** | **Actual (°/A°)** | **Optimal (°/A°)** | **Atoms** | **Actual (°/A°)** | **Optimal (°/A°)** | **Atoms** | **Actual (°/A°)** |
| C(36)-H(60) | 1.10 | 1.11 | H(60)-C(36)-H(59) | 109.42 | 109 | C(23)-O(35)-C(36)-H(58) | -179.94 |
| C(36)-H(59) | 1.10 | 1.11 | H(60)-C(36)-H(58) | 109.30 | 109 | C(23)-O(35)-C(36)-H(59) | -61.21 |
| C(36)-H(58) | 1.09 | 1.11 | H(60)-C(36)-O(35) | 111.45 | 106.7 | C(23)-O(35)-C(36)-H(60) | 61.32 |
| O(35)-C(36) | 1.42 | 1.40 | H(59)-C(36)-H(58) | 109.31 | 109 | C(31)-C(32)-C(33)-C(28) | -0.83 |
| C(34)-H(57) | 1.09 | 1.11 | H(59)-C(36)-O(35) | 111.41 | 106.7 | C(31)-C(32)-C(33)-H(54) | 176.90 |
| C(34)-H(56) | 1.09 | 1.11 | H(58)-C(36)-O(35) | 105.87 | 106.7 | H(53)-C(32)-C(33)-C(28) | -179.89 |
| C(34)-H(55) | 1.09 | 1.11 | C(36)-O(35)-C(23) | 118.43 | 106.8 | H(53)-C(32)-C(33)-H(54) | -2.16 |
| C(33)-H(54) | 1.08 | 1.10 | H(57)-C(34)-H(56) | 109.31 | 109 | C(30)-C(31)-C(32)-C(33) | -0.22 |
| C(32)-H(53) | 1.08 | 1.10 | H(57)-C(34)-H(55) | 109.30 | 109 | C(30)-C(31)-C(32)-H(53) | 178.83 |
| C(32)-C(33) | 1.39 | 1.42 | H(57)-C(34)-C(17) | 108.66 | 109.47 | H(52)-C(31)-C(32)-C(33) | -179.84 |
| C(31)-H(52) | 1.08 | 1.10 | H(56)-C(34)-H(55) | 107.53 | 109 | H(52)-C(31)-C(32)-H(53) | -0.80 |
| C(31)-C(32) | 1.39 | 1.42 | H(56)-C(34)-C(17) | 110.98 | 109.47 | C(29)-C(30)-C(31)-C(32) | 0.78 |
| C(30)-H(51) | 1.08 | 1.10 | H(55)-C(34)-C(17) | 111.04 | 109.47 | C(29)-C(30)-C(31)-H(52) | -179.59 |
| C(30)-C(31) | 1.40 | 1.42 | H(54)-C(33)-C(32) | 119.30 | 120 | H(51)-C(30)-C(31)-C(32) | -179.68 |
| C(29)-H(50) | 1.08 | 1.10 | H(54)-C(33)-C(28) | 120.76 | 120 | H(51)-C(30)-C(31)-H(52) | -0.06 |
| C(29)-C(30) | 1.39 | 1.42 | C(32)-C(33)-C(28) | 119.90 |  | C(28)-C(29)-C(30)-C(31) | -0.30 |
| C(28)-C(33) | 1.40 | 1.42 | H(53)-C(32)-C(33) | 119.07 | 120 | C(28)-C(29)-C(30)-H(51) | -179.84 |
| C(28)-C(29) | 1.40 | 1.42 | H(53)-C(32)-C(31) | 120.04 | 120 | H(50)-C(29)-C(30)-C(31) | 179.22 |
| N(27)-H(49) | 1.03 | 1.05 | C(33)-C(32)-C(31) | 120.88 |  | H(50)-C(29)-C(30)-H(51) | -0.32 |
| N(27)-C(28) | 1.41 | 1.46 | H(52)-C(31)-C(32) | 120.39 | 120 | N(27)-C(28)-C(33)-C(32) | 178.79 |
| C(25)-H(48) | 1.08 | 1.11 | H(52)-C(31)-C(30) | 120.40 | 120 | N(27)-C(28)-C(33)-H(54) | 1.09 |
| C(24)-H(47) | 1.08 | 1.11 | C(32)-C(31)-C(30) | 119.20 |  | C(29)-C(28)-C(33)-C(32) | 1.30 |
| C(24)-C(25) | 1.40 | 1.52 | H(51)-C(30)-C(31) | 120.17 | 120 | C(29)-C(28)-C(33)-H(54) | -176.40 |
| C(23)-O(35) | 1.37 | 1.39 | H(51)-C(30)-C(29) | 119.37 | 120 | N(27)-C(28)-C(29)-C(30) | -178.40 |
| C(23)-C(24) | 1.39 | 1.51 | C(31)-C(30)-C(29) | 120.46 |  | N(27)-C(28)-C(29)-H(50) | 2.07 |
| C(22)-H(46) | 1.08 | 1.10 | H(50)-C(29)-C(30) | 120.33 | 120 | C(33)-C(28)-C(29)-C(30) | -0.75 |
| C(22)-C(23) | 1.40 | 1.50 | H(50)-C(29)-C(28) | 119.27 | 120 | C(33)-C(28)-C(29)-H(50) | 179.73 |
| C(21)-H(45) | 1.09 | 1.10 | C(30)-C(29)-C(28) | 120.39 |  | C(3)-N(27)-C(28)-C(29) | -153.38 |
| C(21)-C(22) | 1.38 | 1.42 | C(33)-C(28)-C(29) | 119.15 | 120 | C(3)-N(27)-C(28)-C(33) | 29.08 |
| N(19)-H(44) | 1.01 | 1.05 | C(33)-C(28)-N(27) | 123.70 | 120 | H(49)-N(27)-C(28)-C(29) | 23.19 |
| N(19)-H(43) | 1.02 | 1.05 | C(29)-C(28)-N(27) | 117.10 | 120 | H(49)-N(27)-C(28)-C(33) | -154.34 |
| C(18)-N(19) | 1.39 | 1.46 | H(49)-N(27)-C(28) | 117.99 | 118 | C(23)-C(24)-C(25)-C(6) | 0.00 |
| C(17)-C(34) | 1.52 | 1.51 | H(49)-N(27)-C(3) | 110.49 | 118 | C(23)-C(24)-C(25)-H(48) | -179.60 |
| C(17)-O(26) | 1.22 | 1.21 | C(28)-N(27)-C(3) | 131.44 | 124 | H(47)-C(24)-C(25)-C(6) | 179.90 |
| C(16)-H(42) | 1.08 | 1.11 | H(48)-C(25)-C(24) | 120.02 | 109.41 | H(47)-C(24)-C(25)-H(48) | 0.30 |
| C(15)-H(41) | 1.08 | 1.11 | H(48)-C(25)-C(6) | 119.74 | 109.41 | C(22)-C(23)-O(35)-C(36) | 179.58 |
| C(15)-C(16) | 1.39 | 1.52 | C(24)-C(25)-C(6) | 120.24 | 109.5 | C(24)-C(23)-O(35)-C(36) | -0.29 |
| C(14)-C(17) | 1.49 | 1.51 | H(47)-C(24)-C(25) | 118.32 | 109.41 | C(22)-C(23)-C(24)-C(25) | 0.02 |
| C(14)-C(15) | 1.40 | 1.52 | H(47)-C(24)-C(23) | 120.97 | 109.41 | C(22)-C(23)-C(24)-H(47) | -179.88 |
| C(13)-H(40) | 1.08 | 1.10 | C(25)-C(24)-C(23) | 120.71 | 109.5 | O(35)-C(23)-C(24)-C(25) | 179.89 |
| C(13)-C(14) | 1.40 | 1.50 | O(35)-C(23)-C(24) | 124.96 | 107.7 | O(35)-C(23)-C(24)-H(47) | 0.00 |
| C(12)-H(39) | 1.09 | 1.10 | O(35)-C(23)-C(22) | 115.93 |  | C(21)-C(22)-C(23)-C(24) | 0.04 |
| C(12)-C(13) | 1.39 | 1.42 | C(24)-C(23)-C(22) | 119.11 | 109.51 | C(21)-C(22)-C(23)-O(35) | -179.84 |
| C(11)-C(16) | 1.41 | 1.52 | H(46)-C(22)-C(23) | 118.89 | 118.2 | H(46)-C(22)-C(23)-C(24) | 179.82 |
| C(11)-C(12) | 1.41 | 1.50 | H(46)-C(22)-C(21) | 120.90 | 120 | H(46)-C(22)-C(23)-O(35) | -0.06 |
| N(9)-H(38) | 1.01 | 1.02 | C(23)-C(22)-C(21) | 120.21 | 122 | C(6)-C(21)-C(22)-C(23) | -0.13 |
| N(9)-C(11) | 1.40 | 1.46 | H(45)-C(21)-C(22) | 119.26 | 120 | C(6)-C(21)-C(22)-H(46) | -179.90 |
| C(8)-O(10) | 1.24 | 1.21 | H(45)-C(21)-C(6) | 119.71 | 118.2 | H(45)-C(21)-C(22)-C(23) | 179.68 |
| C(8)-N(9) | 1.39 | 1.37 | C(22)-C(21)-C(6) | 121.03 | 122 | H(45)-C(21)-C(22)-H(46) | -0.09 |
| C(7)-C(18) | 1.39 | 1.42 | H(44)-N(19)-H(43) | 112.64 | 118.8 | C(2)-C(18)-N(19)-H(43) | 177.45 |
| C(7)-C(8) | 1.46 | 1.52 | H(44)-N(19)-C(18) | 114.11 |  | C(2)-C(18)-N(19)-H(44) | 48.69 |
| C(6)-C(25) | 1.40 | 1.52 | H(43)-N(19)-C(18) | 111.21 |  | C(7)-C(18)-N(19)-H(43) | -3.62 |
| C(6)-C(21) | 1.41 | 1.50 | N(19)-C(18)-C(7) | 122.98 | 120 | C(7)-C(18)-N(19)-H(44) | -132.38 |
| N(5)-H(37) | 1.01 | 1.02 | N(19)-C(18)-C(2) | 123.05 | 120 | C(14)-C(17)-C(34)-H(55) | -59.86 |
| N(5)-C(6) | 1.41 | 1.46 | C(7)-C(18)-C(2) | 113.96 | 120 | C(14)-C(17)-C(34)-H(56) | 59.68 |
| C(4)-O(20) | 1.24 | 1.21 | C(34)-C(17)-O(26) | 120.29 | 122.5 | C(14)-C(17)-C(34)-H(57) | 179.90 |
| C(4)-N(5) | 1.37 | 1.37 | C(34)-C(17)-C(14) | 118.82 | 116.6 | O(26)-C(17)-C(34)-H(55) | 120.22 |
| C(3)-N(27) | 1.35 | 1.46 | O(26)-C(17)-C(14) | 120.89 | 122.5 | O(26)-C(17)-C(34)-H(56) | -120.24 |
| C(2)-C(18) | 1.44 | 1.42 | H(42)-C(16)-C(15) | 121.01 | 109.41 | O(26)-C(17)-C(34)-H(57) | -0.02 |
| C(2)-C(4) | 1.48 | 1.52 | H(42)-C(16)-C(11) | 119.56 | 109.41 | C(14)-C(15)-C(16)-C(11) | -0.04 |
| C(2)-C(3) | 1.41 | 1.42 | C(15)-C(16)-C(11) | 119.43 | 109.5 | C(14)-C(15)-C(16)-H(42) | 179.85 |
| S(1)-C(7) | 1.77 | 1.66 | H(41)-C(15)-C(16) | 119.87 | 109.41 | H(41)-C(15)-C(16)-C(11) | 179.98 |
| S(1)-C(3) | 1.75 | 1.66 | H(41)-C(15)-C(14) | 118.12 | 109.41 | H(41)-C(15)-C(16)-H(42) | -0.12 |
|  |  |  | C(16)-C(15)-C(14) | 122.01 | 109.5 | C(13)-C(14)-C(17)-O(26) | -179.56 |
|  |  |  | C(17)-C(14)-C(15) | 119.00 | 109.9 | C(13)-C(14)-C(17)-C(34) | 0.52 |
|  |  |  | C(17)-C(14)-C(13) | 122.98 | 110.51 | C(15)-C(14)-C(17)-O(26) | 0.49 |
|  |  |  | C(15)-C(14)-C(13) | 118.03 | 109.51 | C(15)-C(14)-C(17)-C(34) | -179.43 |
|  |  |  | H(40)-C(13)-C(14) | 120.63 | 118.2 | C(13)-C(14)-C(15)-C(16) | 0.01 |
|  |  |  | H(40)-C(13)-C(12) | 118.63 | 120 | C(13)-C(14)-C(15)-H(41) | 179.98 |
|  |  |  | C(14)-C(13)-C(12) | 120.74 | 122 | C(17)-C(14)-C(15)-C(16) | 179.96 |
|  |  |  | H(39)-C(12)-C(13) | 119.63 | 120 | C(17)-C(14)-C(15)-H(41) | -0.07 |
|  |  |  | H(39)-C(12)-C(11) | 119.64 | 118.2 | C(12)-C(13)-C(14)-C(15) | 0.01 |
|  |  |  | C(13)-C(12)-C(11) | 120.73 | 122 | C(12)-C(13)-C(14)-C(17) | -179.94 |
|  |  |  | C(16)-C(11)-C(12) | 119.05 | 109.51 | H(40)-C(13)-C(14)-C(15) | 179.95 |
|  |  |  | C(16)-C(11)-N(9) | 124.06 | 110.78 | H(40)-C(13)-C(14)-C(17) | 0.00 |
|  |  |  | C(12)-C(11)-N(9) | 116.88 |  | C(11)-C(12)-C(13)-C(14) | 0.01 |
|  |  |  | H(38)-N(9)-C(11) | 114.07 | 118 | C(11)-C(12)-C(13)-H(40) | -179.93 |
|  |  |  | H(38)-N(9)-C(8) | 116.53 | 117.4 | H(39)-C(12)-C(13)-C(14) | 179.89 |
|  |  |  | C(11)-N(9)-C(8) | 129.39 |  | H(39)-C(12)-C(13)-H(40) | -0.05 |
|  |  |  | O(10)-C(8)-N(9) | 122.03 | 122.6 | N(9)-C(11)-C(16)-C(15) | -179.72 |
|  |  |  | O(10)-C(8)-C(7) | 121.57 | 123 | N(9)-C(11)-C(16)-H(42) | 0.39 |
|  |  |  | N(9)-C(8)-C(7) | 116.40 | 112.74 | C(12)-C(11)-C(16)-C(15) | 0.07 |
|  |  |  | C(18)-C(7)-C(8) | 125.11 | 117.6 | C(12)-C(11)-C(16)-H(42) | -179.83 |
|  |  |  | C(18)-C(7)-S(1) | 110.58 | 119 | N(9)-C(11)-C(12)-C(13) | 179.75 |
|  |  |  | C(8)-C(7)-S(1) | 124.07 |  | N(9)-C(11)-C(12)-H(39) | -0.13 |
|  |  |  | C(25)-C(6)-C(21) | 118.71 | 109.51 | C(16)-C(11)-C(12)-C(13) | -0.05 |
|  |  |  | C(25)-C(6)-N(5) | 124.42 | 110.78 | C(16)-C(11)-C(12)-H(39) | -179.93 |
|  |  |  | C(21)-C(6)-N(5) | 116.86 |  | C(8)-N(9)-C(11)-C(12) | 179.67 |
|  |  |  | H(37)-N(5)-C(6) | 115.23 | 118 | C(8)-N(9)-C(11)-C(16) | -0.54 |
|  |  |  | H(37)-N(5)-C(4) | 115.47 | 117.4 | H(38)-N(9)-C(11)-C(12) | 1.01 |
|  |  |  | C(6)-N(5)-C(4) | 128.91 |  | H(38)-N(9)-C(11)-C(16) | -179.20 |
|  |  |  | O(20)-C(4)-N(5) | 122.43 | 122.6 | C(7)-C(8)-N(9)-C(11) | 177.83 |
|  |  |  | O(20)-C(4)-C(2) | 121.22 | 123 | C(7)-C(8)-N(9)-H(38) | -3.54 |
|  |  |  | N(5)-C(4)-C(2) | 116.31 | 112.74 | O(10)-C(8)-N(9)-C(11) | -2.26 |
|  |  |  | N(27)-C(3)-C(2) | 124.17 | 120 | O(10)-C(8)-N(9)-H(38) | 176.37 |
|  |  |  | N(27)-C(3)-S(1) | 124.02 |  | S(1)-C(7)-C(18)-C(2) | -1.09 |
|  |  |  | C(2)-C(3)-S(1) | 111.66 | 119 | S(1)-C(7)-C(18)-N(19) | 179.89 |
|  |  |  | C(18)-C(2)-C(4) | 127.86 | 117.6 | C(8)-C(7)-C(18)-C(2) | -175.64 |
|  |  |  | C(18)-C(2)-C(3) | 111.89 | 120 | C(8)-C(7)-C(18)-N(19) | 5.34 |
|  |  |  | C(4)-C(2)-C(3) | 119.87 | 117.6 | S(1)-C(7)-C(8)-N(9) | -1.44 |
|  |  |  | C(7)-S(1)-C(3) | 91.75 | 98.5 | S(1)-C(7)-C(8)-O(10) | 178.66 |
|  |  |  |  |  |  | C(18)-C(7)-C(8)-N(9) | 172.40 |
|  |  |  |  |  |  | C(18)-C(7)-C(8)-O(10) | -7.50 |
|  |  |  |  |  |  | N(5)-C(6)-C(25)-C(24) | 178.85 |
|  |  |  |  |  |  | N(5)-C(6)-C(25)-H(48) | -1.55 |
|  |  |  |  |  |  | C(21)-C(6)-C(25)-C(24) | -0.09 |
|  |  |  |  |  |  | C(21)-C(6)-C(25)-H(48) | 179.51 |
|  |  |  |  |  |  | N(5)-C(6)-C(21)-C(22) | -178.86 |
|  |  |  |  |  |  | N(5)-C(6)-C(21)-H(45) | 1.33 |
|  |  |  |  |  |  | C(25)-C(6)-C(21)-C(22) | 0.15 |
|  |  |  |  |  |  | C(25)-C(6)-C(21)-H(45) | -179.66 |
|  |  |  |  |  |  | C(4)-N(5)-C(6)-C(21) | -172.25 |
|  |  |  |  |  |  | C(4)-N(5)-C(6)-C(25) | 8.80 |
|  |  |  |  |  |  | H(37)-N(5)-C(6)-C(21) | 0.25 |
|  |  |  |  |  |  | H(37)-N(5)-C(6)-C(25) | -178.70 |
|  |  |  |  |  |  | C(2)-C(4)-N(5)-C(6) | 178.27 |
|  |  |  |  |  |  | C(2)-C(4)-N(5)-H(37) | 5.79 |
|  |  |  |  |  |  | O(20)-C(4)-N(5)-C(6) | 0.71 |
|  |  |  |  |  |  | O(20)-C(4)-N(5)-H(37) | -171.77 |
|  |  |  |  |  |  | S(1)-C(3)-N(27)-C(28) | 1.52 |
|  |  |  |  |  |  | S(1)-C(3)-N(27)-H(49) | -175.25 |
|  |  |  |  |  |  | C(2)-C(3)-N(27)-C(28) | 176.66 |
|  |  |  |  |  |  | C(2)-C(3)-N(27)-H(49) | -0.11 |
|  |  |  |  |  |  | C(3)-C(2)-C(18)-C(7) | 3.61 |
|  |  |  |  |  |  | C(3)-C(2)-C(18)-N(19) | -177.38 |
|  |  |  |  |  |  | C(4)-C(2)-C(18)-C(7) | -169.20 |
|  |  |  |  |  |  | C(4)-C(2)-C(18)-N(19) | 9.82 |
|  |  |  |  |  |  | C(3)-C(2)-C(4)-N(5) | -160.61 |
|  |  |  |  |  |  | C(3)-C(2)-C(4)-O(20) | 16.98 |
|  |  |  |  |  |  | C(18)-C(2)-C(4)-N(5) | 11.69 |
|  |  |  |  |  |  | C(18)-C(2)-C(4)-O(20) | -170.72 |
|  |  |  |  |  |  | C(4)-C(2)-C(3)-S(1) | 168.99 |
|  |  |  |  |  |  | C(4)-C(2)-C(3)-N(27) | -6.68 |
|  |  |  |  |  |  | C(18)-C(2)-C(3)-S(1) | -4.47 |
|  |  |  |  |  |  | C(18)-C(2)-C(3)-N(27) | 179.87 |
|  |  |  |  |  |  | C(3)-S(1)-C(7)-C(8) | 173.38 |
|  |  |  |  |  |  | C(3)-S(1)-C(7)-C(18) | -1.24 |
|  |  |  |  |  |  | C(7)-S(1)-C(3)-C(2) | 3.29 |
|  |  |  |  |  |  | C(7)-S(1)-C(3)-N(27) | 178.97 |

| **7c** |  |  |  |  |  |  |  |  |
| --- | --- | --- | --- | --- | --- | --- | --- | --- |
| **Atoms** | **Actual (°/A°)** | **Optimal (°/A°)** |  | **Atoms** | **Actual (°/A°)** | **Optimal (°/A°)** | **Atoms** | **Actual (°/A°)** |
| C(35)-H(56) | 1.08 | 1.10 |  | H(56)-C(35)-C(34) | 119.39 | 120 | C(33)-C(34)-C(35)-C(30) | -0.91 |
| C(34)-H(55) | 1.08 | 1.10 |  | H(56)-C(35)-C(30) | 120.72 | 120 | C(33)-C(34)-C(35)-H(56) | 176.82 |
| C(34)-C(35) | 1.39 | 1.42 |  | C(34)-C(35)-C(30) | 119.85 |  | H(55)-C(34)-C(35)-C(30) | -179.88 |
| C(33)-H(54) | 1.08 | 1.10 |  | H(55)-C(34)-C(35) | 119.08 | 120 | H(55)-C(34)-C(35)-H(56) | -2.15 |
| C(33)-C(34) | 1.39 | 1.42 |  | H(55)-C(34)-C(33) | 120.06 | 120 | C(32)-C(33)-C(34)-C(35) | -0.24 |
| C(32)-H(53) | 1.08 | 1.10 |  | C(35)-C(34)-C(33) | 120.86 |  | C(32)-C(33)-C(34)-H(55) | 178.72 |
| C(32)-C(33) | 1.40 | 1.42 |  | H(54)-C(33)-C(34) | 120.38 | 120 | H(54)-C(33)-C(34)-C(35) | -179.77 |
| C(31)-H(52) | 1.08 | 1.10 |  | H(54)-C(33)-C(32) | 120.37 | 120 | H(54)-C(33)-C(34)-H(55) | -0.81 |
| C(31)-C(32) | 1.39 | 1.42 |  | C(34)-C(33)-C(32) | 119.25 |  | C(31)-C(32)-C(33)-C(34) | 0.86 |
| C(30)-C(35) | 1.40 | 1.42 |  | H(53)-C(32)-C(33) | 120.17 | 120 | C(31)-C(32)-C(33)-H(54) | -179.61 |
| C(30)-C(31) | 1.40 | 1.42 |  | H(53)-C(32)-C(31) | 119.40 | 120 | H(53)-C(32)-C(33)-C(34) | -179.59 |
| N(29)-H(51) | 1.02 | 1.05 |  | C(33)-C(32)-C(31) | 120.43 |  | H(53)-C(32)-C(33)-H(54) | -0.05 |
| N(29)-C(30) | 1.41 | 1.46 |  | H(52)-C(31)-C(32) | 120.31 | 120 | C(30)-C(31)-C(32)-C(33) | -0.34 |
| C(28)-H(50) | 1.09 | 1.11 |  | H(52)-C(31)-C(30) | 119.34 | 120 | C(30)-C(31)-C(32)-H(53) | -179.89 |
| C(28)-H(49) | 1.09 | 1.11 |  | C(32)-C(31)-C(30) | 120.35 |  | H(52)-C(31)-C(32)-C(33) | 179.25 |
| C(28)-H(48) | 1.09 | 1.11 |  | C(35)-C(30)-C(31) | 119.25 | 120 | H(52)-C(31)-C(32)-H(53) | -0.31 |
| C(25)-H(47) | 1.08 | 1.10 |  | C(35)-C(30)-N(29) | 123.53 | 120 | N(29)-C(30)-C(35)-C(34) | 179.20 |
| C(24)-H(46) | 1.08 | 1.10 |  | C(31)-C(30)-N(29) | 117.18 | 120 | N(29)-C(30)-C(35)-H(56) | 1.50 |
| C(24)-C(25) | 1.39 | 1.42 |  | H(51)-N(29)-C(30) | 117.95 | 118 | C(31)-C(30)-C(35)-C(34) | 1.43 |
| C(23)-Cl(26) | 1.76 | 1.72 |  | H(51)-N(29)-C(3) | 110.86 | 118 | C(31)-C(30)-C(35)-H(56) | -176.28 |
| C(23)-C(24) | 1.39 | 1.42 |  | C(30)-N(29)-C(3) | 131.08 | 124 | N(29)-C(30)-C(31)-C(32) | -178.73 |
| C(22)-H(45) | 1.08 | 1.10 |  | H(50)-C(28)-H(49) | 109.30 | 109 | N(29)-C(30)-C(31)-H(52) | 1.68 |
| C(22)-C(23) | 1.39 | 1.42 |  | H(50)-C(28)-H(48) | 107.52 | 109 | C(35)-C(30)-C(31)-C(32) | -0.81 |
| C(21)-H(44) | 1.09 | 1.10 |  | H(50)-C(28)-C(17) | 111.01 | 109.47 | C(35)-C(30)-C(31)-H(52) | 179.60 |
| C(21)-C(22) | 1.39 | 1.42 |  | H(49)-C(28)-H(48) | 109.31 | 109 | C(3)-N(29)-C(30)-C(31) | -152.12 |
| N(19)-H(43) | 1.01 | 1.05 |  | H(49)-C(28)-C(17) | 108.67 | 109.47 | C(3)-N(29)-C(30)-C(35) | 30.06 |
| N(19)-H(42) | 1.02 | 1.05 |  | H(48)-C(28)-C(17) | 111.01 | 109.47 | H(51)-N(29)-C(30)-C(31) | 23.73 |
| C(18)-N(19) | 1.39 | 1.46 |  | H(47)-C(25)-C(24) | 120.33 | 120 | H(51)-N(29)-C(30)-C(35) | -154.09 |
| C(17)-C(28) | 1.52 | 1.51 |  | H(47)-C(25)-C(6) | 119.81 | 120 | C(23)-C(24)-C(25)-C(6) | -0.08 |
| C(17)-O(27) | 1.22 | 1.21 |  | C(24)-C(25)-C(6) | 119.86 |  | C(23)-C(24)-C(25)-H(47) | 179.54 |
| C(16)-H(41) | 1.08 | 1.11 |  | H(46)-C(24)-C(25) | 119.75 | 120 | H(46)-C(24)-C(25)-C(6) | -179.99 |
| C(15)-H(40) | 1.08 | 1.11 |  | H(46)-C(24)-C(23) | 119.99 | 120 | H(46)-C(24)-C(25)-H(47) | -0.37 |
| C(15)-C(16) | 1.39 | 1.52 |  | C(25)-C(24)-C(23) | 120.26 |  | C(22)-C(23)-C(24)-C(25) | -0.09 |
| C(14)-C(17) | 1.49 | 1.51 |  | Cl(26)-C(23)-C(24) | 119.84 | 118.8 | C(22)-C(23)-C(24)-H(46) | 179.82 |
| C(14)-C(15) | 1.40 | 1.52 |  | Cl(26)-C(23)-C(22) | 119.60 | 118.8 | Cl(26)-C(23)-C(24)-C(25) | -179.98 |
| C(13)-H(39) | 1.08 | 1.10 |  | C(24)-C(23)-C(22) | 120.56 | 120 | Cl(26)-C(23)-C(24)-H(46) | -0.07 |
| C(13)-C(14) | 1.40 | 1.50 |  | H(45)-C(22)-C(23) | 120.36 | 120 | C(21)-C(22)-C(23)-C(24) | 0.10 |
| C(12)-H(38) | 1.09 | 1.10 |  | H(45)-C(22)-C(21) | 120.38 | 120 | C(21)-C(22)-C(23)-Cl(26) | 179.99 |
| C(12)-C(13) | 1.39 | 1.42 |  | C(23)-C(22)-C(21) | 119.27 |  | H(45)-C(22)-C(23)-C(24) | -179.78 |
| C(11)-C(16) | 1.41 | 1.52 |  | H(44)-C(21)-C(22) | 119.22 | 120 | H(45)-C(22)-C(23)-Cl(26) | 0.11 |
| C(11)-C(12) | 1.40 | 1.50 |  | H(44)-C(21)-C(6) | 119.80 | 120 | C(6)-C(21)-C(22)-C(23) | 0.06 |
| N(9)-H(37) | 1.01 | 1.02 |  | C(22)-C(21)-C(6) | 120.97 |  | C(6)-C(21)-C(22)-H(45) | 179.94 |
| N(9)-C(11) | 1.40 | 1.46 |  | H(43)-N(19)-H(42) | 112.63 | 118.8 | H(44)-C(21)-C(22)-C(23) | -179.78 |
| C(8)-O(10) | 1.24 | 1.21 |  | H(43)-N(19)-C(18) | 114.25 |  | H(44)-C(21)-C(22)-H(45) | 0.10 |
| C(8)-N(9) | 1.39 | 1.37 |  | H(42)-N(19)-C(18) | 111.18 |  | C(2)-C(18)-N(19)-H(42) | -178.30 |
| C(7)-C(18) | 1.39 | 1.42 |  | N(19)-C(18)-C(7) | 123.10 | 120 | C(2)-C(18)-N(19)-H(43) | -49.46 |
| C(7)-C(8) | 1.46 | 1.52 |  | N(19)-C(18)-C(2) | 123.05 | 120 | C(7)-C(18)-N(19)-H(42) | 2.98 |
| C(6)-C(25) | 1.40 | 1.42 |  | C(7)-C(18)-C(2) | 113.83 | 120 | C(7)-C(18)-N(19)-H(43) | 131.83 |
| C(6)-C(21) | 1.40 | 1.42 |  | C(28)-C(17)-O(27) | 120.32 | 122.5 | C(14)-C(17)-C(28)-H(48) | 59.69 |
| N(5)-H(36) | 1.01 | 1.01 |  | C(28)-C(17)-C(14) | 118.82 | 116.6 | C(14)-C(17)-C(28)-H(49) | 179.93 |
| N(5)-C(6) | 1.41 | 1.35 |  | O(27)-C(17)-C(14) | 120.86 | 122.5 | C(14)-C(17)-C(28)-H(50) | -59.84 |
| C(4)-O(20) | 1.24 | 1.21 |  | H(41)-C(16)-C(15) | 120.97 | 109.41 | O(27)-C(17)-C(28)-H(48) | -120.32 |
| C(4)-N(5) | 1.37 | 1.37 |  | H(41)-C(16)-C(11) | 119.59 | 109.41 | O(27)-C(17)-C(28)-H(49) | -0.08 |
| C(3)-N(29) | 1.35 | 1.46 |  | C(15)-C(16)-C(11) | 119.43 | 109.5 | O(27)-C(17)-C(28)-H(50) | 120.15 |
| C(2)-C(18) | 1.44 | 1.42 |  | H(40)-C(15)-C(16) | 119.89 | 109.41 | C(14)-C(15)-C(16)-C(11) | 0.11 |
| C(2)-C(4) | 1.48 | 1.52 |  | H(40)-C(15)-C(14) | 118.13 | 109.41 | C(14)-C(15)-C(16)-H(41) | -179.63 |
| C(2)-C(3) | 1.40 | 1.42 |  | C(16)-C(15)-C(14) | 121.99 | 109.5 | H(40)-C(15)-C(16)-C(11) | -179.96 |
| S(1)-C(7) | 1.77 | 1.66 |  | C(17)-C(14)-C(15) | 118.96 | 109.9 | H(40)-C(15)-C(16)-H(41) | 0.30 |
| S(1)-C(3) | 1.75 | 1.66 |  | C(17)-C(14)-C(13) | 122.98 | 110.51 | C(13)-C(14)-C(17)-O(27) | -179.99 |
|  |  |  |  | C(15)-C(14)-C(13) | 118.05 | 109.51 | C(13)-C(14)-C(17)-C(28) | 0.00 |
|  |  |  |  | H(39)-C(13)-C(14) | 120.62 | 118.2 | C(15)-C(14)-C(17)-O(27) | 0.01 |
|  |  |  |  | H(39)-C(13)-C(12) | 118.65 | 120 | C(15)-C(14)-C(17)-C(28) | -180.00 |
|  |  |  |  | C(14)-C(13)-C(12) | 120.73 | 122 | C(13)-C(14)-C(15)-C(16) | -0.06 |
|  |  |  |  | H(38)-C(12)-C(13) | 119.63 | 120 | C(13)-C(14)-C(15)-H(40) | -179.99 |
|  |  |  |  | H(38)-C(12)-C(11) | 119.66 | 118.2 | C(17)-C(14)-C(15)-C(16) | 179.94 |
|  |  |  |  | C(13)-C(12)-C(11) | 120.72 | 122 | C(17)-C(14)-C(15)-H(40) | 0.01 |
|  |  |  |  | C(16)-C(11)-C(12) | 119.08 | 109.51 | C(12)-C(13)-C(14)-C(15) | -0.02 |
|  |  |  |  | C(16)-C(11)-N(9) | 124.01 | 110.78 | C(12)-C(13)-C(14)-C(17) | 179.98 |
|  |  |  |  | C(12)-C(11)-N(9) | 116.90 |  | H(39)-C(13)-C(14)-C(15) | 179.97 |
|  |  |  |  | H(37)-N(9)-C(11) | 114.12 | 118 | H(39)-C(13)-C(14)-C(17) | -0.03 |
|  |  |  |  | H(37)-N(9)-C(8) | 116.49 | 117.4 | C(11)-C(12)-C(13)-C(14) | 0.04 |
|  |  |  |  | C(11)-N(9)-C(8) | 129.31 |  | C(11)-C(12)-C(13)-H(39) | -179.95 |
|  |  |  |  | O(10)-C(8)-N(9) | 122.15 | 122.6 | H(38)-C(12)-C(13)-C(14) | -179.96 |
|  |  |  |  | O(10)-C(8)-C(7) | 121.48 | 123 | H(38)-C(12)-C(13)-H(39) | 0.05 |
|  |  |  |  | N(9)-C(8)-C(7) | 116.36 | 112.74 | N(9)-C(11)-C(16)-C(15) | 179.52 |
|  |  |  |  | C(18)-C(7)-C(8) | 125.08 | 117.6 | N(9)-C(11)-C(16)-H(41) | -0.74 |
|  |  |  |  | C(18)-C(7)-S(1) | 110.68 | 119 | C(12)-C(11)-C(16)-C(15) | -0.08 |
|  |  |  |  | C(8)-C(7)-S(1) | 124.11 |  | C(12)-C(11)-C(16)-H(41) | 179.66 |
|  |  |  |  | C(25)-C(6)-C(21) | 119.08 | 120 | N(9)-C(11)-C(12)-C(13) | -179.62 |
|  |  |  |  | C(25)-C(6)-N(5) | 124.08 | 120 | N(9)-C(11)-C(12)-H(38) | 0.39 |
|  |  |  |  | C(21)-C(6)-N(5) | 116.84 | 120 | C(16)-C(11)-C(12)-C(13) | 0.01 |
|  |  |  |  | H(36)-N(5)-C(6) | 115.40 | 110 | C(16)-C(11)-C(12)-H(38) | -179.98 |
|  |  |  |  | H(36)-N(5)-C(4) | 115.34 | 117.4 | C(8)-N(9)-C(11)-C(12) | -177.51 |
|  |  |  |  | C(6)-N(5)-C(4) | 129.06 |  | C(8)-N(9)-C(11)-C(16) | 2.88 |
|  |  |  |  | O(20)-C(4)-N(5) | 122.56 | 122.6 | H(37)-N(9)-C(11)-C(12) | -0.85 |
|  |  |  |  | O(20)-C(4)-C(2) | 121.43 | 123 | H(37)-N(9)-C(11)-C(16) | 179.54 |
|  |  |  |  | N(5)-C(4)-C(2) | 115.98 | 112.74 | C(7)-C(8)-N(9)-C(11) | -177.90 |
|  |  |  |  | N(29)-C(3)-C(2) | 124.31 | 120 | C(7)-C(8)-N(9)-H(37) | 5.50 |
|  |  |  |  | N(29)-C(3)-S(1) | 124.03 |  | O(10)-C(8)-N(9)-C(11) | 2.52 |
|  |  |  |  | C(2)-C(3)-S(1) | 111.66 | 119 | O(10)-C(8)-N(9)-H(37) | -174.08 |
|  |  |  |  | C(18)-C(2)-C(4) | 127.91 | 117.6 | S(1)-C(7)-C(18)-C(2) | 0.45 |
|  |  |  |  | C(18)-C(2)-C(3) | 112.02 | 120 | S(1)-C(7)-C(18)-N(19) | 179.27 |
|  |  |  |  | C(4)-C(2)-C(3) | 119.98 | 117.6 | C(8)-C(7)-C(18)-C(2) | 176.32 |
|  |  |  |  | C(7)-S(1)-C(3) | 91.67 | 98.5 | C(8)-C(7)-C(18)-N(19) | -4.86 |
|  |  |  |  |  |  |  | S(1)-C(7)-C(8)-N(9) | 4.55 |
|  |  |  |  |  |  |  | S(1)-C(7)-C(8)-O(10) | -175.87 |
|  |  |  |  |  |  |  | C(18)-C(7)-C(8)-N(9) | -170.78 |
|  |  |  |  |  |  |  | C(18)-C(7)-C(8)-O(10) | 8.81 |
|  |  |  |  |  |  |  | N(5)-C(6)-C(25)-C(24) | -179.00 |
|  |  |  |  |  |  |  | N(5)-C(6)-C(25)-H(47) | 1.37 |
|  |  |  |  |  |  |  | C(21)-C(6)-C(25)-C(24) | 0.24 |
|  |  |  |  |  |  |  | C(21)-C(6)-C(25)-H(47) | -179.38 |
|  |  |  |  |  |  |  | N(5)-C(6)-C(21)-C(22) | 179.07 |
|  |  |  |  |  |  |  | N(5)-C(6)-C(21)-H(44) | -1.09 |
|  |  |  |  |  |  |  | C(25)-C(6)-C(21)-C(22) | -0.23 |
|  |  |  |  |  |  |  | C(25)-C(6)-C(21)-H(44) | 179.61 |
|  |  |  |  |  |  |  | C(4)-N(5)-C(6)-C(21) | 174.25 |
|  |  |  |  |  |  |  | C(4)-N(5)-C(6)-C(25) | -6.48 |
|  |  |  |  |  |  |  | H(36)-N(5)-C(6)-C(21) | -0.35 |
|  |  |  |  |  |  |  | H(36)-N(5)-C(6)-C(25) | 178.91 |
|  |  |  |  |  |  |  | C(2)-C(4)-N(5)-C(6) | -179.21 |
|  |  |  |  |  |  |  | C(2)-C(4)-N(5)-H(36) | -4.60 |
|  |  |  |  |  |  |  | O(20)-C(4)-N(5)-C(6) | -1.38 |
|  |  |  |  |  |  |  | O(20)-C(4)-N(5)-H(36) | 173.23 |
|  |  |  |  |  |  |  | S(1)-C(3)-N(29)-C(30) | 3.20 |
|  |  |  |  |  |  |  | S(1)-C(3)-N(29)-H(51) | -172.88 |
|  |  |  |  |  |  |  | C(2)-C(3)-N(29)-C(30) | -177.06 |
|  |  |  |  |  |  |  | C(2)-C(3)-N(29)-H(51) | 6.86 |
|  |  |  |  |  |  |  | C(3)-C(2)-C(18)-C(7) | -2.92 |
|  |  |  |  |  |  |  | C(3)-C(2)-C(18)-N(19) | 178.26 |
|  |  |  |  |  |  |  | C(4)-C(2)-C(18)-C(7) | 173.65 |
|  |  |  |  |  |  |  | C(4)-C(2)-C(18)-N(19) | -5.17 |
|  |  |  |  |  |  |  | C(3)-C(2)-C(4)-N(5) | 158.74 |
|  |  |  |  |  |  |  | C(3)-C(2)-C(4)-O(20) | -19.12 |
|  |  |  |  |  |  |  | C(18)-C(2)-C(4)-N(5) | -17.59 |
|  |  |  |  |  |  |  | C(18)-C(2)-C(4)-O(20) | 164.55 |
|  |  |  |  |  |  |  | C(4)-C(2)-C(3)-S(1) | -172.83 |
|  |  |  |  |  |  |  | C(4)-C(2)-C(3)-N(29) | 7.40 |
|  |  |  |  |  |  |  | C(18)-C(2)-C(3)-S(1) | 4.05 |
|  |  |  |  |  |  |  | C(18)-C(2)-C(3)-N(29) | -175.72 |
|  |  |  |  |  |  |  | C(3)-S(1)-C(7)-C(8) | -174.34 |
|  |  |  |  |  |  |  | C(3)-S(1)-C(7)-C(18) | 1.57 |
|  |  |  |  |  |  |  | C(7)-S(1)-C(3)-C(2) | -3.23 |
|  |  |  |  |  |  |  | C(7)-S(1)-C(3)-N(29) | 176.54 |

**Table S4** The atomic Mulliken’s charges and Fukui’s indices of investigated compounds

|  | **3a** |  |  |  | **3b** |  |  |  | **3c** |  |  |
| --- | --- | --- | --- | --- | --- | --- | --- | --- | --- | --- | --- |
| **Atom** | **charge** | $\boldsymbol{f}_{\boldsymbol{k}}^{\boldsymbol{+}}$ | $\boldsymbol{f}_{\boldsymbol{k}}^{\boldsymbol{-}}$ | **Atom** | **charge** | $\boldsymbol{f}_{\boldsymbol{k}}^{\boldsymbol{+}}$ | $\boldsymbol{f}_{\boldsymbol{k}}^{\boldsymbol{-}}$ | **Atom** | **charge** | $\boldsymbol{f}_{\boldsymbol{k}}^{\boldsymbol{+}}$ | $\boldsymbol{f}_{\boldsymbol{k}}^{\boldsymbol{-}}$ |
| **S** | -0.885 | 0.083 | 0.069 | **S** | -0.901 | 0.081 | 0.065 | **S** | -0.860 | 0.083 | 0.069 |
| **Th^2^** | 0.673 | 0.004 | 0.052 | **Th^2^** | 0.651 | 0.003 | 0.044 | **Th^2^** | 0.729 | 0.005 | 0.052 |
| **Th^3^** | -0.651 | 0.022 | 0.014 | **Th^3^** | -0.602 | 0.022 | 0.012 | **Th^3^** | -0.618 | 0.022 | 0.014 |
| **Th^4^** | -0.439 | -0.006 | 0.04 | **Th^4^** | -0.440 | -0.006 | 0.039 | **Th^4^** | -0.436 | -0.005 | 0.038 |
| **Th^5^** | 0.273 | 0.042 | 0.013 | **Th^5^** | 0.260 | 0.041 | 0.016 | **Th^5^** | 0.227 | 0.042 | 0.013 |
| **N_azo(1)_** | -0.299 | 0.085 | 0.004 | **N_azo(1)_** | -0.313 | 0.082 | 0.006 | **N_azo(1)_** | -0.265 | 0.084 | 0.006 |
| **N_azo(2)_** | 0.317 | 0.09 | 0.034 | **N_azo(2)_** | 0.325 | 0.09 | 0.03 | **N_azo(2)_** | 0.275 | 0.086 | 0.037 |
| **C_Ph(Azo)_** | -0.099 | 0.001 | 0.004 | **C_Ph(Azo)_** | -0.125 | -0.001 | 0.012 | **C_Ph(Azo)_** | -0.417 | 0.002 | 0.003 |
| **C_O_ _(amide Th_^2^_)_** | 0.393 | 0.019 | 0.016 | **C_O_ _(amide Th_^2^_)_** | 0.364 | 0.019 | 0.017 | **C_O_ _(amide Th_^2^_)_** | 0.371 | 0.018 | 0.016 |
| **NH_(amide Th_^2^_)_** | 0.138 | 0.001 | 0.013 | **NH_(amide Th_^2^_)_** | 0.138 | 0 | 0.011 | **NH_(amide Th_^2^_)_** | 0.132 | 0.001 | 0.014 |
| **O_(amide Th_^2^_)_** | -0.337 | 0.021 | 0.033 | **O_(amide Th_^2^_)_** | -0.339 | 0.021 | 0.031 | **O_(amide Th_^2^_)_** | -0.337 | 0.021 | 0.032 |
| **C_Ph(amide Th_^2^_)_** | -0.152 | -0.003 | -0.004 | **C_Ph(amide Th_^2^_)_** | -0.169 | -0.003 | -0.004 | **C_Ph(amide Th_^2^_)_** | -0.169 | -0.004 | -0.004 |
| **C4_Ph(amide Th_^2^_)_** | 1.515 | 0.009 | 0.014 | **C4_Ph(amide Th_^2^_)_** | 1.518 | 0.008 | 0.012 | **C4_Ph(amide Th_^2^_)_** | 1.501 | 0.014 | 0.008 |
| **C_oacyl_** | -0.429 | 0.008 | 0.009 | **C_Oacyl_** | -0.430 | 0.009 | 0.008 | **C_Oacyl_** | -0.416 | 0.007 | 0.009 |
| **OH_(Th)_** | -0.226 | 0.007 | 0.023 | **OH_(Th)_** | -0.225 | 0.007 | 0.018 | **OH_(Th)_** | -0.226 | 0.007 | 0.023 |
| **C4_Ph(Azo)_** | 0.488 | 0.032 | 0.019 | **C4_Ph(Azo)_** | -0.541 | 0.041 | 0.026 | **C4_Ph(Azo)_** | 0.203 | 0.024 | 0.012 |
| **C_MeCO_** | -0.456 | -0.004 | -0.006 | **C_MeCO_** | -0.456 | -0.005 | -0.005 | **C_MeCO_** | -0.457 | -0.004 | -0.006 |
| **O_COMe_** | -0.252 | 0.019 | 0.025 | **O _COCH3_** | -0.253 | 0.02 | 0.023 | **O _COCH3_** | -0.251 | 0.018 | 0.025 |
| **NH_Th_** | 0.211 | 0.011 | 0.047 | **NH_Th_** | 0.221 | 0.01 | 0.045 | **NH_Th_** | 0.204 | 0.013 | 0.046 |
| **C_Ph(NH)_** | -0.214 | -0.003 | -0.002 | **C_Ph(NH)_** | -0.204 | -0.002 | -0.003 | **C_Ph(NH)_** | -0.236 | -0.004 | -0.002 |
| **C_MeAzo_** | -0.529 | -0.014 | -0.009 | **O_methoxy_** | -0.160 | 0.027 | 0.031 | **Cl** | 0.460 | 0.092 | 0.071 |
|  |  |  |  | **C_methoxy_** | -0.325 | -0.021 | -0.017 |  |  |  |  |

|  | **5a** |  |  |  | **5b** |  |  |  | **5c** |  |  |
| --- | --- | --- | --- | --- | --- | --- | --- | --- | --- | --- | --- |
| **Atom** | **charge** | $\boldsymbol{f}_{\boldsymbol{k}}^{\boldsymbol{+}}$ | $\boldsymbol{f}_{\boldsymbol{k}}^{\boldsymbol{-}}$ | **Atom** | **charge** | $\boldsymbol{f}_{\boldsymbol{k}}^{\boldsymbol{+}}$ | $\boldsymbol{f}_{\boldsymbol{k}}^{\boldsymbol{-}}$ | **Atom** | **charge** | $\boldsymbol{f}_{\boldsymbol{k}}^{\boldsymbol{+}}$ | $\boldsymbol{f}_{\boldsymbol{k}}^{\boldsymbol{-}}$ |
| **S** | -1.012 | 0.074 | 0.065 | **S** | -1.005 | 0.072 | 0.063 | **S** | -1.001 | 0.075 | 0.065 |
| **Th^2^** | 0.436 | 0.012 | 0.045 | **Th^2^** | 0.455 | 0.011 | 0.042 | **Th^2^** | 0.464 | 0.013 | 0.045 |
| **Th^3^** | 0.211 | 0.019 | 0.008 | **Th^3^** | 0.199 | 0.019 | 0.007 | **Th^3^** | 0.229 | 0.018 | 0.009 |
| **Th^4^** | -0.240 | -0.006 | 0.045 | **Th^4^** | -0.219 | -0.006 | 0.041 | **Th^4^** | -0.217 | -0.005 | 0.044 |
| **Th^5^** | 0.323 | 0.042 | 0.013 | **Th^5^** | 0.312 | 0.04 | 0.016 | **Th^5^** | 0.277 | 0.043 | 0.012 |
| **N_azo(1)_** | -0.047 | 0.084 | 0.007 | **N_azo(1)_** | -0.051 | 0.082 | 0.007 | **N_azo(1)_** | -0.052 | 0.083 | 0.006 |
| **N_azo(2)_** | 0.129 | 0.084 | 0.042 | **N_azo(2)_** | 0.125 | 0.084 | 0.036 | **N_azo(2)_** | 0.131 | 0.08 | 0.042 |
| **C_Ph(Azo)_** | -0.040 | 0.001 | 0.005 | **C_Ph(Azo)_** | -0.157 | -0.001 | 0.012 | **C_Ph(Azo)_** | -0.265 | 0.002 | 0.003 |
| **C_O_ _(amide Th_^2^_)_** | 0.014 | 0.015 | 0.012 | **C_O_ _(amide Th_^2^_)_** | 0.009 | 0.016 | 0.012 | **C_O_ _(amide Th_^2^_)_** | 0.000 | 0.014 | 0.012 |
| **NH_(amide Th_^2^_)_** | 0.108 | -0.003 | 0.006 | **NH_(amide Th_^2^_)_** | 0.102 | -0.003 | 0.004 | **NH_(amide Th_^2^_)_** | 0.110 | -0.002 | 0.006 |
| **O_(amide Th_^2^_)_** | -0.263 | 0.024 | 0.033 | **O_(amide Th_^2^_)_** | -0.263 | 0.024 | 0.031 | **O_CO_ _(amide)_** | -0.262 | 0.023 | 0.032 |
| **C_Ph(amide Th_^2^_)_** | -0.305 | -0.004 | -0.005 | **C_Ph(amide Th_^2^_)_** | -0.280 | -0.003 | -0.005 | **C_Ph(amide Th_^2^_)_** | -0.304 | -0.005 | -0.005 |
| **C4_Ph(amide Th_^2^_)_** | 1.428 | 0.008 | 0.011 | **C4_Ph(amide Th_^2^_)_** | 1.415 | 0.008 | 0.01 | **C4_Ph(amide Th_^2^_)_** | 1.424 | 0.003 | 0.005 |
| **C_Oacyl_** | -0.430 | 0.008 | 0.007 | **C_Oacyl_** | -0.431 | 0.008 | 0.007 | **C_Oacyl_** | -0.430 | 0.007 | 0.007 |
| **Me_Th_** | -0.795 | -0.009 | -0.01 | **Me_Th_** | -0.838 | -0.009 | -0.009 | **Me_Th_** | -0.795 | -0.009 | -0.01 |
| **C4_Ph(Azo)_** | 0.503 | 0.031 | 0.021 | **C4_Ph(Azo)_** | -0.511 | 0.04 | 0.027 | **C4_Ph(Azo)_** | 0.220 | 0.024 | 0.013 |
| **C_MeCO_** | -0.450 | -0.004 | -0.005 | **C_MeCO_** | -0.450 | -0.005 | -0.005 | **C_MeCO_** | -0.450 | -0.004 | -0.005 |
| **O _COCH3_** | -0.254 | 0.02 | 0.022 | **O _COCH3_** | -0.254 | 0.02 | 0.021 | **O _COCH3_** | -0.253 | 0.018 | 0.022 |
| **NH_Th_** | 0.219 | 0.012 | 0.048 | **NH_Th_** | 0.229 | 0.011 | 0.045 | **NH_Th_** | 0.219 | 0.014 | 0.048 |
| **C_Ph(NH)_** | -0.209 | -0.003 | -0.002 | **C_Ph(NH)_** | -0.205 | -0.003 | -0.002 | **C_Ph(NH)_** | -0.218 | -0.004 | -0.001 |
| **C_MeAzo_** | -0.524 | -0.013 | -0.01 | **O_methoxy_** | -0.159 | 0.026 | 0.032 | **Cl** | 0.454 | 0.09 | 0.076 |
|  |  |  |  | **C_methoxy_** | -0.327 | -0.021 | -0.018 |  |  |  |  |

|  | **7a** |  |  |  | **7b** |  |  |  | **7c** |  |  |
| --- | --- | --- | --- | --- | --- | --- | --- | --- | --- | --- | --- |
| **Atom** | **charge** | $\boldsymbol{f}_{\boldsymbol{k}}^{\boldsymbol{+}}$ | $\boldsymbol{f}_{\boldsymbol{k}}^{\boldsymbol{-}}$ | **Atom** | **charge** | $\boldsymbol{f}_{\boldsymbol{k}}^{\boldsymbol{+}}$ | $\boldsymbol{f}_{\boldsymbol{k}}^{\boldsymbol{-}}$ | **Atom** | **charge** | $\boldsymbol{f}_{\boldsymbol{k}}^{\boldsymbol{+}}$ | $\boldsymbol{f}_{\boldsymbol{k}}^{\boldsymbol{-}}$ |
| **S** | -1.135 | 0.043 | 0.079 | **S** | -0.974 | 0.041 | 0.063 | **S** | -1.135 | 0.048 | 0.08 |
| **Th^2^** | 0.462 | -0.003 | 0.055 | **Th^2^** | 0.254 | -0.004 | 0.037 | **Th^2^** | 0.469 | -0.002 | 0.056 |
| **Th^3^** | -0.180 | 0.034 | 0.015 | **Th^3^** | -0.066 | 0.035 | 0.01 | **Th^3^** | -0.160 | 0.035 | 0.016 |
| **Th^4^** | -0.051 | 0.008 | 0.035 | **Th^4^** | 0.046 | 0.008 | 0.024 | **Th^4^** | -0.077 | 0.008 | 0.036 |
| **Th^5^** | 0.174 | 0.035 | 0.01 | **Th^5^** | 0.008 | 0.032 | 0.011 | **Th^5^** | 0.160 | 0.038 | 0.01 |
| **C_O amideTh_^4^** | -0.121 | 0.009 | 0.01 | **C_O amideTh_^4^** | -0.085 | 0.008 | 0.01 | **C_O amideTh_^4^** | -0.155 | 0.01 | 0.011 |
| **NH_amideTh_^4^** | 0.027 | 0.002 | 0.016 | **NH_amideTh_^4^** | 0.006 | 0.002 | 0.025 | **NH_amideTh_^4^** | 0.011 | 0.002 | 0.014 |
| **C_Ph(amideTh_^4^_)_** | 0.114 | -0.004 | 0.001 | **C_Ph(amideTh_^4^_)_** | -0.033 | -0.004 | 0.02 | **C_Ph(amideTh_^4^_)_** | -0.160 | -0.004 | -0.001 |
| **C_O_ _(amide Th_^2^_)_** | 0.150 | 0.042 | 0.016 | **C_O_ _(amide Th_^2^_)_** | 0.034 | 0.043 | 0.014 | **C_O_ _(amide Th_^2^_)_** | 0.148 | 0.043 | 0.016 |
| **NH_(amide Th_^2^_)_** | 0.117 | -0.001 | 0.012 | **NH_(amide Th_^2^_)_** | 0.140 | -0.001 | 0.007 | **NH_(amide Th_^2^_)_** | 0.116 | -0.001 | 0.013 |
| **O_(amide Th_^2^_)_** | -0.314 | 0.043 | 0.035 | **O_(amide Th_^2^_)_** | -0.314 | 0.043 | 0.026 | **O_(amide Th_^2^_)_** | -0.313 | 0.044 | 0.035 |
| **C_Ph(amide Th_^2^_)_** | -0.212 | 0.034 | -0.004 | **C_Ph(amide Th_^2^_)_** | -0.196 | 0.036 | -0.004 | **C_Ph(amide Th_^2^_)_** | -0.206 | 0.031 | -0.004 |
| **C4_Ph(amide Th_^2^_)_** | 1.481 | 0.015 | 0.014 | **C4_Ph(amide Th_^2^_)_** | 1.444 | 0.016 | 0.011 | **C4_Ph(amide Th_^2^_)_** | 1.474 | 0.016 | 0.015 |
| **C_Oacyl_** | -0.421 | 0.056 | 0.009 | **C_Oacyl_** | -0.419 | 0.058 | 0.007 | **C_Oacyl_** | -0.419 | 0.051 | 0.009 |
| **NH_2 Th_^3^** | -0.456 | 0.018 | 0.018 | **NH_2 Th_^3^** | -0.471 | 0.017 | 0.004 | **NH_2 Th_^3^** | -0.465 | 0.018 | 0.019 |
| **C4_Ph(amideTh_^4^_)_** | 0.447 | 0.006 | 0.014 | **C4_Ph(amideTh_^4^_)_** | -0.686 | 0.01 | 0.029 | **C4_Ph(amideTh_^4^_)_** | -0.203 | 0.004 | 0.008 |
| **C_MeCO_** | -0.456 | -0.017 | -0.006 | **C_MeCO_** | -0.457 | -0.017 | -0.005 | **C_MeCO_** | -0.456 | -0.016 | -0.006 |
| **O _COCH3_** | -0.254 | 0.076 | 0.026 | **O _COCH3_** | -0.254 | 0.078 | 0.021 | **O _COCH3_** | -0.253 | 0.071 | 0.026 |
| **NH_Th_** | 0.166 | 0.012 | 0.052 | **NH_Th_** | 0.175 | 0.012 | 0.04 | **NH_Th_** | 0.168 | 0.012 | 0.053 |
| **C_Ph(NH)_** | -0.378 | 0.001 | -0.002 | **C_Ph(NH)_** | -0.256 | 0.001 | -0.002 | **C_Ph(NH)_** | -0.363 | 0.001 | -0.002 |
| **O _(amide Th_^4^_)_** | -0.316 | 0.014 | 0.016 | **O _(amide Th_^4^_)_** | -0.320 | 0.013 | 0.018 | **O _(amide Th_^4^_)_** | -0.318 | 0.014 | 0.016 |
| **C_Me(Th_^4^_)_** | -0.525 | -0.004 | -0.008 | **O_methoxy_** | -0.167 | 0.007 | 0.039 | **Cl** | 0.445 | 0.031 | 0.054 |
|  |  |  |  | **C_methoxy_** | -0.323 | -0.007 | -0.021 |  |  |  |  |

**Molecular Modeling**

| **3a** | 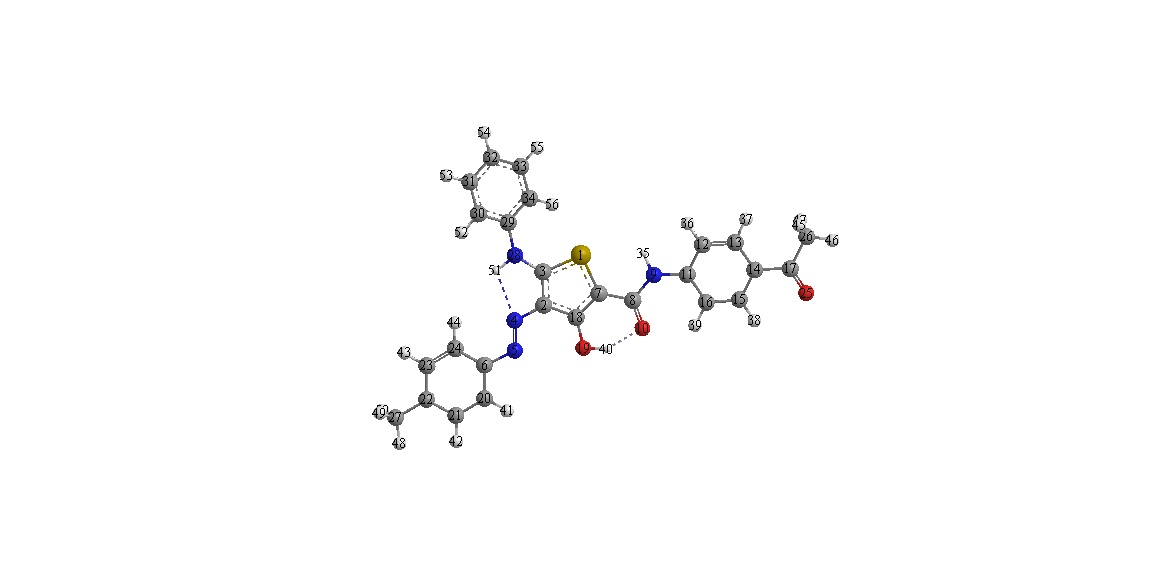 |
| --- | --- |
| **3b** | 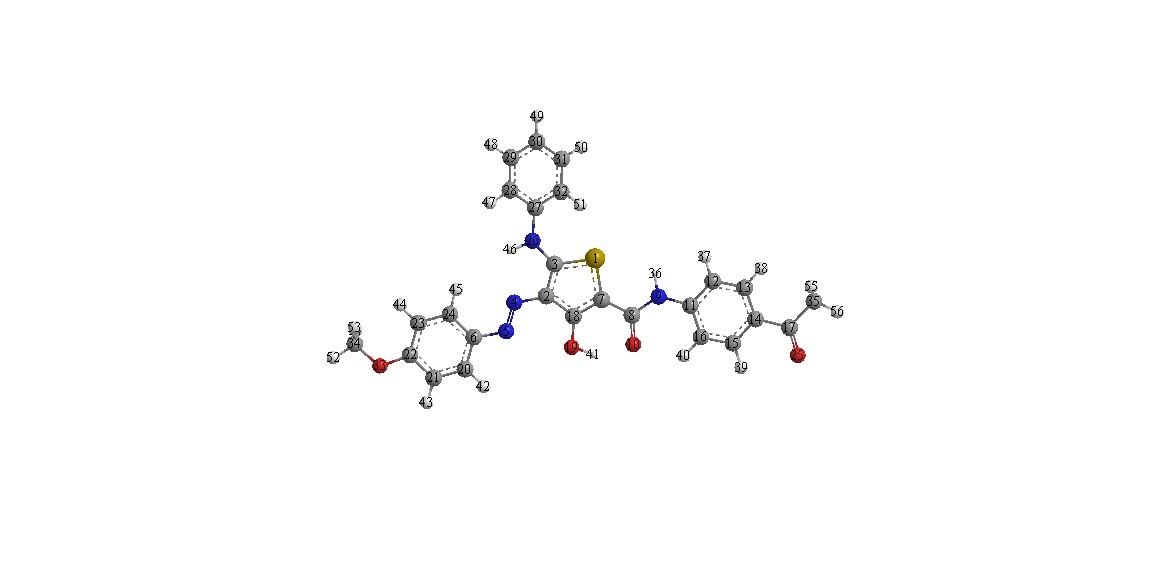 |
| **3c** | 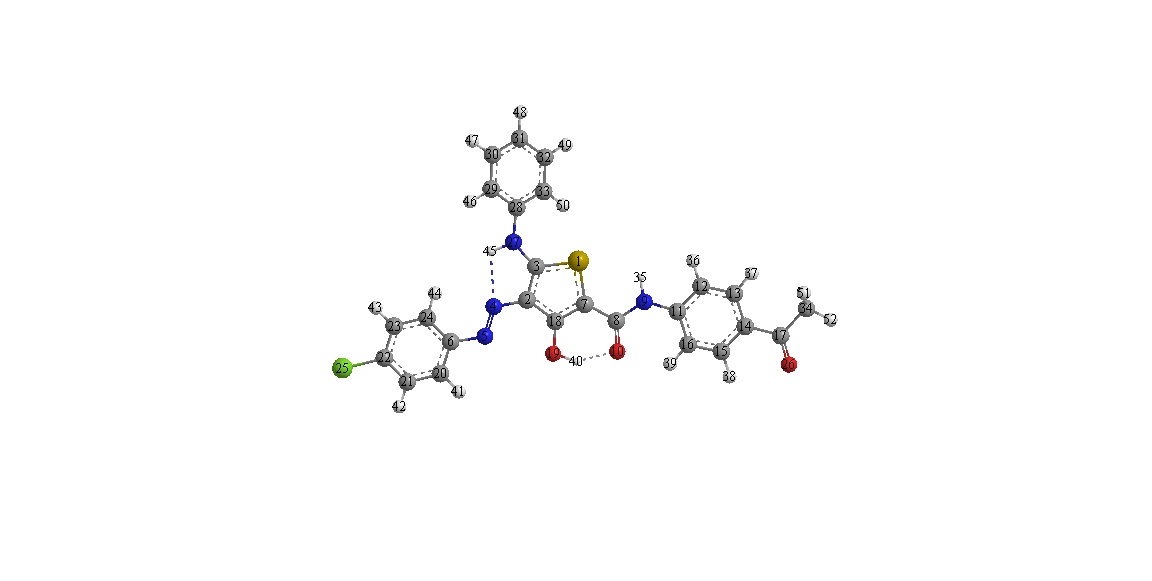 |
| **5a** | 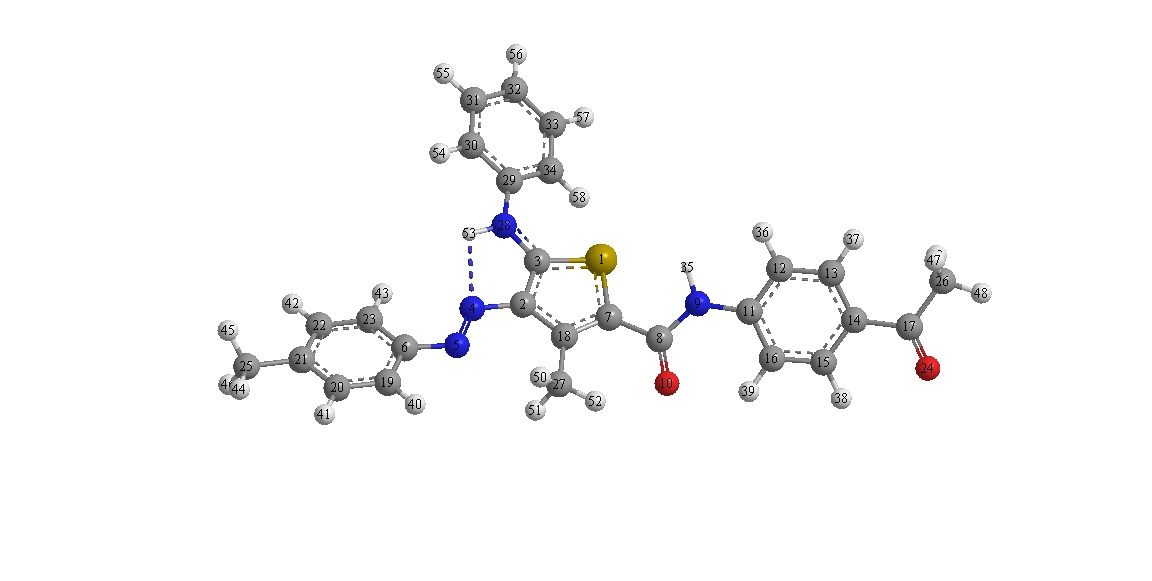 |
| **5b** | 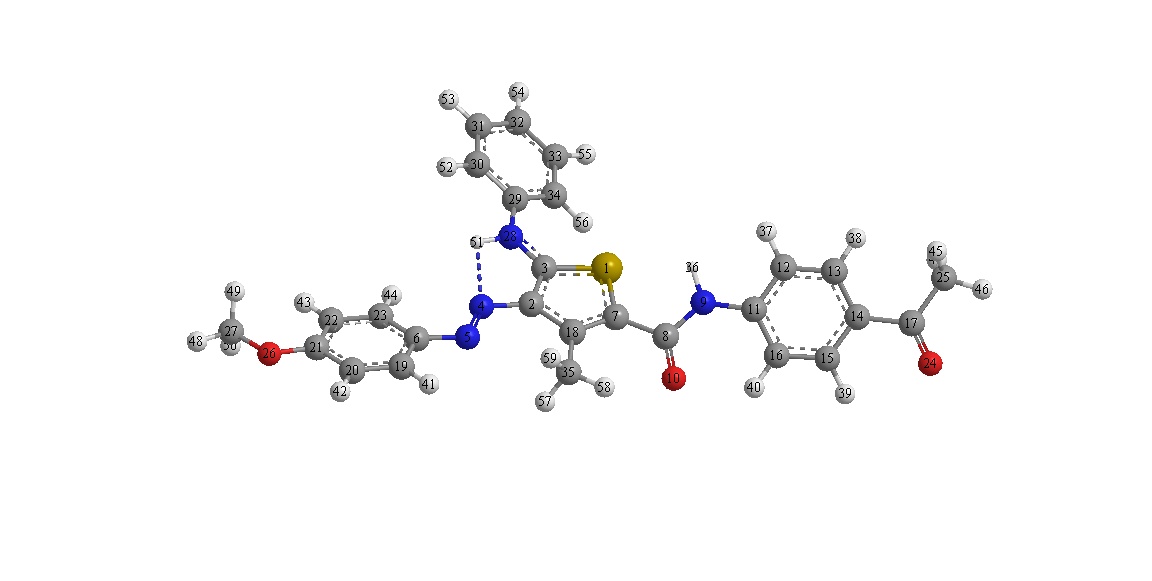 |
| **5c** | 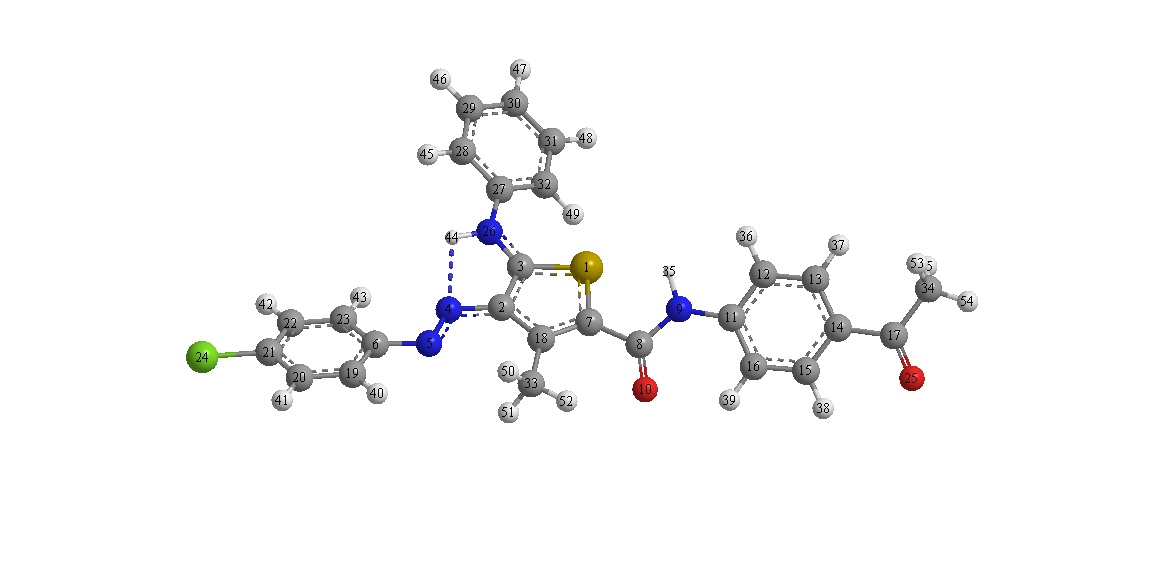 |
| **7a** | 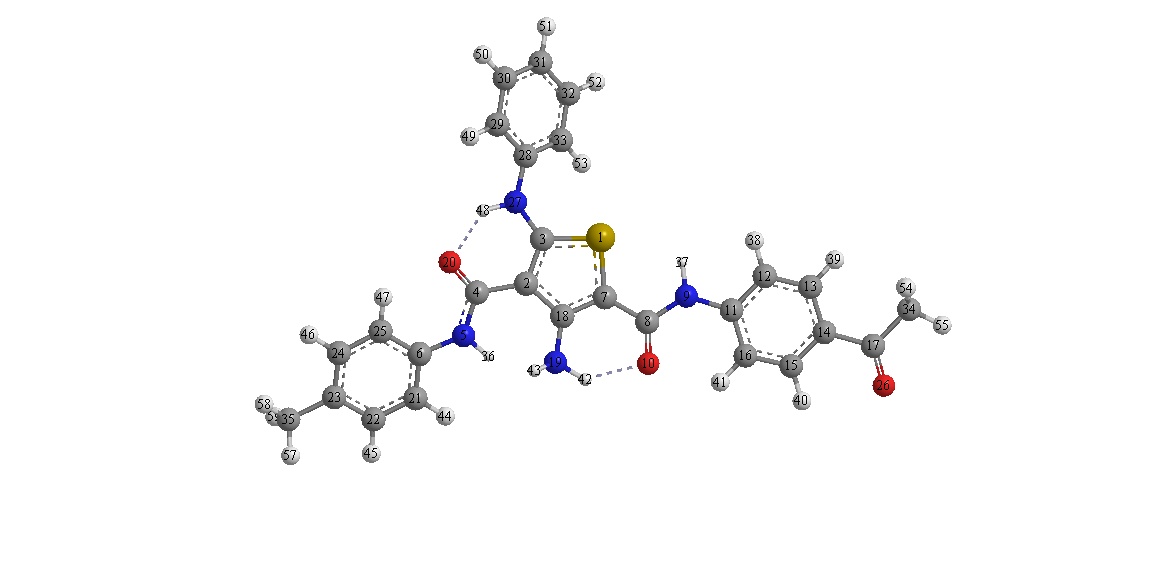 |
| **7b** | 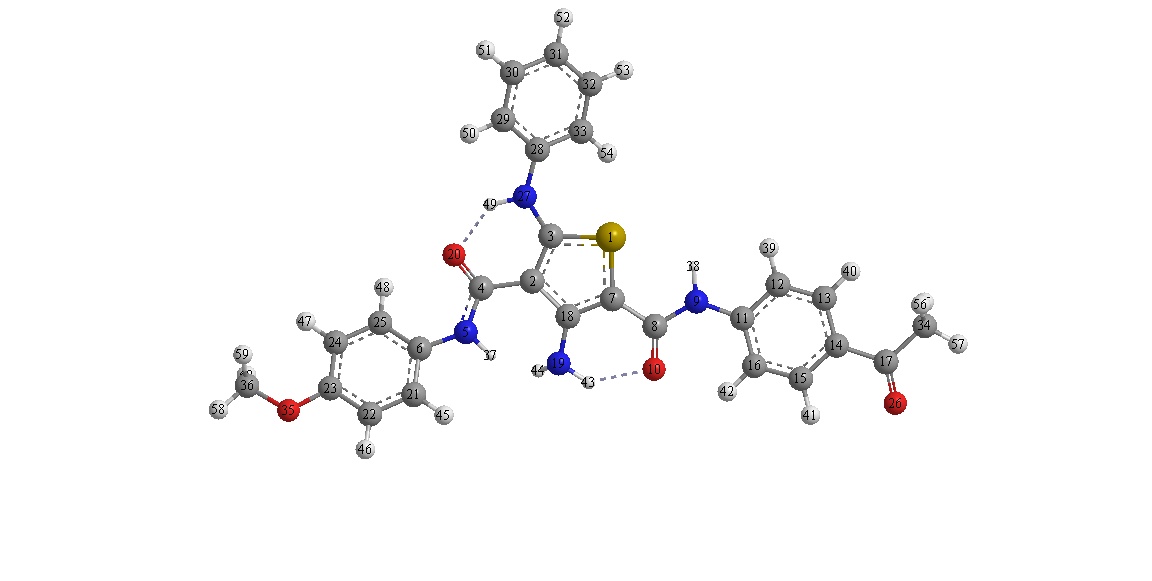 |
| **7c** | 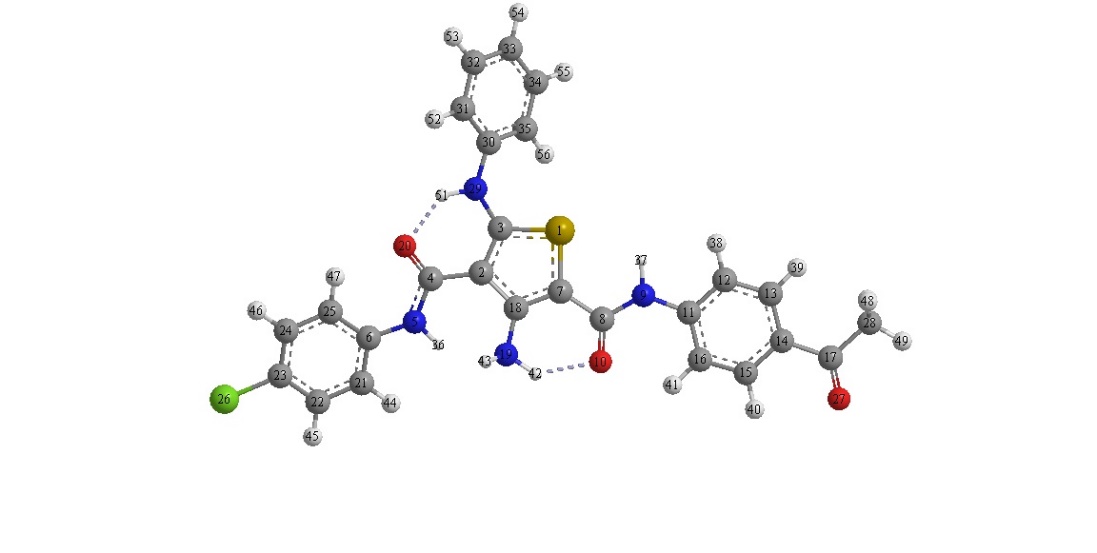 |

**Fig. S1** DFT optimized structures for compounds **3a-c, 5a-c,** and **7a-c**

**Molecular docking**

**Anti-Oxidant –PDB ID: 2AS1**

| 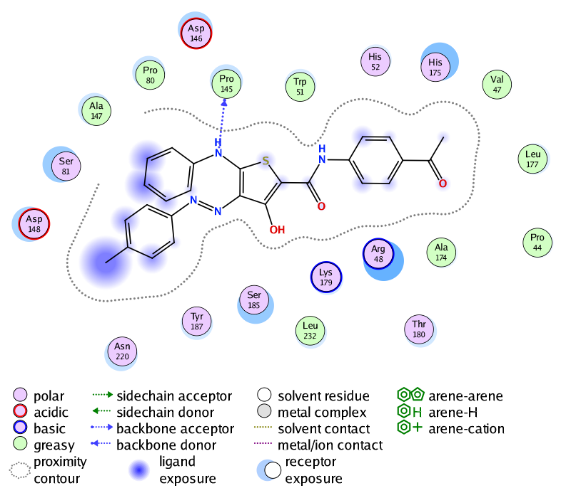 | 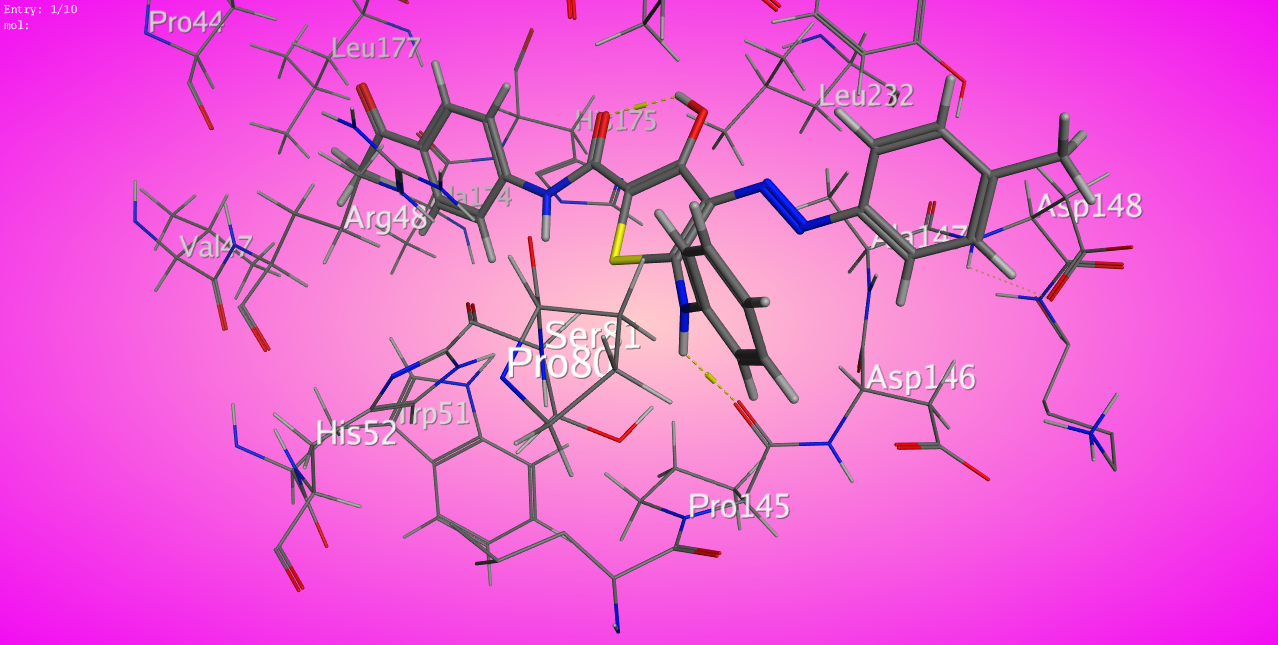 |
| --- | --- |
| **2D** | **3D** |

**Fig. S2** The binding interaction of 3-hydroxythiophene **3a** with (PDB ID: 2AS1).

| 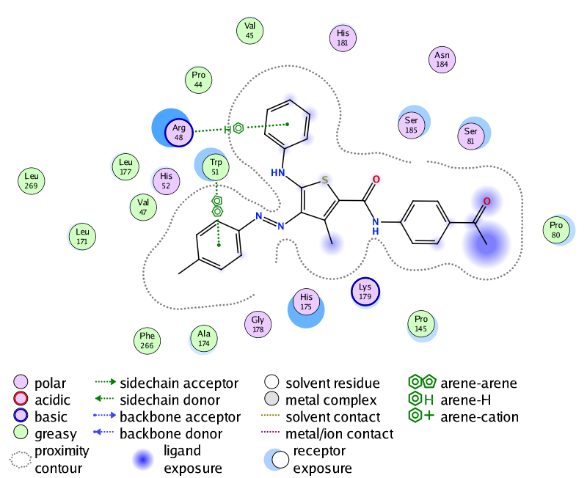 | 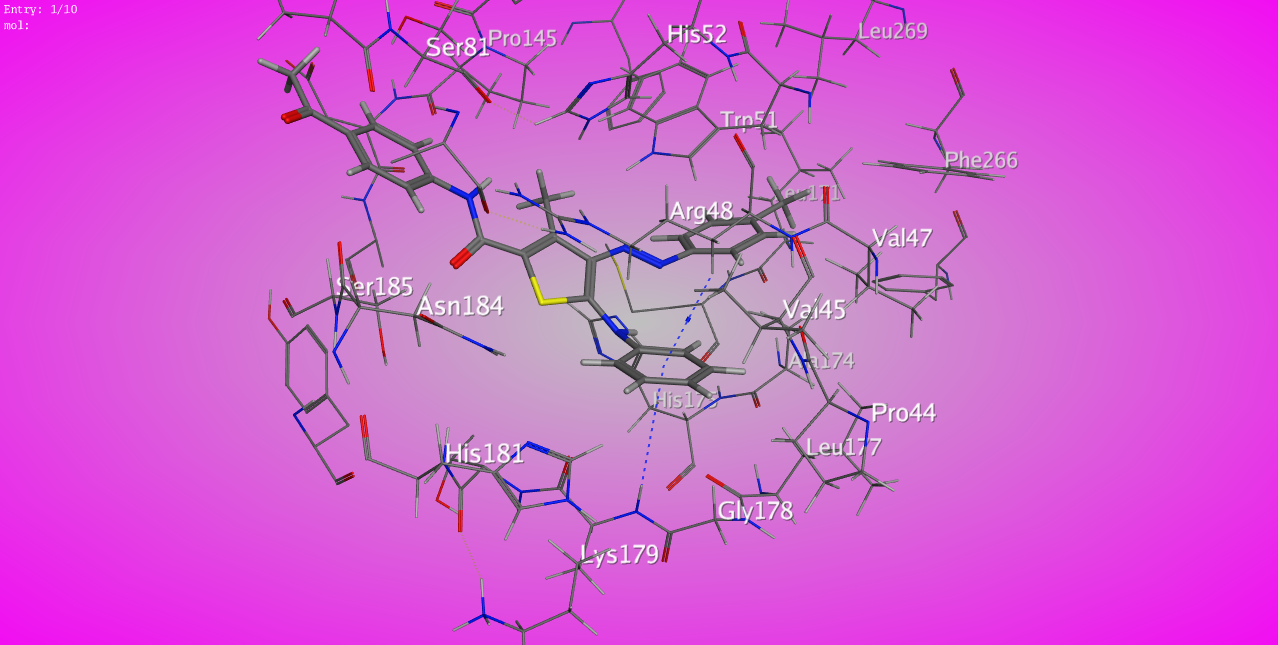 |
| --- | --- |
| **2D** | **3D** |

**Fig. S3** The binding interaction of 3-hydroxythiophene **5a** with (PDB ID: 2AS1).

| 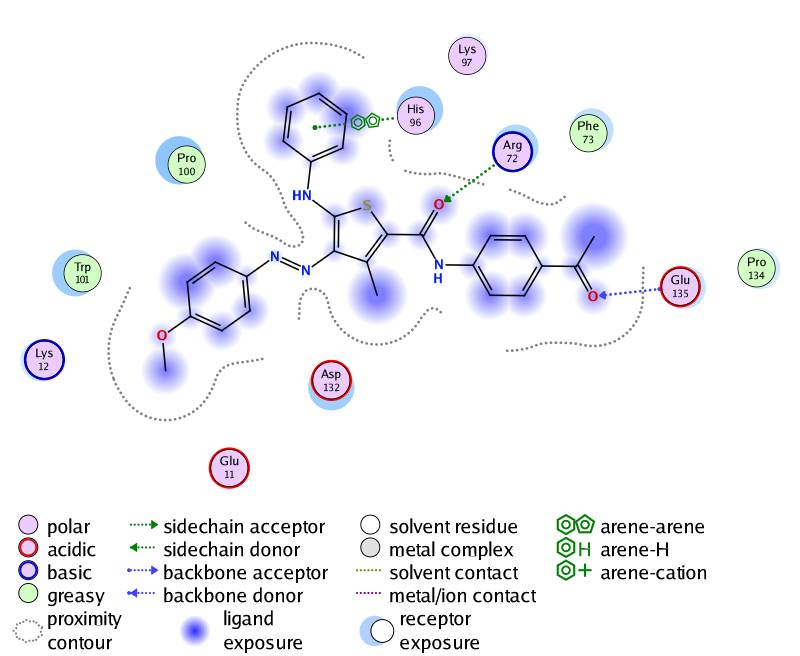 | 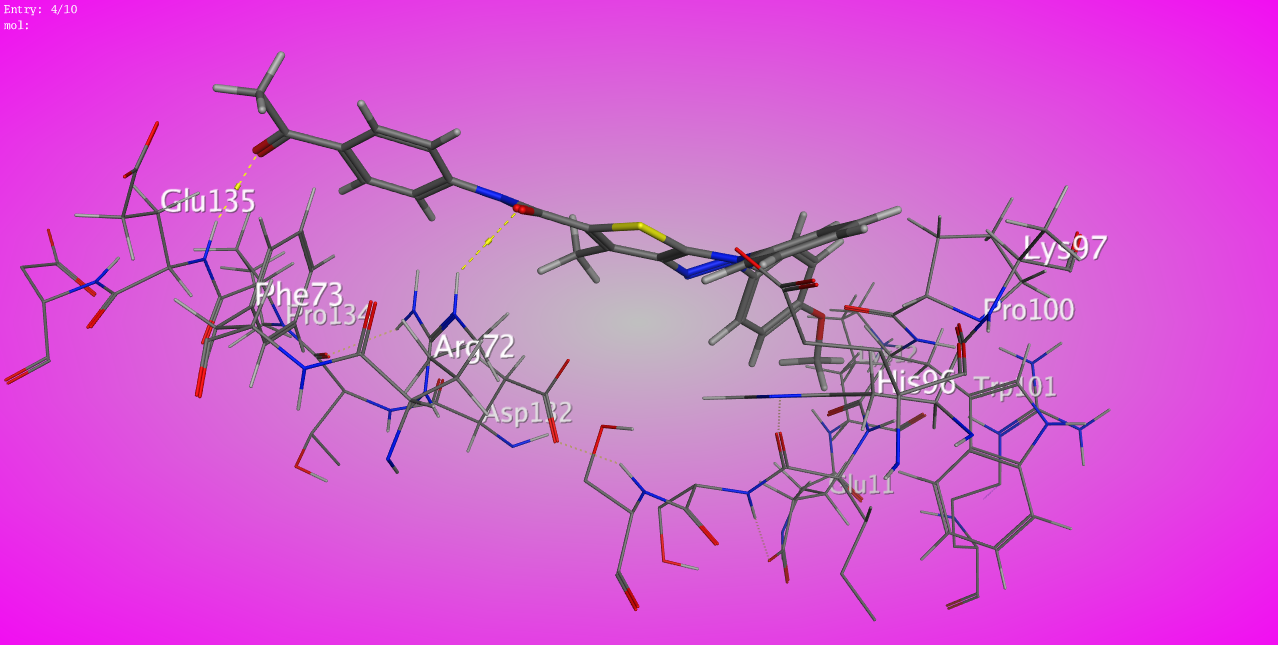 |
| --- | --- |
| **2D** | **3D** |

**Fig. S4** The binding interaction of 3-hydroxythiophene **5b** with (PDB ID: 2AS1).

| 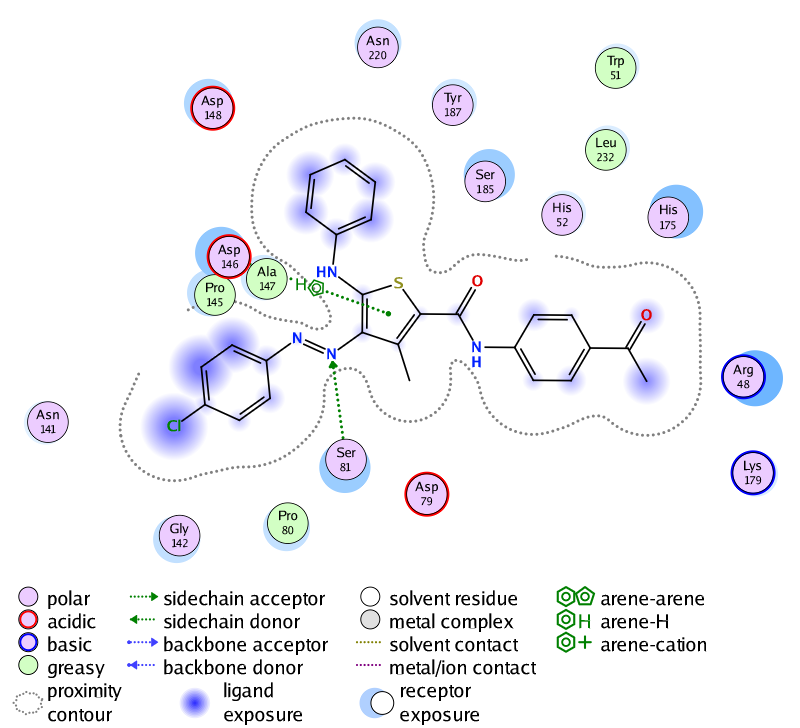 | 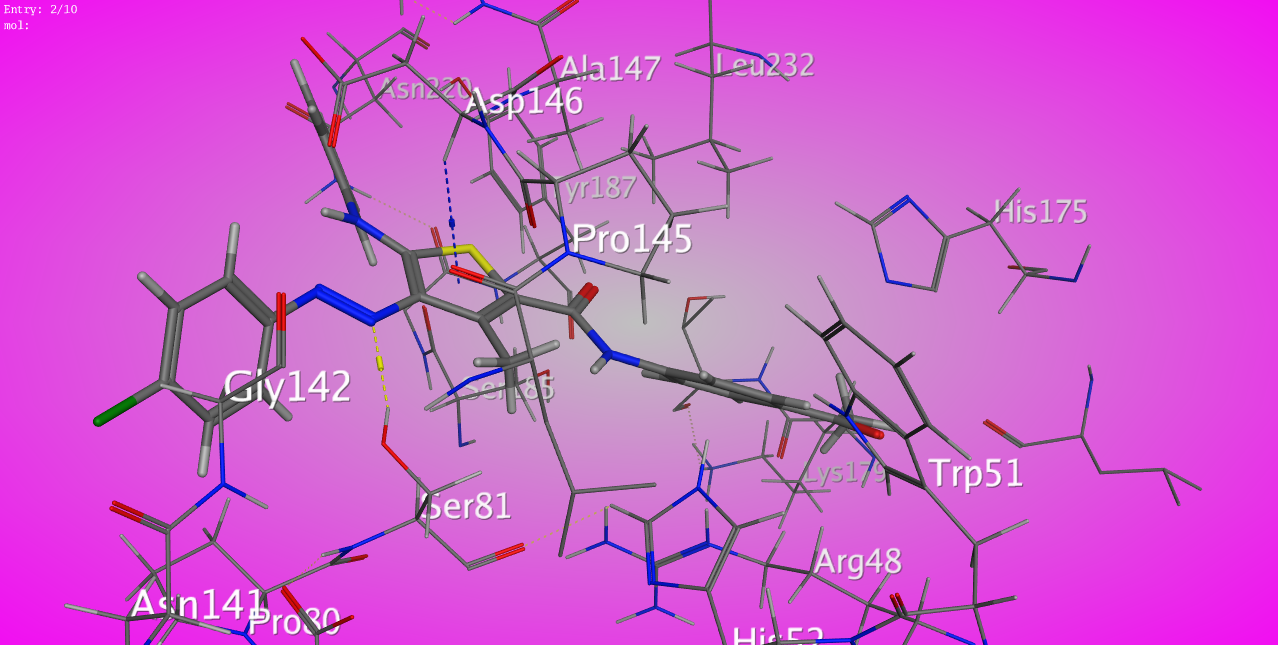 |
| --- | --- |
| **2D** | **3D** |

**Fig. S5** The binding interaction of 3-hydroxythiophene **5c** with (PDB ID: 2AS1).

| 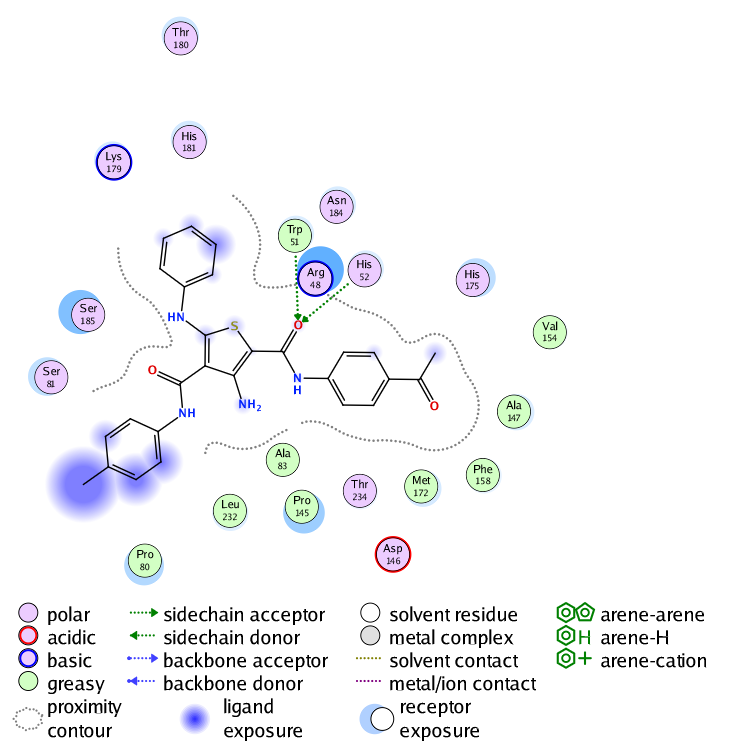 | 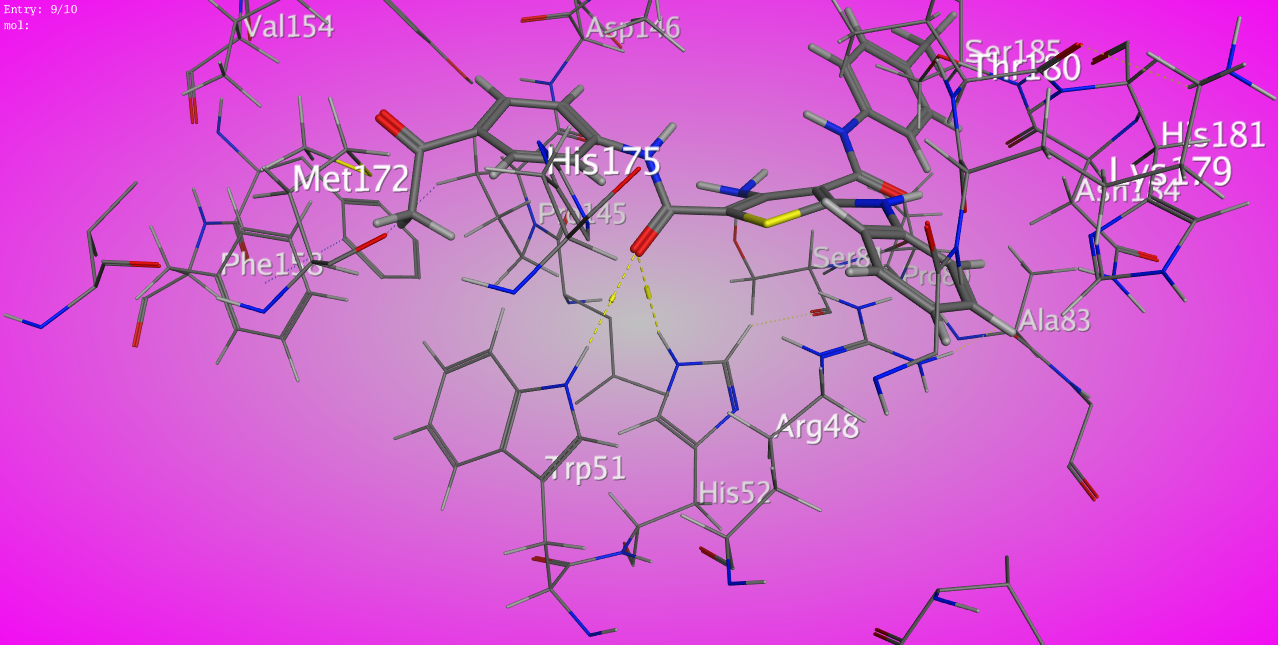 |
| --- | --- |
| **2D** | **3D** |

**Fig. S6** The binding interaction of 3-hydroxythiophene **7a** with (PDB ID: 2AS1).

| 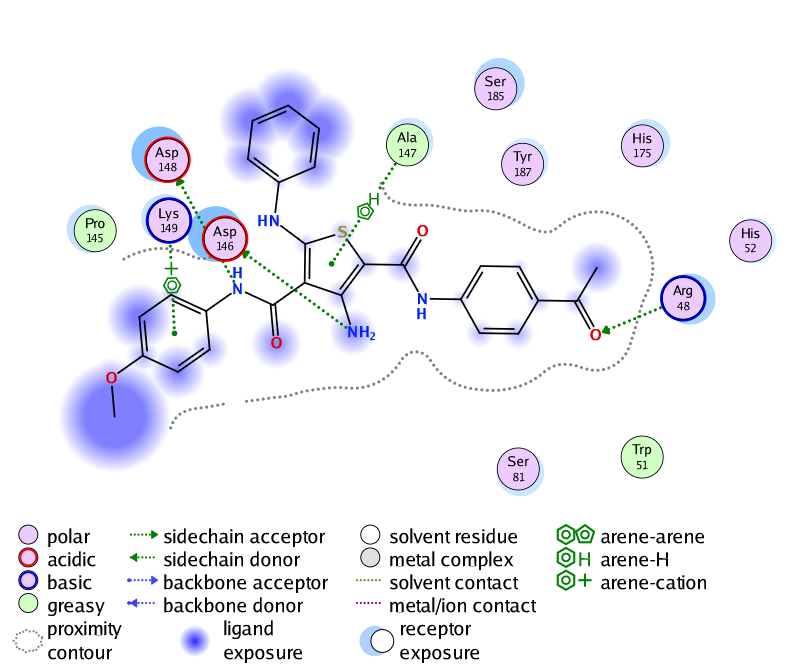 | 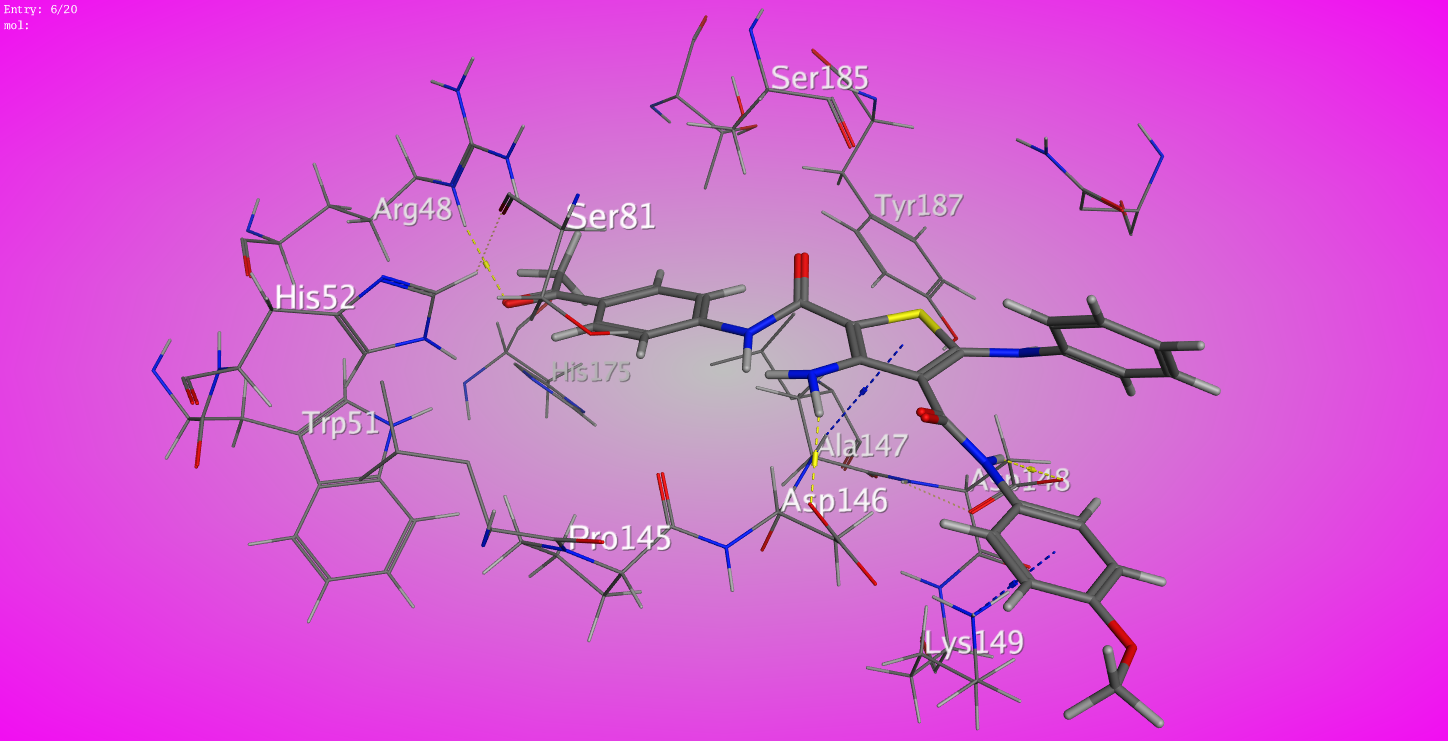 |
| --- | --- |
| **2D** | **3D** |

**Fig. S7** The binding interaction of 3-hydroxythiophene **7b** with (PDB ID: 2AS1).

| 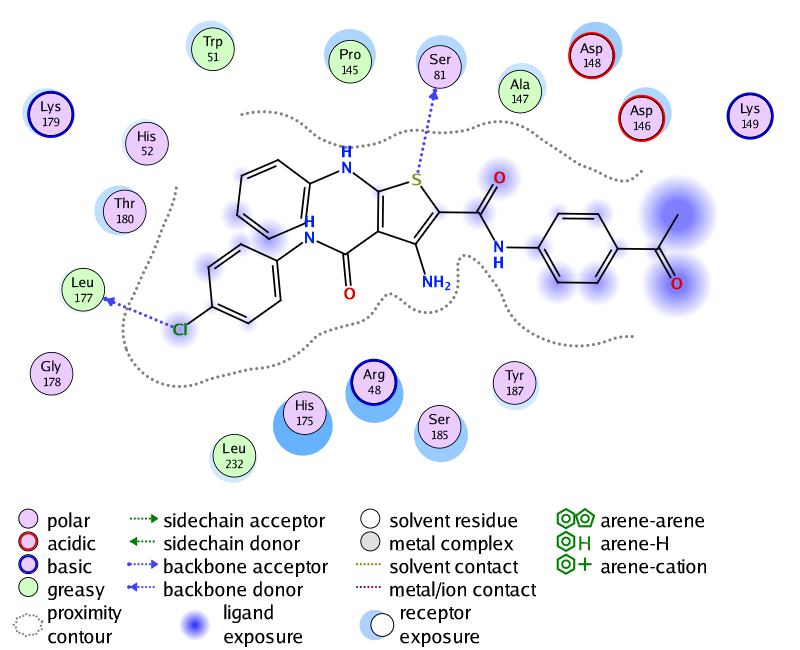 | 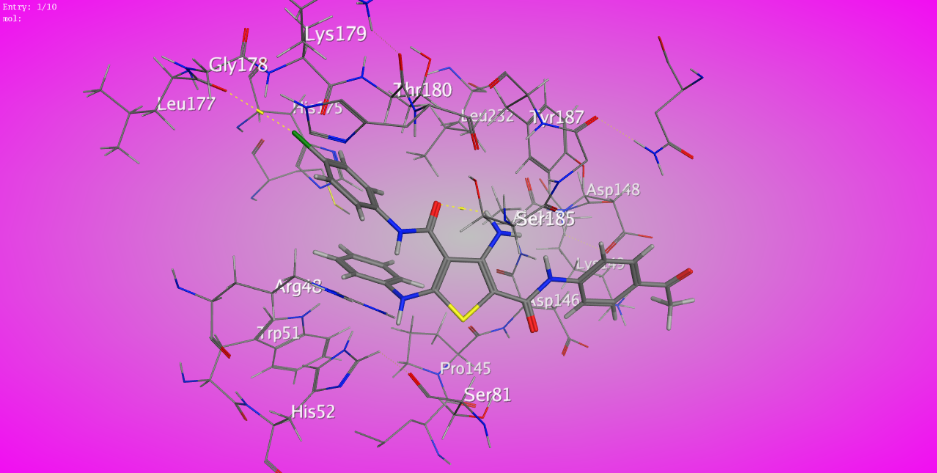 |
| --- | --- |
| **2D** | **3D** |

**Fig. S8** The binding interaction of 3-hydroxythiophene **7c** with (PDB ID: 2AS1).

| 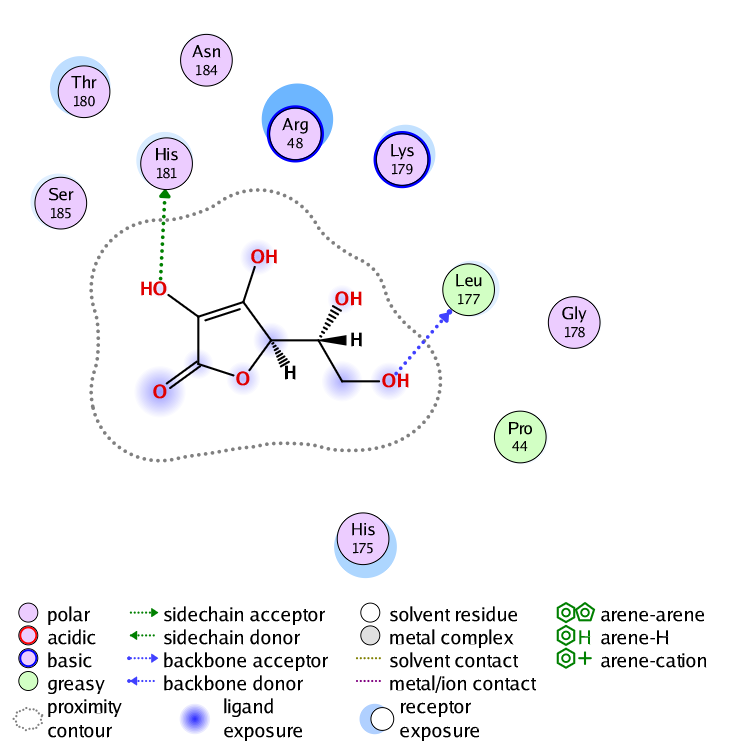 | 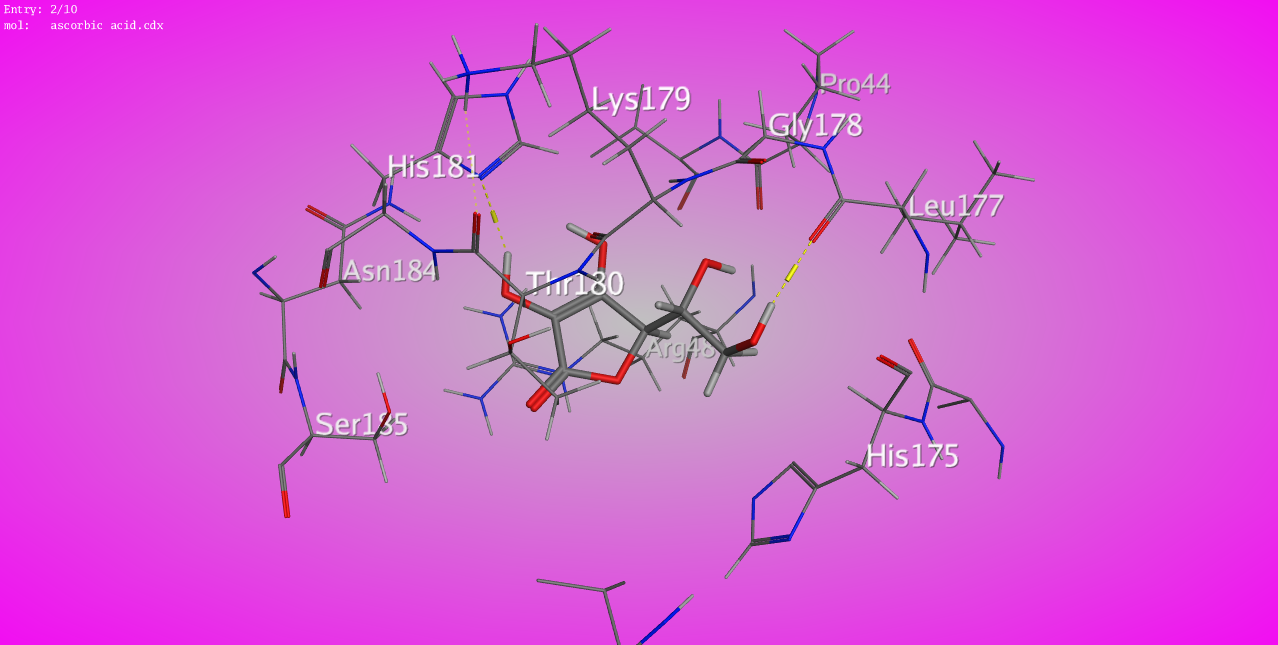 |
| --- | --- |
| **2D** | **3D** |

**Fig. S9** The binding interaction ascorbic acid with (PDB ID: 2AS1).

**P. aeruginosa- PDB ID: 1DD6**

| 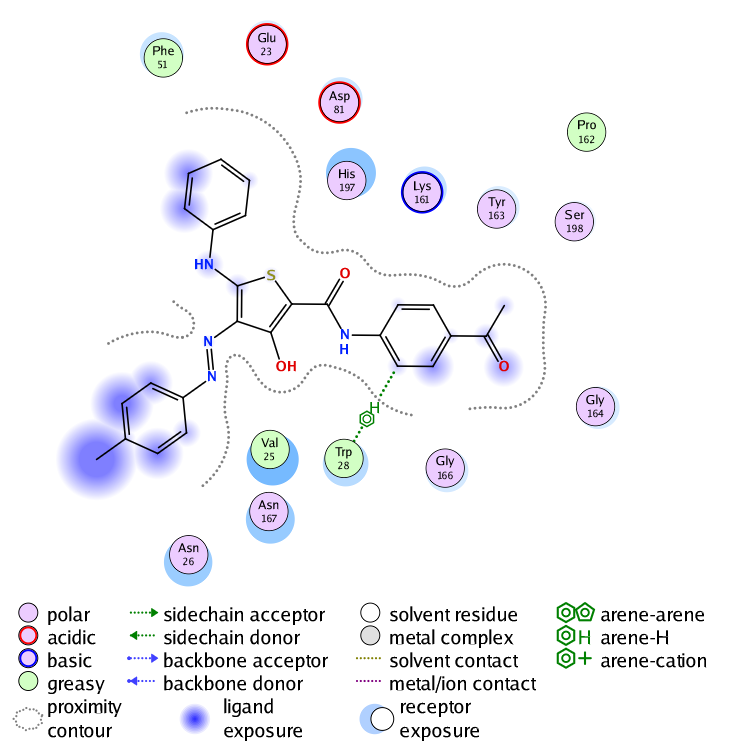 | 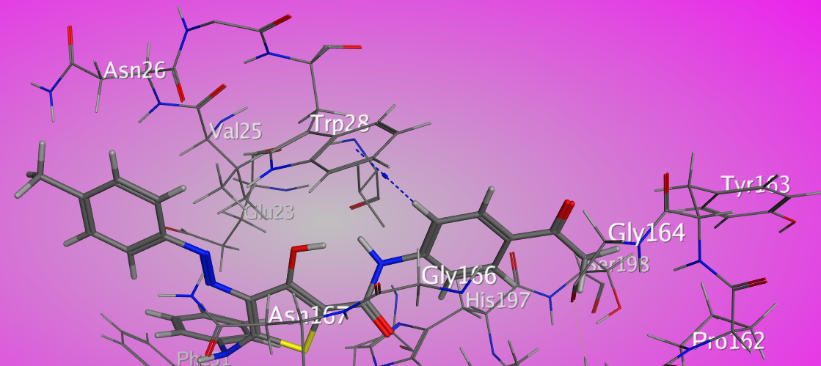 |
| --- | --- |
| **2D** | **3D** |

**Fig. S10** The binding interaction of 3-hydroxythiophene **3a** with (PDB ID: 1DD6).

| 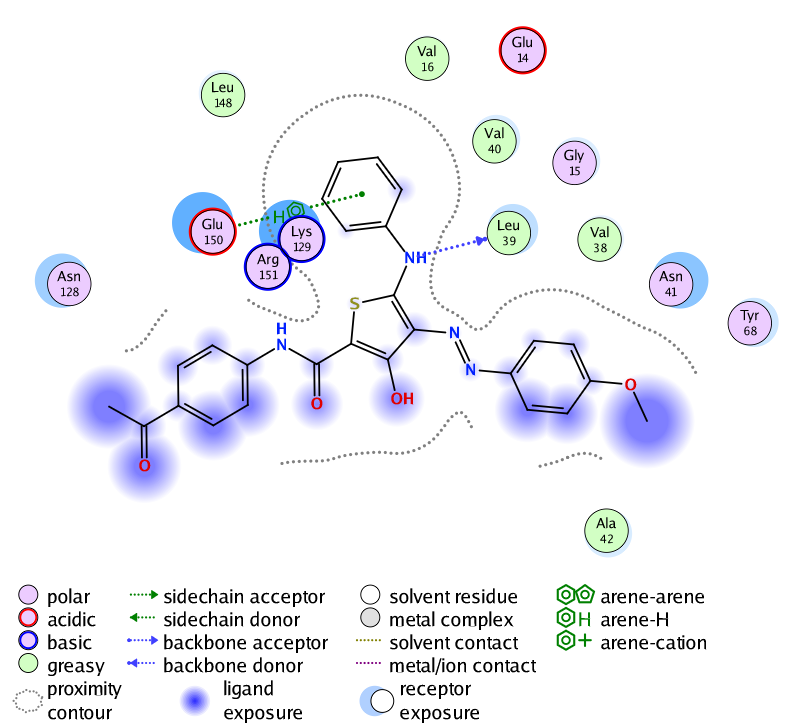 | 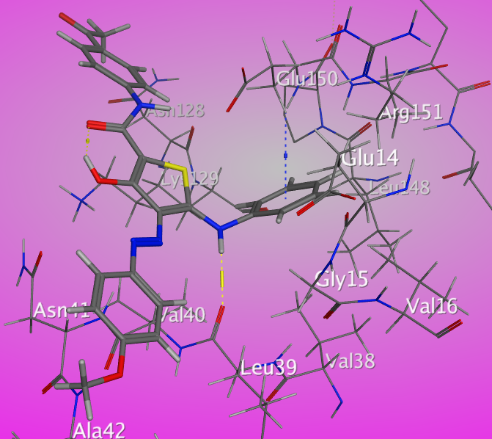 |
| --- | --- |
| 2D | 3D |

**Fig. S11** The binding interaction of 3-hydroxythiophene **3b** with (PDB ID: 1DD6).

| 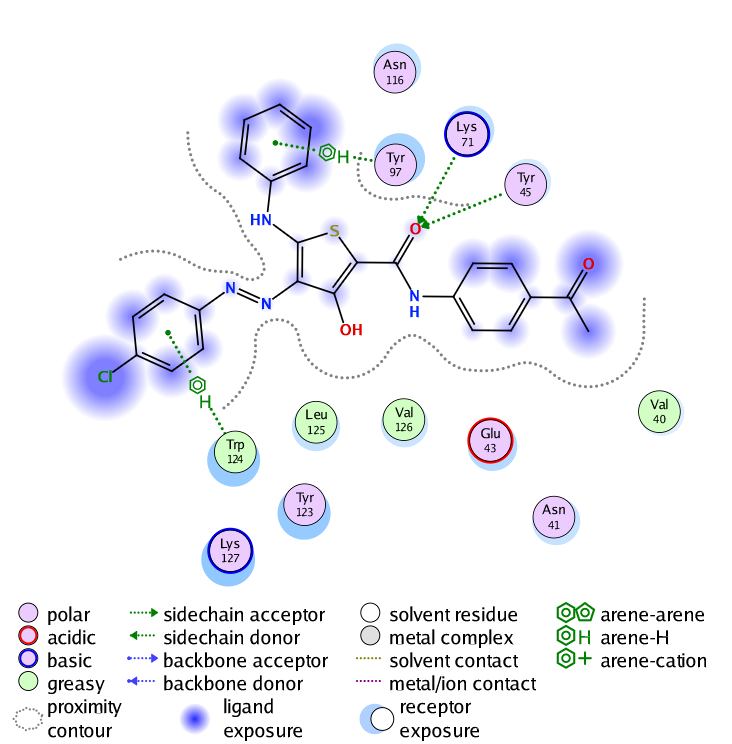 | 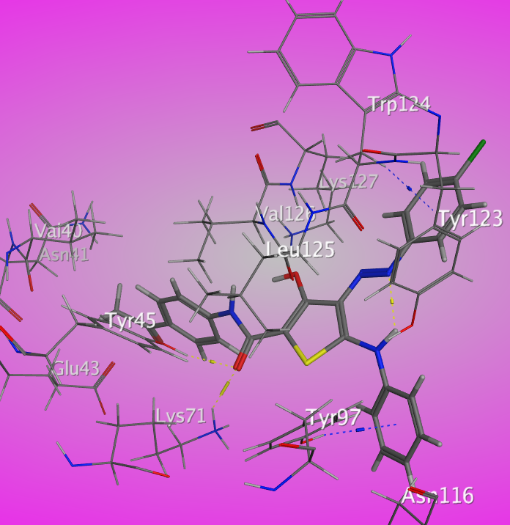 |
| --- | --- |
| 2D | 3D |

**Fig. S12** The binding interaction of 3-hydroxythiophene **3c** with (PDB ID: 1DD6).

| 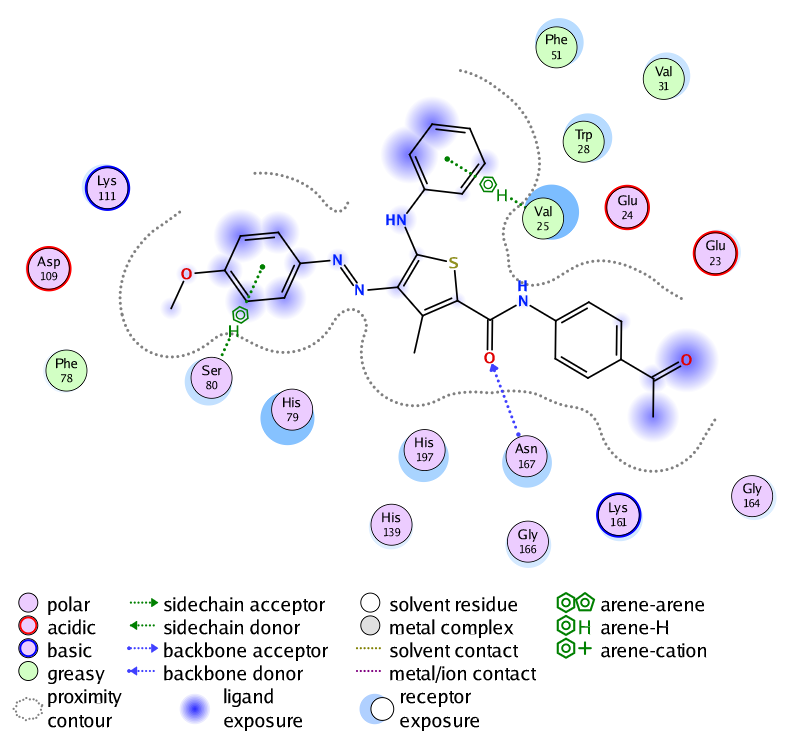 | 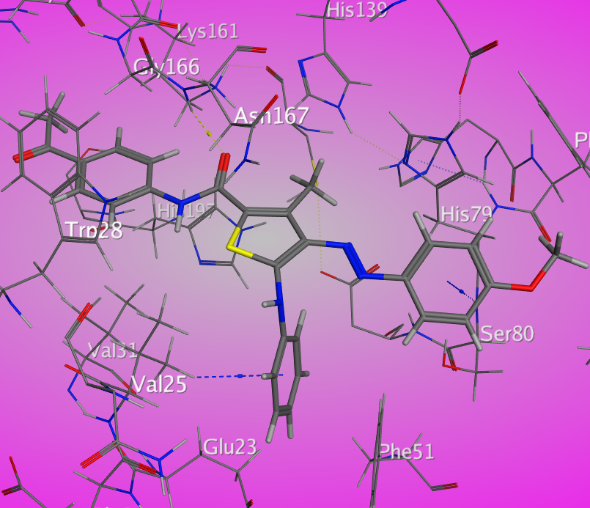 |
| --- | --- |
| 2D | 3D |

**Fig. S13** The binding interaction of 3-methylthiophene **5b** with (PDB ID: 1DD6).

| 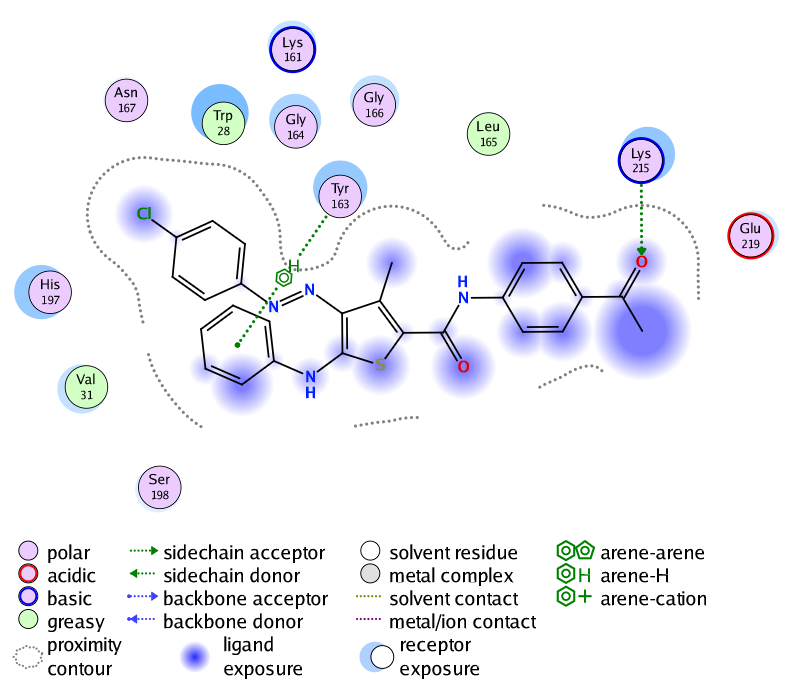 | 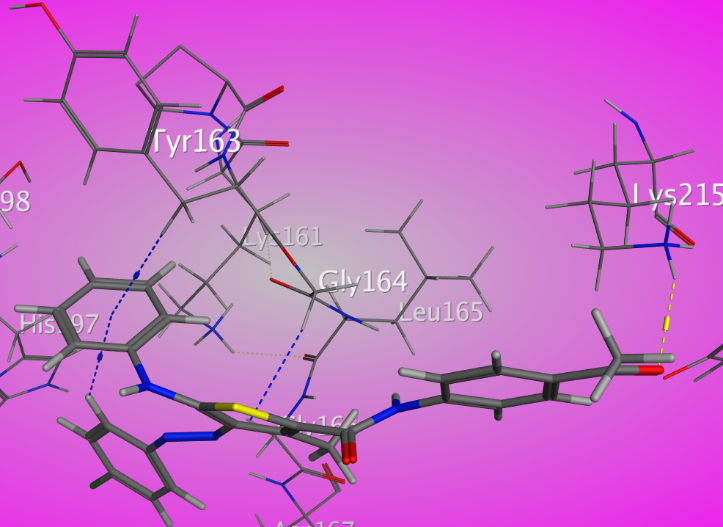 |
| --- | --- |
| 2D | 3D |

**Fig. S14** The binding interaction of 3-methylthiophene **5c** with (PDB ID: 1DD6).

| 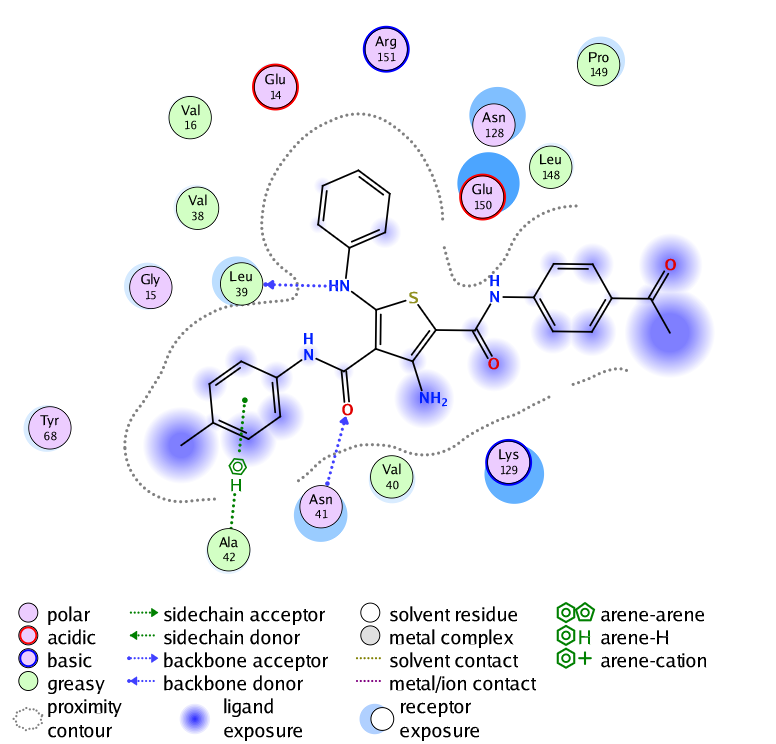 | 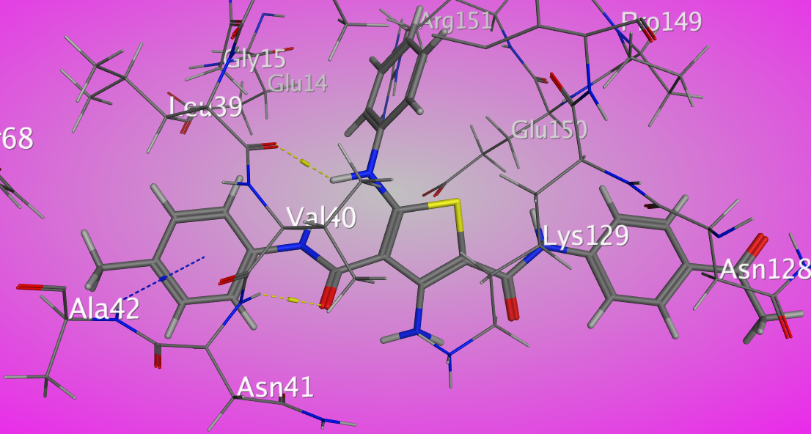 |
| --- | --- |
| 2D | 3D |

**Fig. S15** The binding interaction of 3-aminothiophene **7a** with (PDB ID: 1DD6).

| 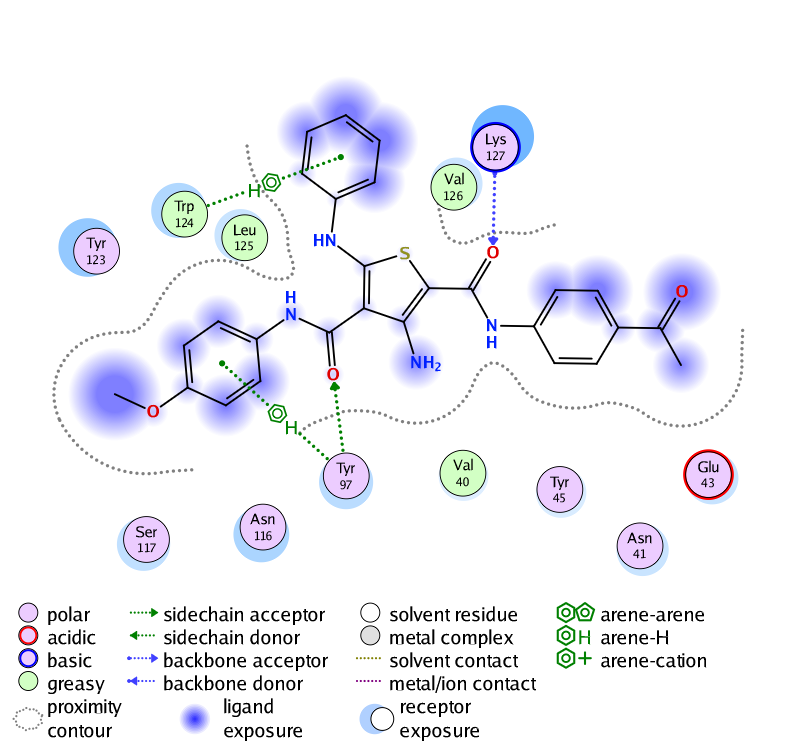 | 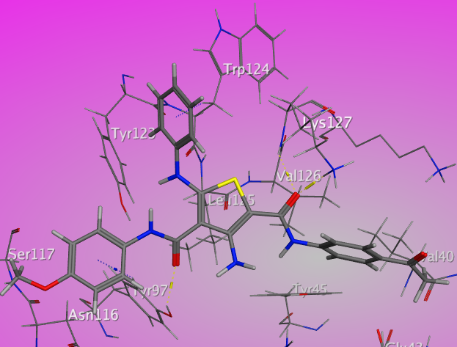 |
| --- | --- |
| 2D | 3D |

**Fig. S16** The binding interaction of 3-aminothiophene **7b** with (PDB ID: 1DD6).

| 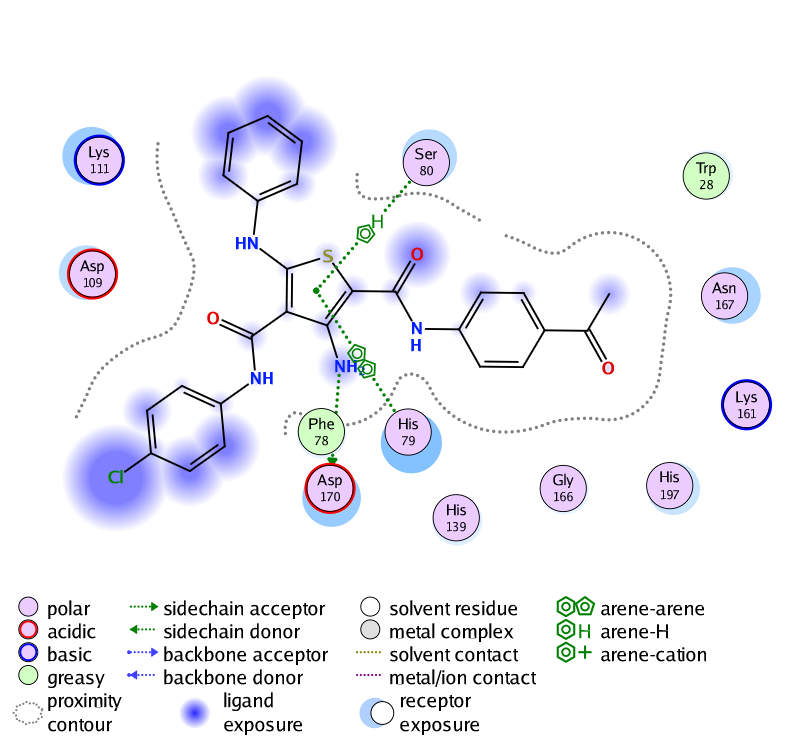 | 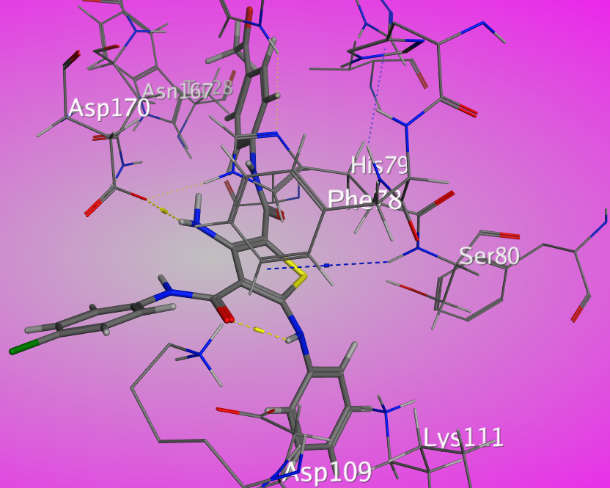 |
| --- | --- |
| 2D | 3D |

**Fig. S17** The binding interaction of 3-aminothiophene **7c** with (PDB ID: 1DD6).

| 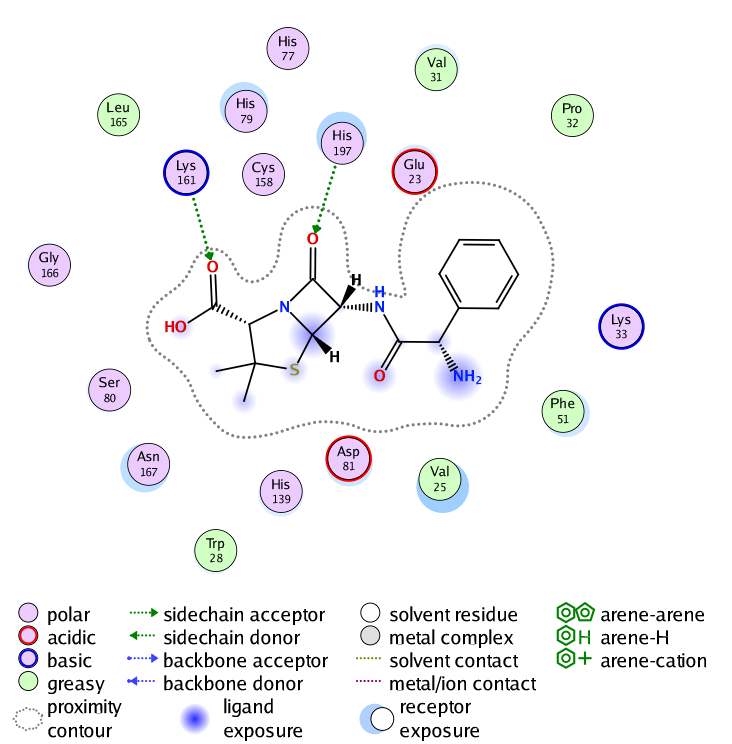 | 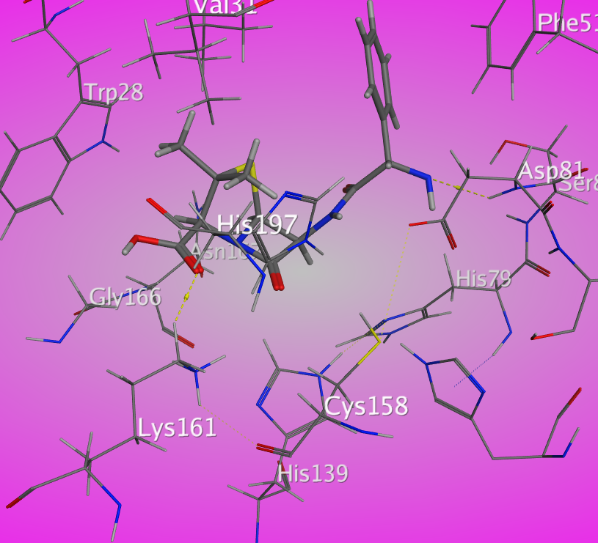 |
| --- | --- |
| 2D | 3D |

**Fig. S18** The binding interaction of with ampicillin (PDB ID: 1DD6).

**S. Aureus - PDB ID: 2MLM**

| 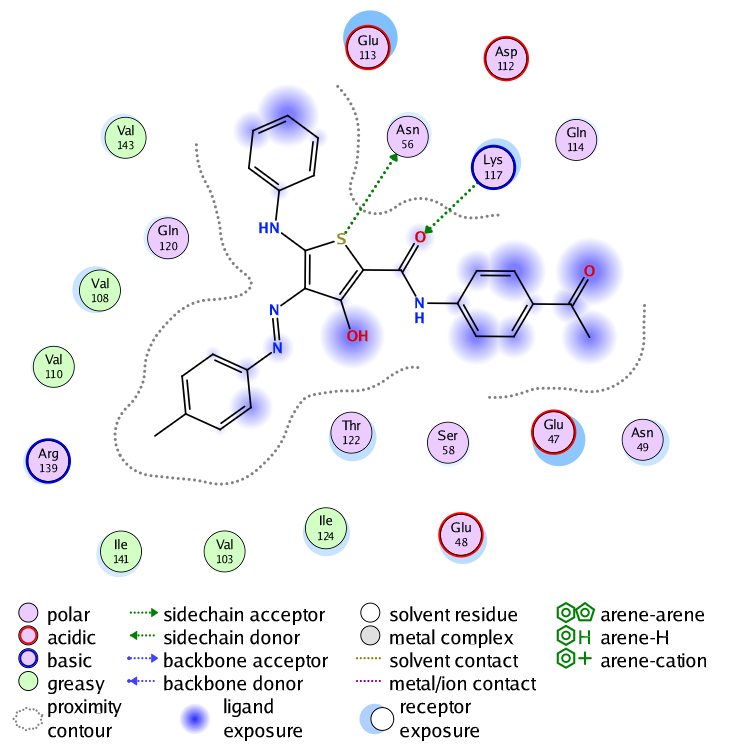 | 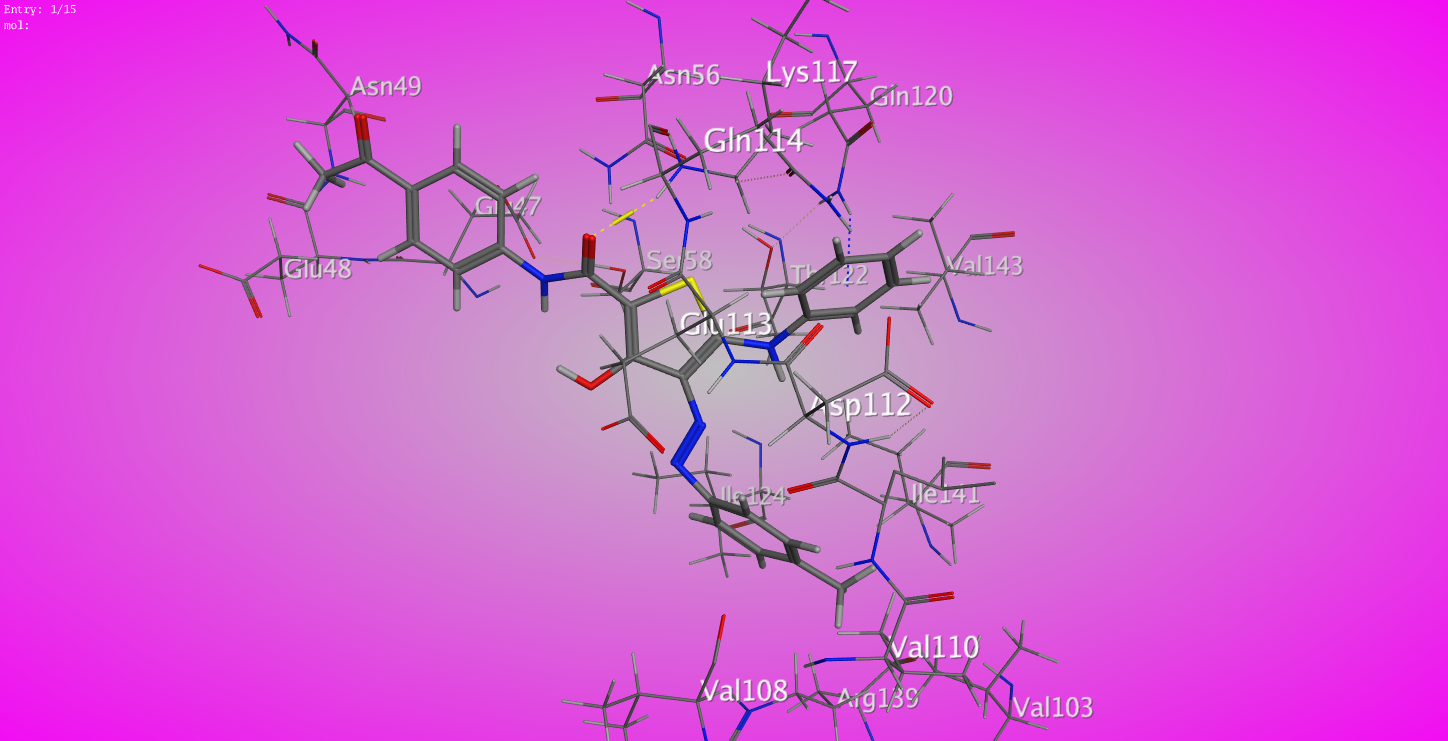 |
| --- | --- |
| 2D | 3D |

**Fig. S19** The binding interaction of 3-hydroxythiophene **3a** with (PDB ID: 2MLM).

| 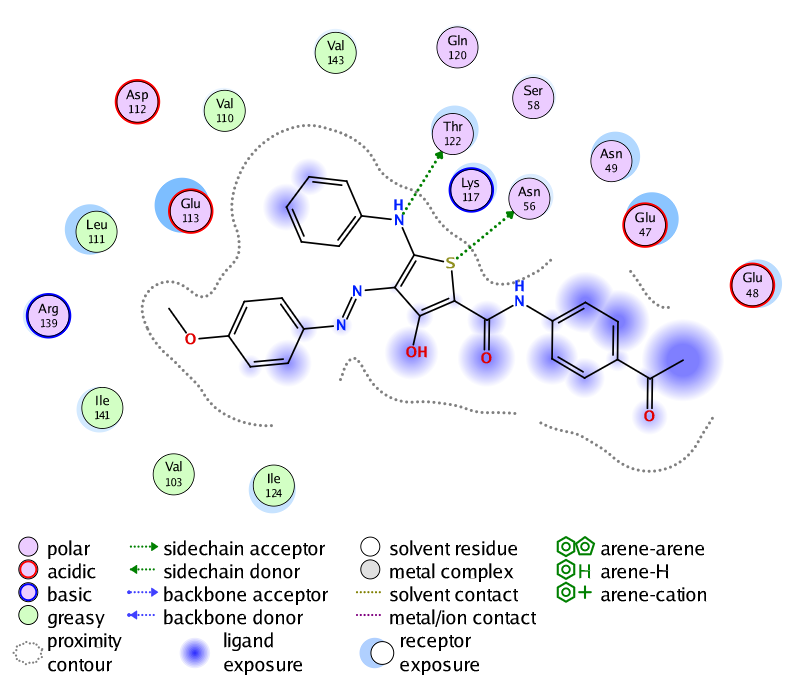 | 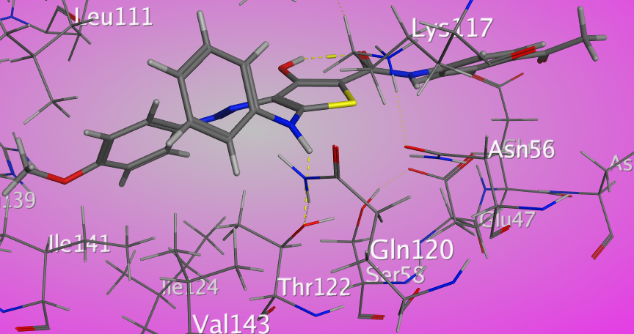 |
| --- | --- |
| 2D | 3D |

**Fig. S20** The binding interaction of 3-hydroxythiophene **3b** with (PDB ID: 2MLM).

| 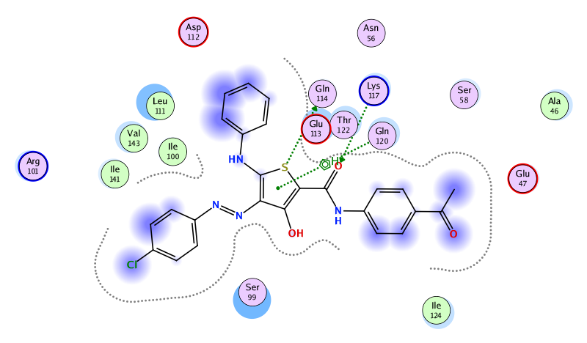 | 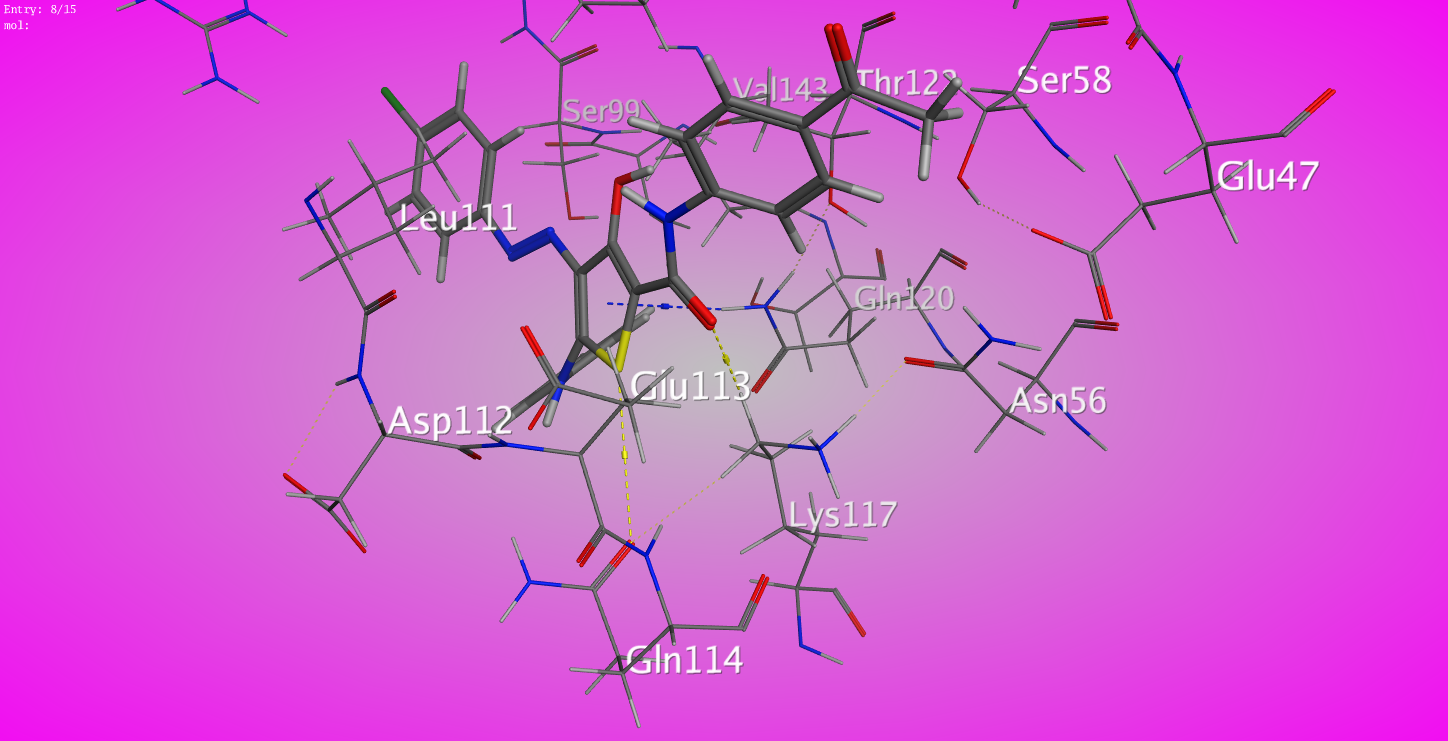 |
| --- | --- |
| 2D | 3D |

**Fig. S21** The binding interaction of 3-hydroxythiophene **3c** with (PDB ID: 2MLM).

| 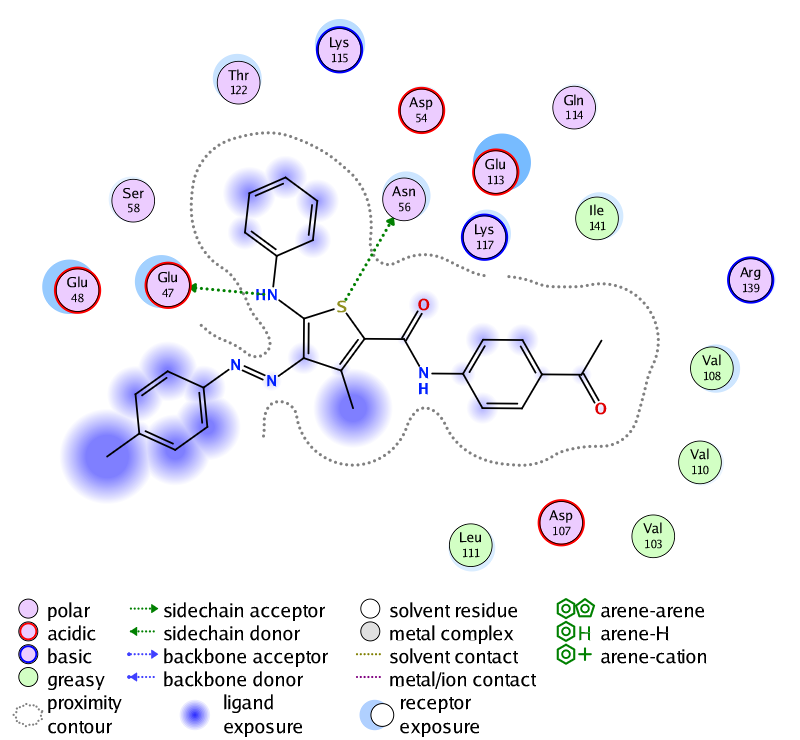 | 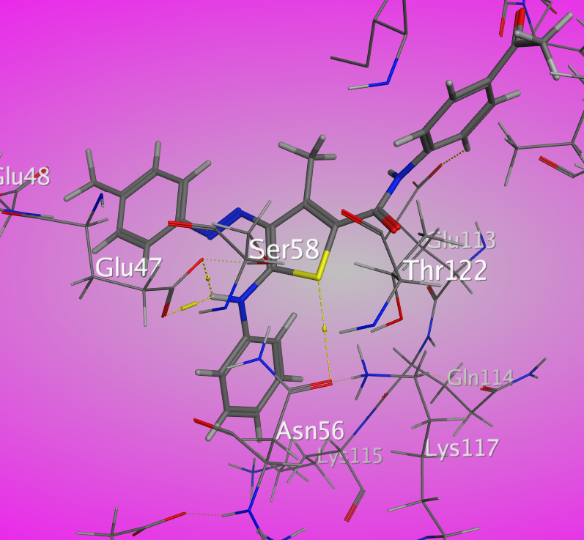 |
| --- | --- |
| 2D | 3D |

**Fig. S22** The binding interaction of 3-methylthiophene **5a** with (PDB ID: 2MLM).

| 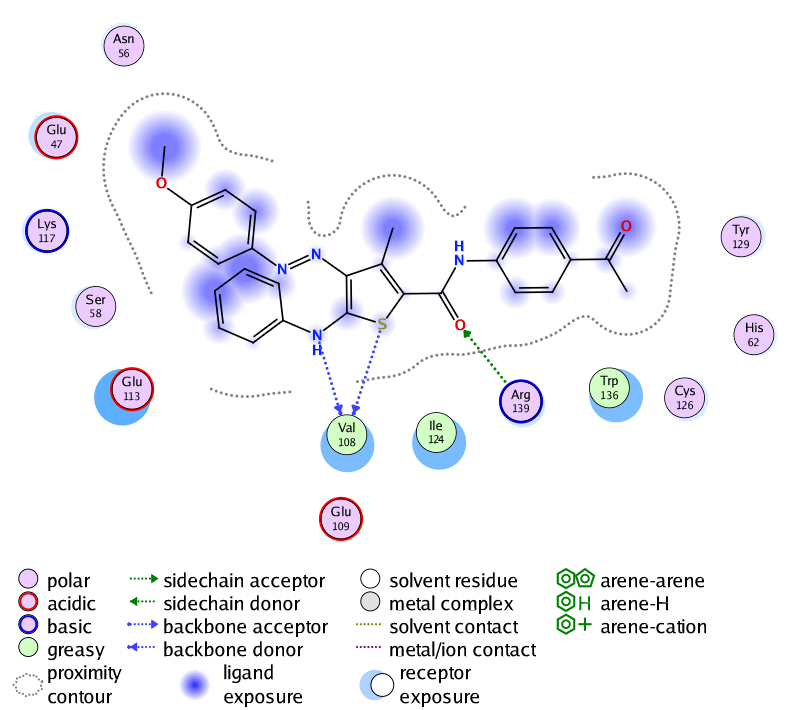 | 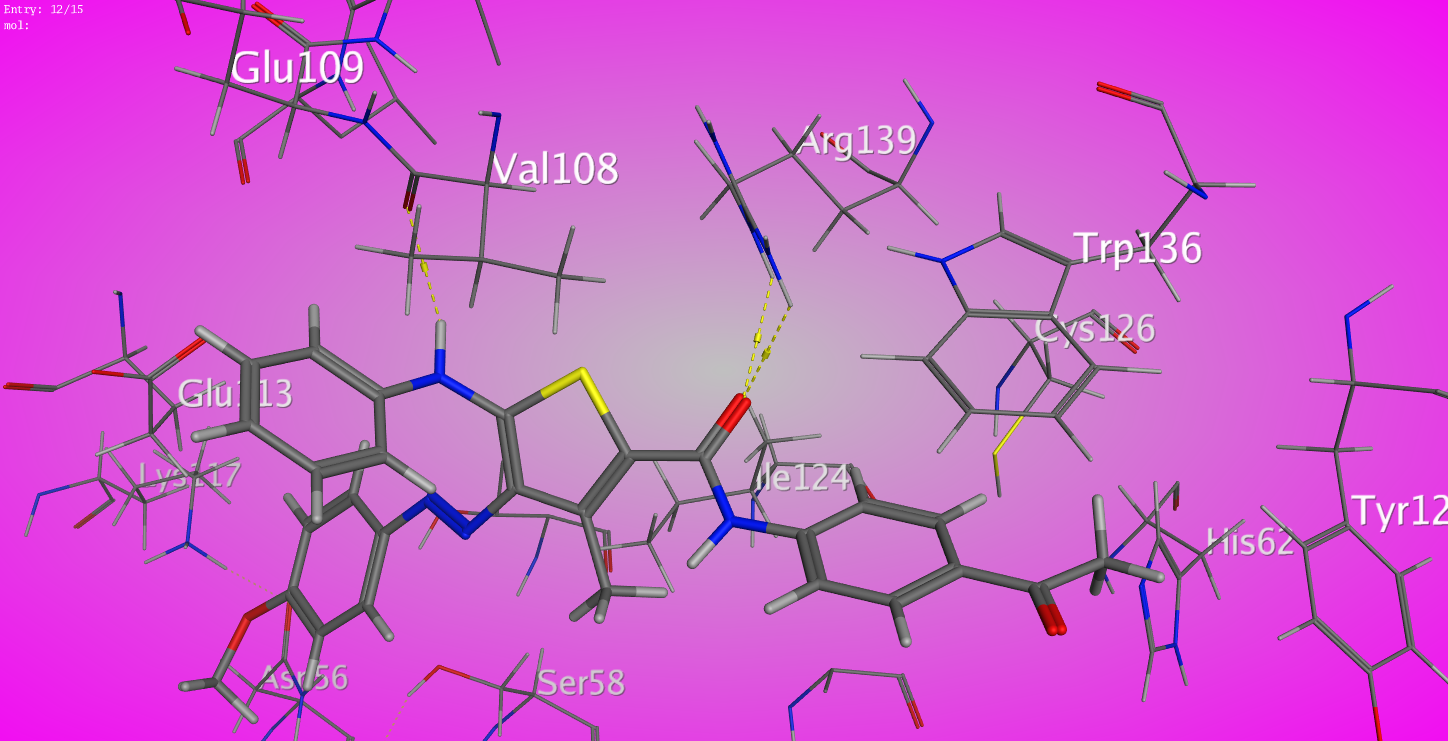 |
| --- | --- |
| 2D | 3D |

**Fig. S23** The binding interaction of 3-methylthiophene **5b** with (PDB ID: 2MLM).

| 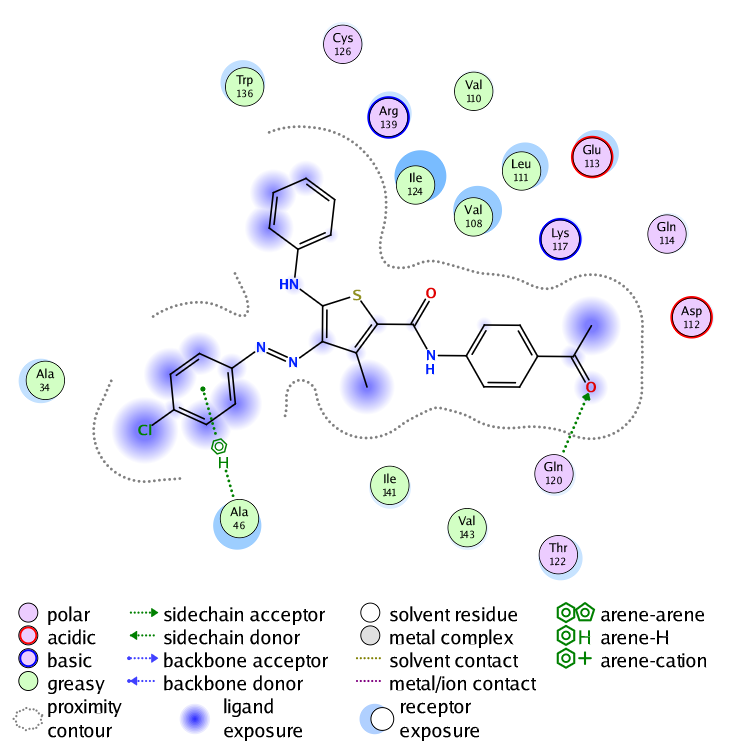 | 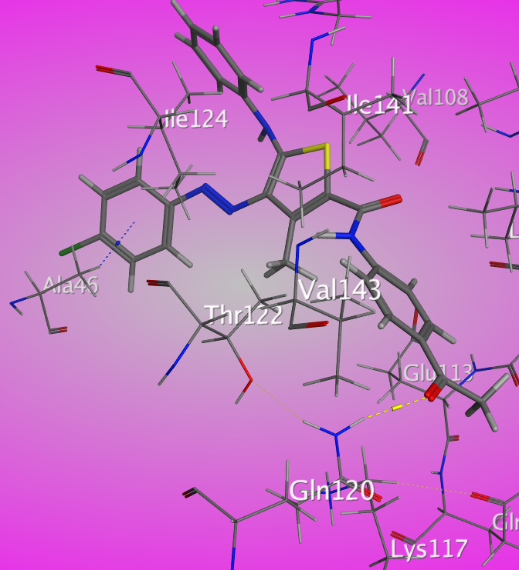 |
| --- | --- |
| 2D | 3D |

**Fig. S24** The binding interaction of 3-methylthiophene **5c** with (PDB ID: 2MLM).

| 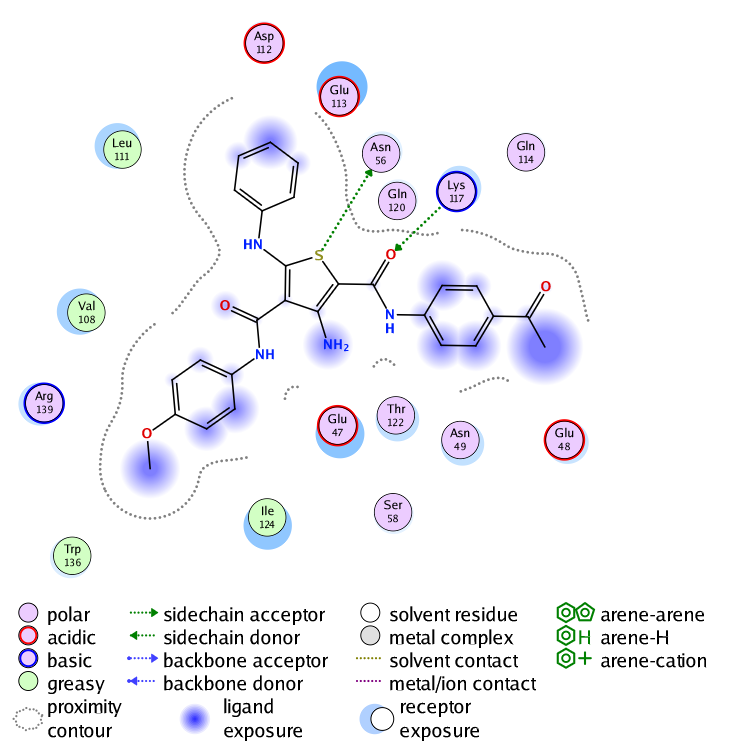 | 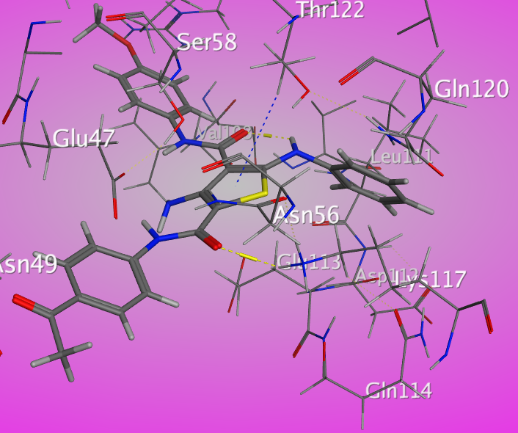 |
| --- | --- |
| 2D | 3D |

**Fig. S25** The binding interaction of 3-aminothiophene **7b** with (PDB ID: 2MLM).

| 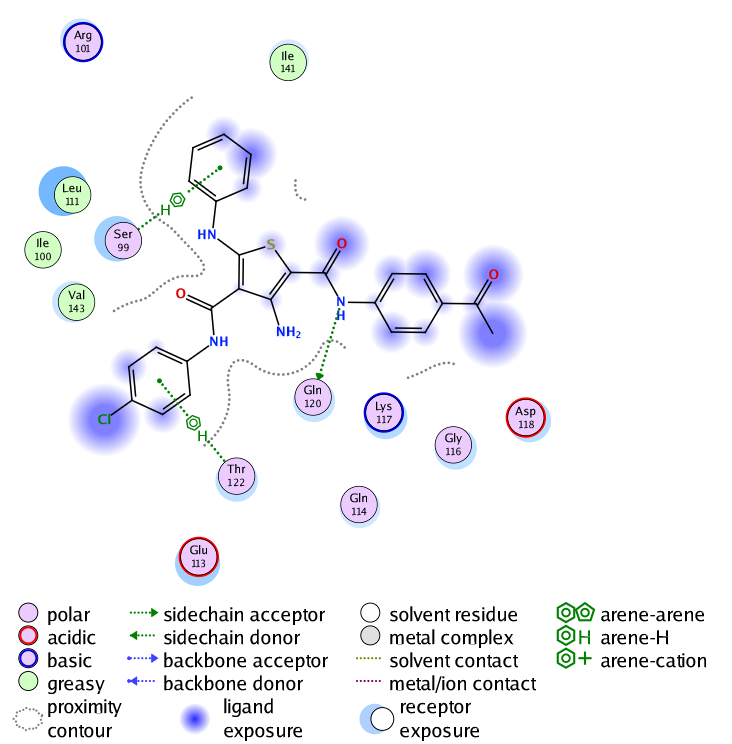 | 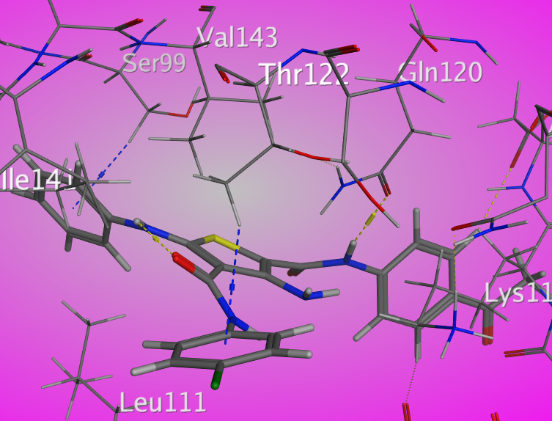 |
| --- | --- |
| 2D | 3D |

**Fig. S26** The binding interaction of 3-aminothiophene **7c** with (PDB ID: 2MLM).

| 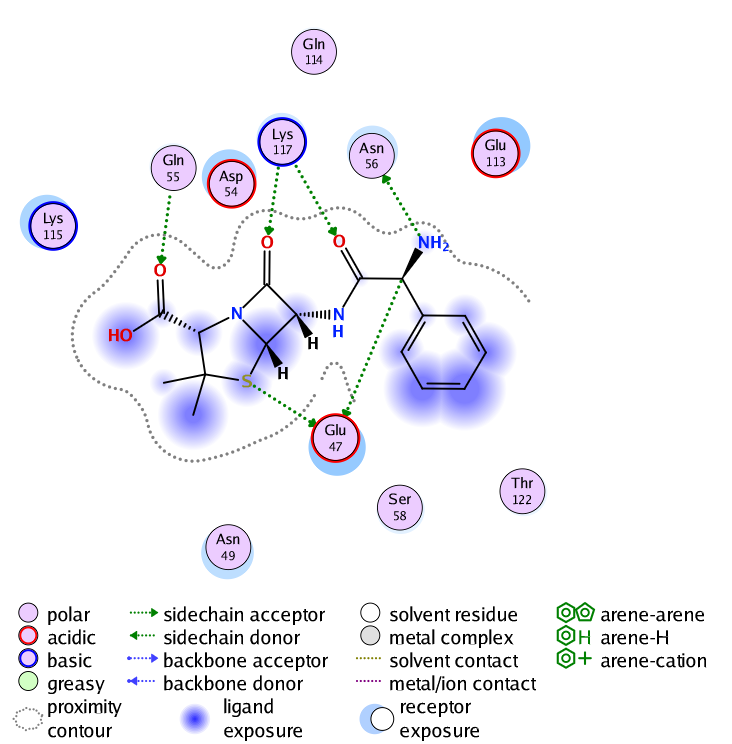 | 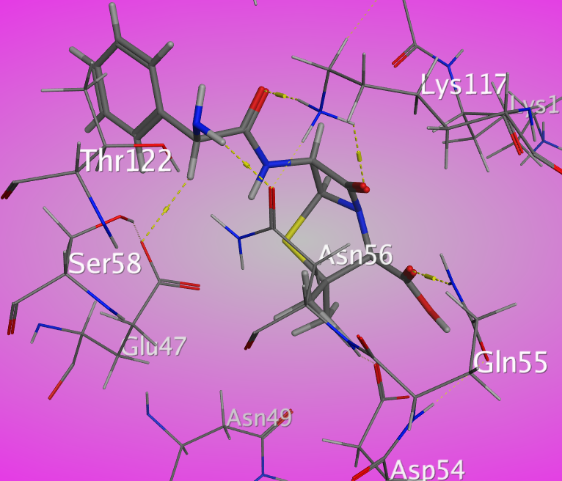 |
| --- | --- |
| 2D | 3D |

**Fig. S27** The binding interaction of ampicillin with (PDB ID: 2MLM).

**E. Coli - PDB ID: 3ZMI**

| 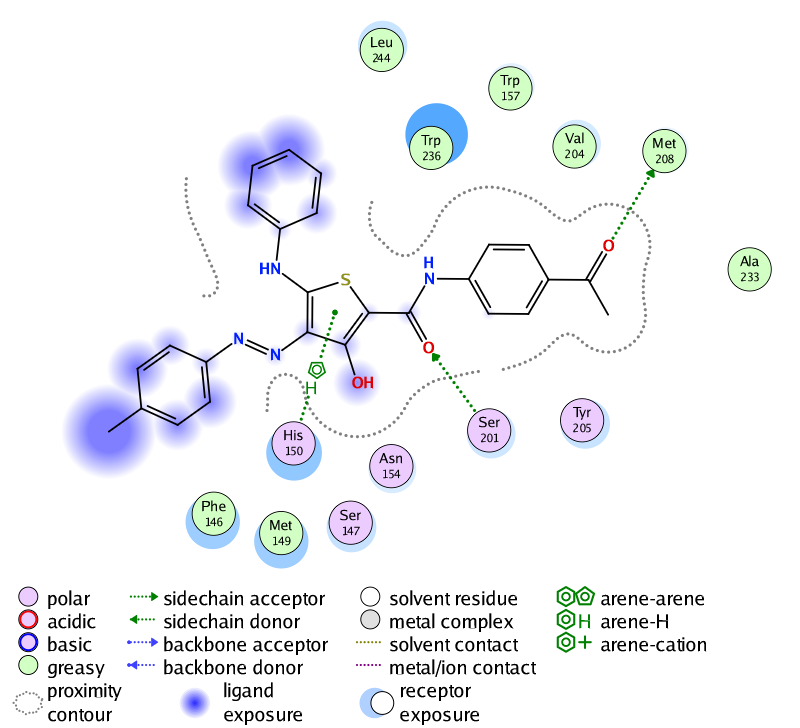 | 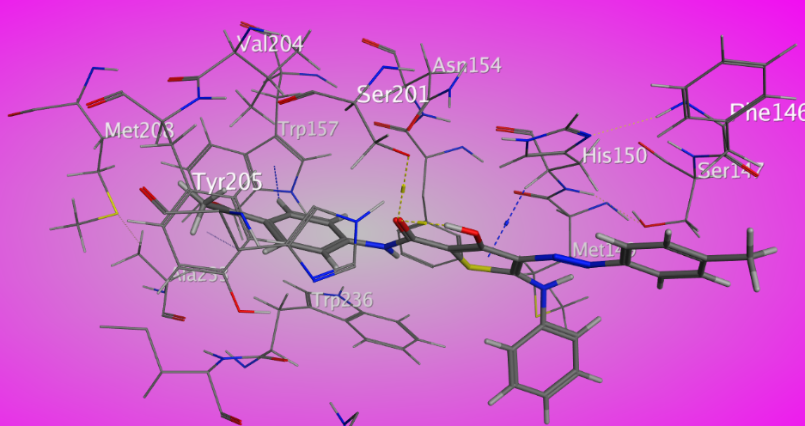 |
| --- | --- |
| 2D | 3D |

**Fig.** **S28** The binding interaction of 3-hydroxythiophene **3a** with (PDB ID: 3ZMI).

| 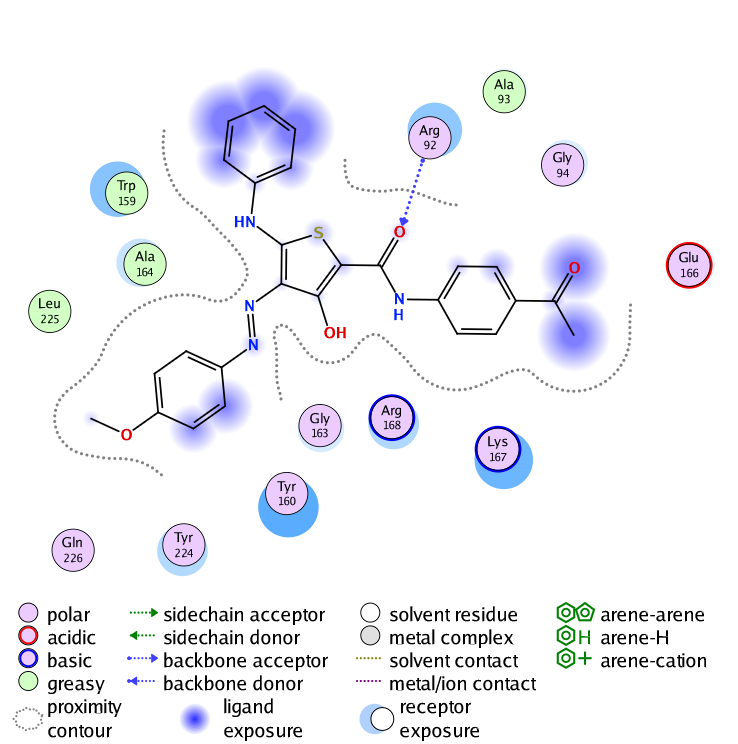 | 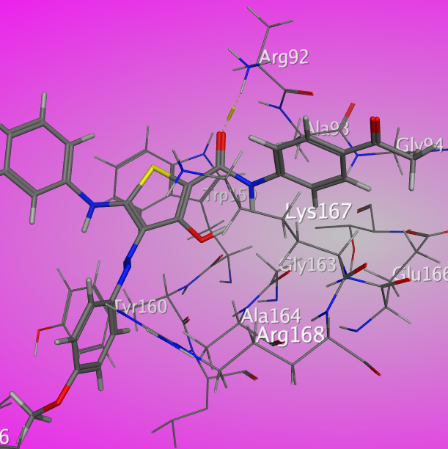 |
| --- | --- |
| 2D | 3D |

**Fig. S29** The binding interaction of 3-hydroxythiophene **3b** with (PDB ID: 3ZMI).

| 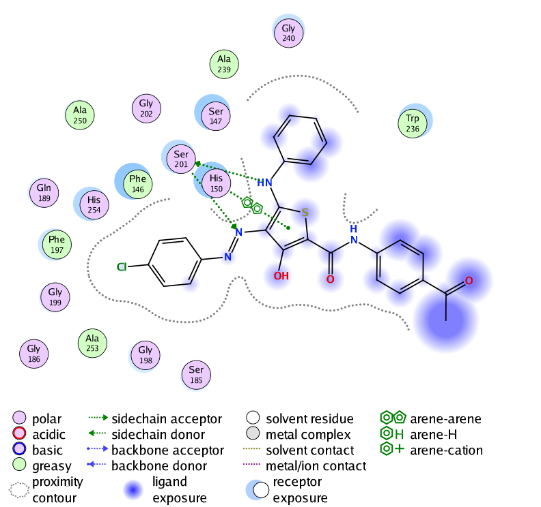 | 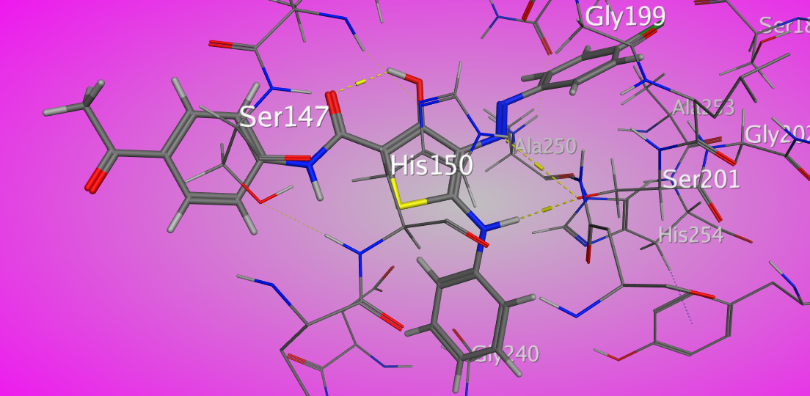 |
| --- | --- |
| 2D | 3D |

**Fig. S30** The binding interaction of 3-hydroxythiophene **3c** with (PDB ID: 3ZMI).

| 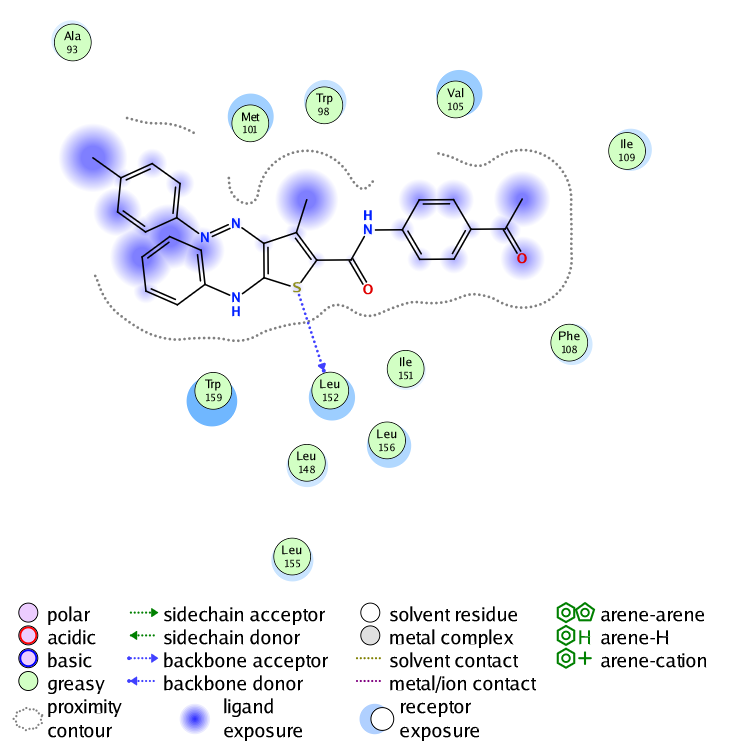 | 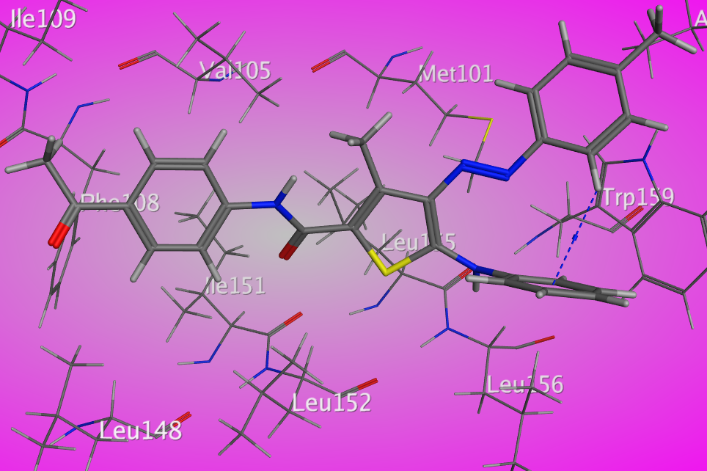 |
| --- | --- |
| 2D | 3D |

**Fig. S31** The binding interaction of 3-methylthiophene **5a** with (PDB ID: 3ZMI).

| 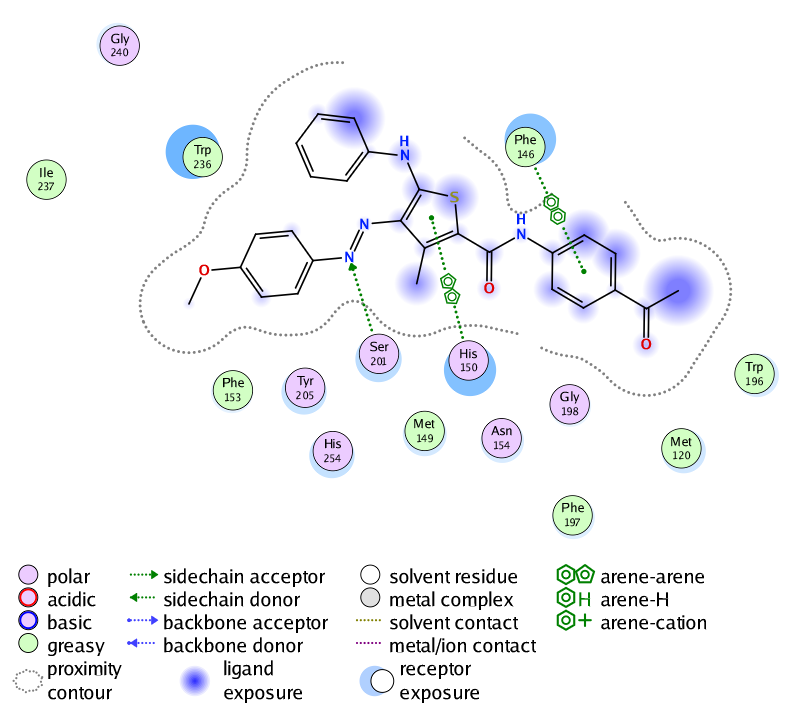 | 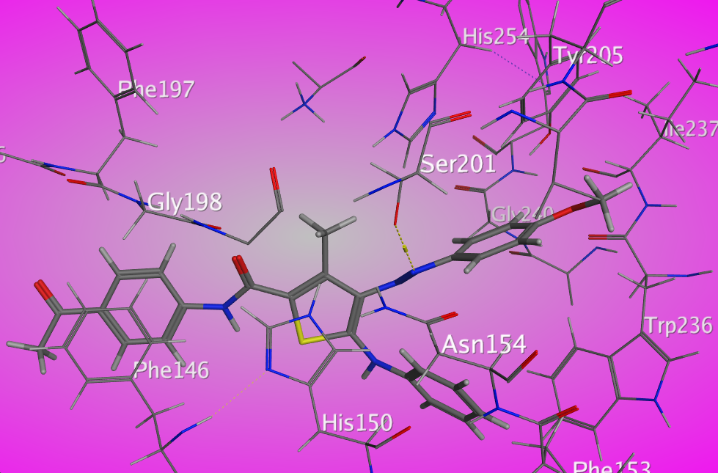 |
| --- | --- |
| 2D | 3D |

**Fig. S32** The binding interaction of 3-methylthiophene **5b** with (PDB ID: 3ZMI).

| 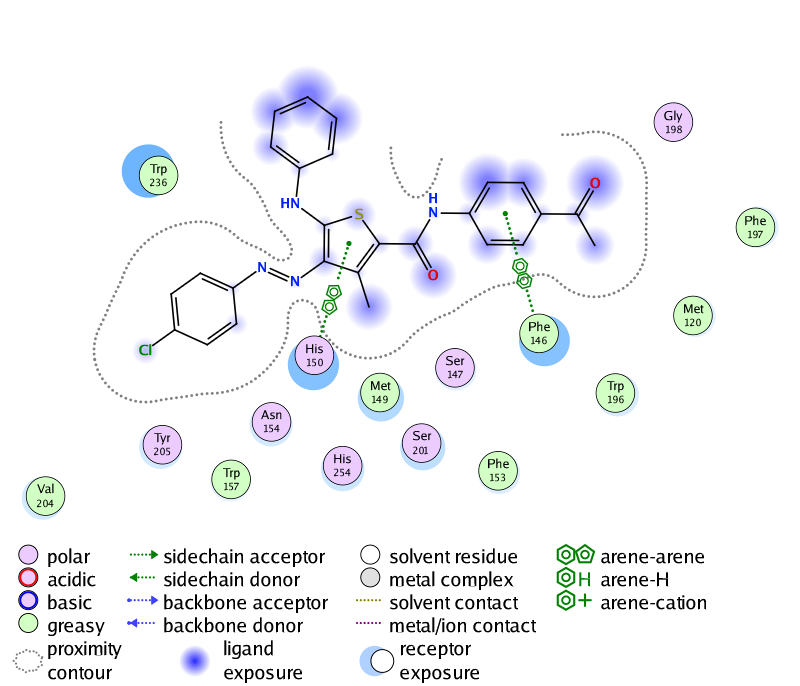 | 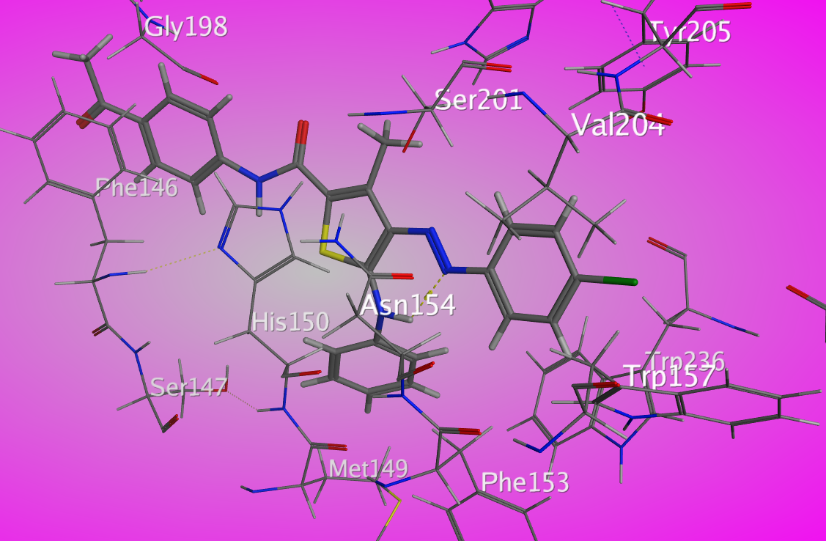 |
| --- | --- |
| 2D | 3D |

**Fig. S33** The binding interaction of 3-methylthiophene **5c** with (PDB ID: 3ZMI).

| 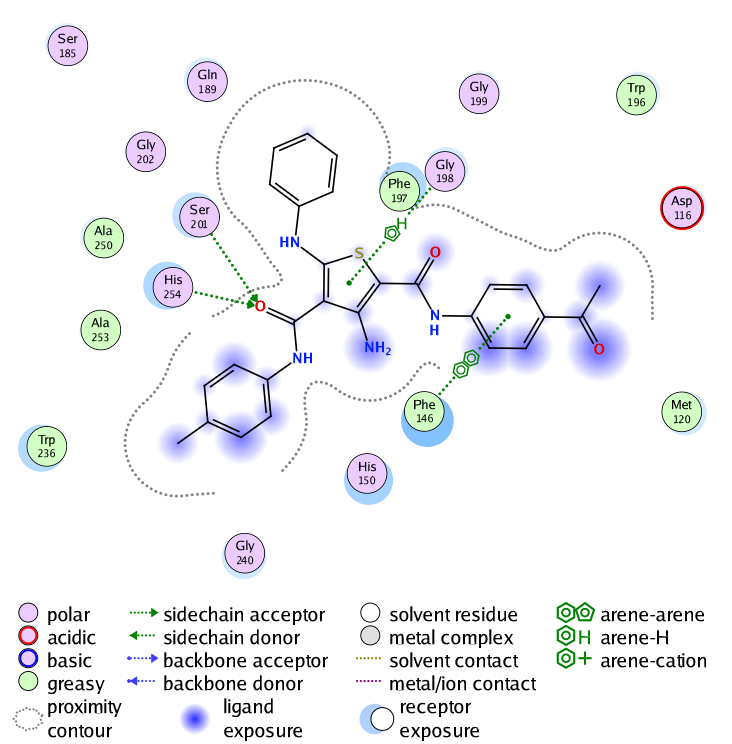 | 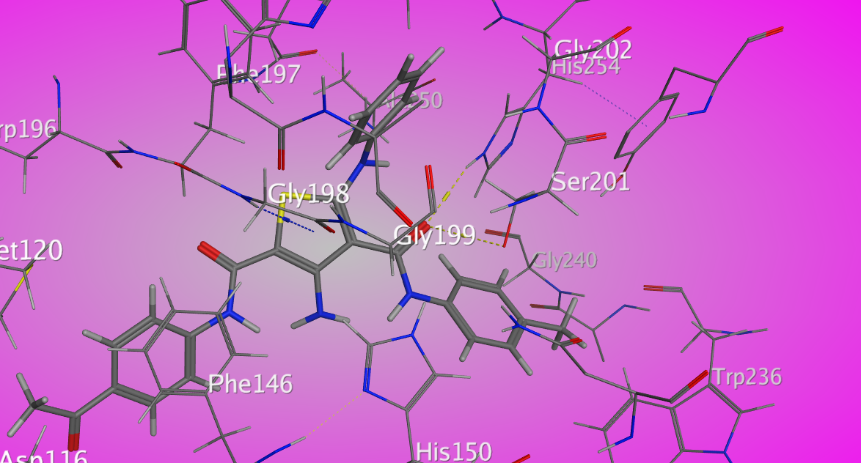 |
| --- | --- |
| 2D | 3D |

**Fig. S34** The binding interaction of 3-aminothiophene **7a** with (PDB ID: 3ZMI).

| 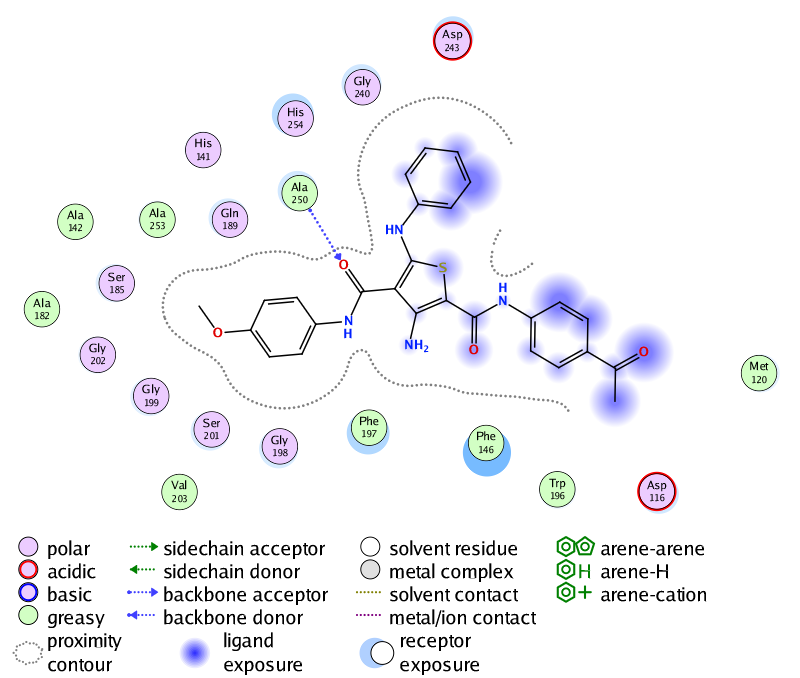 | 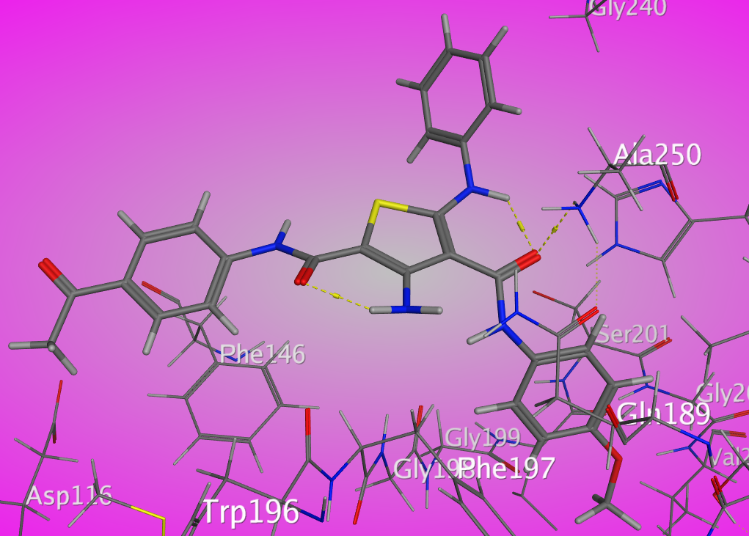 |
| --- | --- |
| 2D | 3D |

**Fig. S35** The binding interaction of 3-aminothiophene **7b** with (PDB ID: 3ZMI).

| 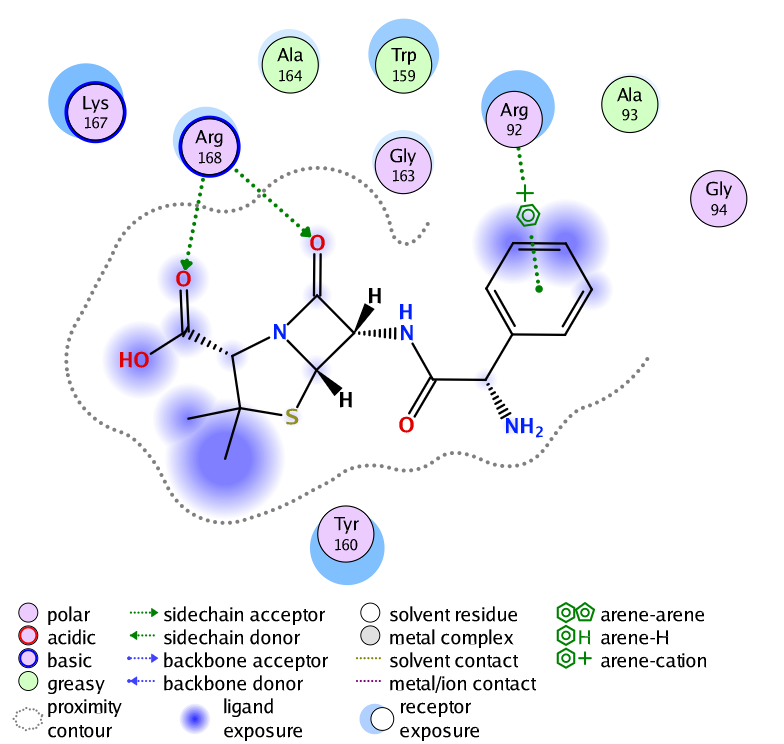 | 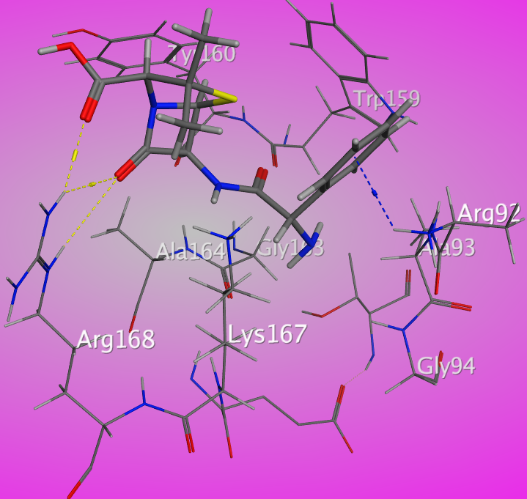 |
| --- | --- |
| 2D | 3D |

**Fig. S36** The binding interaction of with ampicillin (PDB ID: 3ZMI).

**B. subtilis - PDB ID: 4d3v**

| 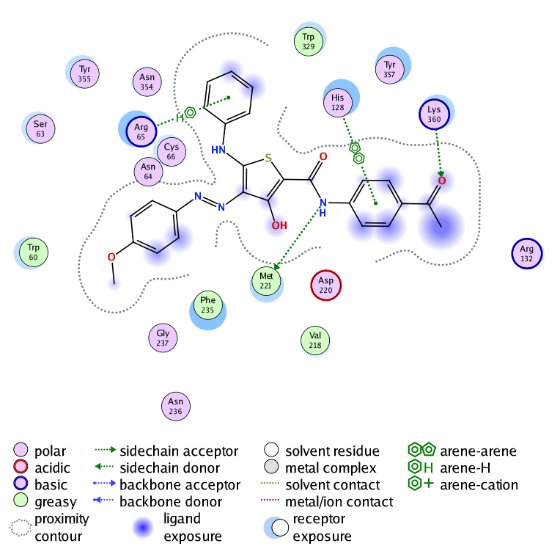 | 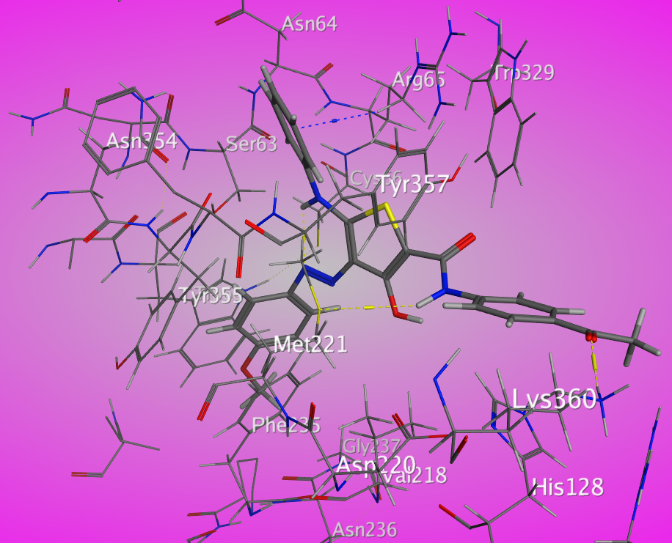 |
| --- | --- |
| 2D | 3D |

**Fig. S37** The binding interaction of 3-hydroxythiophene **3b** with (PDB ID: 4d3v).

| 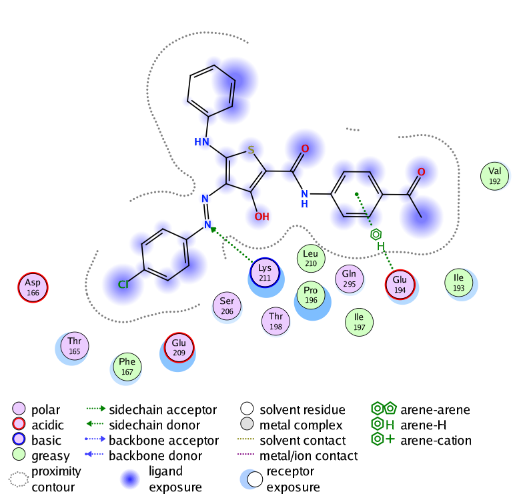 | 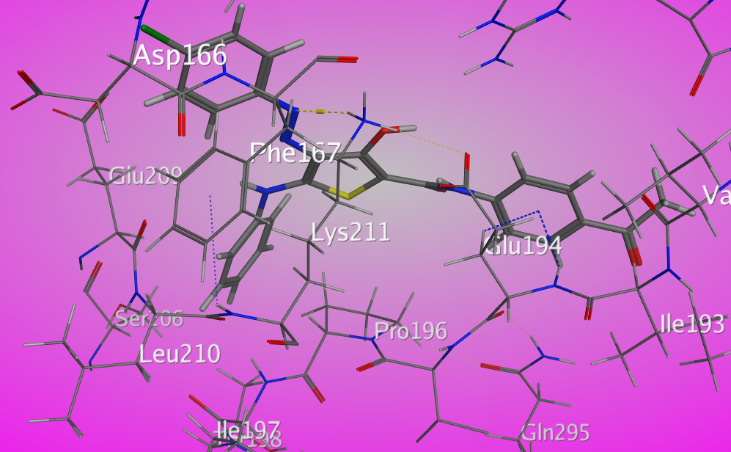 |
| --- | --- |
| 2D | 3D |

**Fig. S38** The binding interaction of 3-hydroxythiophene **3c** with (PDB ID: 4d3v).

| 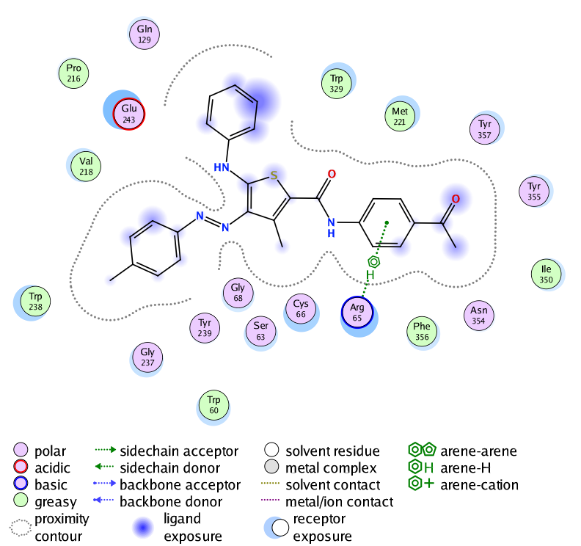 | 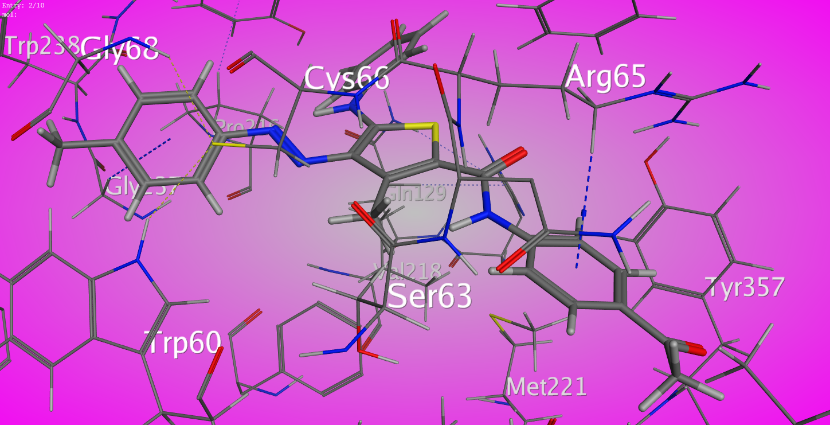 |
| --- | --- |
| 2D | 3D |

**Fig. S39** The binding interaction of 3-methylthiophene **5a** with (PDB ID: 4d3v).

| 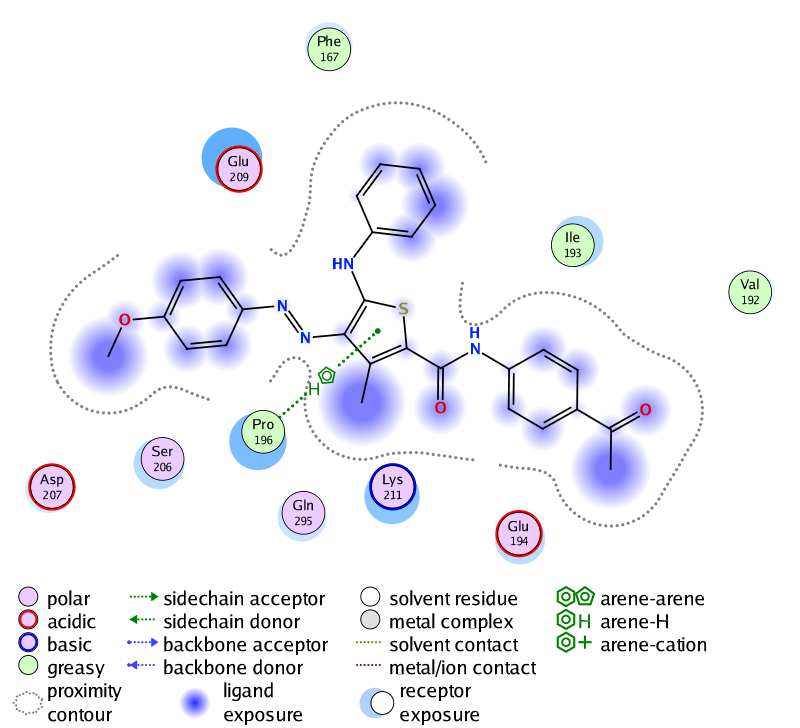 | 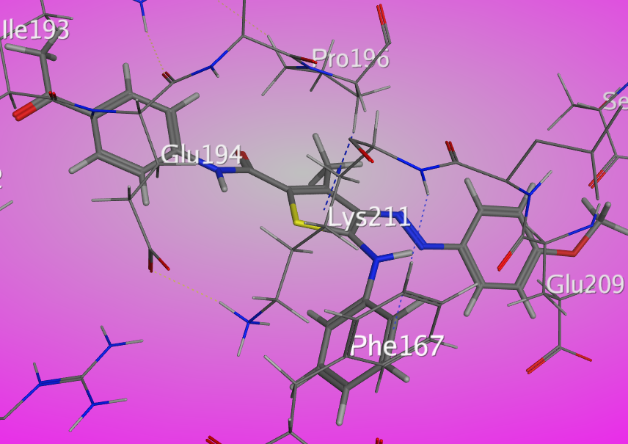 |
| --- | --- |
| 2D | 3D |

**Fig. S40** The binding interaction of 3-methylthiophene **5b** with (PDB ID: 4d3v).

| 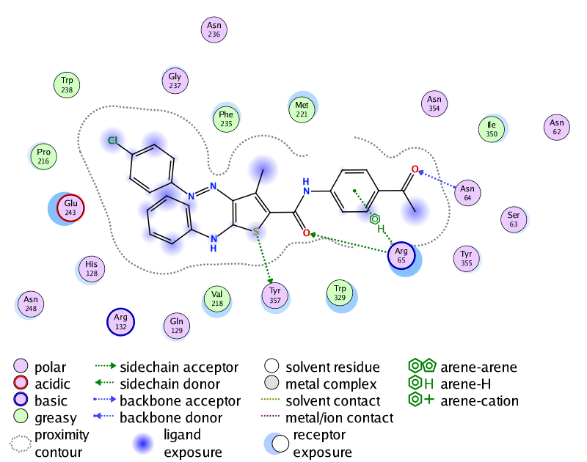 | 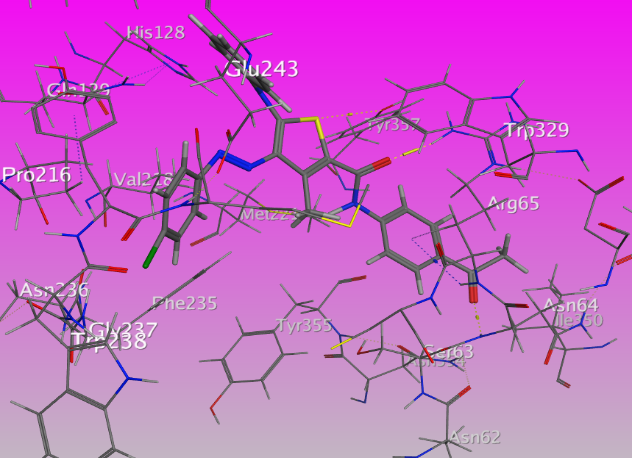 |
| --- | --- |
| 2D | 3D |

**Fig. S41** The binding interaction of 3-methylthiophene **5c** with (PDB ID: 4d3v).

| 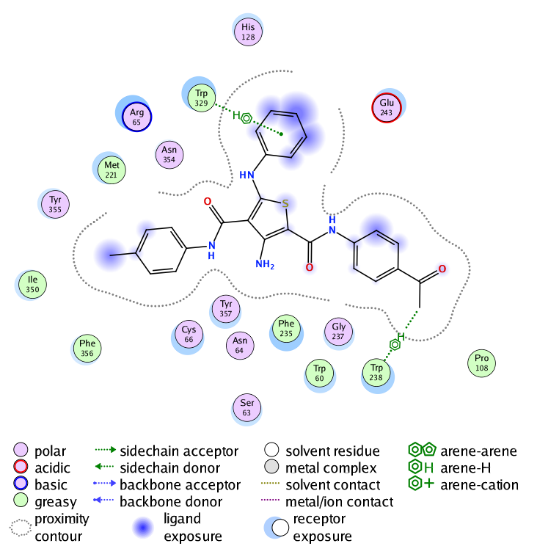 | 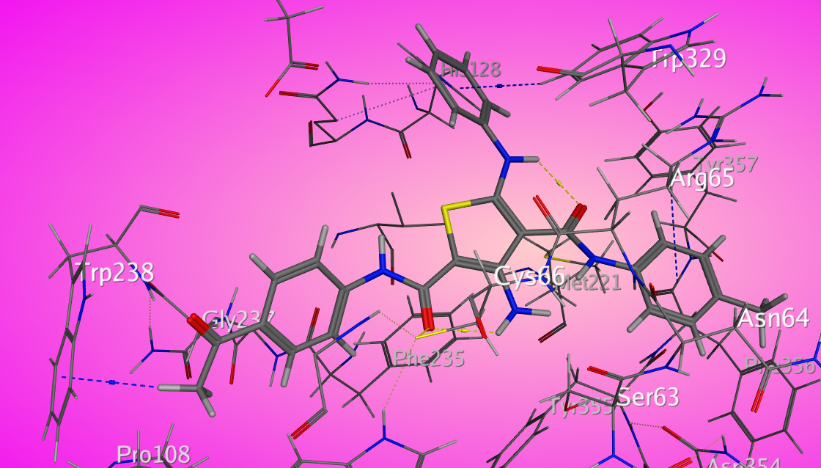 |
| --- | --- |
| 2D | 3D |

**Fig. S42** The binding interaction of 3-aminothiophene **7a** with (PDB ID: 4d3v).

| 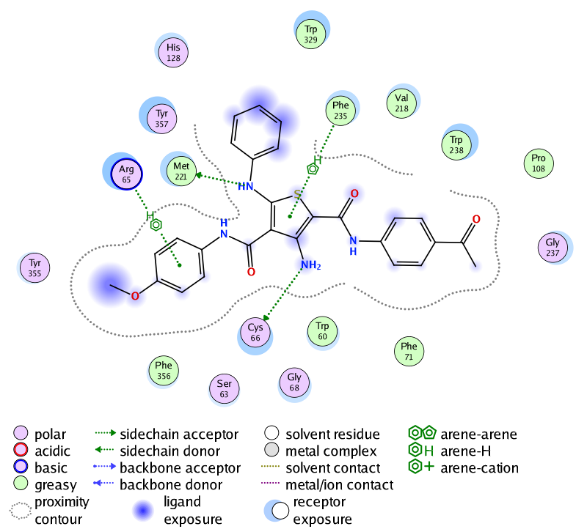 | 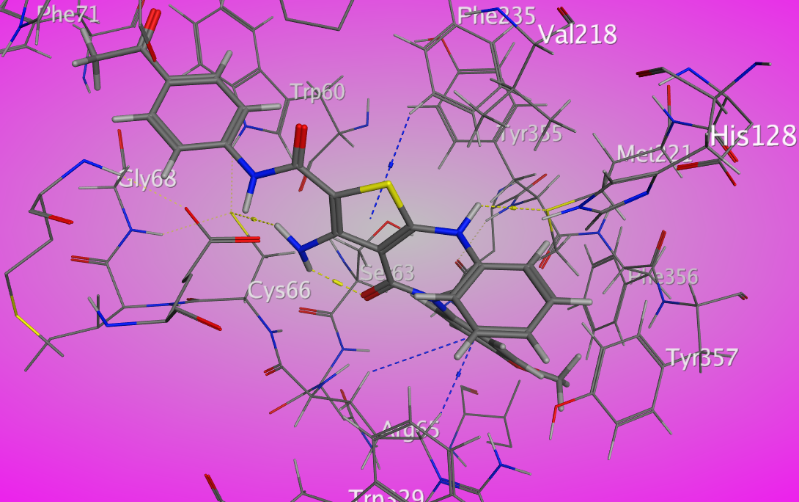 |
| --- | --- |
| 2D | 3D |

**Fig. S43** The binding interaction of 3-aminothiophene **7b** with (PDB ID: 4d3v).

| 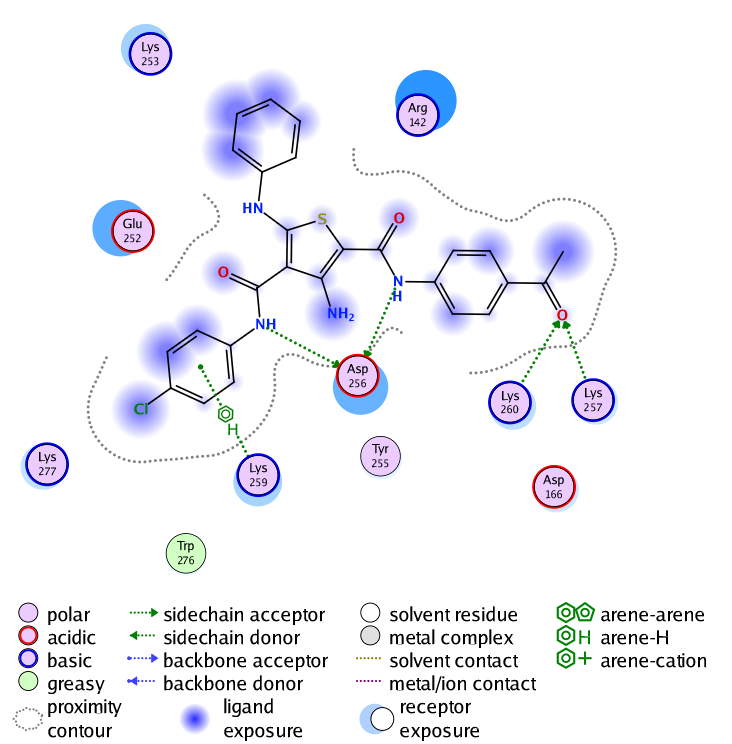 | 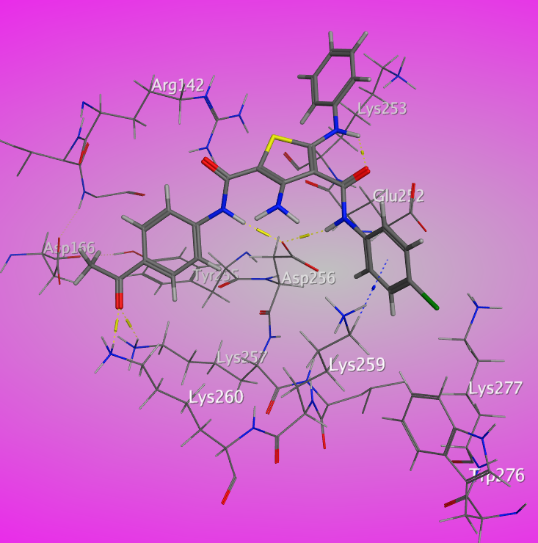 |
| --- | --- |
| 2D | 3D |

**Fig. S44** The binding interaction of 3-aminothiophene **7c** with (PDB ID: 4d3v).

| 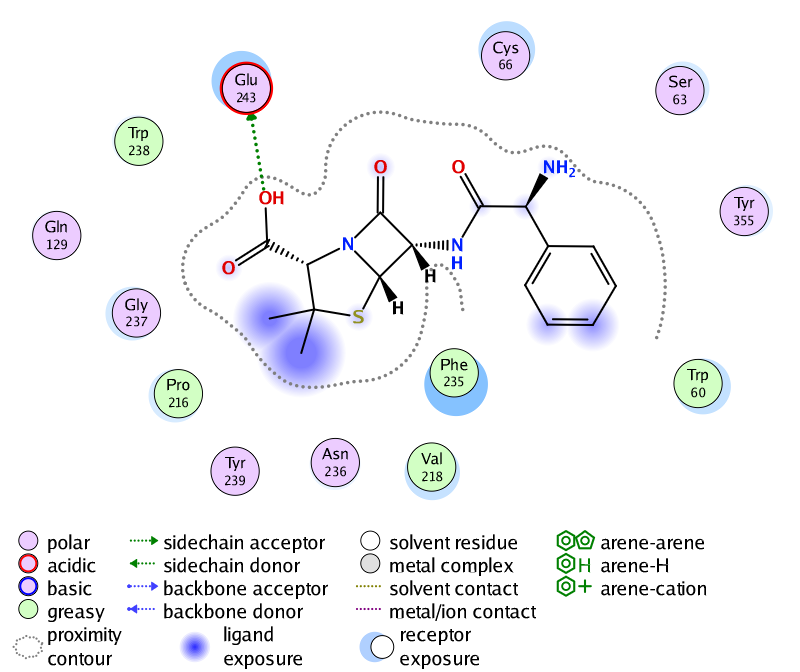 | 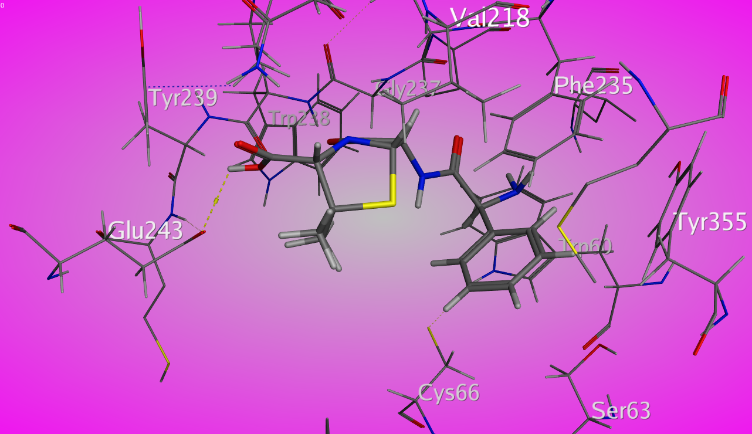 |
| --- | --- |
| 2D | 3D |

**Fig. S45** The binding interaction of with ampicillin (PDB ID: 4d3v).

**Spectral Analyses**

**
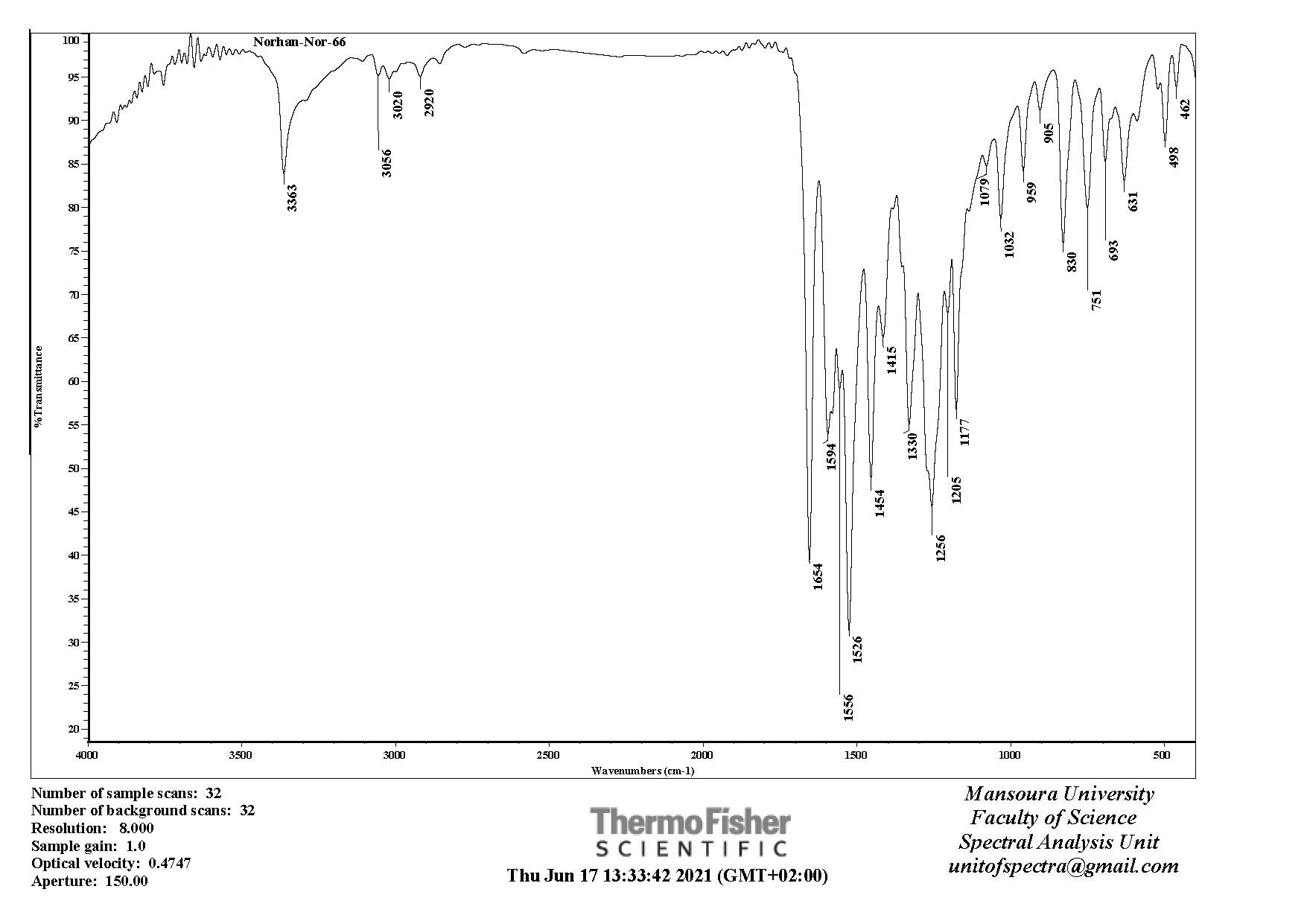
**

**Figure S46: IR spectrum of compound 3a**

**
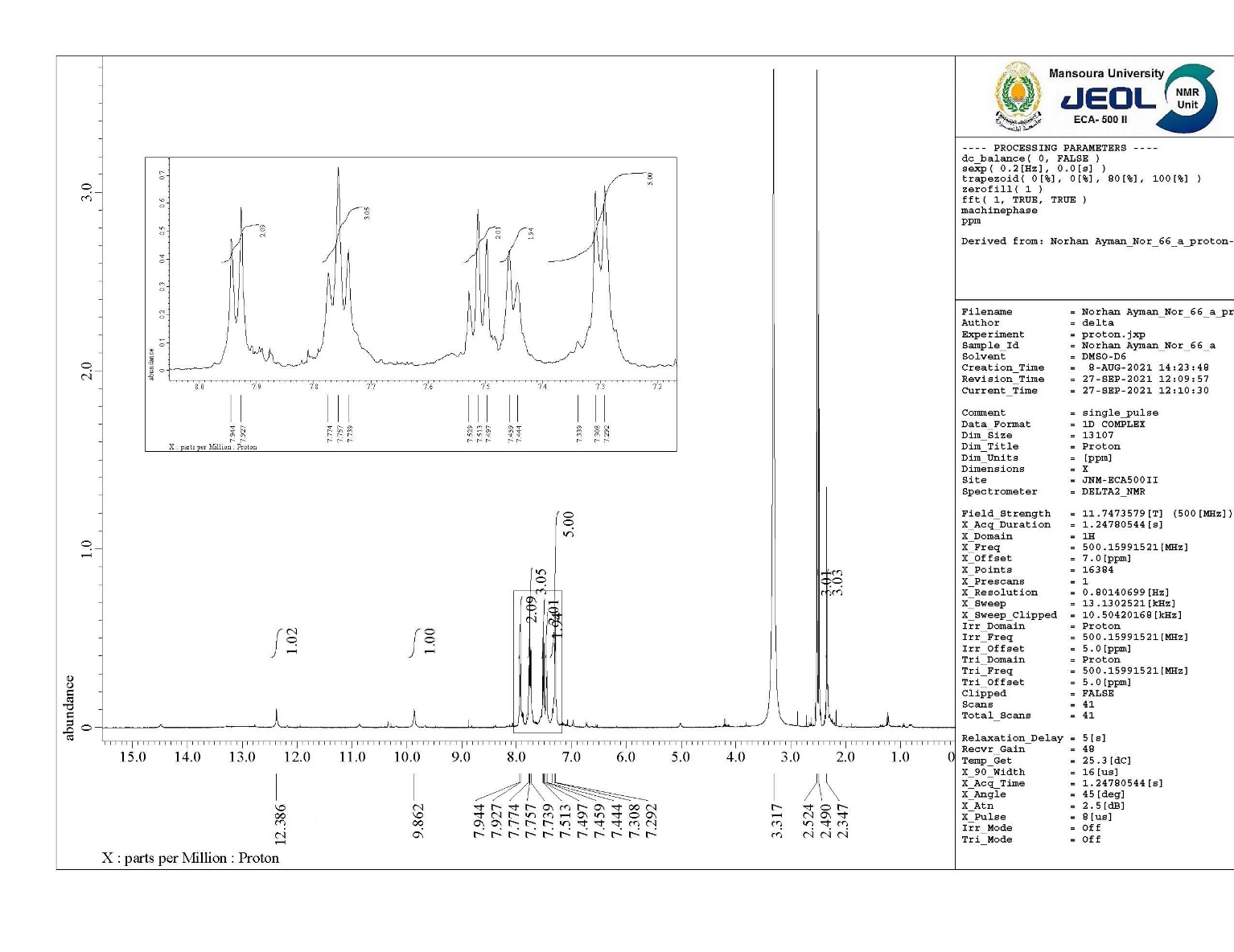
**

**Figure S47: ^1^H NMR spectrum of compound 3a**

**
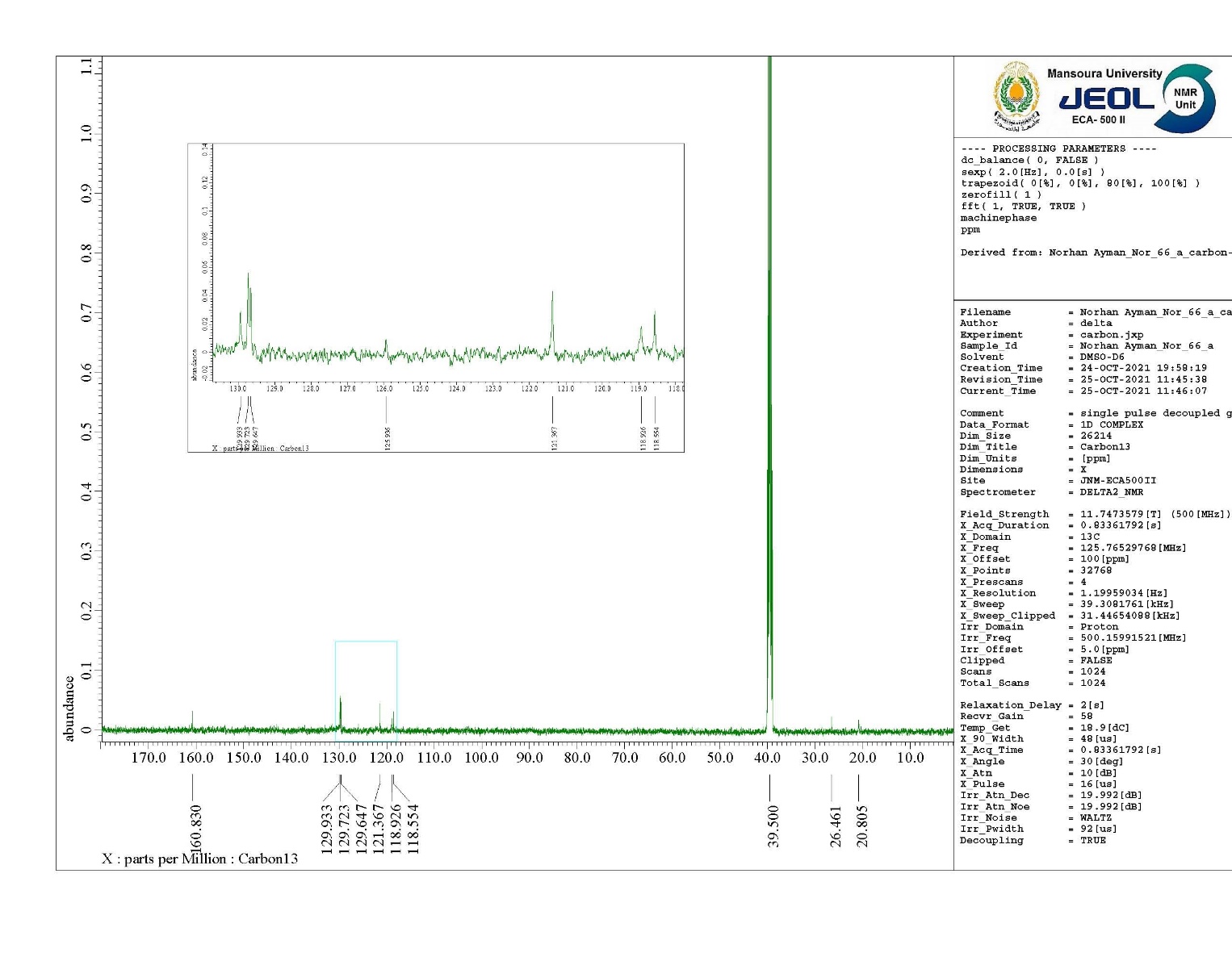
**

**Figure S48: ^13^C NMR spectrum of compound 3a**

**Figure S49: IR spectrum of compound 3b**

**Figure S50: ^1^H NMR spectrum of compound 3b**

**Figure S51: ^13^C NMR spectrum of compound 3b**

**Figure S52: IR spectrum of compound 3c**

**Figure S53: ^1^H NMR spectrum of compound 3c**

**Figure S54: IR spectrum of compound 5a**

**Figure S55: ^1^H NMR spectrum of compound 5a**

**Figure S56: ^13^C NMR spectrum of compound 5a**

**Figure S57: IR spectrum of compound 5b**

**Figure S58: ^1^H NMR spectrum of compound 5b**

**Figure S59: ^13^C NMR spectrum of compound 5b**

**Figure S60: IR spectrum of compound 5c**

**Figure S61: ^1^H NMR spectrum of compound 5c**

**Figure S62: IR spectrum of compound 7a**

**Figure S63: ^1^H NMR spectrum of compound 7a**

**Figure S64: ^13^C NMR spectrum of compound 7a**

**Figure S65: IR spectrum of compound 7b**

**Figure S66: ^1^H NMR spectrum of compound 7b**

**Figure S67: IR spectrum of compound 7c**

**Figure S68: ^1^H NMR spectrum of compound 7c**
